# A prognostic human brain network for diffuse midline glioma

**DOI:** 10.1038/s41586-026-10631-3

**Published:** 2026-06-10

**Authors:** Jai Sidpra, Valentina Lind, Alexander L. Cohen, Frederic L. W. V. J. Schaper, Thomas J. Stone, Yura Grabovska, Asthik Biswas, Sniya Sudhakar, Francisco Sepulveda, Bruno S. Peres, Greta Veronese, Cristina Alemán-Charlet, Olumide Ogunbiyi, Kiarash Shamardani, Jiaqi Zhao, Alberto Castro Palacin, Gillian Miller, Raffaella S. Opipari, Enrico De Vita, Deborah Ridout, Suely F. Ferraciolli, Leandro T. Lucato, Jernej Avsenik, Eleonora Piccirilli, Andrés Morales-La Madrid, Jordi Muchart, Maura E. Ryan, Rajan Patel, Parthiv Haldipur, Ciaran S. Hill, Marie T. Krüger, Ludvic Zrinzo, Noor ul Owase Jeelani, Juan Pedro Martinez-Barbera, Andrew M. Donson, Kathleen Dorris, Paul S. Morgan, Alan Mackay, Humsa S. Venkatesh, Andreas Horn, Sabine Mueller, Adam L. Green, David M. Mirsky, Harith Akram, Chris Jones, Kristian Aquilina, Kshitij Mankad, Michelle Monje, Thomas S. Jacques, Michael D. Fox, Darren R. Hargrave

**Affiliations:** 1 Developmental Biology and Cancer Section, University College London Great Ormond Street Institute of Child Health, London, UK; 2 Unit of Functional Neurosurgery, University College London Queen Square Institute of Neurology, London, UK; 3 Victor Horsley Department of Neurosurgery, The National Hospital for Neurology and Neurosurgery, London, UK; 4 Center for Brain Circuit Therapeutics, Departments of Neurology, Psychiatry, Neurosurgery, and Radiology, Brigham & Women’s Hospital, Harvard Medical School, Boston, MA USA; 5 Harvard Medical School, Harvard University, Boston, MA USA; 6 Department of Neurology, Boston Children’s Hospital, Boston, MA USA; 7 Computational Radiology Laboratory, Department of Radiology, Boston Children’s Hospital, Harvard Medical School, Boston, MA USA; 8 Department of Neurology, Brigham and Women’s Hospital, Boston, MA USA; 9 Department of Histopathology, Great Ormond Street Hospital for Children NHS Foundation Trust, London, UK; 10 Centre for Children and Young People’s Cancer, Division of Cancer Biology, Institute of Cancer Research, London, UK; 11 Department of Radiology, Great Ormond Street Hospital for Children NHS Foundation Trust, London, UK; 12 Department of Radiology, Clínica Alemana de Santiago, Facultad de Medicina Clínica Alemana, Universidad del Desarrollo, Santiago, Chile; 13 Department of Neuroradiology, Institute of Neurosurgery Dr. Alfonso Asenjo, National Health Service, Santiago, Chile; 14 Neuroradiology Section, Hospital das Clinicas (HCFMUSP), Faculdade de Medicina, Universidade de São Paulo, Sao Paulo, Brazil; 15 Genetics and Genomic Medicine Section, University College London Great Ormond Street Institute of Child Health, London, UK; 16 Specialist Integrated Haematology and Malignancy Diagnostic Service—Acquired Genomics, Great Ormond Street Hospital for Children NHS Foundation Trust, London, UK; 17 Department of Neurology and Neurological Sciences, Stanford University, Stanford, CA USA; 18 Department of Neuroscience, Neuroradiology Unit, San Bortolo Hospital, Vicenza, Italy; 19 MR Physics Group, Department of Radiology, Great Ormond Street Hospital for Children NHS Foundation Trust, London, UK; 20 Developmental Imaging and Biophysics, Developmental Neurosciences, University College London Great Ormond Street Institute of Child Health, London, UK; 21 Population, Policy, and Practice Programme, University College London Great Ormond Street Institute of Child Health, London, UK; 22 Clinical Institute of Radiology, University Medical Centre Ljubljana, Ljubljana, Slovenia; 23 Department of Radiology, Faculty of Medicine, University of Ljubljana, Ljubljana, Slovenia; 24 Department of Imaging, Oncological and Advanced Neuroradiology Unit, Bambino Gesù Children’s Hospital IRCCS, Rome, Italy; 25 Department of Neuroscience, Imaging and Clinical Sciences, University G. d’Annunzio of Chieti-Pescara, Chieti, Italy; 26 Neuro-Oncology Unit, Paediatric Cancer Center, Sant Joan de Déu Barcelona Children’s Hospital, University of Barcelona, Esplugues de Llobregat, Spain; 27 Department of Radiology, Sant Joan de Déu Barcelona Children’s Hospital, University of Barcelona, Esplugues de Llobregat, Spain; 28 Pediatric Computational Imaging Center (PeCIC), Institut de Recerca Sant Joan de Déu (IRSJD), Esplugues de Llobregat, Spain; 29 Department of Medical Imaging, Ann & Robert H. Lurie Children’s Hospital of Chicago, Northwestern University Feinberg School of Medicine, Chicago, IL USA; 30 Department of Pediatric Radiology, Texas Children’s Hospital, Baylor College of Medicine, Houston, TX USA; 31 Center for Integrative Brain Research, Seattle Children’s Research Institute, Seattle, WA USA; 32 Samantha Dickson Brain Cancer Unit, University College London Cancer Institute, London, UK; 33 Department of Functional Neurosurgery, Albert-Ludwigs-Universität Freiburg, Freiburg im Breisgau, Germany; 34 Department of Neurosurgery, Great Ormond Street Hospital for Children NHS Foundation Trust, London, UK; 35 Morgan Adams Foundation Pediatric Brain Tumour Research Program, Department of Pediatrics, University of Colorado Anschutz School of Medicine, Aurora, CO USA; 36 Neuro-Oncology Program, Center for Cancer and Blood Disorders, Children’s Hospital Colorado, Aurora, CO USA; 37 Department of Medical Physics and Clinical Engineering, Nottingham University Hospitals, Nottingham, UK; 38 School of Medicine, University of Nottingham, Nottingham, UK; 39 NIHR Nottingham Biomedical Research Centre, Nottingham University Hospitals, Nottingham, UK; 40 Institute for Network Stimulation, Department of Stereotactic and Functional Neurosurgery, University Hospital Cologne, Cologne, Germany; 41 Department of Neurology, Neurosurgery and Pediatrics, University of California, San Francisco, San Francisco, CA USA; 42 Department of Neuroradiology, Children’s Hospital Colorado, University of Colorado School of Medicine, Aurora, CO USA; 43 Howard Hughes Medical Institute, Stanford, CA USA; 44 Department of Psychiatry, Brigham and Women’s Hospital, Boston, MA USA; 45 Department of Haematology and Oncology, Great Ormond Street Hospital for Children NHS Foundation Trust, London, UK

**Keywords:** Paediatric cancer, Cancer in the nervous system, CNS cancer, Cancer genomics, Cancer imaging

## Abstract

Diffuse midline gliomas (DMGs) are near-universally lethal tumours of the childhood central nervous system^1,2^. In animal models, DMGs form brain-wide integrated networks through neuron-to-glioma synapses^3–6^ and glioma-to-glioma gap junctional coupling^3^. This extensive connectivity robustly promotes the growth and invasion of DMG^3–9^ and other glial malignancies^10–12^ through paracrine mechanisms and direct neuron-to-glioma synapses. However, the organization and clinical implications of these connections in the living human brain remain to be elucidated. Here, we develop tumour network mapping to compute the brain-wide connectivity profile of DMG, defining a conserved brain network across pontine and thalamic DMG associated with patient short-term survival (DMG network). Tumour functional connectivity with the DMG network was independently predictive of patient overall survival across two external validation cohorts. Tumour growth mapped to DMG network-specific trajectories and peak in-network neurometabolic changes across development spatiotemporally aligned with the peak age incidence of DMG. Analyses of single-nucleus RNA sequencing data confirmed diverse synaptic gene enrichment in high-connectivity DMG. Strikingly, incidental surgical resection of high-connectivity thalamic DMG tissue conferred a significant survival advantage. Collectively, these data define a conserved and prognostically important brain network in children with DMG, consistent with the hypothesis that DMGs exploit otherwise healthy brain circuits to promote tumour growth.

## Abstract (web summary)

Tumour network mapping of diffuse midline glioma (DMG) defines a conserved and prognostically important brain network in children with DMG, consistent with the hypothesis that DMGs exploit otherwise healthy brain circuits to promote tumour growth.

## Main

Diffuse intrinsic pontine glioma (DIPG) and other diffuse midline gliomas (DMGs) are the leading cause of solid-tumour-related death in children which despite clinical trials over the past two decades, retain notorious therapeutic resistance and near-universal lethality^2,13^. A major clinical challenge limiting the efficacy of conventional therapeutic approaches, and recognized in original definitions of the disease, is the extensive infiltration of healthy brain parenchyma^14^. This distributed, brain-wide pattern of tumour progression, including to distant brain regions such as the frontal and temporal poles, is insufficiently explained by current models of tumour evolution^15–17^. Recent work has established DMG integration and communication with otherwise healthy neural circuits through both paracrine signalling (brain-derived neurotrophic factor (BDNF) and neuroligin-3 (NLGN3))^4,7,8^ and bona fide, electrophysiologically functional neuron-to-glioma synapses across a diverse neurotransmitter repertoire, including glutamatergic, calcium-permeable AMPA receptor-mediated^3,4^; cholinergic M1–M3 receptor-mediated^5^; and GABAergic GABA_A_ receptor-mediated^6^ neuron-to-glioma synapses. In animal models, depolarization of glioma cell membranes drives tumour growth through voltage-dependent mechanisms that remain to be fully elucidated^3,4^. Neuronal cell somata local to or distant from and projecting onto malignant cells engage brain-wide neural populations in glioma circuits, activity from which acts as a putative driver of tumour progression^5,9,18^. For example, long-range cholinergic projections from the midbrain pedunculopontine and laterodorsal tegmental nuclei, respectively, promote the circuit-specific growth of pontine and thalamic DMG in preclinical models^5^. Although the impact of other neuromodulatory neurons remains to be fully explored, early preclinical reports suggest that serotonergic projections from the dorsal and median raphe promote the circuit-specific growth of pontine, thalamic and cortical DMG, while noradrenergic projections from the locus coeruleus also promote DMG growth^9,18^.

Thus far, attempts to map the brain-wide neural circuits implicated in tumour growth have been restricted to mouse models, which offer an incomplete representation of human-specific neurobiology and therapeutic relevance. Further, the distinct spatiotemporal pattern of DMG incidence, such that pontine and thalamic tumours peak in incidence during early childhood and early adolescence, respectively, suggests tumour exploitation of an underlying neurodevelopmental process. Here, using patient clinical data and human paediatric connectomic data, we define the spatial topography of brain network connections associated with short-term survival in children with DMG and delineate circuit-specific trajectories of DMG tumour growth.

## Tumour network mapping

**Fig. 1:**
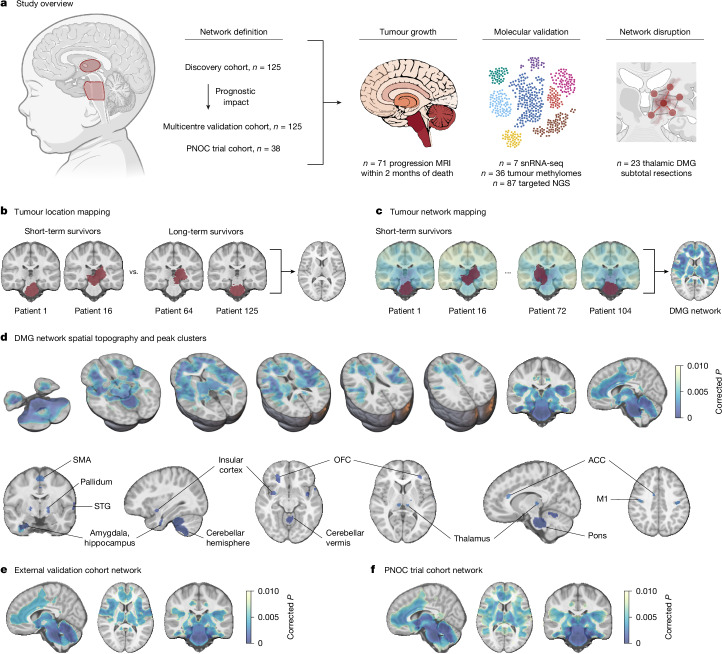
Pontine and thalamic DMG map to a conserved, prognostic brain network. **a**, Overview of the study methodology. NGS, next-generation sequencing. **b**, The tumour locations of short-term and long-term survivors of pontine DMG/DIPG and thalamic DMG from the discovery cohort (*n* = 125; left, red) were compared using VLSM; however, tumour locations did not explain the observed variance in patient overall survival (right; *P*_FWE_ > 0.05). **c**, Tumour network mapping was performed to compute the network of brain regions connected to each patient’s tumour location using resting-state functional connectivity data from 1,000 healthy children aged 9.0 ± 0.2 years (the human connectome). Tumour network maps across patients with short-term overall survival (*n* = 106) were statistically compared to identify brain regions that are significantly associated with shorter patient overall survival (right; network peaks are shown in dark blue), hereafter termed the DMG network. **d**, DMGs exhibit a specific pattern of brain functional connectivity associated with patient short-term overall survival. This includes strong positive connectivity with the pons, thalamus (centromedian, medial pulvinar and ventral intermediate nuclei), motor cortex (M1 and supplementary motor area (SMA)), orbitofrontal (OFC) and insular cortices, limbic system (amygdala, hippocampus, basal ganglia and anterior cingulate cortex (ACC)), superior temporal gyrus (STG) and cerebellum (lobules I–V, VIII and X). **e**,**f**, Identical tumour network mapping performed across two independent external validation cohorts (external validation cohort network (**e**) and PNOC trial cohort network (**f**)) reproduced DMG network topography. *n* = 125 children with primary pontine DMG/DIPG and thalamic DMG, *n* = 108 short-term survivors (**e**); *n* = 38 children with biopsy-confirmed pontine DMG, *n* = 29 short-term survivors (**f**). For **e**, DMG network whole brain spatial *ρ* = 0.952, label permutation *P* < 0.001; spin spatial *ρ* = 0.974, spin permutation *P* < 0.001. For **f**, DMG network whole brain spatial *ρ* = 0.911, label permutation *P* < 0.001, spin spatial *ρ* = 0.885, spin permutation *P* < 0.001. Statistical maps were generated using threshold-free cluster enhancement and corrected for multiple comparisons using familywise error (FWE) rate correction for multiple testing. Two-tailed *P*_FWE_ < 0.01 was considered significant for connectivity data. Brain slices are shown in radiological orientation. The diagram in** a** was created using BioRender; Sidpra, J. https://BioRender.com/vi1xgg0 (2026).

**Extended Data Fig. 1:**
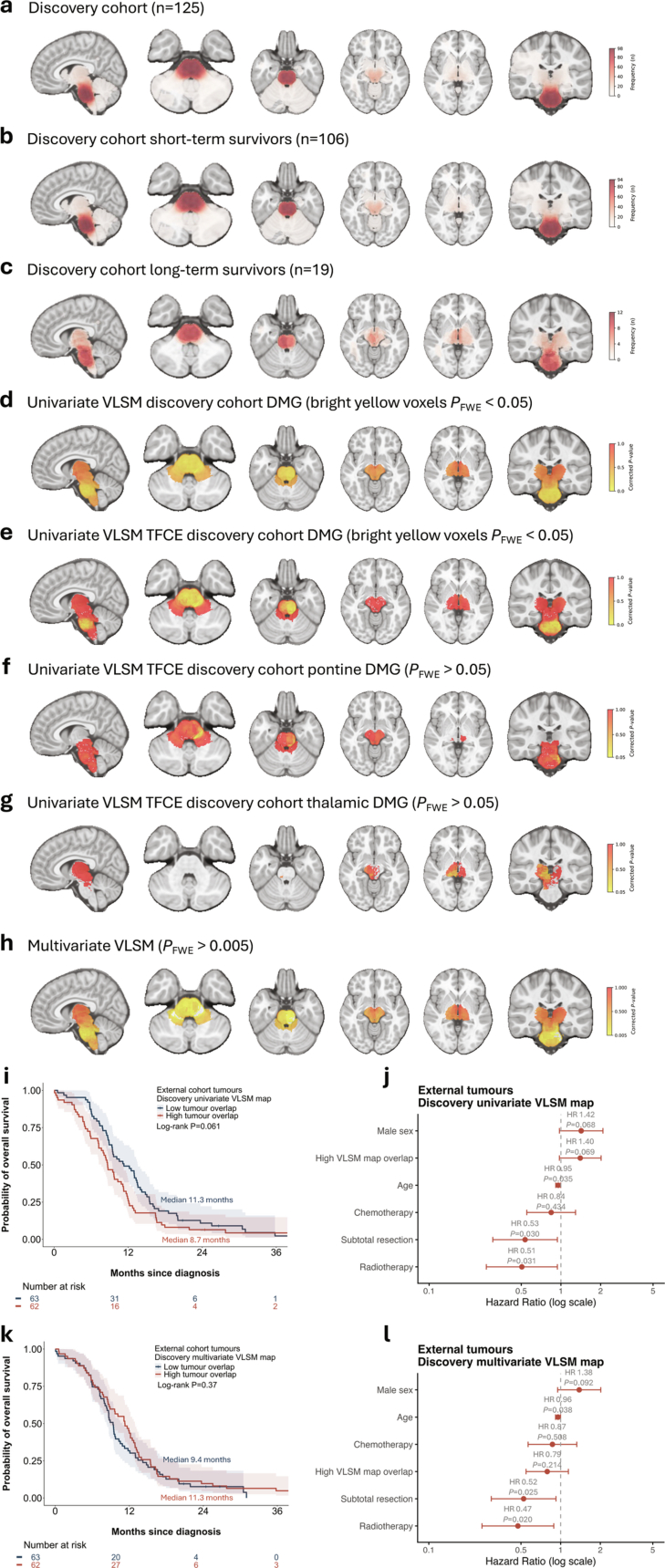
DMG tumour location mapping by voxel-based lesion symptom mapping (VLSM). Summed heatmaps of tumour anatomic locations as delineated on patient baseline MRI across the discovery cohort (**A;** n = 125) and for short- and long-term survivors within the discovery cohort (**B-C**, respectively). There was no difference in tumour volume between short-term and long-term survivors of DMG/DIPG (two-tailed Mann Whitney U-test *P* = 0.131). Short-term survivor median tumour volume 25815mm^3^ (IQR 20735-33705 mm^3^). Long-term survivor median tumour volume 23820mm^3^ (IQR 15745-28980 mm^3^). Results of mass-univariate lesion location mapping, both without and with threshold-free cluster enhancement (TFCE), restricted to at least 5% of overlapping voxels between tumours, across DMG/DIPG in the discovery cohort, respectively (**D-E**), expectedly revealed that tumour location within any pontine voxel was associated with shorter overall survival (*P*_FWE_ < 0.05). When TFCE analyses were repeated restricted to pontine DMG/DIPG (**F**) and thalamic DMG (**G**), respectively, no voxels significantly associated with shorter overall survival were identified (*P*_FWE_ > 0.05). Tumour location mapping was repeated across the discovery network with multivariate methods, the results of which did not identify any voxels across pontine DMG/DIPG or thalamic DMG associated with shorter overall survival (**H**; *P*_FWE_ > 0.005). Univariate and multivariate VLSM results were considered significant at two-tailed P_FWE_ < 0.05 and <0.005, respectively. The overlap of each tumour in an external, independent validation cohort (n = 125 children with DMG) was then computed with each unthresholded discovery VLSM map (univariate and multivariate) to generate a VLSM map overlap score per tumour. (**I,K**) Kaplan-Meier survival curves of all children in the external validation cohort demonstrate no survival difference between tumours with a high vs. low overlap with the unthresholded discovery cohort univariate or multivariate VLSM map. (**J-L**) Results of multivariable cox proportional hazards models confirm no prognostic impact of univariate and multivariate VLSM map overlap (n = 125). In forest plots, each dot represents an estimated HR and whiskers represent the corresponding 95% CI; dot size is for visualization only and does not denote statistical weight. Throughout, brain slices are depicted in radiological orientation.

**Extended Data Fig. 2:**
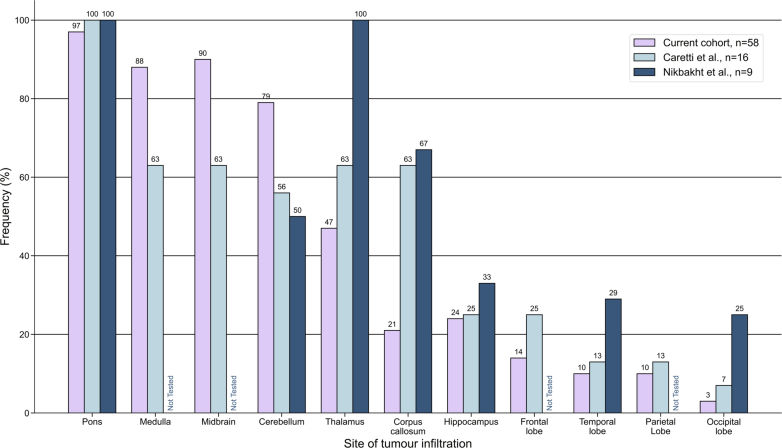
Brain-wide pattern of tumour infiltration in children with pontine DMG. Comparison of brain MRI data from this cohort with published post-mortem series of children with pontine DMG identifies a conserved pattern of tumour anatomic progression across study cohorts (main text refs. ^15,16^).

We first studied a discovery cohort of 125 children aged less than 18 years with primary pontine DMG/DIPG (*n* = 106), or thalamic DMG (*n* = 19), treated at Great Ormond Street Hospital for Children (GOSH; Fig. 1a and Supplementary Table 1). As pontine biopsies were historically not performed^19^, children meeting clinical diagnostic criteria for DIPG^20–22^ and with a disease course consistent with short-term (<18-month)^23^ overall survival were included. In line with the World Health Organisation 2021 definition of the disease^1^, biopsied tumours are classified as DMG, H3K27-altered, while non-biopsied pontine tumours are classified as DIPG. Throughout, reproducibility of findings is demonstrated for the combined (DMG/DIPG) patient cohort and across restricted cohorts of children with biopsied pontine or thalamic DMG, H3K27-altered. Patient tumours were segmented on preoperative brain magnetic resonance imaging (MRI) and mapped to a standard paediatric template (Extended Data Fig. 1a–c). Tumour locations were compared between patients with short- and long-term overall survival using voxel-based lesion-symptom mapping (VLSM). Univariate VLSM across discovery cohort pontine and thalamic tumours identified a significant association between patient short-term survival and tumour pontine location (Extended Data Fig. 1d,e), reflecting the known shorter overall survival of children with pontine versus thalamic DMG^2^. Aiming to identify specific voxels within the pons or thalamus associated with patient short- versus long-term survival, we performed separate univariate VLSM for pontine and thalamic tumour locations and multivariate VLSM across all tumour locations. Across both methods, no voxels associated with short-term survival were identified (Fig. 1b and Extended Data Fig. 1f–h). To test for any prognostic impact of subthreshold VLSM results (defined as weak, non-significant voxelwise associations with short-term survival), we collected data from an independent cohort of children with primary pontine DMG/DIPG (*n* = 80) or thalamic DMG (*n* = 45) treated at one of three international centres or included in the HERBY trial (NCT01390948^24^; Supplementary Table 1), with identical inclusion criteria. The weighted overlap of each tumour in this external validation cohort with univariate and multivariate VLSM maps, respectively, was computed and averaged to generate an overlap score^25^. No survival difference was identified between patient tumours stratified into high- and low-overlap risk groups using a median cut-off across univariate and multivariate VLSM (Extended Data Fig. 1i–l). Given this lack of association between tumour location and patient survival, the influence of neural activity on DMG growth in preclinical models^3–8^ and the fact that human DMG/DIPG exhibits a common pattern of brain infiltration (Extended Data Fig. 2), we hypothesized that tumour connectivity with an underlying brain network would better explain the observed variance in patient survival.

Lesion network mapping is a computational neuroimaging method that harnesses population-level (*n* = 1,000) connectomic data to model common brain network connections across different lesion locations^26–28^. It has successfully identified brain networks and therapeutic targets across neurological and psychiatric disorders, including epilepsy^29,30^, addiction^31^ and depression^32^. Here, we develop tumour network mapping, using a lesion network mapping framework, to identify common brain network connections associated with the short-term survival of patients with DMG, irrespective of tumour primary pontine or thalamic location. Tumour volume was included as a covariate in tumour network mapping analyses to account for potential confounding by lesion size^26,29,31^. In this cohort, tumour volume was not associated with overall survival (univariate Cox hazard ratio (HR) per 1,000 mm^3^, increase of 1.00; 95% confidence interval (CI) = 0.99–1.01; *P* = 0.569), consistent with previous clinical reports^33,34^. Given our aim to identify polysynaptic and direct structural connections between brain regions, whole-brain-to-tumour connectivity analyses were performed across paediatric resting-state functional connectivity (functional MRI, fMRI)^35^ and structural connectivity (diffusion-weighted MRI, dMRI)^36,37^ data. These analyses identified a distributed brain network associated with shorter patient overall survival (Fig. 1c). More specifically, tumours exhibiting greater positive functional connectivity with the pons, thalamus, motor cortex, insular and orbitofrontal cortices, limbic system (amygdala, hippocampus, basal ganglia and cingulate cortex), superior temporal gyrus and cerebellum were associated with shorter patient overall survival (Fig. 1d and Supplementary Table 2). Hereafter, we refer to this discovery cohort-defined map of functional brain network connections associated with shorter patient overall survival as the DMG network (Fig. 1d). Concordantly, tumours exhibiting greater structural connectivity with these brain areas through the cerebellar peduncles, corticospinal tracts, corpus callosum, corticobulbar tracts and spinal–limbic pathways were associated with shorter overall survival (Extended Data Fig. 4a).

**Extended Data Fig. 3:**
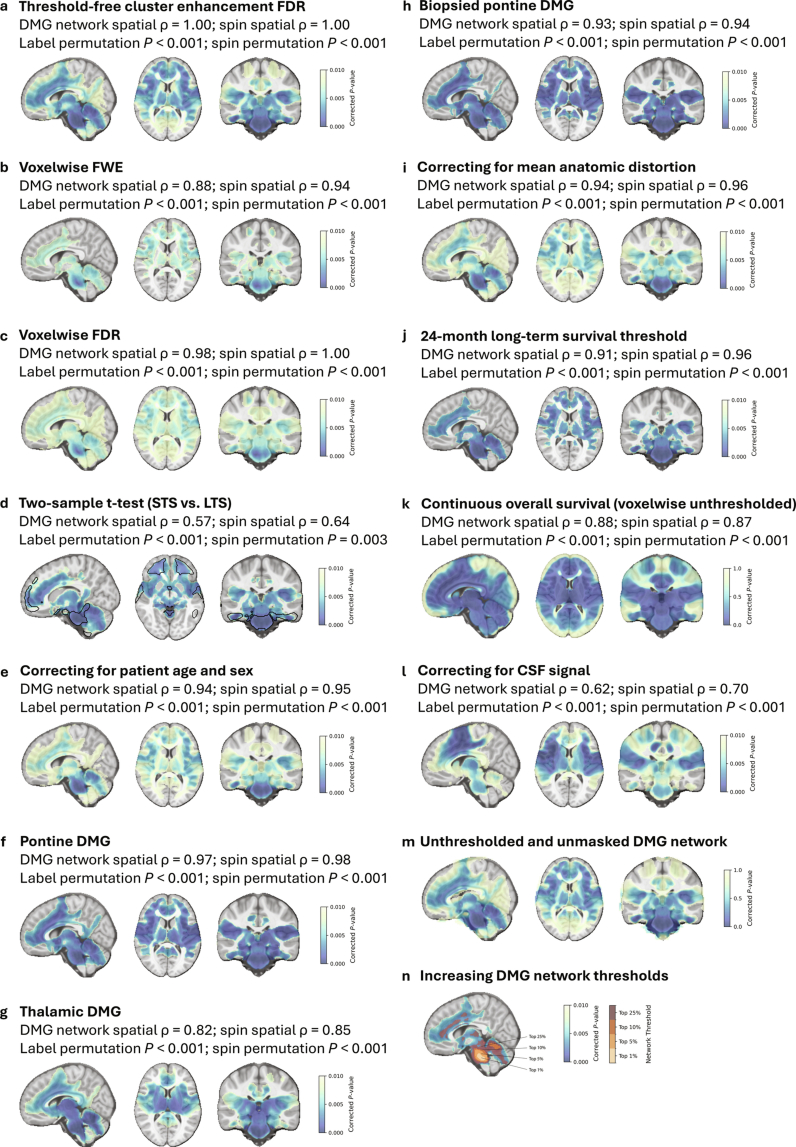
DMG network results are robust to statistical and methodological choices across functional connectivity (fMRI) data. The DMG network identified in our primary analysis (Main Text Fig. 1) is reproducible independent of statistical test employed for tumour network mapping: TFCE FDR (**A**; whole brain spatial ρ = 1.000, label permutation *P* < 0.001; spin spatial ρ = 1.000, spin permutation *P* < 0.001); voxelwise FWE (**B**; whole brain spatial ρ = 0.877, label permutation *P* < 0.001; spin spatial ρ = 0.937, spin permutation *P* < 0.001); and voxelwise FDR (**C**; whole brain spatial ρ = 0.978, label permutation *P* < 0.001; spin spatial ρ = 0.997, spin permutation *P* < 0.001). Importantly, the DMG network remains robust to specificity testing for significant connectivity differences between short- and long-term survivors (**D**; spatial ρ = 0.566, label permutation *P* < 0.001; spin spatial ρ = 0.640, spin permutation *P* = 0.003) and when controlling for patient age and sex (**E**; spatial ρ = 0.944, label permutation *P* < 0.001; spin spatial ρ = 0.953, spin permutation *P* < 0.001); restricting to primary pontine DMG only (**F**; spatial ρ = 0.966, label permutation *P* < 0.001; spin spatial ρ = 0.976, spin permutation *P* < 0.001); restricting to primary thalamic DMG only (**G**; spatial ρ = 0.823, label permutation *P* < 0.001; spin spatial ρ = 0.854, spin permutation *P* < 0.001); and restricting to biopsy-confirmed pontine DMG only (**H**; spatial ρ = 0.927, label permutation *P* < 0.001; spin spatial ρ = 0.943, spin permutation *P* < 0.001). In addition, DMG network topography was robust to correction for mean peritumoral anatomic distortion (**I**; spatial ρ = 0.943, label permutation *P* < 0.001; spin spatial ρ = 0.964, spin permutation *P* < 0.001) and upon increasing the threshold for long-term survival from ≥18 to ≥24 months (**J**; spatial ρ = 0.913, label permutation *P* < 0.001; spin spatial ρ = 0.963, spin permutation *P* < 0.001). Use of a continuous contrast for patient overall survival captured network nodes in the thalami and midbrain at more liberal statistical thresholds but failed to define the entire network, likely reflecting decreased statistical power (**K**; spatial ρ = 0.741, label permutation *P* < 0.001; spin spatial ρ = 0.763, spin permutation *P* < 0.001). The pontine origin of DMG network signal was confirmed via inclusion of fourth ventricular CSF signal as a co-regressor (**L**; spatial ρ = 0.623, label permutation *P* < 0.001; spin spatial ρ = 0.698, spin permutation *P* < 0.001) and through the use of stepped statistical thresholds (*T*-values; red-to-orange) in comparison to the unthresholded DMG network (**M-N**). In all instances, two-tailed *P*_FWE_ < 0.01 or *P*_FDR_ < 0.01, respectively, were considered significant for connectivity data. Two-tailed *P* < 0.05 was considered significant for tests of spatial similarity.

**Extended Data Fig. 4:**
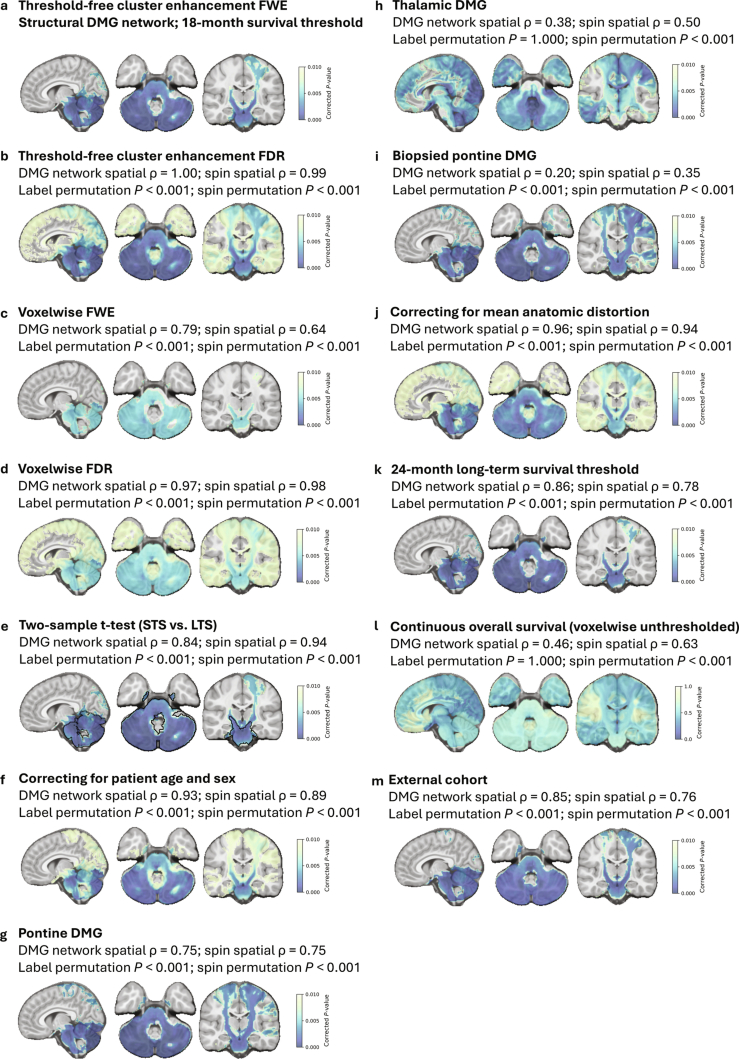
DMG network structural connectivity is robust to statistical and methodological choices across dMRI data. Tumour network mapping was repeated using dMRI data across 497 healthy children from the Human Connectome Project Lifespan Development (HCP-D) dataset, identifying a structural network of monosynaptic tumour connections associated with shorter patient overall survival (**A;** TFCE FWE). Strong connectivity was observed with brain regions identified in the DMG network, particularly via the cerebellar peduncles, corticospinal tracts, corpus callosum, corticobulbar tracts, and spinal-limbic pathways. This result was reproducible independent of statistical test: TFCE FDR (**B**; spatial ρ = 0.999, label permutation *P* < 0.001; spin spatial ρ = 0.993, spin permutation *P* < 0.001); voxelwise FWE (**C**; spatial ρ = 0.787, label permutation *P* < 0.001; spin spatial ρ = 0.638, spin permutation *P* < 0.001); and voxelwise FDR (**D**; spatial ρ = 0.974, label permutation *P* < 0.001; spin spatial ρ = 0.983, spin permutation *P* < 0.001). Importantly, the structural network was also robust to specificity testing for significant connectivity differences between short- and long-term survivors (**E**; spatial ρ = 0.842, label permutation *P* < 0.001; spin spatial ρ = 0.940, spin permutation *P* < 0.001) and when controlling for patient age and sex (**F**; spatial ρ = 0.928, label permutation *P* < 0.001; spin spatial ρ = 0.892, spin permutation *P* < 0.001); restricting to primary pontine DMG only (**G**; spatial ρ = 0.754, label permutation *P* < 0.001; spin spatial ρ = 0.747, spin permutation *P* < 0.001); restricting to primary thalamic DMG only (**H**; spatial ρ = 0.384, label permutation *P* = 1.000; spin spatial ρ = 0.500, spin permutation *P* < 0.001); and restricting to biopsy-confirmed pontine DMG only (**I**; spatial ρ = 0.200, label permutation *P* < 0.001; spin spatial ρ = 0.348, spin permutation *P* < 0.001). Structural network topography was robust to correction for mean peritumoral anatomic distortion, suggesting potential tract distortion by tumour mass effect has a minimal effect on tumour connectivity (**J**; spatial ρ = 0.964, label permutation *P* < 0.001; spin spatial ρ = 0.944, spin permutation *P* < 0.001). It was also robust to an increase in the threshold for long-term survival from ≥18 to ≥24 months (**K**; spatial ρ = 0.857, label permutation *P* < 0.001; spin spatial ρ = 0.780, spin permutation *P* < 0.001); and when using a continuous contrast for patient overall survival, although at more liberal statistical thresholds (**L**; spatial ρ = 0.292, label permutation *P* = 1.000; spin spatial ρ = 0.630, spin permutation *P* < 0.001). Finally, the structural DMG network was reproducible using patient data from an independent, external validation cohort (**M**; n = 125 children with primary pontine and thalamic DMG; spatial ρ = 0.854, label permutation *P* < 0.001; spin spatial ρ = 0.760, spin permutation *P* < 0.001). In all instances, two-tailed *P*_FWE_ < 0.01 or *P*_FDR_ < 0.01, respectively, were considered significant for connectivity data. Two-tailed *P* < 0.05 was considered significant for tests of spatial similarity.

**Extended Data Fig. 5:**
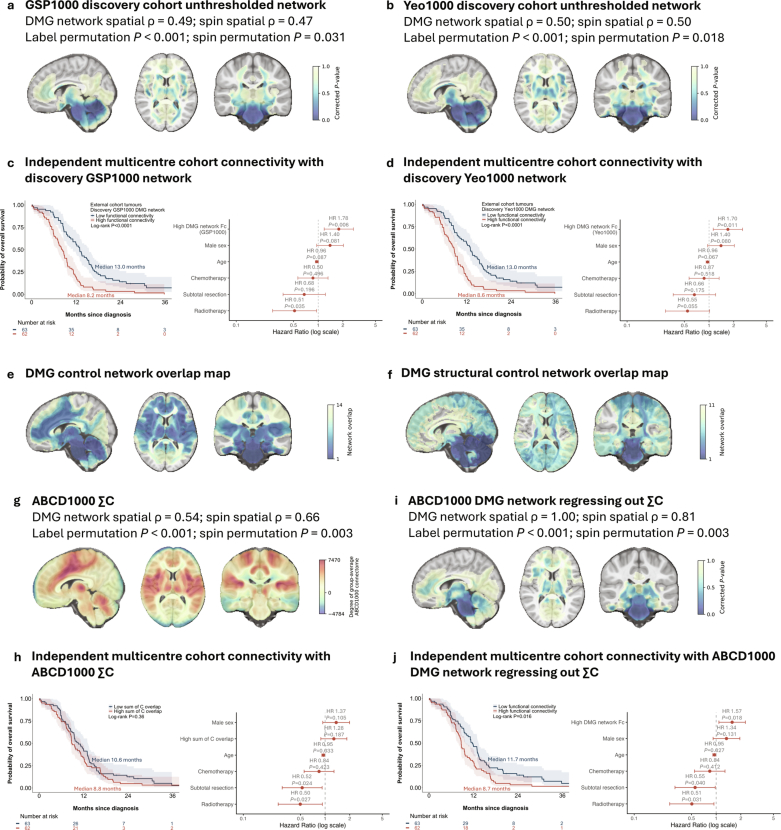
DMG network results and prognostic impact are robust to connectome controls. The functional DMG network identified in our primary analysis (Main Text Fig. 1.) was constructed from a resting-state fMRI dataset of 1000 healthy children aged 9 ± 0.2 years (ABCD1000). DMG network peaks and spatial topography remain unchanged when tumour network mapping was repeated using the discovery cohort across two independent connectomes constructed from the resting-state functional connectivity data of 1000 healthy adults. (**A**) GSP1000: spatial ρ = 0.49, label permutation *P* < 0.001; spin spatial ρ = 0.47, spin permutation *P* = 0.031. (**B**) Yeo1000: whole brain spatial ρ = 0.50, label permutation *P* < 0.001; spin spatial ρ = 0.50, spin permutation *P* = 0.018. Two-tailed *P* < 0.05 was considered significant for tests of spatial similarity. Connectivity maps are shown unthresholded to facilitate comparison. Network-to-tumour connectivity was then computed for children in the external validation cohort across discovery cohort generated GSP1000 and Yeo1000 networks using identical methods to those for the primary, ABCD1000-derived DMG network. Kaplan-Meier survival curves demonstrate a clear and clinically meaningful survival difference when patients are stratified by network-to tumour connectivity whilst multivariable cox proportional hazards models confirm network-to-tumour connectivity as an independent predictor of patient overall survival (**C-D**). (**C**) GSP1000: Kaplan-Meier median overall survival 8.2 vs. 13.0 months; 95% CI 7.1-9.5 vs. 11.1-15.5 months; *P* = 4.02 × 10^−6^. Multivariable cox HR 1.78; 95% CI 1.18-2.69; *P* = 0.006. (**D**) Yeo1000: Kaplan-Meier median overall survival 8.6 vs. 13.0 months; 95% CI 7.2-9.6 vs. 10.6-15.5 months; *P* = 9.43 × 10^−6^. Multivariable cox HR 1.70; 95% CI 1.12-2.56; *P* = 0.012. (**E-F**) Frequency maps of brain regions significantly associated with shorter patient overall survival across control analyses (Extended Data Figs. 3, 4) for functional connectivity (**E**) and structural connectivity (**F**) data. (**G-J**) The summation vector of the group-average ABCD1000 functional connectome (∑C) was also computed and, though spatially similar to the DMG network (**G**; spatial ρ = 0.540, label permutation *P* < 0.001; spin spatial ρ = 0.657, spin permutation *P* = 0.003), was non-prognostic (**H**). Further, the DMG network retained its spatial topography (**I**; spatial ρ = 1.000, label permutation *P* < 0.001; spin spatial ρ = 0.814, spin permutation *P* = 0.003) and prognostic effect (**J**; Kaplan-Meier median overall survival 8.7 vs. 11.7 months; 95% CI 7.96-9.76 vs. 9.70-13.5 months; *P* = 0.016. Multivariable cox HR 1.57; 95% CI 1.08-2.27; *P* = 0.018) when ∑C was regressed out of patient tumour network maps. In forest plots, each dot represents an estimated HR and whiskers represent the corresponding 95% CI; dot size is for visualization only and does not denote statistical weight.

The DMG network and its structural connectivity were robust to statistical methods (Extended Data Figs. 3a–c and 4a–d); specificity testing for connectivity differences between short- and long-term survivors (Extended Data Figs. 3d and 4e); controlling for patient age and sex (Extended Data Figs. 3e and 4f); controlling for tumour primary pontine or thalamic location (Extended Data Figs. 3f,g and 4g,h); restricting to biopsied pontine DMG, H3K27-altered, only (Extended Data Figs. 3h and 4i); and controlling for mean peritumoural anatomical distortion (Extended Data Figs. 3i and 4j). The DMG network was also reproducible when using both clinically reported cut-offs for the long-term survival of patients with DMG (≥18 months^23^ and ≥24 months^38^, respectively; Extended Data Figs. 3a,j and 4a,k) and with a continuous contrast for patient overall survival, although this captured less of the network at stricter statistical thresholds (Extended Data Figs. 3k and 4l). The parenchymal origin of pontine fMRI signal was confirmed through the inclusion of fourth ventricular cerebrospinal fluid signal as a co-regressor in partial correlation analysis when computing the functional connectivity of each patient tumour (Extended Data Fig. 3l) and through visualization of increasing statistical thresholds (Extended Data Fig. 3m,n). DMG network spatial topography was replicated across two unique, population-level adult functional connectomes with different preprocessing methods^39,40^ (Extended Data Fig. 5a–d). In all instances, the similarity of control networks to the DMG network was assessed against a null distribution of 10,000 networks using two methods: (1) label permutation testing, in which each patient’s functional connectivity map was randomly paired with another patient’s overall survival value to generate each null network; and (2) spin permutation testing, in which the DMG network was parcellated and randomly rotated to generate each null network. Finally, visualization of thresholded control network overlap demonstrated convergent topography (Extended Data Fig. 5e,f).

To test for DMG network reproducibility, we performed tumour network mapping using identical methods across two independent cohorts: the previously described multicentre external validation cohort (*n* = 125) and a second cohort of 38 children with biopsy-confirmed pontine DMG, H3K27-altered, included as a secondary analysis of Pediatric Neuro-Oncology Consortium (PNOC) trial data (Supplementary Table 1). Across both independent cohorts, tumours demonstrated the same pattern of brain network connections associated with short-term overall survival, converging to the primary, discovery cohort-defined DMG network (Fig. 1e,f and Extended Data Fig. 4m). Collectively, these data define a conserved, prognostic human brain network across pontine and thalamic DMG, connectivity with which is associated with shorter patient overall survival.

## DMG network connectivity predicts patient survival

**Fig. 2:**
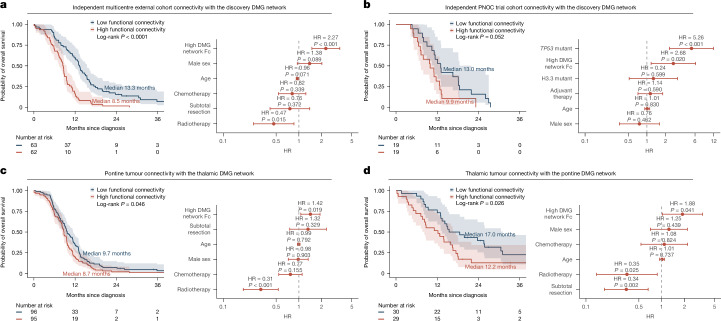
DMG-network-to-tumour connectivity predicts overall survival in children with DMG. Kaplan–Meier survival curves and associated multivariable Cox proportional hazard models demonstrate shorter overall survival in children whose tumours exhibit higher DMG network connectivity and confirm network-to-tumour connectivity as an independent predictor of patient overall survival. **a**, The network-to-tumour connectivity of 125 children with primary pontine DMG/DIPG and thalamic DMG in an independent external validation cohort was computed on the discovery DMG network. Kaplan–Meier median overall survival, 8.5 versus 13.3 months; 95% CI = 7.4–9.5 versus 11.7–15.5 months; *P* = 1.76 × 10^−7^. Multivariable Cox HR = 2.27, 95% CI = 1.48–3.47, *P* = 0.000161. **b**, The network-to-tumour connectivity of 38 children with biopsy-confirmed pontine DMG, H3K27-altered was computed on the discovery DMG network. Kaplan–Meier median overall survival, 9.9 versus 13.0 months; 95% CI = 7.3–13.1 versus 11.1–21.9 months; *P* = 0.052. Multivariable Cox HR = 2.68, 95% CI = 1.17–6.13, *P* = 0.020. **c**, The network-to-tumour connectivity of 191 children with pontine DMG/DIPG was computed on a thalamic DMG network. Kaplan–Meier median overall survival, 8.7 versus 9.7 months; 95% CI = 8.2–9.6 versus 8.9–11.4 months; *P* = 0.046. Multivariable Cox HR = 1.42, 95% CI = 1.06–1.90, *P* = 0.019. **d**, The network-to-tumour connectivity of 59 children with thalamic DMG was computed on a pontine DMG/DIPG network. Kaplan–Meier median overall survival, 12.2 versus 17.0 months; 95% CI = 8.3–16.6 versus 13.4–31.0 months; *P* = 0.026. Multivariable Cox HR = 1.88, 95% CI = 1.03–3.46, *P* = 0.041. In the forest plots, each dot represents an estimated HR and whiskers represent the corresponding 95% CI; dot size is for visualization only and does not denote statistical weight. Fc, functional connectivity.

**Extended Data Fig. 6:**
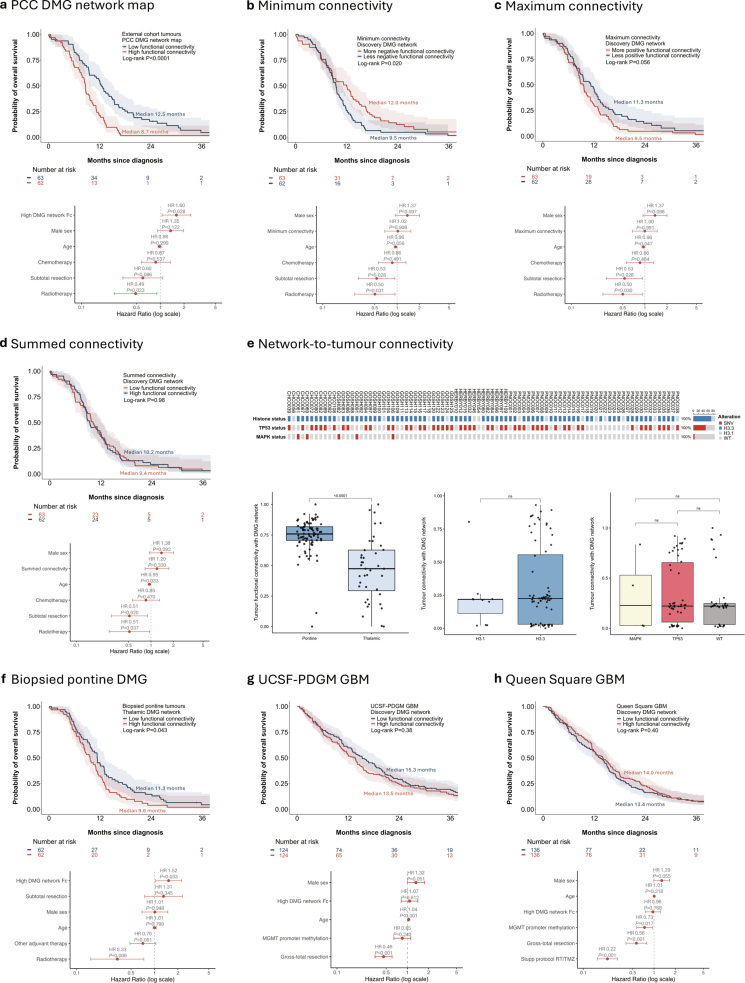
The prognostic significance of DMG network-to-tumour connectivity is robust to methodological choices, independent of prognostic co-mutations, and specific to DMG. (**A-D**) Kaplan-Meier survival curves and associated multivariable cox proportional hazard models constructed using external validation cohort data with network-to-tumour connectivity calculated on the discovery cohort derived DMG network. (**A**) The prognostic impact of network-to-tumour connectivity is robust when computed using an alternate, spatial-correlation method in which each patient tumour-specific functional connectivity map was spatially compared to a pre-computed connectome (PCC) map generated through weighted seeding of DMG network peaks. Kaplan-Meier median overall survival 8.7 vs. 12.5 months; 95% CI 7.8-9.8 vs. 9.6-15.4 months; *P* = 2.69 × 10^−5^. Multivariable cox HR 1.60; 95% CI 1.05-2.43; *P* = 0.028. (**B**) Minimum network-to-tumour connectivity is weakly prognostic but does not independently predict patient overall survival. Kaplan-Meier median overall survival 9.5 vs. 12.0 months; 95% CI 8.4-10.9 vs. 8.6-14.2 months; *P* = 0.020. Multivariable cox HR 1.02; 95% CI 0.68-1.53; *P* = 0.908. (**C**) Maximum network-to-tumour connectivity is weakly prognostic but does not independently predict patient overall survival. Kaplan-Meier median overall survival 9.5 vs. 11.3 months; 95% CI 8.4-11.1 vs. 8.9-13.0 months; P = 0.056. Multivariable cox HR 1.00; 95% CI 0.68-1.47; P = 0.991. (**D**) Summed network-to-tumour connectivity is non-prognostic. Kaplan-Meier median overall survival 9.4 vs. 10.2 months; 95% CI 8.5-12.2 vs. 8.6-12.2 months; *P* = 0.980. Multivariable cox HR 1.20; 95% CI 0.83-1.74; *P* = 0.330. (**E**) Comparison of multicentre external validation cohort network-to-tumour connectivity values revealed that pontine DMG/DIPG exhibited greater DMG network functional connectivity than thalamic DMG (median 0.758 vs. 0.473; two-tailed Mann-Whitney U-test *P* = 3.12e^−9^). Importantly, comparison of 87 tumours across the whole study cohort with next-generation DNA panel sequencing identified no difference in network-to-tumour connectivity when stratifying by oncohistone variant (H3.1 vs. H3.3; median Fc 0.218 vs. 0.225; two-tailed Mann-Whitney U-test *P* = 0.401) and somatic mutation (MAPK pathway (*BRAF*/*FGFR1*) vs. *TP53* vs. WT; median Fc 0.227 vs. 0.222 vs. 0.221; MAPK vs. WT two-tailed Mann-Whitney U-test *P* = 0.934; *TP53* vs. WT two-tailed Mann-Whitney U-test *P* = 0.856). For these latter analyses, network-to-tumour connectivity values were calculated for pontine DMG/DIPG and thalamic DMG from the opposing DMG network (as in Main Text Fig. 2) and normalized within each group, enabling pan-cohort comparison. Data are median (centre line), IQR (box limits), whiskers extending to the most extreme values within 1.5x IQR, and individual observations (dots). (**F**) Network-to-tumour connectivity remained an independent prognostic factor when restricting to children with biopsy-confirmed pontine DMG, H3K27-altered, only across the study cohort (n = 124). Kaplan-Meier median overall survival 8.6 vs. 11.3 months; 95% CI 8.2-11.4 vs. 9.7-13.1 months; *P* = 0.043. Multivariable cox HR 1.52; 95% CI 1.03-2.23; *P* = 0.033. (**G-H**) Kaplan-Meier survival curves and multivariable cox proportional hazards models demonstrate that GBM connectivity with the DMG network is not prognostic across two validation datasets and that, therefore, the DMG network is specific to DMG. (**G**) UCSF-PDGM, n = 248. Kaplan-Meier median overall survival 13.5 vs. 15.3 months; 95% CI 10.6-15.5 vs. 12.8-18.6 months; *P* = 0.38. Multivariable cox HR 1.07; 95% CI 0.82-1.41; *P* = 0.612. (**H**) Queen Square, n = 272. Kaplan-Meier median overall survival 13.4 vs. 14.0 months; 95% CI 11.8-15.2 vs. 11.6-16.2 months; *P* = 0.40. Multivariable cox HR 0.96; 95% CI 0.75-1.23; *P* = 0.768. In all Kaplan-Meier curves, the statistical difference between groups was evaluated using the log-rank test with error bars representative of 95% CI. In all forest plots, mean values are represented by solid circles and whiskers represent 95% CI. In forest plots, each dot represents an estimated HR and whiskers represent the corresponding 95% CI; dot size is for visualization only and does not denote statistical weight.

**Extended Data Fig. 7:**
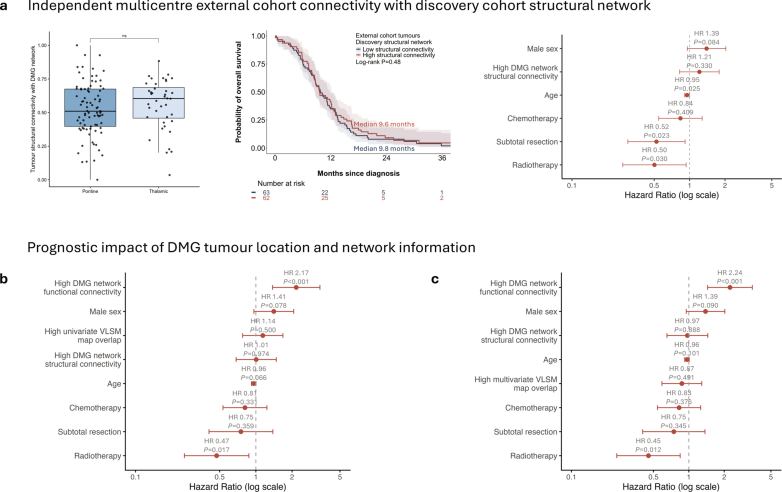
DMG network functional connectivity, rather than tumour location and DMG network structural connectivity, is associated with patient overall survival. (**A**) Comparison of multicentre external validation cohort network-to-tumour connectivity values revealed that there was no significant difference in structural connectivity to the DMG network between pontine DMG/DIPG and thalamic DMG (median 0.510 vs. 0.604; two-tailed Mann-Whitney U-test *P* = 0.234). DMG network-to-tumour structural connectivity was not independently associated with patient overall survival across the external multicentre validation cohort (centre and right). Kaplan-Meier median overall survival 9.8 vs. 9.6 months; 95% CI 8.6-11.9 vs. 8.6-12.2 months; *P* = 0.48. Multivariable cox HR 1.21; 95% CI 0.82-1.78; *P* = 0.330. Data are median (centre line), IQR (box limits), whiskers extending to the most extreme values within 1.5x IQR, and individual observations (dots). (**B-C**) Direct comparison of the prognostic impact of tumour location and tumour network information across the external multicentre validation cohort (**B**, univariate VLSM; **C**, multivariate VLSM). In both instances, network-to-tumour functional connectivity was an independent predictor of patient overall survival (**B**: multivariable cox HR 2.17 95% CI 1.38-3.41; *P* = 0.000839; **C**: multivariable cox HR 2.24 95% CI 1.45-3.47; *P* = 0.000307). In forest plots, each dot represents an estimated HR and whiskers represent the corresponding 95% CI; dot size is for visualization only and does not denote statistical weight.

To validate the prognostic importance of the DMG network, the mean strength of network-to-tumour connectivity with the primary, discovery cohort-defined DMG network was computed for each tumour across both external validation cohorts: data that were not used to define the DMG network. Network-to-tumour connectivity was computed as the weighted overlap of patient-level tumour functional connections with DMG network prognostic connections and patients stratified into high- and low-connectivity risk groups using a median cut-off^25^. Kaplan–Meier survival analyses demonstrated a significant survival difference across both independent cohorts such that children with higher network-to-tumour connectivity had shorter overall survival (Fig. 2a,b). Univariable and multivariable Cox proportional hazards models incorporating clinical covariates (age, sex, extent of resection, and completion of adjuvant radiotherapy and chemotherapy) confirmed network-to-tumour connectivity as an independent predictor of overall survival in children with DMG/DIPG (Fig. 2a,b). These findings were replicated across connectomes (Extended Data Fig. 5c,d) and an orthogonal method for computing network-to-tumour connectivity^29^ (Extended Data Fig. 6a). Notably, minimum, maximum and summed network-to-tumour connectivity were not prognostic, reinforcing the importance of distributed, network-level effects rather than isolated strong or weak connections (Extended Data Fig. 6b–d). Although minimum connectivity stratified patients into survival groups, this reflected a tumour-wide shift towards higher or lower mean network-to-tumour functional connectivity (Spearman’s *ρ* = 0.23; *P* = 0.011), and failed to survive multivariable modelling. Similarly, DMG network structural connectivity, representative of monosynaptic connections, was non-prognostic (Extended Data Fig. 7a). To confirm this and directly compare the prognostic impact of tumour network and tumour location information, we constructed Cox proportional hazards models incorporating tumour–VLSM map overlap and DMG network-to-tumour functional and structural connectivity. High functional connectivity was robustly associated with survival, whereas neither structural connectivity nor tumour–VLSM overlap showed prognostic significance (Extended Data Fig. 7b,c). Taken together, mean DMG network-to-tumour functional connectivity was robustly and reproducibly associated with patient overall survival.

DMGs are characterized by canonical (H3.1 or H3.2) or non-canonical (H3.3) alterations in histone 3^41^ or *EZHIP* overexpression^42^, all of which result in polycomb repressor complex 2 (PRC2) inhibition^43^, global loss of H3K27 trimethylation and widespread epigenetic dysregulation^44^. Two molecular prognostic groups have been reported, defined by *TP53* or *BRAF/FGFR1* oncogenic variants^23^. We analysed paediatric solid tumour next-generation DNA panel sequencing data for a subcohort of 87 children with pontine or thalamic DMG, comparing the mean DMG network-to-tumour connectivity strength for each alteration. Negative results suggest that network-to-tumour connectivity is independent of tumour histone status and co-mutations (Extended Data Fig. 6e).

To formally test whether pontine and thalamic DMGs exhibit prognostic convergence to the same brain network, we performed tumour network mapping separately for all pontine DMG/DIPG and all thalamic DMG, defining distinct prognostic networks (Extended Data Fig. 3f,g). The mean connectivity strength of pontine DMG/DIPG and thalamic DMG was computed with the alternate location network to test for a survival association. Kaplan–Meier survival curves and univariable and multivariable Cox proportional hazards models incorporating clinical covariates consistently demonstrated an inverse relationship between high network-to-tumour connectivity and patient survival, confirming convergent prognostic networks independent of tumour primary location (Fig. 2c,d). Similar and overlapping HRs suggest conserved network effects across tumour locations. The prognostic impact of DMG network-to-tumour connectivity was also reproducible when restricting the external validation cohort to children with biopsy-confirmed pontine DMG, H3K27-altered (Extended Data Fig. 6f), and when analysing a second, independent cohort of children with biopsy-confirmed pontine DMG, H3K27-altered (PNOC trial cohort; Fig. 2b).

We next sought to confirm the predictive power and incremental prognostic value of network-to-tumour connectivity beyond standard clinical variables. To this end, we fitted clinical-only and combined clinical–connectivity multivariable Cox proportional hazards models on all patients in the discovery cohort and applied the fully parameterized models with frozen coefficients to the independent external validation cohort, comparing model discriminatory power. In the external validation cohort, the inclusion of network-to-tumour connectivity improved prognostic discrimination relative to clinical variables only (concordance index, 0.621 versus 0.577; difference in concordance index (Δ*C*) = 0.044, 95% CI = 0.016–0.074, *P* = 0.002), confirming that network-to-tumour connectivity provides independent prognostic information beyond established clinical factors.

We hypothesized that the DMG network would specifically predict the survival of children with DMG rather than patients with other high-grade glioma subtypes: mirroring preclinical evidence for some distinct, tumour subtype-specific mechanisms of neuron–glioma interaction^6,45^. To test this, we analysed two independent cohorts of adult patients with glioblastoma (GBM), *IDH*-wild type: a previously reported cohort of 248 patients (UCSF-PDGM)^46^ and a cohort of 272 patients treated at the National Hospital for Neurology and Neurosurgery, London (Supplementary Table 3). Across cohorts, GBM connectivity with the DMG network was not associated with GBM patient overall survival (Extended Data Fig. 6g,h). To further test DMG network specificity, we investigated whether DMG network prognostic effects reflect common paediatric connectome brain architecture, computing the row summation vector of the group-average Adolescent Brain Cognitive Development Study (ABCD1000) connectivity matrix (∑*C*)^47^. While ∑*C* was spatially similar to the DMG network (Extended Data Fig. 5g), tumour connectivity with ∑*C* was non-prognostic (Extended Data Fig. 5h). Moreover, after regressing out ∑*C* from tumour network maps, the residual DMG network remained topographically similar to the primary DMG network and predicted overall survival in independent datasets (Extended Data Fig. 5i,j). These results indicate that, although DMGs exhibit connectivity with constitutively active hub-like brain regions, the prognostic value of the DMG network is insufficiently explained by connectome structure and, instead, reflects a disease-specific prognostic circuit^48^. Taken together, these findings demonstrate that, despite disparate anatomic origins, DMG network-to-tumour connectivity is independently and specifically predictive of overall survival in children with DMG.

## Tumour progression maps to the DMG network

**Fig. 3:**
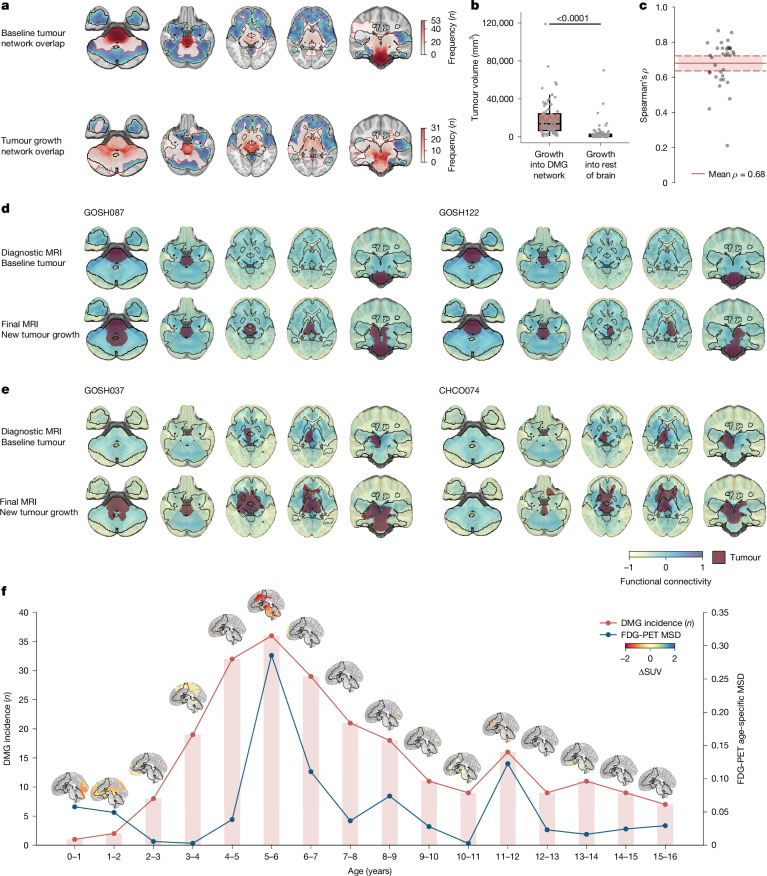
DMG network topography aligns with tumour growth and neurometabolic development. **a**, Tumour network overlap maps illustrate DMG/DIPG anatomic distribution at the time of diagnostic MRI and new tumour growth from diagnostic to final MRI within 2 months of death.* n* = 71 children with paired MRI. The voxelwise tumour frequency is plotted on a scale of red to white; DMG network is shown on a scale of blue to yellow. **b**, New tumour growth was greater within the DMG network than out of the DMG network (*n* = 71; median, 13,768 mm^3^ versus 1,270 mm^3^; 95% CI = 11,293–17,763 mm^3^ versus 773–1,792 mm^3^; two-tailed paired Wilcoxon signed-rank test, *P* = 6.85 × 10^−13^). The box plots show the median (centre line), interquartile range (IQR; box limits) and individual observations (dots), and the whiskers extend to the most extreme values within 1.5× IQR. **c**, Tumour growth into DMG network nodes correlated with node-to-tumour connectivity, correcting for tumour-wise distance to each node. *n* = 34 children with metastatic growth beyond primary tumour site. Mean Spearman’s *ρ* = 0.68, 95% CI = 0.63–0.72. All but two individual patient correlations were significant after Benjamini–Hochberg correction (two-tailed *P*_FDR_ < 0.05). The patient-level *ρ* is shown in grey; the group-level median is shown in red with bootstrapped 95% CIs. **d**,**e**, Tumour-network overlap maps of representative pontine (**d**) and thalamic (**e**) DMG/DIPG at the time of the diagnostic and final MRI illustrate growth aligned with DMG network connectivity. **f**, Bar plot of whole-cohort age incidence (red; *n* = 284 children, four children aged >17 years excluded) with line and scatter plots of age-consecutive mean-squared differences (MSDs) in brain-wide [^18^F]FDG-PET signal across childhood (blue; 0–16 years) demonstrate temporal alignment of peak tumour incidence with peak [18^F^]FDG-PET signal changes (*R* = 0.612, linear regression *P* = 0.012). Brain-wide spatial maps above each bar depict brain regions with peak neurometabolic flux at each age (standardized uptake value (SUV_Max_); red indicates positive change), which predominate within the DMG network at ages of peak DMG incidence (5–6 years: voxelwise spatial *ρ* = 0.199, label permutation *P* < 0.001, spin spatial *ρ* = 0.512, spin permutation *P* < 0.001; 10–11 years: voxelwise spatial *ρ* = 0.044, label permutation *P* < 0.001, spin spatial *ρ* = 0.179, spin permutation *P* < 0.001).

**Extended Data Fig. 8:**
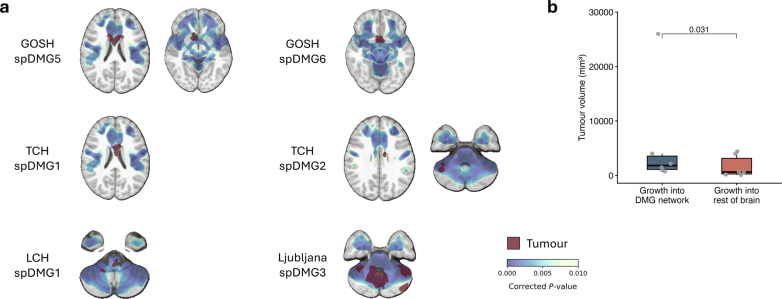
Intracranial metastases of primary spinal DMG exhibit anatomic tropism to the DMG network. (**A**) Six children with parenchymal (non-leptomeningeal) metastases of primary spinal DMG were identified from an independent international cohort of children with primary spinal DMG (n = 36). All intracranial lesions anatomically mapped to the DMG network. GOSH: Great Ormond Street Hospital for Children NHS Foundation Trust; LCH: Lurie Children’s Hospital; spDMG: spinal DMG; TCH: Texas Children’s Hospital. (**B**) Metastatic tumour volume (mm^3^) in the DMG network was significantly greater than in the rest of the brain (n = ; median 1813.0 vs. 618.5 mm^3^; IQR 712.0-2600.0 vs. 94.0-4415.0 mm^3^; two-tailed paired Wilcoxon signed-rank test *P* = 0.031). Data are median (centre line), IQR (box limits), whiskers extending to the most extreme values within 1.5x IQR, and individual observations (dots).

DMG diffusely and contiguously infiltrate brain parenchyma. Neural activity in distant brain regions projecting onto high-grade glioma cells promotes directional growth through paracrine and synaptic mechanisms in mouse models^12,49^. We investigated whether tumour invasion and metastasis exhibit directionality to defined DMG network peaks. First, we identified a subcohort of 71 children with paired diagnostic and final follow-up MRI acquired within 2 months of death, representing near-final disease progression (Supplementary Table 4). Tumour growth maps were generated through subtraction of final progression maps from baseline tumour maps (Fig. 3a). Anatomically, tumour growth mapped to the DMG network, with greater growth in network than out-of-network brain (Fig. 3b). To examine the specificity of tumour growth to peaks of greatest connectivity in the DMG network, we studied 34 children with tumour progression beyond the primary site and computed the connectivity of each tumour with DMG network peak clusters (Supplementary Table 2), controlling for the distance of each primary tumour to each network peak. Correlative analyses identified greater tumour burden in DMG network peaks with which each tumour was more functionally connected (Fig. 3c). Paired examples of tumour maps at diagnosis and final follow-up illustrate the convergence of tumour growth with DMG network-defined trajectories (Fig. 3d,e). Of note, we did not identify a correlation between network-to-tumour connectivity and tumour volumetric growth (Spearman’s *ρ* = −0.02; *P* = 0.932), consistent with initial survival analyses, which did not identify an association between tumour volume and overall survival. Finally, we investigated whether non-contiguous metastatic DMGs exhibit similar tropism to the DMG network: identifying six children with intracranial parenchymal (non-leptomeningeal) metastases secondary to primary spinal DMG, H3K27-altered, in an independent cohort of children with primary spinal DMG (total *n* = 36). Tumour network overlap maps illustrate almost complete localization of intracranial metastatic lesions to the DMG network, with significantly greater metastatic disease in network than out-of-network brain (Extended Data Fig. 8). Collectively, these data provide evidence linking the anatomic pattern of DMG growth to circuit-specific trajectories within the DMG network.

## DMG incidence reflects neurometabolic development

The early childhood peak incidence of pontine gliomas has been reported since the mid-1950s^50^. Specifically, DMGs occur in a distinct spatiotemporal pattern during childhood and are rare in adulthood (pontine and thalamic DMG peak incidence, 6 and 11 years, respectively)^2^. This aligns with periodic changes in postnatal myelination and neural circuit maturation, concordant with key neurodevelopmental stages and the oligodendrocyte precursor cell (OPC)-like lineage of DMG^51,52^. We hypothesized that the neurometabolic development of brain circuits in the DMG network would explain the spatiotemporal pattern of DMG incidence. To investigate this, we examined the relationship between DMG whole-cohort age incidence and normative [^18^F]fluoro-2-deoxy-d-glucose positron emission tomography ([^18^F]FDG-PET) signal within the DMG network from 0 to 16 years of life^53^. Regression of age incidence and voxelwise mean-squared differences in consecutive, age-averaged signal changes exhibited a strong linear dependence (*R* = 0.612, *P* = 0.0118), with the maximal peak change in [^18^F]FDG-PET signal at 5–6 years of life and the second maximal peak change at 11–12 years of life, coinciding with the respective peak age incidence of pontine and thalamic DMG (Fig. 3f).

We next evaluated whether peak neurometabolic changes were specific to brain regions within the DMG network. First, the [^18^F]FDG-PET signal from each of 46 brain regions, defined as within or out of the DMG network, was extracted and regressed with DMG age incidence, identifying that [^18^F]FDG-PET signal changes in brain regions within the DMG network explained significantly greater variance in incidence than the signal in the rest of the brain (median in-network *R* = 0.327 versus median out-of-network *R* = 0.197; two-tailed Mann–Whitney *U*-test, false-discovery rate (FDR)-corrected *P* = 0.015). Next, the greatest voxelwise changes in [^18^F]FDG-PET signal at each age were identified and spatially compared to DMG network topography. Strikingly, the top two peak positive neurometabolic changes during childhood were spatially dominated by brain areas within the DMG network at ages of peak DMG incidence (5–6 years, pontine peak; 10–11 years, thalamic second peak; Fig. 3f). Anatomically, these peak neurometabolic changes were more similar to the DMG network than expected by chance, in contrast to changes at other ages (Supplementary Table 5). Together, these data characterize the DMG network as a site of spatiotemporally organized neurometabolic development associated with tumour incidence. This alignment defines a neurodevelopmental window in which brain regions within the DMG network undergo their most significant neurometabolic transitions, possibly reflecting critical periods of vulnerability to DMG initiation through neural activity.

## Synaptic gene enrichment in high-connectivity DMG

**Fig. 4:**
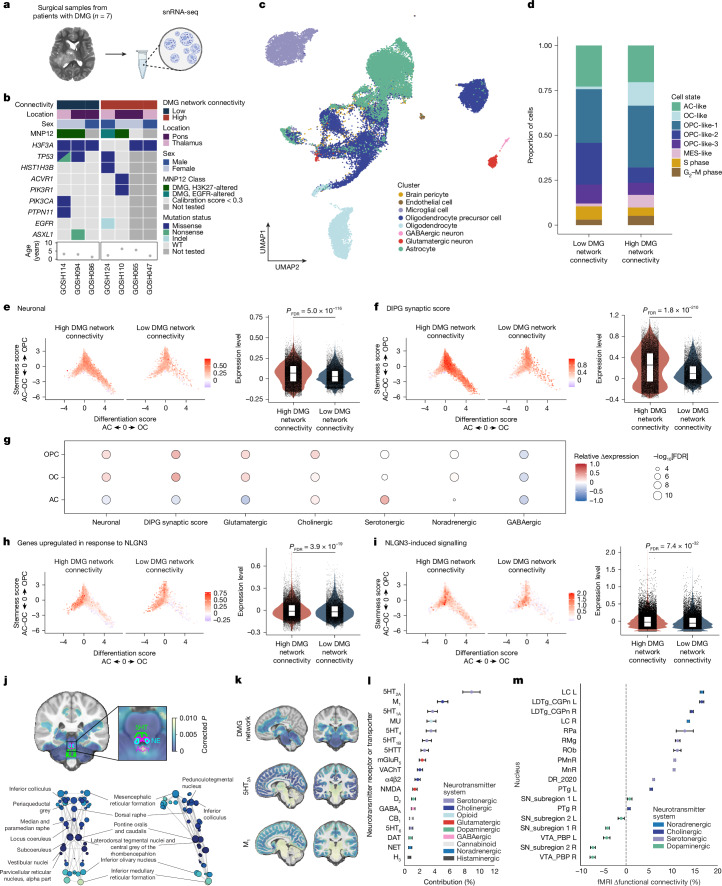
Synaptic gene enrichment in high-connectivity DMG. **a**, Methodological schema. **b**, snRNA-seq cohort characteristics (*n* = 7). WT, wild type. **c**, Uniform manifold approximation and projection (UMAP) of 42,504 single nuclei and identified cell type clusters. **d**, The frequency of malignant cell states in high- and low-connectivity DMG. **e**,**f**, Malignant cell signature expression projected onto cell lineage (*x* axis; astrocytic (AC)-to-oligodendrocytic (OC) differentiation) and stemness (*y* axis; stem to differentiated) scores with corresponding violin plots. The red–blue colour scale indicates the relative gene signature enrichment: neuronal^12^ (**e**) and DIPG synaptic score^3^ (**f**). **g**, Relative neurotransmitter gene signature enrichment in high- versus low-connectivity DMG. **h**,**i**, Malignant cell signature expression projected onto cell lineage and stemness scores with corresponding violin plots: genes upregulated in response to NLGN3^3^ (**h**) and genes encoding NLGN3-induced signalling proteins^3^ (**i**). **j**, Spatial representation of brainstem nuclei DMG network connectivity strength (network peaks dark blue). For visualization, only the strongest 5% of brainstem nucleus-nucleus functional connections are shown. **k**, The top two brain-wide neurotransmitter receptor and transporter density distributions with the greatest contribution to DMG network chemoarchitecture. **l**, The percentage neurotransmitter contribution to overall fit of a multiple linear regression model between brain-wide DMG network functional connectivity and 18 neurotransmitter receptor and transporter density profiles. **m**, Pairwise comparison of nucleus-to-tumour connectivity across short- and long-term survivors of DMG/DIPG demonstrated significantly greater connectivity to cholinergic (*P*_FWE_ = 0.001), serotonergic (*P*_FWE_ = 0.0002) and noradrenergic (*P*_FWE_ = 0.0004) nuclei than dopaminergic nuclei in short-term survivors (one-way analysis of variance (ANOVA) with Tukey’s post-hoc test, *F *= 16.84, *P* = 6.40 × 10^−5^; factor neurotransmitter system with four levels). In **l** and **m**, the dots represent the adjusted *R*^2^ for each neurotransmitter receptor and transporter; the whiskers represent the 95% CI. Signature enrichment across malignant single nuclei (*n* = 32,543) was assessed using a two-tailed Wilcoxon signed-rank test. The violin plots denote median (centre line) and IQR (box limits), the whiskers extend to the most extreme values within 1.5× IQR and individual observations are shown by dots. The diagram in **a** was created using BioRender; Sidpra, J. https://BioRender.com/vi1xgg0 (2026).

**Extended Data Fig. 9:**
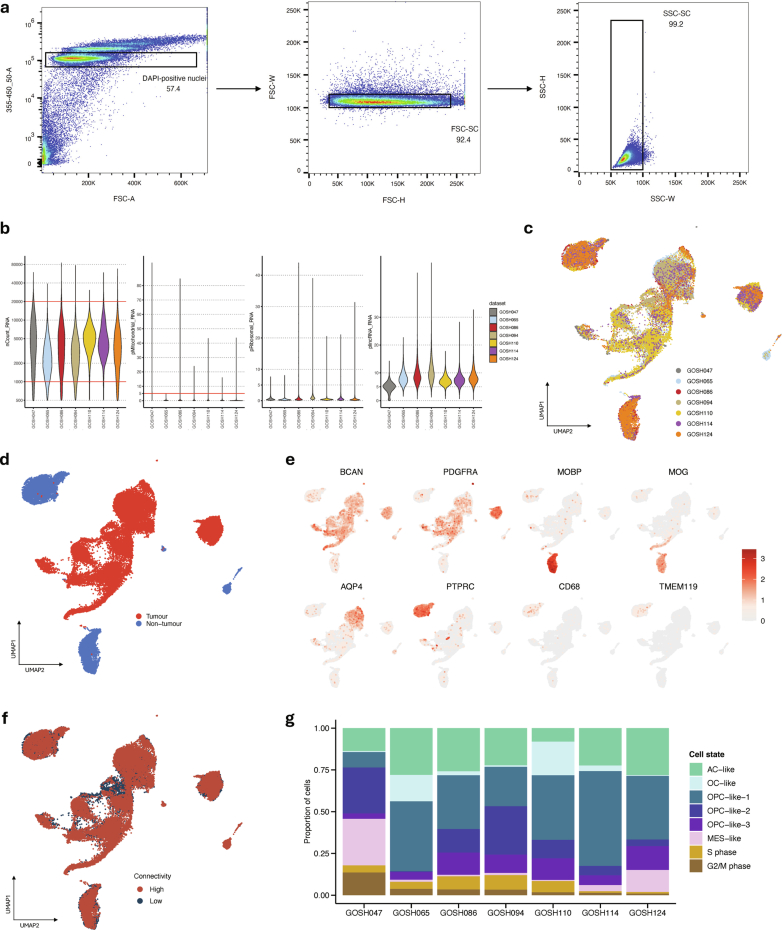
DMG patient snRNA-seq data quality control and integration. (**A**) Representative FACS gating strategy for single-nucleus purification. (**B**) Library quality control per nucleus for the number of detected genes; total transcripts; percentage of mitochondrial transcripts; and percentage of ribosomal transcripts. Red lines indicate applied filtering thresholds. (**C-D**) UMAPs of 42,504 single-nuclei coloured by library (**C**) and malignant vs. non-malignant cell type assignment (**D**). (**E**) UMAP projections highlighting malignant (*BCAN*, *PDGFRA*) and non-malignant cell clusters by expression of canonical markers for oligodendrocytes (*MOBP*, *MOG*); astrocytes (*AQP4*); leukocytes (*PTPRC*); and microglia (*CD68*, *TMEM19*). (**F**) UMAP of 42,504 single-nuclei coloured by tumour DMG network connectivity. (**G**) Frequency of malignant cell states across DMG patient samples.

**Extended Data Fig. 10:**
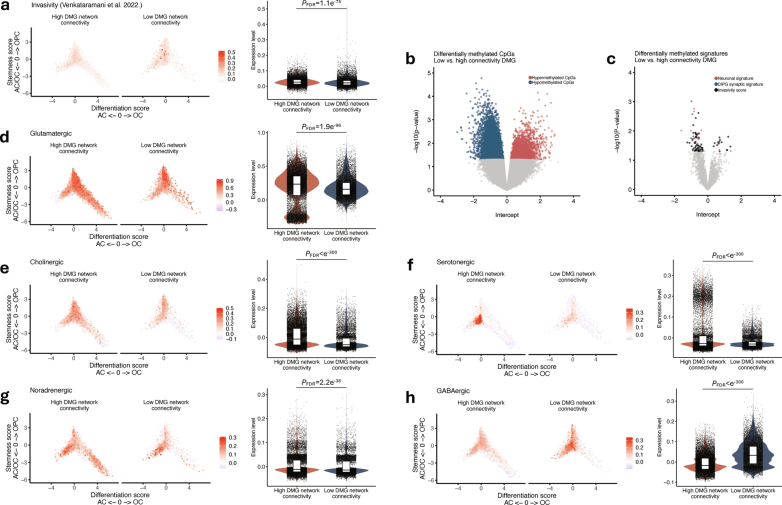
Neuronal and synaptic gene expression in high- versus low-connectivity DMG. (**A**) Malignant cell signature expression projected onto cell lineage (x-axis; astrocytic to oligodendrocytic differentiation) and stemness (y-axis; stem to differentiated) scores with corresponding violin plots. The red-blue colour overlay indicates relative invasivity gene signature enrichment. (**B**) Differential enrichment analysis of tumours with neuroimaging-defined high- and low-connectivity with the DMG network identified 15,522 hypomethylated and 2,873 hypermethylated CpGs (*P* < 0.05). (**C**) Restriction of the CpGs identified in (**B**) to gene signatures previously associated with neuron-glioma interactions demonstrates significant hypomethylation of neuronal (red; two-tailed Mann-Whitney U-test *P* = 1.6e^−5^); DIPG synaptic (blue; two-tailed Mann-Whitney U-test *P* = 5.0e^−8^); and invasivity (black; two-tailed Mann-Whitney U-test *P* = 5.5e^−12^) signature genes in high-connectivity DMG. (**D-H**) Malignant cell signature expression projected onto cell lineage (x-axis; astrocytic to oligodendrocytic differentiation) and stemness (y-axis; stem to differentiated) scores with corresponding violin plots. The red-blue colour overlay indicates relative gene signature enrichment: glutamatergic (**D**); cholinergic (**E**); serotonergic (**F**); noradrenergic (**G**); and GABAergic (**H**). Signature enrichment across malignant single-nuclei (n = 32,543) was assessed using a two-tailed Wilcoxon signed-rank test. Violin plots denote the median (centre line), interquartile range (IQR; box limits), whiskers extending to the most extreme values within 1.5x IQR, and individual observations (dots).

To elucidate the transcriptional programs underpinning phenotypic differences between high- and low-connectivity DMG, we analysed single-nucleus RNA sequencing (snRNA-seq) data from seven fresh-frozen patient surgical samples within our cohort (Fig. 4a,b). Tumour tissue was dissociated and underwent fluorescence-activated cell sorting to isolate single nuclei (Extended Data Fig. 9a). A median of 4,707 nuclei per sample and 1,734 genes per nucleus passed quality control, totalling 42,504 single nuclei (Extended Data Fig. 9b,c). Malignant and non-malignant cell populations were first classified using copy-number variation and refined using Azimuth reference mapping and previously defined marker genes (Fig. 4c and Extended Data Fig. 9d,e). Across malignant cells (*n* = 32,543), OPC-like cells (glial cells that normally engage in neuron-to-glial cell synapses^54^) predominated using reference cell state signatures^52^ (Fig. 4c). DMG transcriptional architecture was conserved between high- and low-connectivity DMG, with no specific cell state expansion (Fig. 4d and Extended Data Fig. 9f,g). Strikingly, however, high-connectivity DMG demonstrated specific enrichment of transcriptional signatures previously associated with the formation of neuron-to-glioma synapses^12^ (Fig. 4e), patient biopsy-isolated DIPG synaptic character^3^ (Fig. 4f) and tumour invasivity^12^ (Extended Data Fig. 10a). Tumour genome-wide bulk DNA methylation data from a larger cohort of children with primary pontine and thalamic DMG (*n* = 36; Supplementary Table 6) confirmed significant hypomethylation of gene CpG sites within these signatures in high-connectivity DMG (Extended Data Fig. 10b,c). Specific enrichment of synaptic genes in high-connectivity DMG held true across a diverse neurotransmitter repertoire, including glutamatergic, cholinergic, serotonergic and noradrenergic signalling (Fig. 4g and Extended Data Fig. 10d–g). This increased expression was particularly prominent in OPC-like tumour cells but persisted with lineage maturation along the OPC-oligodendrocyte (OC) axis, suggesting the presence of a more mature, neuronal-responsive population in high-connectivity DMG (Fig. 4g). Despite the known growth-promoting role of GABAergic signalling on DMG cells^6^, GABAergic signalling was paradoxically decreased in high-connectivity DMG, perhaps reflecting a degree of circuit-dependent plasticity and the relatively short-range nature of GABAergic interneurons located in the local tumour microenvironment rather than projecting from distant brain regions (Extended Data Fig. 10h). Importantly, the activity-dependent release of the paracrine factor NGLN3 is required for DMG progression^7,8^. Previous work has defined genes upregulated in DMG cells exposed to NLGN3 versus control as well as genes encoding the signalling proteins that are responsive to NLGN3 (refs. ^7,8^). High-connectivity DMG exhibited upregulation of both signatures, suggesting increased activity-dependent NLGN3 signalling (Fig. 4h,i). Together, these data demonstrate meaningful, tissue-level differences between high- and low-connectivity DMG. Furthermore, they support preclinical evidence implicating neuromodulatory neurotransmitters in DMG progression^5,9,18^ and suggest increased neuron-to-glioma synaptic integration as a feature of high-connectivity DMG.

## DMG network chemoarchitectural organization

These observations raised the question of whether the DMG network represents a functional architecture comprised of specific neurotransmitter projections. Parcellation of the DMG network into brainstem nuclei identified peaks in the locus coeruleus and subcoeruleus (noradrenergic); parvicellular reticular nuclei, part alpha (serotonergic); pontine oralis reticular nuclei (serotonergic and cholinergic); and laterodorsal tegmental nuclei (cholinergic) (Fig. 4j). As cortical functional topographies align with projections from brainstem neuromodulatory nuclei^55^, we next sought to define the chemoarchitecture of the DMG network (defined as the spatial alignment of brain-wide neurotransmitter receptor and transporter densities with network topography). Leveraging a PET atlas, we modelled the distribution of 18 human neurotransmitter receptor and transporter densities across the DMG network^56^. Dominance analyses identified signalling through serotonergic 5HT_2A_ and cholinergic M1 muscarinic receptors as the greatest contributors to DMG network chemoarchitecture (>25% model fit; Fig. 4k,l). Further, pairwise comparison of brainstem nucleus-to-tumour connectivity in short- versus long-term survivors of DMG demonstrated significantly greater connectivity with cholinergic, serotonergic and noradrenergic nuclei (but not dopaminergic nuclei) in short-term survivors (Fig. 4m). Taken together, these results suggest that tumour connectivity with the DMG network occurs within a chemoarchitectural niche that DMGs are known to exploit.

## DMG resection as network disconnection

**Fig. 5:**
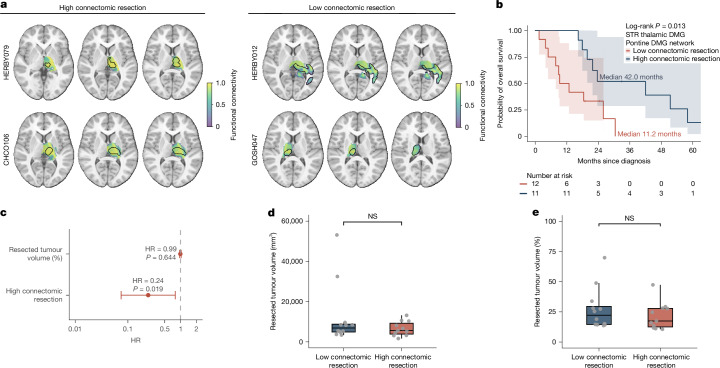
Survival benefit of high connectomic resections in children with primary thalamic DMG. **a**, Tumour resection maps illustrate intratumoural, subregion-specific tumour tissue connectivity with the DMG network in children with thalamic DMG and high-connectomic or low-connectomic subtotal resections across the whole study cohort (*n* = 23). High-connectivity tumour subregions are shown in yellow and low-connectivity tumour subregions are shown in dark blue. Resected tumour margins are defined in black ink. **b**, Kaplan–Meier survival curve showing the prognostic benefit of high connectomic resections in children with incidental subtotal resections (STRs) of thalamic DMG (*n* = 23; median overall survival, 11.2 versus 42.0 months; log-rank test, *P* = 0.013). The error bars represent the 95% CI. **c**, The results of a multivariable Cox proportional hazards model demonstrate that the prognostic impact of high-connectomic resection in children with thalamic DMG is independent of extent of resection (*n* = 23; HR = 0.24, 95% CI = 0.07–0.79, *P* = 0.019). Mean values are represented by solid circles and the whiskers represent the 95% CI. **d**,**e**, No difference was identified in the absolute volume of tumour resected (**d**; *n* = 23; median, 6,892 versus 5,603 mm^3^; two-tailed Mann–Whitney *U* test, *P* = 0.479) or in the volumetric percentage of tumour resected (**e**; *n* = 23; median, 22.2% versus 17.4%; two-tailed Mann–Whitney *U* test, *P* = 0.372) when comparing low- and high-connectivity resections. In **b** and **e**, data are median (centre line) and IQR (box limits), the whiskers extend to the most extreme values within 1.5× IQR and individual observations are shown (dots). NS, not significant.

High-grade gliomas form interconnected, communicating cellular networks^3^. Selective surgical and pharmacological disconnection of network hubs compromises network activity and tumour growth in preclinical models^3,11^. Although neurosurgical interventions for pontine DMG are typically limited to tissue biopsy given the brainstem location of the disease, the prognostic benefit of gross-total resection for thalamic DMG is well reported, if rarely achieved^57^. The role of more limited debulking procedures (defined as a subtotal resection >10% but <90% tumour volume), and whether such procedures positively impact patient survival, remain to be demonstrated. We hypothesized that targeted subtotal resections of high-connectivity thalamic DMG subregions would increase overall survival by disrupting prognostic DMG network connections. To investigate this, we defined tumour resection maps for a subcohort of 23 children with thalamic DMG who underwent subtotal tumour resections (Supplementary Table 7). DMG network connectivity values were overlayed onto tumour resection maps to define the mean connectivity strength of resected and non-resected (residual) tumour subregions (Fig. 5a). A significant survival benefit was observed for patients in whom highly connected tumour subregions were resected when compared with lower-connectivity resections (Fig. 5b). This was robust to the inclusion of the percentage of tumour volume resected as a covariate in a multivariable Cox proportional hazards model (Fig. 5c) and no difference was identified in the absolute or percentage tumour volume resected between groups (Fig. 5d,e), indicating that the prognostic impact of high connectomic resection is independent of resected tumour volume. Although these observations require validation in a prospective clinical trial, our combined survival data suggest a network dependence to DMG tumour progression and demonstrate a prognostic benefit for children with thalamic DMG, in whom gross-total resection was not feasible, following selective, volume-agnostic disruption of intratumoural DMG network connectivity.

## Discussion

Although the recruitment and bidirectional interactions of otherwise healthy neural populations in neuron–glioma networks are well-characterized in preclinical models, the organization and clinical implications of these connections in the living human brain remained to be elucidated. Here, we develop tumour network mapping to demonstrate that pontine and thalamic DMG map to a conserved brain network, connectivity with which is independently and inversely associated with patient overall survival. The tropism of tumour growth for DMG network connectivity peaks reflects directional, neural activity-dependent tumour infiltration in preclinical models^3,5,9^ and offers an explanation for the conserved pattern of DMG tumour anatomic progression^15–17^. Although none of the experiments presented here demonstrate, nor could have demonstrated, a direct relationship between tumour neural activity and tumour growth, convergent data presented across multiple modalities^27^ are consistent with the hypothesis that DMG network connectivity is associated with tumour growth and patient survival. This said, our definition of prognostic brain network connections common to all DMG does not preclude the importance or influence of individual variance in neuron-to-glioma connections within the DMG network that remain to be elucidated. We expect the application of these methods to other glial malignancies will enable the definition of shared and tumour-specific subcircuits important for tumour growth and patient survival.

Neural activity is implicated not only in potentiating glioma growth but also glioma initiation and maintenance^58,59^. We demonstrate that high-connectivity DMGs are enriched for neuronal and synaptic gene programmes and genes upregulated in DMG cells in response to neuronal input, validating DMG network stratification at single-cell resolution. We further show that peak changes in neurometabolic activity across childhood localize to the DMG network, potentially reflecting the developmental maturation and increased neural activity of brain circuits connected to and communicative with DMG as well as a developmental period of increased cellular stress during which DMG may acquire secondary mutations. Emerging human pontine snRNA-seq data spanning the first trimester of life to early adulthood demonstrate upregulated synaptic plasticity gene expression in OPCs at ages of peak DMG incidence, suggesting an interplay between neural, neurometabolic, and transcriptional vulnerability^60^. Whether such an association extends to other cerebral tumours of childhood remains to be demonstrated and requires a greater understanding of tumour subtype-intrinsic neuron–glioma interactions.

The brain-wide prognostic connections represented by the DMG network support preclinical evidence for DMG and other high-grade gliomas occupying an infiltrative niche communicative with brain areas distant from and projecting onto primary tumour cells. Selective surgical disruption of this communication through resection of high-connectivity DMG tumour subregions was associated with longer patient overall survival, suggesting that focal, volume-agnostic lesions of tumour networks may modulate tumour growth, and proposing the DMG network as a testable target for therapeutic neuromodulation in prospective clinical trials. Taken together, our results define a spatially conserved map of prognostic brain network connections in children with DMG and are consistent with preclinical evidence that DMG growth and infiltration are robustly regulated by diverse, brain-wide neuronal interactions. Pharmacological and surgical targeting of these defined vulnerabilities may be critical for the development of novel circuit-level therapeutics for this devastating childhood cancer.

## Methods

### Patient cohorts

This study was approved by the institutional review board of GOSH NHS Foundation Trust (24/HRA/4335) with informed consent waived for retrospective analyses. Primary data collection was also approved by the institutional review boards of each participating study site before study commencement in accordance with the Declaration of Helsinki as amended^61^. Three independent cohorts of children diagnosed with primary pontine DMG/DIPG or thalamic DMG were identified and analysed retrospectively: (1) a discovery cohort from GOSH, UK; (2) an independent, multicentre external validation cohort from the Children’s Hospital Colorado (CHCO), USA; University of São Paulo, Brazil; Institute of Neurosurgery Dr. Alfonso Asenjo, Chile; and the HERBY clinical trial (NCT01390948)^24^; (3) a second, independent cohort of children with biopsied pontine DMG, H3K27-altered, enrolled on PNOC clinical trials. Participating centres are major international institutions with recognized subspecialty expertise in paediatric neuro-oncology. PNOC trial inclusion criteria have been previously reported. Inclusion criteria were identical across cohorts 1 and 2, with children reported between January 2000 and January 2024 inclusive:

1. Consensus clinical–radiological diagnosis of DIPG, defined as the rapid (<3 month) onset of cranial nerve palsies, long-tract signs or cerebellar signs accompanied by MRI identification of an expansile, diffusely infiltrative mass arising from and involving ≥50% of the pons and which is T1-hypo- or iso-intense, T2-hyperintense and lacks or has minimal contrast enhancement^20–22^.
  or
2. Neuropathological (tissue biopsy) diagnosis of a primary pontine or thalamic DMG, H3K27-altered, consistent with World Health Organization 2021 criteria^1^.
  and
3. Child (aged under 18 years).
4. Treatment-naive brain MRI to include a three-dimensional volumetric T1-weighted sequence with high spatial resolution and two-dimensional T2-weighted or T2-weighted fluid-attenuated inversion recovery (FLAIR) sequences, both with a slice thickness of ≤5 mm.
5. Complete clinical data available for analysis, defined as treatment covariates and time to last follow-up or death, as appropriate.

Neuropathological diagnosis was required for the inclusion of long-term survivors of DMG (defined a priori as an overall survival ≥18 months from diagnosis), irrespective of tumour primary pontine or thalamic location^23^. Individuals with brain MRI of insufficient quality for analysis (for example, partial brain coverage or severe artefact(s) due to metal or motion) were excluded. Individuals lost to follow-up within 18 months (before the threshold for long-term survivorship) were excluded due to indeterminate outcome. Inclusion was based exclusively on the availability of the above data with no exclusions based on race, ethnicity, sex or other characteristics.

### Clinical definitions

Age at diagnosis was defined as age at first brain MRI. Extent of resection was categorically stratified as unoperated, biopsy only, subtotal or gross-total by a board-certified paediatric neuroradiologist on postoperative brain MRI performed within 48 h of surgical resection. Gross-total resection was defined as the removal of all contrast-enhancing tumour volume while subtotal resection was defined as a greater than 10% but less than 90% reduction in tumour volume^62,63^. Further data were also collected for treatment modality, including but not limited to adjuvant radiotherapy and chemotherapy. Overall survival was defined as the time from diagnosis to death. Surviving patients were censored at the date of database closure (20 September 2024). Long-term survivors who did not participate in follow-up were censored at the date of last clinical follow-up. The time to last clinical follow-up was defined as the time from diagnosis to last clinical encounter.

### Neuroimaging acquisition, segmentation and preprocessing

All individuals in the discovery cohort underwent brain MRI on the Siemens MAGNETOM Avanto (1.5 T) or Prisma (3 T) scanner. Given the retrospective multicentre nature of the study, there was significant heterogeneity in terms of scanner manufacturer and magnet field strength across the external validation cohorts. Patient brain MRI data were downloaded from institutional picture archiving and communications systems as digital communications in medicine (DICOM) files. Tumours were manually segmented in three planes (axial, coronal and sagittal), with reference to all available MRI sequences acquired at diagnosis, by researchers with expertise in neuroimaging (J.S., V.L., F.S., B.S.P. and R.S.O.) using ITK-SNAP (v.4.2.0)^64^. This yielded a binary tumour mask which was reviewed and, if necessary, corrected in consensus review with three board-certified paediatric neuroradiologists (K.M., A.B. and S.S.) consistent with a standardized, pan-institutional protocol for neuro-oncology MRI segmentation. After segmentation, the DICOM files were converted to Neuroimaging Informatics Technology Initiative (NIfTI) format using the nifti2dicom Python library (v.1.2.6). Brains were skull-stripped and extracted using SynthStrip and then registered to the Montreal Neurological Institute (MNI) MINC1 60MB paediatric template in MNI space (1.0 × 1.0 × 1.0 mm) using ANTs symmetric image normalization (SyN) registration^65–67^. Cost-function masking was applied to tumour masks to remove them from computations^68^. Tumours were then transformed to MNI space using forward SyN transforms and nearest-neighbour interpolation^69^. All registered tumours were manually compared to original, patient-specific neuroimaging and reviewed for neuroanatomic accuracy in MNI space by a board-certified paediatric neuroradiologist (K.M.). All segmentations were performed blinded to patient identity and clinical outcome.

### Human connectome data acquisition and pre-processing

Integrative analyses of fMRI and dMRI data were performed to clinically translate the previously reported neural integration of DMG through tumour-specific whole-brain connectivity. High-resolution normative structural (three-dimensional T1-weighted) and resting-state fMRI were obtained for 1,000 healthy children aged 9.0 ± 0.2 years from the Adolescent Brain Cognitive Development Study (ABCD1000)^35^. High-resolution normative structural (three-dimensional T1-weighted) and advanced dMRI (multiple *b* values and directions) were obtained for 497 healthy children aged 13.0 ± 2.9 years from the Human Connectome Project (HCP) Lifespan Development dataset^36,37^. Cohort demographics and imaging parameters for both normative connectomes have been previously reported^35–37^. fMRI data preprocessing was performed using the Computational Brain Imaging Group preprocessing pipeline (https://github.com/bchcohenlab/BIDS_to_CBIG_fMRI_Preproc2016)^70,71^. dMRI data were preprocessed in line with the HCP minimal processing pipelines (https://github.com/Washington-University/HCPpipelines.git)^72,73^ and the resulting distortion-corrected *b* = 0 image was registered to MINC1 60MB through ANTs SyN registration^74,75^.

### Tumour location mapping

#### Identifying tumour voxels associated with patient overall survival

Tumour location frequency maps were plotted in MNI space as the sum of all binary tumour masks using ANTs iMath functions. To establish whether tumour anatomic location alone was associated with patient overall survival, we first implemented a univariate VLSM approach. A voxelwise general linear model with permutation testing was applied to the data to compare voxels between short- and long-term survival groups in NiiStat (https://github.com/neurolabusc/NiiStat)^29,76^. Analyses were limited to voxels involved in ≥5% tumours, controlling for tumour volume and correcting for multiple comparisons^76–79^. The significance at each voxel was assessed using 2,000 Freedman–Lane (shuffled) permutations. Given the conservative nature of voxel-based permutation testing^80^, we replicated univariate VLSM by further implementing threshold-free cluster enhancement in FMRIB Software Library (FSL) permutation analysis of linear models (PALM)^77,81^. Given the significantly shorter overall survival in children with pontine versus thalamic DMG, we also performed univariate VLSM across pontine DMG/DIPG and thalamic DMG separately to control for the prognostic effect of primary tumour location. In each of these analyses, we limited VLSM to voxels involved in at least seven patient tumours to avoid single case-driven effects, consistent with best-practice recommendations^76,78,79^. We replicated analyses implementing multivariate support vector regression (SVR)-VLSM, which can identify complex dependencies between neighbouring voxels that traditional univariate VLSM approaches cannot^82,83^. SVR-VLSM was implemented in SVR-LSM^84^, a MATLAB toolbox for multivariate and cluster-based lesion symptom mapping, and limited to voxels involved in ≥5% tumours, controlling for tumour volume using direct total lesion volume control and correcting for multiple comparisons. Significance was assessed using 10,000 permutations. The results of univariate VLSM and multivariate SVR-VLSM were considered to be significant at a two-tailed FWE-corrected *P* < 0.05 and *P *< 0.005, respectively, as in previous work^29^.

#### Interrogating the impact of tumour VLSM map scores on patient overall survival

The resulting univariate and multivariate VLSM maps represented unified, group-level maps of the prognostic strength of each voxel across overlapping tumours. We examined whether VLSM maps could determine patient overall survival based on tumour location alone, irrespective of the presence or absence of voxels significantly associated with overall survival in the discovery cohort, using data from an independent, multicentre external validation cohort, with identical inclusion criteria and identical methodology for tumour segmentation and brain MRI pre-processing. Each binary tumour segmentation in the external validation cohort was weighted by overlapping voxels in each VLSM map. A mean ‘tumour VLSM map score’ was then computed as the average of these weighted values for univariate and multivariate analyses, respectively^25^. Patients were stratified into two risk groups using a median cut-off for each VLSM map: high risk (defined as median or above-median tumour VLSM map score) and low risk (defined as below-median tumour VLSM map score). Kaplan–Meier models were then used to model the overall survival for each VLSM map score-defined risk group, with the difference between groups evaluated using a two-tailed log-rank test. We also examined whether the tumour VLSM map score could act as an independent predictor of patient overall survival through inclusion in both univariable and multivariable Cox proportional hazards models incorporating clinical covariates for patient demography (age at diagnosis and sex) and clinical management (extent of resection, and completion of adjuvant radiotherapy and chemotherapy). In all instances, the proportional hazards assumption was verified before analysis and the results were expressed as HRs with 95% CIs and corresponding *P* values.

### Tumour network mapping

#### Modelling tumour networks from tumour locations using normative fMRI

To define patient-specific tumour network maps, every tumour location was used as a ‘seed’ from which to compute resting-state functional connectivity with every other voxel in the brain across all individuals in the ABCD1000 normative fMRI dataset^26^. The correlated time series (R-map) from seeded fMRI data per patient was then Fisher *z*-transformed and compared using a two-tailed, one-sample* t* test, thereby unifying each R-map into patient-specific voxelwise *T*-maps: a statistical representation of each tumour’s resting-state functional connectivity with all other brain voxels^26,85,86^.

#### Identifying functional connections associated with overall survival

Resting-state functional connections associated with patient overall survival were identified by performing whole-brain general linear model permutation testing, correcting for multiple comparisons, and implemented in FSL PALM, adapting methods from lesion network mapping^77,81^. First, a voxelwise one-sample *t* test was performed across short-term survivor patient *T*-maps (as previously defined, *n* = 106), correcting for tumour volume as a covariate with 2,000 Freedman–Lane sign-flip permutations^77^. These analyses generated a unified, group-level statistical spatial map of significant voxels with greater and more consistent positive or negative resting-state functional connectivity across short-term survivor tumours—the DMG network. Next, to identify functional connections significantly different between short-term and long-term survivors, an independent voxelwise Aspin–Welch test was performed across all patient *T*-maps (*n* = 125), controlling for tumour volume as a covariate^29^. Hereafter, we refer to this methodological framework as tumour network mapping. Regions of peak prognostic significance in the DMG network were identified using an unsupervised, cluster identification algorithm via FSL Cluster^87^ using a minimum voxel count threshold ≥20^75^. DMG network cluster peak significance values, centre of gravity coordinates and neuroanatomical location, hereafter termed network nodes or peaks, were reported^88^.

#### Examination of DMG network robustness

To investigate the robustness of the DMG network to statistical choices, we compared voxelwise as opposed to threshold-free cluster enhancement and FWE as opposed to FDR approaches. We also examined the robustness of the DMG network to methodological choices and clinical covariates using the following experiments conducted in PALM: covarying patient overall survival with patient age at diagnosis and sex; restricting to pontine DMG/DIPG only; restricting to biopsied pontine DMG only; restricting to thalamic DMG only; and increasing the threshold for long-term survival from ≥18 to ≥24 months, consistent with both clinically reported thresholds^23,38^. While all tumour segmentations normalized to MNI space were manually checked for neuroanatomic accuracy by a board-certified paediatric neuroradiologist (K.M.) against patient-specific imaging before seeding in ABCD1000, to further rule out the possibility that tumour-induced neuroanatomical distortions might influence tumour-specific normative functional connections, we also conducted an additional experiment covarying patient overall survival with a measure of mean peritumoral anatomic distortion. While also potentially reflective of patient head size, age and sex, the mean voxelwise SyN deformation fields required to normalize each patient brain to MNI space, excluding the tumour cost-function mask, were used as a measure of mean peritumoral anatomic distortion. Furthermore, given the brainstem-predominant location of DMG, we sought to exclude the possibility of the DMG network being derived from or confounded by fMRI signal originating within surrounding cerebrospinal fluid (CSF) (for example, secondary to heartbeat or CSF pulsations)^89^. We verified this by implementing a voxelwise partial correlation analysis when computing whole-brain-to-tumour connectivity for each patient, controlling for temporal signal derived from a fourth ventricular CSF mask^90^. Tumour network mapping was then repeated in PALM across these patient tumour functional connectivity maps with CSF signal partialled out^54^. We also tested the convergence of our results across three normative connectomes with different preprocessing methods: ABCD1000^35^, Yeo1000^39^ and GSP1000^40^. DMG network signal origin was also examined using increasingly strict, stepped statistical thresholds to visualize and eliminate noise. Finally, we also replicated analyses in PALM using a continuous, rather than binary, contrast for patient overall survival.

The correspondence in brain-wide spatial topography between the DMG network and each control network was assessed using both label permutation^32,91^ and spin permutation^91,92^ tests. First, the whole-brain spatial Spearman’s correlation, or ‘spatial similarity’, between each control network and the DMG network was compared against a null distribution of 10,000 permuted network results generated by randomly permuting short- and long-term survival labels 10,000 times across patients in the discovery cohort, yielding an empiric *P* value reflecting the significance of each observed spatial similarity. Next, spatial autocorrelation-preserving permutation testing, or ‘spin testing’, was achieved by parcellating the DMG network into paediatric MINCI 60 MB SynthSeg masks^65^ and randomly rotating the spherical projections of the parcellated network 10,000 times. Each control network was parcellated into the same brain regions and its spatial similarity to each randomly rotated DMG network computed. The *P* value for each spin test was computed as for each label permutation test. Both spin and label permutation tests were performed and reported given the widespread use of spin testing despite reports that spin permutation tests probably inflate the statistical significance of spatial similarity between network maps, in contrast to label permutation-based approaches. Finally, the convergence of brain regions identified across all control analyses was also compared through the visualization of control network overlap maps plotted as overlapping binarized voxels surviving statistical thresholds.

#### Examination of network convergence through tumour network mapping on a normative structural connectome

Fibre orientations in preprocessed diffusion data were estimated using GPU-accelerated FSL Bayesian estimation of diffusion parameters obtained using sampling techniques (BEDPOSTX). Each tumour mask was transformed into participant-specific diffusion space and used as a ‘seed’ in GPU-accelerated probabilistic tracking with crossing fibres (ProbtrackX) in FSL with the following, default parameters: number of samples = 5,000; curvature threshold = 0.2; step length = 0.5 mm; subsidiary fibre volume fraction threshold = 0.01. *b* = 0 images were automatically segmented into CSF and grey matter with SynthSeg and were used as ‘exclusion’ and ‘waypoint’ masks, respectively in probabilistic tractography to obtain patient-specific whole-brain-to-tumour structural connectivity data^65^. The streamline counts for each voxel were normalized by dividing each value by the total number of streamlines. Participant-level structural connectivity data were transformed to MINC1 60 MB and averaged across all individuals to generate population-level structural connectivity data for each tumour. Tumour-specific group-level structural connectivity maps were then analysed in PALM, in an identical manner to tumour network mapping described above for fMRI data, to examine the voxelwise association of tumour structural connectivity with overall survival^29^. Convergence of control structural networks to the DMG structural network was tested through label and spin permutation analysis of spatial similarity, as described.

### Reproducibility of the DMG network and the role of independent datasets

To ensure reproducibility of the DMG network and to examine its prognostic importance, we replicated these analyses across both independent, external validation cohorts, with identical methodology for tumour segmentation; brain MRI preprocessing; and tumour network mapping across functional and structural connectivity data. Convergence of both external networks to the DMG network was tested through label and spin permutation testing of spatial similarity, as previously described.

### Evaluating the prognostic significance of the DMG network

#### Examining the impact of tumour functional connectivity on overall survival

We assessed the impact of mean network-to-tumour connectivity on patient overall survival. Mean network-to-tumour functional connectivity was derived by weighting the functional connections of each patient tumour to the DMG network with *T* values in the DMG network, restricted to only prognostically significant voxels, and then computing the mean of this weighted tumour connectivity. Hereafter, we refer to this as each patient’s DMG network-to-tumour functional connectivity. Such an approach has been used to test the out-of-sample robustness of connections associated with clinical improvements after effective deep brain stimulation for obsessive–compulsive disorder^25,66^, Parkinson’s disease^25^, Gilles de la Tourette’s disorder^25^ and dystonia^25^. Given systematic differences in absolute functional connectivity between pontine and thalamic tumours, for analyses in which both tumour locations were included, network-to-tumour connectivity values were normalized within each anatomic location to ensure that survival associations reflected relative network connectivity within a given anatomic context rather than gross, location-dependent differences in tumour connectivity. Patients were then stratified into two risk groups using a median cut-off: high risk (defined as median or above-median network-to-tumour connectivity) and low risk (defined as below-median network-to-tumour connectivity). Kaplan–Meier models were used to model overall survival for each connectivity-defined risk group, with the difference between groups evaluated using a two-tailed log-rank test. We also examined whether network-to-tumour connectivity acts as an independent predictor of patient overall survival through inclusion as a variable in both univariable and multivariable Cox proportional hazards models, as described for tumour location mapping analyses.

To confirm out-of-sample prediction, we fitted clinical-only and combined clinical-connectivity multivariable Cox proportional hazards models exclusively on the discovery cohort, incorporating patient age and sex and tumour location, DMG network-to-tumour connectivity, extent of resection, and adjuvant radiotherapy and chemotherapy. Model coefficients were subsequently frozen and the fully parameterized models were applied to generate overall survival estimates across the independent external validation cohort. Prognostic discrimination was quantified using the Harrell concordance index (*C* index) calculated from model-derived risk scores. The incremental predictive value of network-to-tumour connectivity was assessed as the difference in *C* index between combined and clinical-only models (Δ*C* index) across the external validation cohort. The Δ*C* index and the statistical uncertainty around the Δ*C* index were estimated using paired nonparametric bootstrap resampling of patients within the external validation cohort with 2,000 iterations. A 95% CI was calculated using the percentile bootstrap method. Two-tailed *P* values were derived from the bootstrap distribution of Δ*C* index relative to the null hypothesis Δ*C* index = 0.

To ensure that the prognostic impact of DMG network-to-tumour connectivity was robust to methodological choices, we repeated the above survival analysis using an orthogonal method to compute DMG network-to-tumour connectivity for each patient. DMG network peaks were used as weighted seeds in ABCD1000 to obtain a brain-wide distributed map representative of functional connectivity with brain regions of increased or decreased risk of shorter overall survival^29^. Each patient tumour-specific functional connectivity map across the external validation cohort was spatially compared to this precomputed connectome discovery DMG network peak functional connectivity map with a whole-brain spatial Spearman’s correlation^26,29,31,67^. The patients were stratified into high- and low-risk groups on the basis of a median cut-off in spatial similarity values, whereby high spatial similarities were indicative of high DMG network-to-tumour functional connectivity. We also repeated survival analyses using DMG network-to-tumour mean functional connectivity with maximum connectivity, minimum connectivity and the sum of connectivity values. Lastly, survival analyses were replicated across the DMG network as derived from Yeo1000 and GSP1000 adult connectomes, as well as tumour structural connectivity data obtained from the DMG structural network.

#### Testing the robustness of DMG network prognostic impact across DMG histone status and co-mutational burden

Given the potential for canonical and non-canonical oncohistone variants^41,93^ and recent evidence for two prognostic, molecularly defined DMG subgroups containing co-mutations in either *TP53* or *BRAF*/*FGFR1*^ (refs. 23,94^), we compared the mean network-to-tumour connectivity of each tumour in a subcohort of 87 children with paediatric solid tumour next-generation DNA panel sequencing using two-tailed Mann–Whitney *U* tests. The prognostic importance of pontine network-to-tumour connectivity was also confirmed across our external validation cohort restricting to children with biopsied pontine DMG only.

#### Specificity testing of DMG network prognostic impact

To ensure the prognostic specificity of the DMG network, the impact of DMG network-to-tumour connectivity on overall survival was assessed against across two independent cohorts of adult patients (aged >18 years) with GBM, *IDH*-wild type: (1) a previously reported, open-access 248-patient cohort from the University of California, San Francisco (UCSF-PDGM)^46^ and (2) a newly generated institutional 272-patient cohort from the National Hospital for Neurology and Neurosurgery, Queen Square, London. Patients with midline tumours in the UCSF-PDGM cohort (*n* = 9) were excluded as tumours were not tested for histone alterations and a diagnosis of DMG could therefore not be wholly excluded. Previously generated tumour segmentation masks in the UCSF-PDGM data were checked against available T2-FLAIR sequences while tumour segmentation masks for patients at Queen Square were manually segmented from all available MRI sequences at diagnosis per patient, as previously described for children with DMG. GBM locations were used as seeds in the ABCD1000 connectome^26^ to obtain brain-wide maps of tumour functional connections across both cohorts, enabling the computation of DMG network-to-tumour connectivity per patient. Survival analyses were replicated using identical methods and with the inclusion of clinical covariates which are reported to influence the overall survival of adult patients with GBM, *IDH*-wild type: patient demography (age at diagnosis and sex); *O*^6^-methylguanine-DNA methyltransferase (*MGMT*) promoter methylation; and clinical management (extent of resection, and completion of adjuvant radiotherapy and chemotherapy)^70^. Furthermore, we investigated whether the prognostic effects of the DMG network reflected underlying common structure within the paediatric functional connectome^47^. We first computed the row-summation vector of the group-average connectivity matrix of the ABCD1000 connectome (∑*C*), using previously reported methods^47^. Next, we generated a ∑*C*-residual DMG network by regressing out ∑*C* from each discovery cohort patient *T*-map and reperformed tumour network mapping as previously described. ∑*C*-to-tumour and ∑*C*-residual DMG network-to-tumour functional connectivity was computed as previously described. To test the prognostic impact of ∑*C* alone and the DMG network with ∑*C* regressed out, respectively, connectivity scores were then used for Kaplan–Meier and univariable and multivariable Cox proportional hazards survival analyses. Spatial similarities of ∑*C* and the ∑*C*-residual DMG network to the primary result DMG network were tested using both spin and label permutation tests.

### Evaluating the role of the DMG network in tumour anatomic progression

#### Tumour progression mapping

We assessed whether tumour anatomic progression mapped to DMG network trajectories. A subcohort of 71 individuals who underwent brain MRI within 2 months of death were identified; final follow-up tumours were segmented; and brain MRIs were preprocessed in an identical manner to that described above. Segmented tumour progression maps, not including baseline tumour volume, were combined across this subcohort to generate a frequency map of new tumour growth. Patterns of tumour progression were qualitatively reviewed by a central panel of three board-certified paediatric neuroradiologists (K.M., A.B. and S.S.) and compared to previously published post mortem series^15,17^. The volume of new tumour growth within the DMG network and out of the DMG network was also computed and compared using a Wilcoxon signed-rank test. This subcohort was then further refined to 34 individuals whose tumour exhibited progression beyond the primary tumour site to other brain areas. To test the hypothesis that tumours infiltrate more functionally connected DMG network nodes, the degree of concordance between functional connectivity and tumour burden per node was assessed across pre-defined DMG network nodes for all 34 individuals using methods adapted from network diffusion modelling^95^. DMG network nodes, or peak clusters, were defined as previously described using FSL cluster and brain regions representative of each node generated with paediatric MINCI 60 MB SynthSeg masks^65^. The voxelwise degree of tumour overlap with each of the 18 DMG network nodes was computed for each tumour progression map, defined as the proportion (percentage overlap) of each DMG network node that intersected each tumour. Simultaneously, mean node-to-tumour functional connectivity values were computed from each patient-specific functional connectivity map. These mean functional connectivity values were then distance-corrected by division by the Euclidean distance (in mm) from the centroid of each DMG network node to each baseline tumour. Intraindividual Spearman’s correlation of distance-normalized functional connectivity values and tumour burden per node were computed and averaged for group-level inference. Group-level 95% CIs were obtained by bootstrapping with 2,000 iterations.

#### Spinal DMG cohort

An independent cohort of six children with intracranial parenchymal (non-leptomeningeal) metastases secondary to a biopsy-confirmed diagnosis of primary spinal DMG, H3K27-altered, were identified from a larger international cohort of 36 children with primary spinal DMG. Intracranial metastatic tumours were preprocessed, segmented and transformed to MNI space as previously described before anatomic comparison with the DMG network. The volume of metastatic disease within the DMG network and out of the DMG network was also computed and compared using a Wilcoxon signed-rank test.

### Evaluating the spatiotemporal relationship between DMG incidence and brain metabolism

#### Modelling DMG incidence with [^18^F]FDG-PET data

DMG incidence was extracted from frequency of diagnosis at each age by rounding age at diagnosis across the whole study cohort down to the nearest year. Age-averaged [^18^F]FDG-PET data were obtained from a population-level paediatric [^18^F]FDG-PET atlas comprised of 795 children with inclusion criteria and acquisition parameters as previously reported^53^. The voxelwise mean-squared difference between age-consecutive whole-brain standardized uptake values was computed, reflecting the mean difference in [^18^F]FDG-PET uptake between consecutive ages, and plotted against DMG incidence data across childhood (0–16 years) rounded to the nearest whole year. A linear regression model was fit between age-wise mean-squared difference and whole-cohort age incidence to assess the variance explained by model fit. 95% CI for the variance in age incidence data explained by [^18^F]FDG-PET signal changes was evaluated using 2,000 bootstrap iterations. Next, to assess the spatial specificity of the linear relationship between [^18^F]FDG-PET signal and age incidence, age-averaged [^18^F]FDG-PET data were parcellated according to paediatric MINCI 60 MB SynthSeg anatomic masks (*n* = 46). Brain region anatomic masks were defined as within the DMG network if ≥50% of voxels in each mask overlapped with those in the DMG network; brain regions not meeting this criterion were categorized as out of the DMG network. Region-wise and age-wise [18 F]FDG-PET signal changes within and out of DMG network were then compared in relation to their linear regression model fit with age incidence using a two-tailed Mann–Whitney *U* test with Benjamini–Hochberg correction for multiple comparisons.

#### Spatiotemporal specificity of peak childhood [^18^F]FDG-PET changes

[^18^F]FDG-PET maps were compared to identify voxelwise differences between consecutive ages. The age at which the maximal difference in normalized [^18^F]FDG-PET signal occurred across all ages was identified per voxel and assigned this age value. Voxelwise spatial maps of age-wise peak metabolic changes were thereby generated and compared for age-wise spatial similarity with the DMG network with label permutation and spin permutation tests, as described.

### Neuropathological validation and chemoarchitectural characterization of the DMG network

#### Patient tumour tissue collection and dissociation for snRNA-seq

Tumour tissue samples (*n* = 7) collected at the time of primary biopsy or resection were snap-frozen and processed for snRNA-seq analysis under BRAIN UK approval^96^. Tumour tissue was placed in 1.0 ml prechilled nucleus isolation medium containing 10 mM Tris-HCl pH 8.0, 250 mM sucrose, 25 mM KCl, 5 mM MgCl_2_, 0.1% Triton X-100, 0.1 mM DTT, 1× protease inhibitor cocktail (Promega) and 1% Protector RNase Inhibitor (Merck)^97^. Tumour tissue was then homogenized using a Wheaton Douce homogenizer (10 strokes loose pestle followed by 10 strokes fine pestle). Homogenized tissue was filtered through a 40-µm cell strainer and centrifuged (900*g*) for 10 min at 4 °C. The supernatant was aspirated to a residual 200 µl volume without disturbing pelleted nuclei and resuspended to total volume 1.0 ml using resuspension buffer (PBS with nuclease-free bovine serum albumin (1%) containing 0.5% Protector RNase Inhibitor). The samples were then centrifuged under the same conditions a second time and the supernatant aspirated to a residual 200 µl volume without disturbing pelleted nuclei which were then stained with DAPI (0.1 µg ml^−1^) and resuspended to 500 µl total volume for sorting. All of the procedures were performed using aseptic technique on ice or at 4 °C.

Single-nucleus sorting was performed using a BD FACSymphony S6 cell sorter running FACSDiva v.9.0 using a 355 nm laser (band pass filter 450/50, long pass filter 410). Unstained and single-stained controls were included for all tumours. A standard gating strategy was applied to all of the samples (Extended Data Fig. 9a). Nuclei were identified by positive staining for DAPI. Doublets were excluded by stringent singlet-gating based on forward scatter height (FSC-H) versus width (FSC-W) and side scatter height (SSC-H) versus width (SSC-W). Singlet nuclei were sorted, using a 70-µm nozzle, into PCR tubes containing 0.2 µl prechilled resuspension buffer and centrifuged (200*g*) for 1 min at 4 °C twice before counting and processing for whole-transcriptome amplification, library preparation and sequencing.

#### snRNA-seq data generation

snRNA-seq libraries were generated using the 10x Chromium Controller and Chromium Next GEM Single Cell 3’ kit v.3.1 according to the manufacturer’s instructions (CG000315 Rev F; https://cdn.10xgenomics.com/image/upload/v1722285481/support-documents/CG000315_ChromiumNextGEMSingleCell3__GeneExpression_v3.1_DualIndex__RevF.pdf). Generated libraries were sequenced using NextSeq 2000 P3 XLEAP-SBS Reagent Kits (Illumina) at targeted 20,000 reads per nucleus.

#### snRNA-seq data analysis

Sequencing fastq files were processed with cellranger v.8.0.1, using the GRCh38 2024-A reference obtained from 10x Genomics (https://www.10xgenomics.com/support/software/cell-ranger/downloads#reference-downloads), and with the chemistry flag set to ‘threeprime’ and other arguments set as default. The resulting filtered matrix h5 files were analysed in R using Seurat v.5.3.1. Quality-control metrics for mitochondrial and ribosomal genes were calculated using the function PercentageFeatureSet with regular expression patterns ‘^MT-’ and ‘^PR[SL]’, respectively. Cells were filtered to have at least 1,000 and no more than 50,000 UMI counts and no more than 10% total counts attributed to mitochondrial genes.

Copy-number estimation was performed on filtered count matrices using infercnv v.1.26.0 and SCEVAN v.1.0.3. In both pipelines, a reference dataset was used comprising normal brain expression data from GSE168408, selecting only samples from individuals aged <21 years^98^ such that 500 each of neurons, astrocytes, oligodendrocyte precursor cells, oligodendrocytes, endothelial, and vascular leptomeningeal cells, and 1,000 CD45^+^ immune cells were retained at random.

Cell type assignment was performed in Azimuth v.0.5.0 using the human motor cortex reference and filtering for class confidence score >0.5. Additional cell type assignment was performed using specific gene markers: *BCAN* and *PDGFRA* for malignant cells; *PTPRC* (also known as *CD45*) for immune cells; *CD68* as a general macrophage and microglia marker; and *P2RY12* and *TMEM119* for microglia. Detection of lymphocytes was attempted using *CD3E*, *CD4* and *CD8A* but no significant expression was observed. Oligodendrocytes were confirmed using *MOBP* and *MOG* expression; astrocytes were confirmed using expression of *AQP4*.

Normalization and variable feature detection were performed using the function SCTransform with variable features set to 2,000. Dataset integration was performed using Harmony v.1.2.4 with theta = 2, sigma = 0.1, dims.use = 1:30 and lambda estimation determined empirically by the algorithm. UMAP dimensionality reduction was performed on the first 20 dimensions of the Harmony output. Graphical clustering was obtained using FindNeighbours k.param = 20 and FindClusters resolution = 0.2.

Stemness score and differentiation score for triplot projections were calculated using methods and gene signatures from a previous study^52^. Stemness scoring was performed by obtaining the maximum of either astrocytic (AC) or oligodendrocytic (OC) state. If the higher score was AC, it was multiplied by −1. If either score was ≤0, the value was set to 0 with jitter. Differentiation scoring was obtained through subtraction of the maximum of either AC or OC from the OPC score. The expression of previously defined gene signatures in high- and low-connectivity DMG malignant cells was compared: neuronal^12^, invasivity^12^, DIPG synaptic score^3^, and NLGN3-upregulated^8^. The median difference in expression between high- and low-connectivity DMG per signature was computed and evaluated using a two-tailed Wilcoxon signed-rank test with Benjamini–Hochberg correction for multiple comparisons.

#### DNA methylation profiling

For a subset of individuals treated at GOSH or CHCO (*n* = 29), DNA was extracted from primary resection-derived 50-μm formalin-fixed paraffin-embedded (FFPE) tissue, taken either as 5 μm × 10 μm rolls or as macrodissected sections to enrich for tumour content, using the Maxwell 16 FFPE Tissue LEV DNA Purification Kit on a Maxwell 16 Research Instrument (Promega), consistent with the manufacturer’s instructions. Up to 500 ng of eluted DNA was subjected to bisulphite conversion using the Zymo EZ DNA Methylation-Gold Kit (ZymoResearch). Bisulphite-converted DNA was then further treated using the Infinium FFPE DNA Restore Kit before being assayed on the Illumina Infinium Human MethylationEPIC (850k) BeadChip platform, according to the Infinium HD FFPE Methylation Assay automated protocol (Illumina). DNA methylation data were also acquired for patient tumours included in the HERBY clinical trial (*n* = 7) with Illumina 450k DNA methylation arrays performed as previously reported^99^. DNA methylation array results were submitted to the molecular neuropathology methylation classifier v.12.8 hosted by the German Cancer Research Centre (DKFZ) to verify tissue primary histopathological diagnosis^100^. Individuals with a methylation class-specific calibrated score ≥0.90 were included, consistent with current clinical recommendations^100,101^.

#### DNA methylation data preprocessing

IDAT files corresponding to raw DNA methylation array data were imported using the minfi package (v.1.48.0) in R and preprocessed using the preprocessIllumina function^102^. The samples in which >10% of probes failed to hybridize were designated suboptimal and excluded from subsequent analyses. Probes mapping to sex chromosomes, within 50 bp of a single-nucleotide polymorphism, with at least one cross-reactive target, or with a minor allele frequency >5%, were also excluded^103^. Missing CpG beta values were then imputed using the inpute.knn function. CpG intensities were finally converted into methylation beta values.

#### Differential methylation analyses

Probes differentially methylated in neuroimaging-defined high- and low-connectivity groups using a median cut-off were identified using the dmpfinder function within minfi^102^. The resulting dataframe was filtered to satisfy the following cut-offs: Benjamini–Hochberg-corrected *P* < 0.05 and absolute beta value difference >0.25. Hypermethylated and hypomethylated probes in this dataset were annotated with CpG-derived gene annotations from the Illumina EPIC Manifest file (v.1.0 B5). All gene region feature categories were retained. We restricted differentially methylated CpG sites to previously defined gene sets implicated in high-grade glioma growth and assessed using our snRNA-seq dataset, namely ‘neuronal genes’, ‘DIPG synaptic genes’ and ‘invasivity genes’^3,12^. The differential expression of these gene sets was statistically compared using a two-tailed Mann–Whitney *U* test.

#### Next-generation DNA panel sequencing

For a subset of children treated at GOSH (*n* = 26), tumour DNA was screened against a curated panel of genes defined as clinically actionable or reported as recurrently altered across paediatric solid tumours^104^. Before sequencing, DNA was assayed using a Qubit 2.0 fluorometer (Thermo Fisher Scientific) and TapeStation 2200 (Agilent) to determine the quantity and degree of fragmentation. Library preparation, sequencing and variant calling were performed as described previously^104^. DNA panel sequencing data were also collected for children included in the PNOC (*n* = 38), HERBY (*n* = 12)^24^ and CHCO (*n* = 11)^105^ datasets as previously reported.

#### Chemoarchitectural dominance analyses

The chemoarchitectural profile of the DMG network was investigated by using publicly available data representing the receptor and transporter densities of 18 unique neurotransmitter receptors and transporters derived from a PET atlas of nine human neurotransmitter systems (https://github.com/netneurolab/neuromaps)^55,56,106^. These data were used to model the voxelwise brain-wide distribution of human neurotransmitter receptors and transporters and to identify which neurotransmitter receptor and transporter density data best characterized the DMG network^55,56^. To this end, the DMG network and 18 neurotransmitter receptor and transporter density maps were resampled to 3 mm voxel space and masked to include only grey matter brain regions, ensuring consistent resolution and data availability across maps. A multiple linear regression model was then fit to the neurotransmitter receptor and transporter density data to predict connectivity strength across the DMG network. The dominance of each neurotransmitter density profile to this overall model fit was then assessed by fitting this same regression model to every possible combination of input variables. The total dominance for each neurotransmitter was reported, which is defined as the relative increase in adjusted *R*^2^ when adding the same neurotransmitter data to each input variable combination, and represents the relative contribution of each neurotransmitter receptor and transporter distribution to the distribution of DMG network connectivity strength^55^. To compare the connectivity profile of short-term and long-term survivors to brainstem neuromodulatory nuclei, the mean nucleus-to-tumour connectivity values were computed and compared using a one-way ANOVA with Tukey’s post-hoc test (factor neurotransmitter system with four levels: cholinergic, serotonergic, noradrenergic and dopaminergic).

### Modelling the prognostic impact of extent of resection

A subcohort of 23 children with subtotal resections of primary thalamic DMG and brain MRI data available for analysis were identified across our study cohort. Two binary masks per patient were then manually segmented on high-resolution structural postoperative brain MRI acquired within 48 h of primary surgical resection: (1) residual tumour and (2) resection cavity. We hypothesized that surgical resections prioritizing cytoreduction and disconnection of infiltrated tumour networks would be associated with improved patient overall survival. To derive a biomarker of surgical disconnection (the disconnectome), delineated surgical paths were overlayed onto the DMG network and mean connectivity values for both the resected and non-resected masks were extracted as previously described^31,88^. Patients were stratified by their resection ratio, defined as the mean network-to-tumour connectivity of the resected tumour divided by the mean network-to-tumour connectivity of the non-resected tumour^88^, before inclusion in a Kaplan–Meier survival model. Univariable and multivariable Cox proportional hazards models in addition to statistical mediation analyses were used to examine whether the degree of connectomic resection was independent of tumour volume resected. The volume of tumour resected in each survival class was compared using a two-tailed Mann–Whitney *U* test.

### Statistical analyses

Data are reported in line with the Strengthening Reporting of Observational Studies in Epidemiology (STROBE) statement^107^. Modelling results are reported in line with the Transparent Reporting of a Multivariable Prediction Model for Individual Prognosis or Diagnosis (TRIPOD) statement^108^. Data analyses and visualization were performed using the programming languages Python (v.3.12.1; Python Software Foundation); R (v.4.3.2; R Foundation for Statistical Computing); and MATLAB (v.R2024a; MathWorks). Statistical methods were independently verified by a consulting statistician (D.R.). The normality of continuous variables was tested using histogram visualization and the Shapiro–Wilk test. Normally (Gaussian) distributed continuous variables are reported as the mean and s.d. Non-normally distributed continuous variables are reported as the median and IQR. Categoric variables are reported descriptively as percentage frequencies and compared using the *χ*^2^ or Fisher’s exact test, as appropriate. Pairwise differences in parametric data were examined using an unpaired two-tailed Student’s *t* test or one-way ANOVA with Tukey’s post-hoc test. Paired two-tailed Student’s *t *tests were performed for intraindividual pairwise comparisons. For nonparametric data, a two-tailed unpaired Mann–Whitney *U* test or one-tailed Wilcoxon signed-rank test was performed. Sample sizes are reported for all analyses. Unless otherwise stated, hypotheses were two-tailed and *P* < 0.05 was considered to be statistically significant with adjustment for multiple comparisons made where appropriate.

### Reporting summary

Further information on research design is available in the Nature Portfolio Reporting Summary linked to this article.

## Online content

Any methods, additional references, Nature Portfolio reporting summaries, source data, extended data, supplementary information, acknowledgements, peer review information; details of author contributions and competing interests; and statements of data and code availability are available at 10.1038/s41586-026-10631-3.

## Supplementary information

Supplementary Tables Supplementary Tables 1–7.

Reporting Summary

Peer Review File

## Data Availability

Lifespan HCP (development; HCP-D) and Adolescent Brain Cognitive Development (ABCD1000) study data are available from the National Institute of Mental Health Data Archive subject to appropriate permissions^35–37^. GSP1000 study data are open access, including our preprocessed distribution through the Harvard Dataverse (10.7910/DVN/ILXIKS)^40^. Yeo1000 data are subject to restricted access given study participant privacy restrictions^39^. Developmental [^18^F]FDG-PET data are available on request from the authors of the study^53^. Human neurotransmitter PET data are available through neuromaps (https://github.com/netneurolab/neuromaps)^56^. HERBY trial data are available on request from the authors of the study^57,99^. PNOC trial data are available on application (https://pnoc.us). UCSF-PDGM data are available open access through The Cancer Imaging Archive (https://www.cancerimagingarchive.net/collection/ucsf-pdgm/)^46^. Other patient datasets used in this study were curated with institutional permission for the present analyses and are not publicly available due to patient privacy and consent restrictions. Deidentified, individual-level data that can be shared are subject to institutional approvals and data transfer agreements; requests should be directed to the corresponding authors, who will respond within 10 working days. As a condition of local institutional approval, GOSH NHS patient data are not permitted to leave the GOSH environment; accordingly, these data are not available to external researchers. snRNA-seq data generated by this study have been deposited in the European Genome-Phenome Archive (https://ega-archive.org/) under controlled access (EGAD50000002514). Clinical metadata provided by BRAIN UK Participating Centres are not publicly available due to patient privacy and ethical restrictions^96^.

## Code availability

All analyses were conducted using freely available code and software using the programming languages Python (v.3.12.1; Python Software Foundation); R (v.4.3.2; R Foundation for Statistical Computing); and MATLAB (v.R2024a; MathWorks). Code used to preprocess functional connectivity data is available at GitHub (https://github.com/bchcohenlab/BIDS_to_CBIG_fMRI_Preproc2016). dMRI data were preprocessed in line with the HCP minimal processing pipelines (https://github.com/Washington-University/HCPpipelines.git). The code for lesion connectivity analyses is freely available in Lead DBS v.3.0 (www.lead-dbs.org; https://github.com/netstim/leaddbs). Voxelwise univariate VLSM was implemented in NiiStat (https://github.com/neurolabusc/NiiStat). Multivariate VLSM was implemented in SVR-LSM (https://github.com/atdemarco/svrlsmgui). The FMRIB Software Library v.6.0.7.18 (FSL; https://fsl.fmrib.ox.ac.uk/fsl/docs/#/) was also used for both resting-state fMRI and dMRI analyses, as reported in the Methods. Fluorescence-activated cell sorting was performed using FACSDiva (v.9.0; BD FACSDiva) and analysed using FlowJo (v.11.0; FlowJo), both of which are commercially available from BD Biosciences. snRNA-seq fastq files were processed with cellranger v.8.0.1, using the GRCh38 2024-A reference obtained from 10x Genomics (https://www.10xgenomics.com/support/software/cell-ranger/downloads#reference-downloads) with the chemistry flag set to ‘threeprime’ with the other arguments set as the default. The resulting filtered matrix h5 files were analysed in R using Seurat v.5.3.1. DNA methylation arrays were processed in minfi and submitted to the molecular neuropathology methylation classifier v.12.8 hosted by Heidelberg Epignostix (https://app.epignostix.com). References for all source code are provided in the Article.
