# Supplementary material for: A prognostic human brain network for diffuse midline glioma
Source: Nature. 2026 Jun 10;655(8123):769–79. doi: 10.1038/s41586-026-10631-3 (PMC13372695; doi:10.1038/s41586-026-10631-3)
Supplement: Supplementary file 1 — Supplementary Tables 1–7. [file 41586_2026_10631_MOESM1_ESM.pdf]

nature portfolio

https://doi.org/10.1038/s41586-026-10631-3

Supplementary information

A prognostic human brain network for diffuse midline glioma

In the format provided by the authors and unedited

	**Whole cohort (n=288)**	**Discovery cohort**	**External validation cohort**					***P* (discovery vs. external validation cohort)**	**PNOC trial cohort (n=38)**	***P* (discovery vs. PNOC trial cohort)**
		**GOSH (n=125)**	**Whole cohort (n=125)**	**CHCO (n=81)**	**HERBY (n=23)**	**AA (n=11)**	**USP (n=10)**			
**Age at diagnosis**, median (IQR), years	6.8 (4.9 – 11.1)	6.6 (5.2 – 9.6)	7.0 (4.8 – 11.8)	6.3 (4.4 – 11.2)	13.0 (9.9 – 14.7)	6.1 (4.0 – 7.1)	6.2 (5.4 – 7.4)	0.593	6.3 (4.8 – 9.2)	0.615
**Sex**, n (%)										
Female	158 (54.9)	71 (56.8)	70 (56.0)	44 (54.3)	13 (56.5)	6 (54.5)	7 (70.0)	1.000	17 (44.7)	0.120
Male	130 (45.1)	54 (43.2)	55 (44.0)	37 (45.7)	10 (43.5)	5 (45.5)	3 (30.0)		21 (55.3)	
**Tumour location**, n (%)										
Pons	229 (79.5)	106 (84.8)	85 (68.0)	66 (81.5)	0 (0.0)	11 (100.0)	8 (80.0)	0.003*	38 (100.0)	0.008*
Thalamus	59 (20.5)	19 (15.2)	40 (32.0)	15 (18.5)	23 (100.0)	0 (0.0)	2 (20.0)		0 (0.0)	
**Tumour volume**, median (IQR), mm^3	27635.0 (21215.0 – 37020.0)	25640.0 (20350.0 – 33340.0)	28980.0 (21980.0 – 40660.0)	27940.0 (21500.0 – 35720.0)	43820.0 (23735.0 – 68745.0)	27830.0 (23630.0 – 32390.0)	27210.0 (22597.5 – 40612.5)	0.016*	32974.5 (26040.5 – 41050.0)	0.003*
**Extent of resection**										
Gross-total	2 (0.7)	1 (0.8)	1 (0.8)	1 (1.2)	0 (0.0)	0 (0.0)	0 (0.0)	0.075	0 (0.0)	<0.001*
Subtotal	24 (8.3)	10 (8.0)	13 (10.4)	4 (4.9)	9 (39.1)	0 (0.0)	0 (0.0)		1 (2.6)	
Biopsy only	157 (54.5)	52 (41.6)	68 (54.4)	44 (54.3)	14 (60.9)	1 (9.1)	9 (90.0)		37 (97.4)	
None	105 (36.5)	62 (49.6)	43 (34.4)	32 (39.5)	0 (0.0)	10 (90.9)	1 (10.0)		0 (0.0)	
**Adjuvant radiotherapy**										
Yes	265 (92.0)	116 (92.8)	112 (89.6)	75 (92.6)	22 (95.7)	9 (81.8)	6 (60.0)	0.504	37 (97.4)	0.455
No	23 (8.0)	9 (7.2)	13 (10.4)	6 (7.4)	1 (4.3)	2 (18.2)	4 (40.0)		1 (2.6)	
**Other adjuvant therapy**										
Yes	134 (46.5)	45 (36.0)	51 (40.8)	23 (28.4)	22 (95.7)	0 (0.0)	6 (60.0)	0.516	38 (100.0)	<0.001*
No	154 (53.5)	80 (64.0)	74 (59.2)	58 (71.6)	1 (4.3)	11 (100.0)	4 (40.0)		0 (0.0)	

Supplementary Table 1. DMG patient cohort clinical characteristics and between-cohort comparisons.

GOSH: Great Ormond Street Hospital for Children NHS Foundation Trust; CHCO: Children's Hospital Colorado; HERBY: Phase II, Open-Label, Randomised, Multicentre Trial of Bevacizumab in Paediatric Patients with Newly Diagnosed High-Grade Glioma (NCT01390948); AA: Institute of Neurosurgery Dr. Alfonso Asenjo; USP: University of São Paulo; PNOC: Pediatric Neuro-Oncology Consortium; IQR: interquartile range; SD: standard deviation. Asterisks denote significant values.

	Centre of gravity coordinates			P_{FWE}	Voxels (n)	Cluster location(s)
	x	*y*	*z*			
**Cluster 1**	-2.55	-41.20	-40.60	*P*≤0.0005	6639	Brainstem, cerebellum
**Cluster 2**	-2.27	-55.70	-12.60	*P*≤0.0005	324	Cerebellar vermis
**Cluster 3**	-63.50	-0.60	2.54	*P*≤0.0005	122	Left superior temporal gyrus
**Cluster 4**	23.30	34.20	-11.00	*P*≤0.0005	102	Right orbitofrontal cortex
**Cluster 5**	33.00	-5.98	-35.60	*P*≤0.0005	93	Right hippocampus
**Cluster 6**	35.50	1.21	-15.80	*P*≤0.0005	77	Right amygdala, right insular cortex
**Cluster 7**	-36.40	36.20	8.01	*P*≤0.0005	65	Left orbitofrontal cortex
**Cluster 8**	-39.20	0.15	-14.60	*P*≤0.0005	60	Left amygdala, left insular cortex
**Cluster 9**	-19.40	-12.20	-1.02	*P*≤0.0005	75	Left pallidum
**Cluster 10**	-42.00	-18.10	39.30	*P*≤0.0005	173	Left precentral gyrus
**Cluster 11**	8.70	-24.00	5.79	*P*≤0.0005	109	Right thalamus
**Cluster 12**	18.20	-8.84	0.01	*P*≤0.0005	35	Right pallidum
**Cluster 13**	18.40	-28.50	59.70	*P*≤0.0005	21	Right precentral gyrus
**Cluster 14**	-1.03	-7.79	59.60	*P*≤0.0005	93	Right and left supplementary motor area
**Cluster 15**	6.79	-1.08	42.30	*P*≤0.0005	37	Right cingulate cortex
**Cluster 16**	-26.60	-36.40	-3.43	*P*≤0.0005	29	Left hippocampus
**Cluster 17**	-10.30	27.80	17.50	*P*≤0.0005	21	Left cingulate cortex
**Cluster 18**	-10.80	-25.20	4.26	*P*≤0.0005	20	Left thalamus

Supplementary Table 2. Significant clusters in the DMG network.

Clusters are illustrated spatially in **Main Text Fig. 1d**. Coordinates are reported in MNI space (mm) with cluster sizes in voxels (1.0 x 1.0 x 1.0mm). Several clusters included more than one neuroanatomic location, listed under cluster location(s).

	**Whole cohort (n=520)**	**UCSF-PDGM (n=248)**	**NHNN (n=272)**	***P***
**Age at diagnosis**, median (IQR), years	62.0 (54.0 – 69.7)	62.0 (54.0 – 70.0)	62.0 (53.8 – 69.6)	*P*=0.786
**Sex**, n (%)				
Female	197 (37.9)	102 (39.7)	95 (34.9)	*P*=0.149
Male	323 (62.1)	146 (56.8)	177 (65.1)	
**Tumour volume**, median (IQR), mm^3	24594.5 (10724.5 – 45888.5)	16555.0 (7558.5 – 32030.0)	30699.5 (15375.0 – 53634.0)	*P*<0.001*
**Extent of resection**				
Gross-total	196 (37.7)	152 (61.3)	44 (16.2)	*P*<0.001*
Subtotal	203 (39.0)	74 (29.8)	129 (47.4)	
Biopsy	121 (23.3)	22 (8.9)	99 (36.4)	
**MGMT promoter methylation**				
Yes	289 (55.6)	168 (67.7)	120 (44.1)	*P*<0.001*
No	221 (42.5)	70 (28.2)	152 (55.9)	
Indeterminate or not tested	10 (1.9)	10 (4.0)	0 (0.0)	
**RT + TMZ**				
Yes	457 (87.9)	248 (100.0)	209 (76.8)	*P*<0.001*
No	63 (12.1)	0 (0.0)	63 (23.2)	

Supplementary Table 3. GBM patient cohort characteristics and between-cohort comparison.

UCSF: University of California, San Francisco; NHNN: National Hospital for Neurology and Neurosurgery; IQR: interquartile range; SD: standard deviation. Asterisks denote significant values.

	Whole cohort (n=288)	Final follow-up cohort (n=71)	*P*
**Age at diagnosis**, median (IQR), years	6.8 (4.9 – 11.1)	7.5 (5.2 – 10.8)	0.473
**Sex**, n (%)			
Female	158 (54.9)	36 (50.7)	0.595
Male	130 (45.1)	35 (49.3)	
**Tumour location**, n (%)			
Pons	229 (79.5)	58 (81.7)	0.743
Thalamus	59 (20.5)	13 (18.3)	
**Tumour volume**, median (IQR), mm^3	27635.0 (21215.0 – 37020.0)	26650.0 (19465.0 – 33295.0)	0.162
**Extent of resection**			
Gross-total	2 (0.7)	1 (1.4)	0.605
Subtotal	24 (8.3)	8 (11.3)	
Biopsy only	157 (54.5)	36 (50.7)	
None	105 (36.5)	26 (36.6)	
**Adjuvant radiotherapy**			
Yes	265 (92.0)	68 (95.8)	0.441
No	23 (8.0)	3 (4.2)	
**Adjuvant chemotherapy**			
Yes	134 (46.5)	32 (45.1)	0.895
No	154 (53.5)	39 (54.9)	

Supplementary Table 4. Follow-up imaging cohort characteristics and comparison with whole study cohort.

IQR: interquartile range; SD: standard deviation.

	Label permutation test		Spin permutation test	
	ρ	P_{FDR}	ρ	P_{FDR}
**0-1 years**	-0.03	0.018	-0.13	0.436
**1-2 years**	-0.08	1.000	-0.14	0.366
**2-3 years**	-0.05	<0.001	-0.13	0.463
**3-4 years**	-0.07	1.000	-0.19	0.454
**4-5 years**	0.06	<0.001	0.12	0.461
**5-6 years***	0.20	<0.001	0.51	0.001
**6-7 years**	-0.05	<0.001	-0.15	0.402
**7-8 years**	-0.05	<0.001	-0.14	0.420
**8-9 years**	0.04	<0.001	0.16	0.221
**9-10 years**	-0.02	1.000	-0.01	0.924
**10-11 years***	0.12	<0.001	0.37	0.002
**11-12 years**	0.08	0.228	0.37	0.023
**12-13 years**	0.02	<0.001	0.01	0.980
**13-14 years**	0.04	1.000	0.13	0.258
**14-15 years**	0.02	<0.001	0.08	0.639
**15-16 years**	-0.03	<0.001	-0.08	0.440

Supplementary Table 5. Spatial comparison of age-wise changes in neurometabolic activity with DMG network topography.

Values represent label and spin permutation test results. Asterisks represent significant spatial similarity of peak, age-wise neurometabolic changes with DMG network topography. Red text indicates negative values.

	**Whole cohort (n=288)**	**DNA methylation cohort (n=36)**	***P***
**Age at diagnosis**, median (IQR), years	6.8 (4.9 – 11.1)	10.7 (6.9 – 13.7)	0.002*
**Sex**, n (%)			
Female	158 (54.9)	21 (58.3)	0.726
Male	130 (45.1)	15 (41.7)	
**Tumour location**, n (%)			
Pons	229 (79.5)	15 (41.7)	<0.001*
Thalamus	59 (20.5)	21 (58.3)	
**Tumour volume**, median (IQR), mm^3	27635.0 (21215.0 – 37020.0)	26580.0 (18825.0 – 41895.0)	1.000

Supplementary Table 6. DNA methylation cohort characteristics and comparison with whole study cohort.

IQR: interquartile range; SD: standard deviation. Asterisks denote significant values.

	**All thalamic DMG (n=59)**	**Subtotal resection cohort (n=23)**	***P***
**Age at diagnosis**, median (IQR), years	11.4 (7.3 – 14.6)	12.75 (7.5 – 14.5)	0.788
**Sex**, n (%)			
Female	32 (54.2)	12 (52.2)	0.782
Male	27 (45.8)	11 (47.8)	
**Tumour volume**, median (IQR), mm^3	37200.0 (25260.0 – 58050.0)	32440.0 (23310.0 – 39870.0)	0.128
**Extent of resection**			
Gross-total	2 (3.4)	0 (0.0)	NA (cohort defining)
Subtotal	28 (47.5)	23 (100.0)	
Biopsy only	29 (49.2)	0 (0.0)	
None	0 (0.0)	0 (0.0)	
**Adjuvant radiotherapy**			
Yes	54 (91.5)	20 (87.0)	0.680
No	5 (8.5)	3 (13.0)	
**Adjuvant chemotherapy**			
Yes	45 (76.3)	21 (91.3)	0.213
No	14 (23.7)	2 (8.7)	

Supplementary Table 7. Thalamic DMG subtotal resection cohort characteristics and comparison with all included thalamic DMG across the study cohort.

IQR: interquartile range; SD: standard deviation. Asterisks denote significant values.
