# Supplementary material for: A prognostic human brain network for diffuse midline glioma
Source: Nature. 2026 Jun 10;655(8123):769–79. doi: 10.1038/s41586-026-10631-3 (PMC13372695; doi:10.1038/s41586-026-10631-3)
Supplement: Supplementary file 2 — Reporting Summary [file 41586_2026_10631_MOESM2_ESM.pdf]

nature portfolio

Corresponding author(s): Jai Sidpra, Valentina Lind, Darren R. Hargrave

Last updated by author(s): Apr 16, 2026

Reporting Summary

Nature Portfolio wishes to improve the reproducibility of the work that we publish. This form provides structure for consistency and transparency in reporting. For further information on Nature Portfolio policies, see our Editorial Policies and the Editorial Policy Checklist.

Statistics

For all statistical analyses, confirm that the following items are present in the figure legend, table legend, main text, or Methods section.

n/a	Confirmed	
☐	☒	The exact sample size (*n*) for each experimental group/condition, given as a discrete number and unit of measurement
☐	☒	A statement on whether measurements were taken from distinct samples or whether the same sample was measured repeatedly
☐	☒	The statistical test(s) used AND whether they are one- or two-sided<br>*Only common tests should be described solely by name; describe more complex techniques in the Methods section.*
☐	☒	A description of all covariates tested
☐	☒	A description of any assumptions or corrections, such as tests of normality and adjustment for multiple comparisons
☐	☒	A full description of the statistical parameters including central tendency (e.g. means) or other basic estimates (e.g. regression coefficient) AND variation (e.g. standard deviation) or associated estimates of uncertainty (e.g. confidence intervals)
☐	☒	For null hypothesis testing, the test statistic (e.g. *F*, *t*, *r*) with confidence intervals, effect sizes, degrees of freedom and *P* value noted<br>*Give P values as exact values whenever suitable.*
☒	☐	For Bayesian analysis, information on the choice of priors and Markov chain Monte Carlo settings
☒	☐	For hierarchical and complex designs, identification of the appropriate level for tests and full reporting of outcomes
☐	☒	Estimates of effect sizes (e.g. Cohen's *d*, Pearson's *r*), indicating how they were calculated

Our web collection on statistics for biologists contains articles on many of the points above.

Software and code

Policy information about availability of computer code

Data collection	Three-dimensional (3D) volumetric patient tumour segmentations were delineated by board-certified paediatric neuroradiologists using ITK-SNAP (version 4.2.0).
Data analysis	All analyses were conducted using freely available code and software using the programming languages Python (version 3.12.1; Python Software Foundation, Wilmington, Delaware, USA); R (version 4.3.2; R Foundation for Statistical Computing, Vienna, Austria); and MATLAB (version R2024a; The MathWorks Inc., Natick, Massachusetts, USA). Code used to pre-process functional connectivity data is available in the following repository: https://github.com/bchcohenlab/BIDS_to_CBIG_fMRI_Preproc2016. dMRI data were pre-processed in line with the Human Connectome Project minimal processing pipelines (https://github.com/Washington-University/HCPpipelines.git). The code for lesion connectivity analyses is freely available in Lead DBS v3.0 (www.lead-dbs.org; https://github.com/netstim/leaddbs). Voxelwise univariate VLSM was implemented in NiiStat (https://github.com/neurolabusc/NiiStat). Multivariate VLSM was implemented in SVR-LSM (https://github.com/atdemarco/svrlsmgui). The FMRIB Software Library version 6.0.7.18 (FSL; https://fsl.fmrib.ox.ac.uk/fsl/docs/#/) was also used for both resting-state fMRI and dMRI analyses, as reported in the Methods. FACS was performed using FACSDiva™ (v9.0; BD FACSDiva) and analysed using FlowJo (v11.0; FlowJo), both of which are commercially available from BD Biosciences. snRNA-seq fastq files were processed with cellranger version8.0.1, using the GRCh38 2024-A reference obtained from 10x Genomics (https://www.10xgenomics.com/support/software/cell-ranger/downloads#reference-downloads) and chemistry flag set to "threeprime" with other arguments set as default. The resulting filtered matrix h5 files were analysed in R using Seurat v5.3.1. DNA methylation arrays were processed in minfi and submitted to the molecular neuropathology (MNP) methylation classifier v12.8 hosted by Heidelberg Epignostix (https://app.epignostix.com). References for all source code are provided in the manuscript.

For manuscripts utilizing custom algorithms or software that are central to the research but not yet described in published literature, software must be made available to editors and reviewers. We strongly encourage code deposition in a community repository (e.g. GitHub). See the Nature Portfolio guidelines for submitting code & software for further information.

Data

Policy information about availability of data

All manuscripts must include a data availability statement. This statement should provide the following information, where applicable:

- Accession codes, unique identifiers, or web links for publicly available datasets
- A description of any restrictions on data availability
- For clinical datasets or third party data, please ensure that the statement adheres to our policy

Lifespan HCP (Development; HCP-D) and Adolescent Brain Cognitive Development (ABCD1000) study data are available from the National Institute of Mental Health (NIMH) Data Archive (NDA) subject to appropriate permissions. GSP1000 study data are open access, including our pre-processed distribution via the Harvard Dataverse (https://doi.org/10.7910/DVN/ILXIKS). Yeo1000 data are subject to restricted access given study participant privacy restrictions. Developmental [18F]FDG-PET data are available upon request from Cruz-Cortes et al. Human neurotransmitter PET data are available via neuromaps (https://github.com/netneurolab/neuromaps).

HERBY trial data are available upon request from Mackay et al. and Rodriguez et al. PNOC trial data are available upon application (https://pnoc.us). UCSF-PDGM data are available open access via The Cancer Imaging Archive (https://www.cancerimagingarchive.net/collection/ucsf-pdgm/). Other patient datasets used in this study were curated with institutional permission for the present analyses and are not publicly available due to patient privacy and consent restrictions. De-identified, individual-level data that can be shared are subject to institutional approvals and data transfer agreements; requests should be directed to the corresponding authors, who will respond within 10 working days. As a condition of local institutional approval, GOSH NHS patient data are not permitted to leave the GOSH environment; accordingly, these data are not available to external researchers.

snRNA-seq data generated by this study have been deposited in the European Genome-Phenome Archive (EGA; ebi.ac.uk/ega/home) under controlled access (accession number EGAD50000002514). Clinical metadata provided by BRAIN UK Participating Centres are not publicly available due to patient privacy and ethical restrictions.

Research involving human participants, their data, or biological material

Policy information about studies with human participants or human data. See also policy information about sex, gender (identity/presentation), and sexual orientation and race, ethnicity and racism.

Reporting on sex and gender

Human subject data was collected from male and female children diagnosed with primary diffuse midline glioma. Our findings apply to both sexes. Detailed reporting of patient sex across the whole cohort and analysed subcohorts is provided in the Extended Data Tables. Patient sex was determined via self-reporting or parental reporting for infants and younger children. Sex was included as a prognostic variable alongside other clinical covariates in order to identify clinically relevant survival risk groups in multivariable analyses.

Reporting on race, ethnicity, or other socially relevant groupings

We do not report data on race, ethnicity, or other socially relevant groupings.

Population characteristics

Human subject data were collected from children (aged <18 years) diagnosed with primary diffuse midline glioma and adults (aged ≥18 years) with glioblastoma, IDH-wildtype. Detailed reporting of patient characteristics across the whole cohort and analysed subcohorts is provided in the Supplementary Information. DMG cohort summary characteristics: n=288; 54.9% female; median age at diagnosis 6.8 years (IQR 4.9-11.1 years). GBM cohort summary characteristics: n=520; 37.9% female; median age at diagnosis 62.0 years (IQR 54.0-69.7 years).

Recruitment

Three independent cohorts of children diagnosed with primary pontine or thalamic DMG were identified and analysed:
(1) A discovery cohort from Great Ormond Street Hospital for Children (GOSH), UK;
(2) An independent, multicentre external validation cohort from the Children's Hospital Colorado (CHCO), USA; University of São Paulo (USP), Brazil; Institute of Neurosurgery Dr. Alfonso Asenjo (AA), Chile; and the HERBY clinical trial (NCT01390948);
(3) A second independent, external validation cohort of children with biopsied pontine DMG, H3K27-altered, enrolled on Paediatric Neuro-Oncology Consortium (PNOC) clinical trials.

PNOC trial inclusion criteria have been previously reported. Inclusion criteria were identical across cohorts (1) and (2), with children reported between January 2000 and January 2024 inclusive:
1. Consensus clinical-radiological diagnosis of pontine DMG, defined as the rapid (<3 month) onset of cranial nerve palsies, long-tract signs, or cerebellar signs accompanied by MRI identification of an expansile, diffusely infiltrative mass arising from and involving ≥50% of the pons and which is T1-hypo/iso-intense, T2-hyperintense, and lacks or has minimal contrast-enhancement.
OR
2. Neuropathological (tissue biopsy) diagnosis of a primary pontine or thalamic DMG, H3K27-altered, in line with WHO 2021 criteria.
AND
3. Child (aged <18 years).
4. Treatment-naïve brain MRI to include a three-dimensional (3D) volumetric T1-weighted sequence with high spatial resolution and two-dimensional (2D) T2-weighted or T2-weighted fluid-attenuated inversion recovery (FLAIR) sequences, both with slice thickness ≤5mm.
5. Complete clinical data available for analysis, defined as treatment covariates and time to last follow-up or death, as appropriate.

	Neuropathological diagnosis was required for the inclusion of long-term survivors of DMG (defined a priori as an overall survival ≥18 months from diagnosis), irrespective of tumour primary pontine or thalamic location. Individuals with brain MRI of insufficient quality for analysis (e.g. partial brain coverage or severe artefact(s) due to metal or motion) were excluded. Individuals lost to follow-up within 18 months (before the threshold for long-term survivorship) were excluded due to indeterminate outcome. Inclusion was based exclusively on the availability of the above data with no exclusions based on race, ethnicity, sex, gender, or other protected characteristics. A further, independent cohort of children with biopsied pontine DMG enrolled on PNOC trials was also collected and analysed with inclusion criteria as previously reported.<br><br>Two independent cohorts of adults diagnosed with primary glioblastoma, IDH-wildtype, were also identified and analysed retrospectively:<br>1. An institutional cohort from the National Hospital for Neurology and Neurosurgery, Queen Square, London.<br>2. The open-access UCSF-PDGM cohort.
Ethics oversight	This study was approved by the institutional review board of Great Ormond Street Hospital for Children NHS Foundation Trust (GOSH; 24/HRA/4335) with informed consent waived for retrospective analyses. Analyses of human tissue were approved by BRAIN UK (25/001). Primary data collection was also approved by the institutional review boards of each participating study site prior to study commencement in accordance with the Declaration of Helsinki as amended.

Note that full information on the approval of the study protocol must also be provided in the manuscript.

Field-specific reporting

Please select the one below that is the best fit for your research. If you are not sure, read the appropriate sections before making your selection.

☒ Life sciences ☐ Behavioural & social sciences ☐ Ecological, evolutionary & environmental sciences

For a reference copy of the document with all sections, see nature.com/documents/nr-reporting-summary-flat.pdf

Life sciences study design

All studies must disclose on these points even when the disclosure is negative.

Sample size	Given the rarity of diffuse midline glioma and because there is no standard method for estimating sample size for this study type, we attempted to identify as many patients as possible, including via established international collaborations.
Data exclusions	No data were excluded from analyses.
Replication	The use of experimental replicates is not applicable to this study of human patients.
Randomization	For survival, snRNA-seq, and DNA methylation analyses, patients were stratified into low-connectivity and high-connectivity groups based on the connectivity of their tumour with the DMG network.
Blinding	Clinical and neuroimaging data were collected by independent research teams at each study site. Neuroimaging segmentations were performed blinded to patient identity and clinical outcome. Investigators were also blinded to the study group(s) being analysed.

Reporting for specific materials, systems and methods

We require information from authors about some types of materials, experimental systems and methods used in many studies. Here, indicate whether each material, system or method listed is relevant to your study. If you are not sure if a list item applies to your research, read the appropriate section before selecting a response.

Materials & experimental systems

n/a	Involved in the study	
☒	☐	Antibodies
☒	☐	Eukaryotic cell lines
☒	☐	Palaeontology and archaeology
☒	☐	Animals and other organisms
☐	☒	Clinical data
☒	☐	Dual use research of concern
☒	☐	Plants

Methods

n/a	Involved in the study	
☒	☐	ChIP-seq
☐	☒	Flow cytometry
☐	☒	MRI-based neuroimaging

Clinical data

Policy information about clinical studies

All manuscripts should comply with the ICMJE guidelines for publication of clinical research and a completed CONSORT checklist must be included with all submissions.

Clinical trial registration	N/A - this study is not a clinical trial.
Study protocol	N/A - this study is not a clinical trial.
Data collection	Three independent cohorts of children diagnosed with primary pontine or thalamic DMG were identified and analysed:<br>(1) A discovery cohort from Great Ormond Street Hospital for Children (GOSH), UK;<br>(2) An independent, multicentre external validation cohort from the Children's Hospital Colorado (CHCO), USA; University of São Paulo (USP), Brazil; Institute of Neurosurgery Dr. Alfonso Asenjo (AA), Chile; and the HERBY clinical trial (NCT01390948);<br>(3) A second independent, external validation cohort of children with biopsied pontine DMG, H3K27-altered, enrolled on Paediatric Neuro-Oncology Consortium (PNOC) clinical trials.<br>Participating centres are major international institutions with recognised subspecialty expertise in paediatric neuro-oncology. PNOC trial inclusion criteria have been previously reported. Inclusion criteria were identical across cohorts (1) and (2), with children reported between January 2000 and January 2024 inclusive.<br><br>Two independent cohorts of adults diagnosed with primary glioblastoma, IDH-wildtype, were also identified and analysed retrospectively: (1) a previously reported, open access 248 patient cohort from the University of California, San Francisco (UCSF-PDGM); (2) a newly generated institutional 272 patient cohort from the National Hospital for Neurology and Neurosurgery, Queen Square, London (patients diagnosed January 2016 - January 2024 included). Patients with midline tumours in the UCSF-PDGM cohort (n=9) were excluded as tumours were not tested for histone alterations and so a diagnosis of DMG could not be wholly excluded.
Outcomes	The primary study outcome used to define the DMG network was patient overall survival, defined as the time from diagnosis to death, and validated using survival analyses (Kaplan-Meier, univariable and multivariable cox proportional hazards models) across independent cohorts. No secondary outcomes were used to define the DMG network.

Plants

Seed stocks	N/A
Novel plant genotypes	N/A
Authentication	N/A

Flow Cytometry

Plots

Confirm that:

☒ The axis labels state the marker and fluorochrome used (e.g. CD4-FITC).

☒ The axis scales are clearly visible. Include numbers along axes only for bottom left plot of group (a 'group' is an analysis of identical markers).

☒ All plots are contour plots with outliers or pseudocolor plots.

☒ A numerical value for number of cells or percentage (with statistics) is provided.

Methodology

Sample preparation	Single-nuclei suspensions from frozen DMG patient samples were stained with DAPI (0.1µg/mL; Sigma-Aldrich D9542) immiediately prior to FACS. Intact nuclei were identified by positive staining for DAPI on a BD FACSymphony™ S6 Cell Sorter running BD FACSDiva™ v9.0 using an 355nm laser (band pass filter 450/50, long pass filter 410).
Instrument	BD FACSymphony™ S6 Cell Sorter
Software	BD FACSDiva™ v9.0 and FlowJo v11.0
Cell population abundance	We do not report data on tumour purity in this study.

Gating strategy	Nuclei were identified and debris excluded by positive staining for DAPI against forward scatter area (FSC-A). Doublets and aggregates were excluded by stringent singlet-gating based on forward scatter height (FSC-H) versus width (FSC-W) and side scatter height (SSC-H) versus width (SSC-W).

☒ Tick this box to confirm that a figure exemplifying the gating strategy is provided in the Supplementary Information.

Magnetic resonance imaging

Experimental design

Design type	Structural tumour locations defined on brain MRI combined with normative resting-state fMRI and normative dMRI.
Design specifications	Structural MRI scans were used to localise tumour locations. Resting-state fMRI data and dMRI data from each human connectome were used to compute the brain-wide connectivity of each tumour site.
Behavioral performance measures	No behavioural outcomes were assessed.

Acquisition

Imaging type(s)	DMG patient structural MRI (n=288 patients).<br>GBM patient structural MRI (n=520 patients).<br>ABCD1000, paediatric resting-state fMRI (n=1000 healthy children).<br>D-HCP, paediatric dMRI (n=497 healthy children).<br>Yeo1000, adult resting-state fMRI (n=1000 healthy adults).<br>GSP1000, adult resting-state fMRI (n=1000 healthy adults).
Field strength	All individuals in the discovery cohort underwent brain MRI on a Siemens MAGNETOM® Avanto (1.5 Tesla) or Prisma (3 Tesla) scanner. Given the retrospective multicentre nature of the study, there was significant heterogeneity in terms of scanner manufacturer and magnet field strength (1.5 or 3 Tesla) across the external validation cohort, reflective of a real-world clinical dataset.<br><br>Connectivity data (resting-state fMRI and dMRI) were acquired at 3 Tesla.
Sequence & imaging parameters	Patient structural brain MRI included at minimum a three-dimensional (3D) volumetric T1-weighted sequence with high spatial resolution and two-dimensional (2D) T2-weighted or T2-weighted fluid-attenuated inversion recovery (FLAIR) sequences, both with slice thickness ≤5mm.<br><br>Normative resting-state fMRI and dMRI connectivity data were acquired as previously reported:<br>1. ABCD1000, paediatric resting-state fMRI (Hagler et al. (Neuroimage) 2019).<br>2. D-HCP, paediatric dMRI (Somerville et al. (Neuroimage) 2018; Harms et al. (Neuroimage) 2018).<br>3. Yeo1000, adult resting-state fMRI (Yeo et al. (J Neurophysiol.) 2011).<br>4. GSP1000, adult resting-state fMRI (Holmes et al. (Sci Data) 2015).
Area of acquisition	Whole brain.

Diffusion MRI ☒ Used ☐ Not used

Parameters	As reported in Somerville et al. (Neuroimage) 2018 & Harms et al. (Neuroimage) 2018.

Preprocessing

Preprocessing software	fMRI data pre-processing was performed using the Computational Brain Imaging Group (CBIG) pre-processing pipeline (https://github.com/bchcohenlab/BIDS_to_CBIG_fMRI_Preproc2016).<br><br>dMRI data were pre-processed in line with the Human Connectome Project minimal processing pipeline (https://github.com/Washington-University/HCPpipelines.git).
Normalization	Brains were skull-stripped and extracted using SynthStrip and then registered to the Montreal Neurological Institute (MNI) MINC1 60MB paediatric template in MNI space (1.0x1.0x1.0mm) using ANTs symmetric image normalisation (SyN) registration. Cost-function masking was applied to tumour masks in order to remove them from computations. Tumours were then transformed to MNI space using forward SyN transforms and nearest neighbour interpolation.
Normalization template	Montreal Neurological Institute (MNI) MINC1 60MB paediatric template.
Noise and artifact removal	Low-pass temporal filtering, head-motion regression, global signal regression, and ventricular and white matter signal regression.
Volume censoring	Motion regression.<br>Final tumour masks were reviewed in MNI space by one or more board-certified paediatric neuroradiologist(s).

Statistical modeling & inference

Model type and settings	Tumour network mapping (details described in manuscript Methods).
Effect(s) tested	The primary effect tested was the tumour location connectivity across patients with short-term survival (defined as ≤18 months from diagnosis). This was verified by testing the difference in tumour location connectivity between patients with a priori defined short- and long-term survival.

Specify type of analysis: ☒ Whole brain ☐ ROI-based ☐ Both

Statistic type for inference<br>(See Eklund et al. 2016)	Threshold-free cluster enhancement (TFCE).<br>We also show robust results with voxelwise inference.
Correction	Family-wise error (FWE).<br>We also show robust results with false discovery rate (FDR) correction.

Models & analysis

n/a	Involved in the study
☐	☒ Functional and/or effective connectivity
☒	☐ Graph analysis
☐	☒ Multivariate modeling or predictive analysis

Functional and/or effective connectivity	Mean Pearson correlation across the normative connectivity datasets for each tumour location.
Multivariate modeling and predictive analysis	To interrogate the prognostic importance of the DMG network (as defined in the manuscript), we computed the mean network-to-tumour connectivity value for each patient. Patients were then stratified into two risk groups using a median cut-off: high risk (defined as median or above-median network-to-tumour connectivity) and low risk (defined as below-median network-to-tumour connectivity). In addition to univariate methods, we interrogated whether network-to-tumour connectivity acts as an independent predictor of patient overall survival via inclusion as a variable in multivariable cox proportional hazards models, correcting for patient demography (age at diagnosis and sex); tumour volume; and clinical management (extent of resection, and completion of adjuvant radiotherapy and chemotherapy). In all instances, the proportional hazards assumption was verified prior to analysis and results are expressed as hazard ratios with 95% confidence intervals (CI) and corresponding P values. All statistical analyses were independently reviewed by a statistician.
