# Supplementary material for: A prognostic human brain network for diffuse midline glioma
Source: Nature. 2026 Jun 10;655(8123):769–79. doi: 10.1038/s41586-026-10631-3 (PMC13372695; doi:10.1038/s41586-026-10631-3)
Supplement: Supplementary file 3 — Peer Review File [file 41586_2026_10631_MOESM3_ESM.pdf]

nature portfolio

Peer Review File

A prognostic human brain network for diffuse midline glioma

Corresponding Author: Professor Darren Hargrave

Any redactions in this file are there to maintain patient confidentiality, the confidentiality of unpublished data, or to remove third-party material.

This file contains all reviewer reports in order by version, followed by all author rebuttals in order by version.

Version 0:

Reviewer comments:

Referee #1

(Remarks to the Author)
To Authors:
A. The team here ranging from Sidpra to Hargrave present a substantial body of research regarding neuronal activity and DMG, though the data is much more compelling regarding thalamic tumors compared to pontine tumors.
B. At this point, there is a great deal of preclinical work regarding neuro-to-glioma signaling in malignant brain tumors, prominently GBM and DMG. This imaging mapping clinical interrogation is however novel and commendable to bring such a fine team of scientists together on this clinical neuroimaging investigation.
C/D. Without paediatric neuroradiologic expertise I am limited in analysis of the methodology.
E. Ultimately, an expert group of scientists with years of experience in treating this patients, analyzing their correlative data, and performing laboratory experiments have come together to assess a substantial cohort. It is of immense value to continue investigating the imaging of these patients, as it is often under-utilized in the age of targeted therapies. The major concern throughout the manuscript is that at times conclusions appear to overstate discoveries. This is evident even in the abstract as in the closing where it is written, "real-world evidence for neuronal activity modulating tumor growth in human." It would appear that the imaging data correlates with tumor presence and that resection of neuronal integrated tumor improves outcomes, however there is not clear in these human patients that neuronal activity is "modulating" tumor growth. Also the discussion references a map to a "unified brain network" - it is unclear what the definition of that is – perhaps that is a standard paediatric neuroimaging term but I am unfamiliar with it. A second major concern is that the discussion uses DMG in aggerate when the body of work shows dramatically different roles for thalamic vs. pontine tumors. It seems like the natural structure would be more appropriate that this tumor mapping is much more important for thalamic than pontine tumors. There is also repeated language of divergence from Darwinian models of evolution - however, many aggressive tumors are not clonal many of them have no neuronal integration (that is known), so I am not sure there is biologic evidence this is a break from known patterns of evolution. Statements such as "our results define a model of DMG in which tumors arise..." in the discussion presses against the boundary of what is scientifically supported in the paper. Points presented in a more detailed answerable format reside below:

F. Major--
**In the title and throughout the methods the authors refer to DMG, but in the methods its listed that “consensus clinical-radiographical…”. Does that mean that some tumors without histone status are included and considered DMG? If tumors without primary histopathologic or integrated molecular diagnosis from primary tissue is not available, those tumors would best not be included or segregated as not being diffuse midline glioma, H3K27M-altered. Also, can the authors define “consensus”?
**Figure data and relevant data comparing thalamic DMG to pontine DMG (again, are these all biopsied pontine tumors?) sees to show a relatively insignificant effect on the network in the pontine group. At best, the thalamic results are much more impressive which as a reader makes be believe there should be a much greater emphasis throughout the manuscript that this is prominent in thalamic networks, but much less important in pontine networks? I would recommend this is carried forward from the title through the manuscript, other the broad DMG classification could even inadvertently suggest equivalency of the findings in thalamus, pons, and spine when the spine is not even evaluated and the thalamic vs. pontine datasets are substantially distinct results.
**What is the overlap of diffusion imaging data for this cohort? Couldn’t the effect on the network be explained by simply more tumor cells present?

**DMG are known to be metastatic, how was the presence of metastases incorporated in the analysis? Does local extension or far distant tumor metastases associate with any findings?
**The connection of tumor initiation/growth/development to neurometabolic development is unclear. The area under the curve in Figure 4a would also include many other childhood brain tumors, for example medulloblastoma peaks between ages 4-7 but also is not dissimilar from the overall median age of all paediatric brain tumors. How is this being associated with DMG as opposed to other tumors? If tumors like medulloblastoma have overlapping incidences would it be beneficial to compare another subset of tumors hypothesized to not have this network and compare results?

Minor
**I would recommend a refrain from using “real-world”, if the goal is to distinguish from preclinical work that could be stated in other ways.
**Conflicts are listed to include FxNeuromodulation and MapLight Therapeutics, however it was unclear how they were related to this work. Were they technologies incorporated in the analysis?

G. No concerns
H. Discussed above.

Referee #2

(Remarks to the Author)
This very interesting set of analyses implicates a highly interconnected network of brain regions in poor prognosis of childhood diffuse midline glioma. The authors interpret anatomical brain tumour data from a rare patient cohort in the context of normative MRI and PET imaging data. Although the presented approach is inferential rather than causal, the converging findings support recent proposals that neural activity might play a role in growth of glial brain tumours. The results in a sub-group of operated children further suggest that removing tumour subregions highly integrated with this network may also prolong survival.

Outstanding features of the work include
1) use of an independent replication cohort and metastatic tumours of similar cell origin;
2) supplementation of the normative brain connectivity data with normative molecular and receptor density imaging data;
3) high quality and comprehensive post-hoc analyses exploring the specificity of the findings (e.g. robustness against different processing / statistical choices; verification that resting network topology is not affected by possible age differences using 9-10 year-old vs adult open datasets).

A few suggestions to help reinforce and support the proposed findings:

The paper’s aim is to characterise brain-to-tumour connectivity and its relationship with clinical prognosis. A methodological uncertainty is how accurately networks derived from normative data represent connectivity and localisation of small subcortical nuclei in individuals with anatomy-distorting pathology. Spatial displacement of fibre bundles is well characterised in adult gliomas. I am less familiar with the paediatric literature but tract displacement has been described in some childhood thalamic (Kiran et al 2013 doi.org/10.1007/s10143-013-0452-3; Kim et al 2018, doi.org/10.14791/btrt.2018.6.e14) and brainstem tumours (Helton et al 2006, PMID: 16611765 ; Xiao et al 2021, doi.org/10.3171/2020.10.FOCUS20166). Effects on connections and structures inside the thalamus could be less pronounced. However, a small case series of 5 thalamic gliomas with diffusion MRI showed marked displacement of thalamic nuclei that was not predictable from anatomical scans (Kis et al 2014, DOI: 10.1227/NEU.0000000000000333). Since registration algorithms cannot resolve boundaries at the nuclear/tract/functional level, inaccuracies in these substructures seems inevitable. It would enhance confidence in the findings if the authors could show that normative connectivity predictions do, or are likely to, hold true in midline gliomas. Perhaps there are relevant exemplars within open databases containing diffusion MRI data (e.g. https://www.cancerimagingarchive.net/collection/ucsf-pdgm/)?

Interpretationally, the data seem consistent with the idea that neural activity plays a role in glioma growth. A question is whether co-localisation offers causal evidence, and how well we can differentiate “neural activity” that is incidental vs instrumental to tumour growth? The relative sparing of the occipital and parietal lobes (Fig 1, Fig 3f) is interesting (also noted in adults), given the thalamus is proposed as a central coordinating hub. Could the preferential involvement of some, but not all, highly-connected thalamic regions alternatively point towards characteristics other than neural activity facilitating tumour growth, or effecting a worse outcome from this pattern of growth? Without the ability to selectively disrupt neural activity and show this halts tumour growth, the “real-world evidence for neural activity modulating tumour growth” (abstract) may be constrained. However, the data raise fascinating questions about what aspect of specific functional networks or network activity seem so preferred by gliomas.

Small queries:

Why was 18 months chosen to classify long-term vs short-term survival given the median survival is typically 8-11 months, and a similar median was present in these cohorts (8-9 months from Fig 2b)?

Fig 1. The sensitivity of the voxel-based tumour location mapping may be enhanced using a multivariate rather than mass univariate approach (arguments put forward by numerous authors, eg Mah et al, Brain 2014 10.1093/brain/awu164). Was there any sub-threshold effect?

In the context of shared co-localisation with the ‘DMG network’ do you have thoughts what might drive the much lower survival even in the ‘low functional connectivity’ subgroup of pontine compared to thalamic tumours (Fig 2e vs 2f)?

Could the data in relation to tumour growth patterns (Fig g) be examined statistically as a percentage / proportion of DMG network (as computed) versus the percent of non-DMG network brain affected?

Fig 5 c.d. Do the reported p-values (0.03 and 0.042) reflect correction for multiple comparisons? It would help for visual comparison to match the x-axis scales.

I found it sometimes difficult to tease out the number of participants in specific sub-group analyses. It would be helpful to state these when describing key results (examples: long-term vs short-term survivors, numbers with TP53 vs BRAF/FGFR1, how many of the 23 subtotal resection children fell into high vs low network connectivity subgroups, …). Extended Data table 1 would be useful in the primary data.

Referee #3

(Remarks to the Author)
I co-reviewed this manuscript with one of the reviewers who provided the listed reports.

Referee #4

(Remarks to the Author)

Referee #5

(Remarks to the Author)
Narrative Summary
This study evaluated the prognostic value of tumor location and tumor connectivity to distributed brain regions in patients with DMG. While overall survival was not significantly associated with lesion location while controlling for various covariates, there were significant differences in estimates of tumor connectivity between short vs. long-term survivors, which were used to define the “DMG network”. Subsequent analyses demonstrate the robustness of the DMG network and highlight its potential prognostic value as a predictor of survival outcomes in patients with DMG. Several follow-up analyses provide evidence to suggest that DMG infiltration/tumor growth preferentially occurs in locations within the DMG network, that neurometabolic changes in the DMG network occur at ages associated with peak thalamic and pontine DMG incidence, that the DMG network’s chemoarchitectural profile may make it susceptible to DMG growth, and that tumors with epigenetic neural signatures and/or stem/progenitor cell-like states tend to exhibit greater connectivity to the DMG network. The authors conclude that their results provide real-world evidence of neural activity modulating tumor growth in humans.

I think that overall, this is a relatively strong study that provides important insights into the anatomical/connectomic, metabolic, chemoarchitectonic, and epigenetic properties of DMGs and how they relate to survival outcomes and incidence rates. The study has many strengths, including the use of discovery and validation datasets and the breadth of the research questions addressed. However, there are several aspects that could be improved.

The manuscript is well-written, but it is also extremely dense in terms of analytical content, and it is written in a hyper-abbreviated style that can make the reader feel overwhelmed by the rapid-fire presentation of new and often complex analyses and results. I found that I needed to constantly jump back and forth across the main text, figures, methods, and supplemental material to achieve anything beyond the most abstract/surface-level understanding each analysis, result, and conclusion described in the main text. The figures are also very dense, and they can at times be difficult to parse and/or follow. Some of the data visualizations take up a lot of space but are not very informative and feel like they should be moved to the supplemental material (e.g. Figure 3, A-C), while others are simple-but-unintuitive and would likely be better represented using “conventional” plots (e.g. Figure 4, H-G). I think the paper would easier to follow if results were spread across more figures with fewer panels, with the goal of ensuring that the included panels feature thorough representations of the primary results. For example, Figure 1 takes up nearly a whole page to show (a) a generic representation of the analysis methods in the discovery cohort (i.e. panels A and B), and (b) 3 slices of the main functional network result, while the main the structural DMG network result is inexplicably relegated to the supplemental material.

While the methods generally seem sound, there are several key details that are very difficult to ascertain from the manuscript, and these details could have potentially substantial implications for the validity and/or interpretation of several key results. For example, it is unclear whether models were trained in the discovery dataset and applied to the validation dataset vs. trained in the validation dataset using features from the discovery dataset, and there appears to be some circularity to the modeling analyses of patients with thalamic vs. pontine DMG.

There are also several methodological choices that would seem to predispose some analyses to fail. For example, they use a continuous survival outcome for the tumor location analysis and covary multiple factors, including tumor location. Covarying for lesion location in an analysis that attempts to relate outcome to lesion location seems counterintuitive. It is not clear why the authors would use this approach for the lesion analysis and use a categorical survival outcome with no

covariates for the primary network analyses, especially since the continuous survival outcome produces much weaker results for the network analyses in the supplemental material. The "network sensitivity" analyses are also performed in a way that predisposes them to produce low correlations between the network templates in the cerebral cortex and the DMG network map that is primarily subcortical. An unthresholded brain-wide map would seem more appropriate for evaluating the target network against the Yeo networks. Several conclusions also appear to rest purely on qualitative/descriptive results, although quantitative analyses that could explicitly support the conclusions should be possible (e.g. "network sensitivity analyses", tumor growth analyses, etc.).

Based on these limitations in the current version I recommend a Major Revision with re-review.

I provide detailed comments and suggestions below.

Major Comments
1. The main focus of the paper is on elucidation of the DMG network, yet visualization of the DMG networks is generally poor. The figures give the impression that the anatomical findings lack specificity, essentially encompassing the brainstem and thalamus. It would be good to include more comprehensive visualizations of the structural and functional networks since they are the main results of the paper on which everything else rests. The authors could explore whether a higher threshold would help for display purposes in demonstrating key nodes of this network.

2. The tumor location mapping approach evaluated the association between tumor location and overall survival using voxel-wise linear regressions. However, the primary network-mapping approach compared voxel-wise tumor connectivity estimates between short- and long-term survivors of DMG. Why were different primary outcomes used for the tumor location mapping vs. network mapping analyses? Extended Data Fig. 5 indicates that when continuous overall survival was used as the outcome for network mapping, the analysis failed to define the entire network even at more liberal statistical thresholds, which the authors interpret as reflecting reduced statistical power using this outcome variable. This suggests that the failure of the tumor location mapping approach could be at least in part due to its use of the continuous overall survival outcome variable, and that the implicit comparison between tumor location vs. network approaches may be biased against tumor location by the analysis strategy. There is not an obvious reason to use a continuous version of the outcome variable and include covariates for one analysis while using a categorical version of the outcome variable with no covariates for the other. The primary tumor location mapping analyses should use the same outcome and modeling strategy as the primary network mapping analyses. To properly compare these two, I would recommend voxel-wise logistic regressions with the same covariates for analyses of both tumor locations and networks, with both analyses using the same categorical outcome variable.

3. Related to the above comment, there is an implicit comparison between the prognostic value of tumor location and tumor network information based on the presence vs. absence of significant effects in the corresponding voxel-wise analyses. However, this is not a statistically valid method for determining if one feature (i.e. network connectivity) is more prognostically valuable than another (i.e. tumor location). It would be more compelling to perform an explicit comparison of the prognostic value provided by tumor location vs. network information. This could be achieved in several ways, for example, by constructing multivariate, data-driven tumor location and network models in the discovery dataset and then directly comparing their performance at predicting survival outcomes via cross-validation and/or via application to the independent validation dataset. This would allow for principled comparisons of tumor location and network information and support explicit conclusions about the relative prognostic value of each type of tumor feature.

Related to this topic, there are clear prognostic implications of the DMG connectivity of tumor location. Analyses that may help the clinically oriented reader ascertain how robust of a predictor the connectivity values are could include R2 (predicted versus actual survival months) or accuracy for out of sample predictions binning groups into high or low survival groups.

4. The "sensitivity" analyses utilizing the 7 Yeo networks are not very compelling (Extended Data Fig. 7). The network maps were binary masks from the cortical parcellation atlas, while the network mapping results were continuous maps that (per the methods) do not appear to have been explicitly restricted to voxels within the cortical parcellation and that strongly feature subcortical/cerebellar structures. Comparing un-masked continuous statistical maps that include robust contributions from subcortical and cerebellar voxels to binary masks that are restricted to cortical parcels will artificially deflate correlations even between similar networks. It would make more sense to, for example, to use the Yeo network masks as seeds in the same rsfMRI dataset used to generate the tumor FC maps, and then compare the continuous whole-brain networks (i.e. including subcortical and cerebellar voxels) against the DMG network. Even then, though, it is unclear what a reasonable similarity threshold should be for determining whether the DMG is truly distinct from the 7 Yeo networks. Fundamentally, these analyses seem to be purely descriptive, and the rationale for how they demonstrate network sensitivity is unclear. The unthresholded, unmasked view of the DMG shown in Extended Fig.8 also suggests that the DMG spans the vast majority of the brain; is it possible that the DMG is just a union of several canonical networks? I think that these analyses should be revised, and that rationale for how they support the conclusions should be clearly stated in the manuscript.

5. There is no attempt to quantify the correspondence between the structural/functional DMGs derived from the discovery vs. validation cohorts. A spin permutation test could be used to explicitly test for statistically significant similarity between the discovery and validation cohort maps. The same could be done for the thalamic-DMG vs. pontine-DMG maps in Figure 2. Most claims about similarity (or lack thereof) in the manuscript are based on purely descriptive or qualitative comparisons.

6. Were the cox proportional hazards models trained in the discovery dataset and then applied to the validation dataset to obtain true estimates of out-of-sample prediction accuracy, or were tumor-DMG connectivity estimates in the validation

dataset used to train an inferential model in the validation dataset? This is important for interpreting results like the prediction of patient overall survival (78%, 89% with covariates) since it could still be optimistically biased (e.g. due to overfitting) if the models were trained in the validation dataset using estimates based on features identified in the discovery dataset. To make claims about prediction accuracy in the validation dataset, it would be necessary to train the models in the discovery dataset and then apply them directly to the discovery dataset, using classification cut-offs defined in the discovery dataset. Please clarify which dataset was used to train the models and define the classification cut-offs, and whether these are true out-of-sample prediction analyses vs. analyses demonstrating that models fit to features identified in the discovery dataset produce statistically significant effects when included in models trained in the validation dataset, and revise claims about prediction accuracy if necessary.

7. For the analyses aiming to ensure prognostic specificity of the DMG network, how was network-to-tumor connectivity computed?

8. The analyses of “all pontine tumors” and “all thalamic tumors” in Figure 2 appear to be circular in the sense that the thalamic-DMG and pontine-DMG networks were identified via tumor-network analyses in the subsets of patients with pontine/thalamic tumors on the basis that these networks showed significant group-level differences in FC between short- vs. long-term survivors within the subsets of patients with pontine/thalamic tumors that were used to define them. The resulting survival and modeling analyses that include mean tumor-DMG connectivity therefore appear to be circular, and the estimated effects/significance could be optimistically inflated (e.g. Vul et al., 2009). If these analyses are circular, then the Hazard Ratios and statistical significance of mean functional connectivity predictors of survival outcomes are not valid since the network used to measure mean functional connectivity was defined based on it showing a significant relationship to survival outcomes in those same patient samples. Please clarify whether these analyses are circular and adjust the reported results accordingly if necessary.

9. The analyses of patients with co-mutations in TP53 or BRAF/FGFR1 are not clearly explained in the main text or the methods. It’s also unclear how many patients were included in the analyses of the “restricted cohort of cases diagnosed only by tissue biopsy”. Based on the “modeling the prognostic impact of extent of resection” section, it appears this may be n=23, but the sample sizes should be clearly stated for each analysis within the section describing that analysis. Please clarify the methods and overarching rationale for this analysis, and please ensure that sample sizes are clearly reported for all analyses.

10. Many analyses simply state that they used “the DMG network”. However, many DMG networks were derived in this study, with two primary networks - a functional network and a structural network identified in the initial analyses of the Discovery cohort. Presumably, “the DMG network” refers to the functional network identified in the initial analyses of the Discovery cohort (e.g. based on Figures), but this should be made clear to the reader. Also, there does not appear to be any clear reason to focus on the functional network over the structural network, and I’m surprised that there are no analyses aimed at evaluating whether one approach is more prognostic than the other. Is there a strong conceptual or mechanistic reason to prioritize the functional network over the structural network? Even if there is, I think that readers (including myself) would be interested in understanding whether there are differences in prognostic value between the structural and functional networks. Multivariate analyses like the one described in Major Comment 2 could also provide insights into the relative prognostic value of the structural and functional networks.

11. The tumor infiltration analyses are interesting, but the claims about the preferential infiltration of the DMG network appear to be supported by only qualitative data (i.e. lines 354-356 and 371-372, Figure 3G, Extended Data Fig.18). I think the authors should explicitly test whether infiltration was preferential to the DMG network. This could be achieved by e.g. evaluating whether the proportion of new tumor tissue was significantly greater within vs. outside of the DMG network across patients? I think that something like this is necessary to support the stated conclusions.

12. The rationale for the cubic spline analysis of FDG-PET is unclear, and the methods do a poor job of explaining why cubic spline interpolation was considered necessary for this analysis and how it impacts the interpretation of results. How were within- vs. out-of-DMG network regions defined (e.g. any overlap with DMG network, minimum % overlap with DMG network, etc.)? How much of the brain/how many parcels were within vs. outside of the DMG network? The ROIs couldn’t be truly defined “a priori” since they depend on the results of the DMG network analyses, but regardless, the specific method for defining them should be clearly reported.

13. Figure 4D – these results also appear to be descriptive. It seems like quantitative statistical tests could be used to demonstrate that the delta SUVmax topography for certain age bins is more similar to the DMG network than the delta SUVmax for other age bins. I think that this should be done to support the conclusions.

14. A primary conclusion of the paper is that it provides real-world evidence demonstrating that neural activity modulates tumor growth in humans. However, I don’t think that that it actually does this. Instead, it shows that tumors in locations with specific network connectivity patterns are associated with worse survival outcomes and have distinct chemoarchitectural and epigenetic features than tumors in locations that don’t exhibit those connectivity patterns. It also provides evidence indicating that age-related changes in neural metabolism within the identified network coincide with ages of peak tumor incidence. While together, these observations are consistent with the conclusion that neural activity is modulating tumor growth and provide valuable insights into how various tumor characteristics relate to survival outcomes and to each other, they don’t provide any direct evidence that it is neural activity itself that is directly modulating tumor growth. I think that this conclusion should be revised to reflect the fact that none of the analyses showed (or could have shown) a direct relationship between neural activity and tumor growth.

Minor Comments

1. It’s unclear how exactly the partial correlation analysis using the 4th ventricle CSF signal was performed. Was this a temporal signal that was included in an fcMRI nuisance regression model during preprocessing of data from each healthy individual? Or was this a temporal signal that was partialled out while computing the connectivity between the tumor and the rest of the brain in the post-processed data? I lean towards it being the latter, but it should be clear from the text.

3. It’s not clear why the post-hoc mediation analyses were necessary. Couldn’t tumor volume just have been included as a covariate for each voxel-wise model when generating the DMG networks? This is a more typical approach for lesion volume control in mass-univariate analyses, so it’s not clear why the post-hoc mediation analysis was used instead. It would be helpful to explain the rationale for this choice in the methods.

4. Many if not most analyses use mean tumor-to-DMG connectivity as the network feature. However, even patients with very high peak tumor-to-DMG connectivity values could have relatively low mean tumor-to-DMG connectivity estimates if there are many weak connections within the tumor. From a conceptual/mechanistic standpoint, is there a reason that the mean tumor-DMG connectivity should be more informative than the peak tumor-to-DMG connectivity or the sum of all tumor-to-DMG connectivity values (i.e. since this would not be penalized by low values in the same way as the mean)? Is it important from a conceptual or mechanistic standpoint that there is consistently strong connectivity between the tumor and DMG?

5. The extended data figures do not appear to be ordered chronologically with respect to when they are referenced in the paper. Please order the extended data figures to match when they are referenced in the main text.

6. Figure 3F - the heatmap is very poor quality. Is it not possible to use a more comprehensive/thorough visualization? It is very difficult to evaluate support for the conclusion that “Anatomically, tumor growth mapped to the DMG network with greater tumor intersection of network than non-network brain (Fig. 3f-g)” (lines 354 to 356) based on this figure. The plot in 3G helps, but an accompanying plot that shows e.g. the proportion of tumor growth into vs. outside of the DMG network across patients would provide more quantitative support for the qualitative results in Figure 3G (see also Major Comment 10). The same is true for Extended Data Figure 18, which only shows qualitative overlap at select slices for select example patients.

7. Figure 3E – this is not a very effective way to visualize the (lack of) differences in tumor volume between high vs. low connectomic resections since the distributions and central tendencies/dispersions are somewhat difficult to ascertain for each group. Box plots (as in Extended Data Fig. 11) would be more effective.

8. Figure 3A-C takes up a lot of space but doesn’t add much. It might be better off being moved to the supplement.

9. Figure 3H – I'm not sure that showing Spearman rank correlations as linear slope is valid since Spearman rank correlations reflect the monotonicity of the relationship, and so the linear slopes may not be accurate representations of the actual relationships. Why not just show the distribution of patient-level rho estimates along with the mean and 95% CIs using a single-axis chart (e.g. stacked dots with mean/95% CIs superimposed) instead?

10. Figure 3I – it's not clear from the methods how the 16 DMG network nodes were defined. Are these parcels? Clusters? This should be clarified. I’m also not sure that this panel is really very informative. I think it would be more informative to show growth trajectories both within and outside of the DMG so that readers could evaluate the extent to which the growth trajectories are constrained to the DMG in the example patients.

11. Extended Data Figure 9 – Why does the unthresholded ABCD map have so many “empty” voxels? Shouldn’t it span the entire brain like GSP and Yeo maps? Does this reflect a data quality issue in the ABCD sample? Also, there is no quantitative comparison here. Would the thresholded GSP/Yeo maps show significant similarity to the ABCD map using e.g. spin permutation testing? Alternately, qualitative support would be stronger if the analyses were performed in all 3 datasets and the thresholded maps were shown in more detail than 3 orthogonal slices.

12. There is not sufficient context provided to frame the relevance/significance of the analyses focusing on neurotransmitter receptor and transporter densities across the DMG network. Even from an exploratory perspective, these results are interesting, but the conclusions suggest that they fit into broader knowledge about the conditions that give rise to DMG, and so it would be helpful if the authors could better explain how the observed results fit into what is known about the chemoarchitectural preferences of DMG. For example, while the Introduction mentions that DMG utilize glutamatergic/AMPA-receptor mediated and GABAergic, GABAa-receptor mediated synapses, little background is provided to suggest that findings related to cholinergic and serotonergic systems vs. dopaminergic systems would be relevant at all, much less support the conclusion that “tumor connectivity to the DMG network is not merely opportunistic but occurs within the context of an organized chemoarchitectural niche which DMG are known to exploit”. It would be good to provide context for this claim to help readers who are less familiar with the known chemoarchitectural preferences of DMG better understand the significance of the results and the conclusions drawn from them.

13. It would be helpful if Figure 4H and Figure 4I were sorted to clarify whether specific systems/nuclei cluster together in terms of their contribution to the models. In general, though, this is an odd and somewhat difficult-to-parse way of visualizing regression/ANOVA results. Simple line or bar plots with CIs/error bars, either grouped by system/nuclei or rank-ordered and colored by system/nuclei, would honestly make it easier to parse and understand these results. I would also suggest splitting

Figure 4 into 2 figures, with the FDG-PET results in Figure 4, the neurotransmitter system analyses in Figure 5, and the epigenetic analyses in Figure 6.

14. What were the factor levels in the one-way ANOVA? Neurotransmitter system? This should be clarified in the methods. The ANOVA F-test results should also be reported in addition to the post-hoc tests for completeness.

15. The methods say that sample sizes are reported for all statistical tests, but the sample sizes for specific analyses are often not unambiguously clear from the text and/or figures. Please make sure that sample sizes and relevant details about samples under analysis (e.g. whether a model was trained in one sample and applied to the other) are unambiguously clear from the main text and figures.

16. For the analyses described in line 335-337 do you have absolute tumor resection volume to use as a covariate instead of the percentage of tumor resected?

17. In line 367 – I may have missed it, but was there an analysis that directly evaluated the relationship between tumor volume and survival, or was this just done in the context of using lesion volume within network analyses? It would be surprising to me if tumor volume is not associated with survival, and I'm not sure that using it as a covariate in network analyses is a clear demonstration that there is no association of tumor volume and survival.

Version 1:

Reviewer comments:

Referee #1

(Remarks to the Author)
The extensive revision by the authors is appreciated though a critical component must be addressed first. In one of their responses they have written "We, therefore, take an inclusive approach to the inclusion of children with pontine DMG, also including those children who meet established clinical-radiological diagnostic criteria for diffuse intrinsic pontine glioma and who disease was in keeping with short-term (<18 month) overall survival."

Despite being an inclusive approach, it does not meet any consensus diagnostic criteria for diffuse midline glioma and would instead would be diffuse intrinsic pontine glioma so I would ask that data then be reclassified as DIPG throughout or only histopathologically confirmed diagnostic tissue cases are included for analysis.

Referee #2

(Remarks to the Author)
Thank you for the detailed responses and additional analyses performed. These are extensive, thoughtful and improve the robustness of the results. Methodological and analytical comments have been addressed in as far as feasible given data available to the authors and within publicly released datasets.

Ultimately all three reviewers highlighted reservations around the extent to which the data can be interpreted as evidence that neural activity is the critical factor modulating tumour growth. Acknowledging the more nuanced discussion in the revised manuscript and overall emphasis on identification of a prognostic network, a tempering limitation remains that the data can neither prove nor disprove this central mechanistic hypothesis, although the findings provide further compelling indications and avenues for research.

Referee #3

(Remarks to the Author)
I co-reviewed this manuscript with one of the reviewers who provided the listed reports.

Referee #5

(Remarks to the Author)
The authors were very comprehensive in their responses to my prior comments. My remaining questions are minor and mostly seek clarification.

- The text indicates that 5-fold cross-validation was used to evaluate the predictive utility of network-to-tumor connectivity, but states that within each fold, models were trained on 60% of the data and tested on the held-out 40%. Normally, 5-fold cross-validation would train a model on 80% of the data (4/5 folds) and test the model on the held out 20% of the data (i.e. held out fold). This seems to actually be 5 repetitions of a 60/40 hold-out cross-validation, not a five-fold cross-validation. Please clarify and/or correct this.

- How are Freedman-Lane permutations performed for a Liebermeister test? Freedman-Lane permutation tests involve residualizing the outcome with respect covariates and then permuting the residuals (e.g. Winkler et al., 2014). How can this

be accomplished with categorical outcomes while maintaining the category labels? Looking at the NiiStat code, it does not appear that covariates are included in Liebermeister test analyses or associated permutation tests (e.g. see https://github.com/neurolabusc/NiiStat/blob/master/nii_stat_core.m - functions lieber_permSub() and lieberSub(). It seems like these analyses may not actually control for lesion size based on evaluation of the code. Can you confirm whether these analyses actually controlled for lesion size, and if so, how?

- Relatedly, how are Freedman-Lane permutations performed for a Support Vector Classifier? The Freedman-Lane approach is specifically designed for the general linear model, and doesn't seem like it would work for an SVC. Indeed, the NiiStat SVM function seems to regress the lesion volume out of the lesion data, which is not consistent with Freedman-Lane (https://github.com/neurolabusc/NiiStat/blob/master/nii_stat_svm.m). Please verify that statistical methods are accurately described.

- How were multiple comparisons corrections accomplished for the VLSM and SVC-VLSM analyses? If voxel-level permutations were used, is 10,000 iterations enough (i.e. the smallest p-value possible would be 1/10001)? Voxel-wise permutation methods are known to be overly conservative (e.g. Mirman et al., 2018 - https://pubmed.ncbi.nlm.nih.gov/28847712/). Why not just use the same approach and correction method as used for the LNM analyses (i.e. PALM)?

- The description of the predictive analyses is somewhat unclear. If I understand correctly, it appears that the network was defined at the group level using the full discovery cohort, and then 5 repeat hold-out analyses (60/40 splits) were performed using one of the independent validation cohorts in which a training set partitioned from the validation cohort was used to define a median risk-cut off, which was then applied to the held-out test set also partitioned from the validation cohort before finally fitting the multivariable cox proportional hazards models in the held-out test set. For a "true" predictive approach, the entire modeling approach, from definition of the DMG network to the fitting the proportional hazards model, should ideally be performed in the training set, and then the fully parametrized model should be applied to the test set to obtain predictions that can be compared to the observed outcomes. This could be done using models that only include the clinical/demographic covariates, and these could be compared against models that also include DMG network connectivity in terms of their out-of-sample prediction performance. I can see an argument for fitting the proportional hazards model in the validation dataset, but this does result in the analyses not being a "true" predictive analysis because the validation set is being used to parametrize the proportional hazards model. The approach should be clarified in the text.

- For the analysis of tumor growth into the DMG network, a Mann-Whitney U-test was performed to compare tumor volume within vs. outside of the network. This appears to be the incorrect statistical test since these are dependent measures (i.e. for each patient, the tumor growth inside the network is being compared against the tumor growth outside of the network). A signed rank test would be more appropriate for dependent samples. This is also relevant for the analysis of the spinal DMG cohort.

- For the analysis assessing concordance of functional connectivity and tumor burden per node, the text st
ates that "the voxelwise degree of tumour overlap with each of the 18 DMG network nodes was computed for each tumour progression map, defined as the percent overlap of tumour volume relative to the volume of each network node mask". It is unclear whether this means that the overlap was computed as the percentage of the tumor that occupied each node, or as the percentage of each node that was occupied by each node. These are distinct measures, and so it should be clear which is being used.

- For the analyses referenced in the above comment, the text states that the connectivity values were distance-corrected, but it does not describe how this correction was performed. This should be clearly stated in the text.

- The authors seem to use at least 2 different parcellations (e.g. MINCI 60MB SynthSeg and Schaefer-Yeo 400 region parcellation + Brainstem Navigator). It's not clear why a single parcellation was not used. Also, does the Schaefer-Yeo + Brainstem Navigator parcellation include parcels for subcortical regions other than the brainstem?

- The Statistical Analyses section states that sample sizes are reported for all analyses, but this is not always the case (at least, the sample sizes are not always clear from the text). Please make sure that sample sizes are clear from the text, and that it is also clear which samples are used for which analyses (e.g. there are portions where it is unclear whether an analysis is performed in the discovery cohort vs. one or more of the independent validation cohorts).

- Extended Data Fig. 3 – the color bar for panel (N) seems inconsistent with the colormap.

- The network overlays shown in Extended Data Fig. 5 appear to be thresholded, but the labels say they are unthresholded.

Version 2:

Reviewer comments:

Referee #1

(Remarks to the Author)
The further defining of DIPG and DMG has improved the interpretability of this paediatric report. I remain concerned by some

answers to comments such as "We thank the reviewer for their recognition of the extensive analyses presented in this manuscript and agree that the data presented do not provide causal evidence for the hypothesis that neural activity modulates tumour growth in humans." I would prefer if this was more clear throughout the manuscript as the tone still remains too conclusive of a causal connection, despite sentences such as 'Finally, none of the experiments presented herein demonstrate nor could have demonstrated a direct relationship between tumour neural activity and tumour growth in children.' in the opinion of this reviewer.

Referee #2

(Remarks to the Author)
My comments were addressed in the previous round. The authors have added a further statement appropriately highlighting the correlative rather than causal nature of the work.

Referee #3

(Remarks to the Author)
I co-reviewed this manuscript with one of the reviewers who provided the listed reports.

Referee #5

(Remarks to the Author)
The authors have thoroughly addressed my concerns. I list some minor edits the authors may wish to incorporate.
I do not feel the need to review revisions made in response to these comments.

Minor Comments

- For the independent validation analyses using Cox proportional hazards models, it should also be stated which nonparametric CI was computed (e.g. percentile, bias-corrected and accelerated, etc.), and how the reported p-value (p=0.002) was obtained.

- The updated methods state that the SVR-LSM analysis was thresholded at a corrected p<0.005, but the updated figure included in the response shows that it was thresholded at an uncorrected p<0.005. This should be corrected in the text.

- P.7 – Line 274 – "It has been used to successfully identify causative brain networks and therapeutic targets for both canonical circuit disorders…". Lesion-network mapping is capable of identifying normative functional connections that are statistically associated with the outcome of interest, but in most if not all cases it is not sufficient to establish a causal relationship between the entire identified network and the outcome of interest. I would recommend hedging this phrasing by saying something like "…identify brain networks associated with…". These are ultimately associative analyses applied to observational data, and so I think the results should be interpreted from that perspective.

- P.8 – "Greater tumour volume was not associated with patient overall survival, (univariate cox hazard ratio 1.00; P=0.569), in keeping with previous clinical reports, and so tumour network mapping analyses were performed controlling for volume, in line with previous work.". Typically, you want to control for things that you expect to influence the outcome of interest, so the way that this is worded is somewhat strange. I think it's fine to covary for tumor size, but the wording here makes it sound like this was done specifically because there was not a significant relationship between tumor volume and survival.

- Figure 1B should probably be updated to reflect that the VLSM analyses were not completely null.

- Unless I am misunderstanding something, it seems like it would not be possible to conclude that the average of the SyN deformation field would represent the "mean tumor-associated anatomic distortion". In the registration step, the tumor itself was

excluded using cost-function masking, and many factors other than the tumor could lead the registration to require substantial deformations (e.g. head size, which would likely at least partially reflect age and sex) unless linear registrations were also used prior to performing SyN. I think that the rationale behind this should be explained in more detail.

- Methods – "Although spin permutation tests are reported to inflate P values and output more liberal statistical estimates due to preserved spatial autocorrelation between null models". My understanding is that "spatially constrained null models" (e.g. spin permutation tests) are actually more conservative and less likely to inflate p-values than spatially naïve null models, in part because they preserve spatial autocorrelation, but that they can still exhibit high false positive rates (e.g. https://doi.org/10.1016/j.neuroimage.2021.118052). It's not clear what spin permutation tests are being compared to in the quoted excerpt, but if it's spatially naïve permutation tests, then I believe the excerpt is misrepresenting the cited papers.

- Methods – " Interrogating predictive accuracy of patient overall survival by DMG network-to-tumour functional connectivity". This section still refers to the repeated 60/40 splits as "stratified five-fold cross-validation". A single five-fold cross-validation

would split the data into 5 folds, with 4 folds used for model training and the 5th fold used for model testing (i.e. an 80/20 split). What is described in the text is 5 repetitions of a 60/40 hold-out cross-validation. “Stratified cross-validation” typically means that the folds themselves are stratified according to some variable (e.g. the outcome variable) to ensure consistent representation of that variable across folds, but that does not appear to be done here. The text should be revised accordingly.

- Methods – “The voxelwise degree of tumour overlap with each of the DMG network nodes was computed for each tumour progression map, defined as the percent overlap of tumour volume relative to the volume of each network node mask”. I still find this wording confusing. Does this mean that it is defined as the proportion of each DMG network node that intersects each tumor?

- Methods – Several portions of the methods state that analyses were corrected for multiple comparisons without specifying how they were corrected (e.g. P. 30, lines 1165-1166). The specific method used to correct for multiple comparisons should be reported for each analysis.

- Figure 5 – I’m not sure that I understand why there are separate rho values provided for “whole-brain rho” vs. “spin rho”. Does this simply reflect one test using voxels and the other using parcels, and so separate spatial correlations are computed using voxels vs. parcels?

- Despite the moderate-to-high spatial correlations for maps shown in Supplemental Figures 3-5, there appear to be some potentially meaningful differences in the cortical areas represented across maps. It might be helpful to include something like frequency maps showing how often each region was identified across analyses so that the reader can better evaluate which regions are most robust to methodological variations.

Version 3:

Reviewer comments:

Referee #1

(Remarks to the Author)

Referee #3

(Remarks to the Author)
I co-reviewed this manuscript with one of the reviewers who provided the listed reports.

Referee #5

(Remarks to the Author)
The authors were able to address each of my concerns in the latest version.

Open Access This Peer Review File is licensed under a Creative Commons Attribution 4.0 International License, which permits use, sharing, adaptation, distribution and reproduction in any medium or format, as long as you give appropriate credit to the original author(s) and the source, provide a link to the Creative Commons license, and indicate if changes were made.
In cases where reviewers are anonymous, credit should be given to 'Anonymous Referee' and the source.
The images or other third party material in this Peer Review File are included in the article's Creative Commons license, unless indicated otherwise in a credit line to the material. If material is not included in the article's Creative Commons license and your intended use is not permitted by statutory regulation or exceeds the permitted use, you will need to obtain permission directly from the copyright holder.
To view a copy of this license, visit https://creativecommons.org/licenses/by/4.0/

Reviewer comments & author response:
'A prognostic human brain network for diffuse midline glioma'

Reviewer reports

Referee #1 (Remarks to the Author):

A. The team here ranging from Sidpra to Hargrave present a substantial body of research regarding neuronal activity and DMG, though the data is much more compelling regarding thalamic tumors compared to pontine tumors.

B. At this point, there is a great deal of preclinical work regarding neuro-to-glioma signaling in malignant brain tumors, prominently GBM and DMG. This imaging mapping clinical interrogation is however novel and commendable to bring such a fine team of scientists together on this clinical neuroimaging investigation.

C/D. Without paediatric neuroradiologic expertise I am limited in analysis of the methodology.

E. Ultimately, an expert group of scientists with years of experience in treating this patients, analyzing their correlative data, and performing laboratory experiments have come together to assess a substantial cohort. It is of immense value to continue investigating the imaging of these patients, as it is often under-utilized in the age of targeted therapies. The major concern throughout the manuscript is that at times conclusions appear to overstate discoveries. This is evident even in the abstract as in the closing where it is written, "real-world evidence for neuronal activity modulating tumor growth in human." It would appear that the imaging data correlates with tumor presence and that resection of neuronal integrated tumor improves outcomes, however there is not clear in these human patients that neuronal activity is "modulating" tumor growth. Also the discussion references a map to a "unified brain network" - it is unclear what the definition of that is – perhaps that is a standard paediatric neuroimaging term but I am unfamiliar with it.

A second major concern is that the discussion uses DMG in aggerate when the body of work shows dramatically different roles for thalamic vs. pontine tumors. It seems like the natural structure would be more appropriate that this tumor mapping is much more important for thalamic than pontine tumors. There is also repeated language of divergence from Darwinian models of evolution - however, many aggressive tumors are not clonal many of them have no neuronal integration (that is known), so I am not sure there is biologic evidence this is a break from known patterns of evolution. Statements such as "our results define a model of DMG in which tumors arise..." in the discussion presses against the boundary of what is scientifically supported in the paper.

Points presented in a more detailed answerable format reside below:

F. Major —

**In the title and throughout the methods the authors refer to DMG, but in the methods its listed that "consensus clinical-radiographical…". Does that mean that some tumors without histone status are included and considered DMG? If tumors without primary histopathologic or integrated molecular diagnosis from primary tissue is not available, those tumors would best not be included or segregated as not being diffuse midline glioma, H3K27M-altered. Also, can the authors define "consensus"?

**Figure data and relevant data comparing thalamic DMG to pontine DMG (again, are these all biopsied pontine tumors?) sees to show a relatively insignificant effect on the network in the pontine group. At best, the thalamic results are much more impressive which as a reader makes be believe there should be a much greater emphasis throughout the manuscript that this is prominent in thalamic networks, but much less important in pontine networks? I would recommend this is carried forward from the title through the manuscript, other the broad DMG classification could even inadvertently suggest equivalency of the findings in thalamus, pons, and spine when the spine is not even evaluated and the thalamic vs. pontine datasets are substantially distinct results.

**What is the overlap of diffusion imaging data for this cohort? Couldn't the effect on the network be explained by simply more tumor cells present?

**DMG are known to be metastatic, how was the presence of metastases incorporated in the analysis? Does local extension or far distant tumor metastases associate with any findings?

**The connection of tumor initiation/growth/development to neurometabolic development is unclear. The area under the curve in Figure 4a would also include many other childhood brain tumors, for example medulloblastoma peaks between ages 4-7 but also is not dissimilar from the overall median age of all paediatric brain tumors. How is this being associated with DMG as opposed to other tumors? If tumors like medulloblastoma have overlapping incidences would it be beneficial to compare another subset of tumors hypothesized to not have this network and compare results?

Minor

**I would recommend a refrain from using "real-world", if the goal is to distinguish from preclinical work that could be stated in other ways.

**Conflicts are listed to include FxNeuromodulation and MapLight Therapeutics, however it was unclear how they were related to this work. Were they technologies incorporated in the analysis?

G. No concerns

H. Discussed above

Referee #2 (Remarks to the Author):

This very interesting set of analyses implicates a highly interconnected network of brain regions in poor prognosis of childhood diffuse midline glioma. The authors interpret anatomical brain tumour data from a rare patient cohort in the context of normative MRI and PET imaging data. Although the presented approach is inferential rather than causal, the converging findings support recent proposals that neural activity might play a role in growth of glial brain tumours. The results in a sub-group of operated children further suggest that removing tumour subregions highly integrated with this network may also prolong survival.

Outstanding features of the work include:
1) use of an independent replication cohort and metastatic tumours of similar cell origin;
2) supplementation of the normative brain connectivity data with normative molecular and receptor density imaging data;
3) high quality and comprehensive post-hoc analyses exploring the specificity of the findings (e.g. robustness against different processing / statistical choices; verification that resting network topology is not affected by possible age differences using 9-10 year-old vs adult open datasets).

A few suggestions to help reinforce and support the proposed findings:

The paper's aim is to characterise brain-to-tumour connectivity and its relationship with clinical prognosis. A methodological uncertainty is how accurately networks derived from normative data represent connectivity and localisation of small subcortical nuclei in individuals with anatomy-distorting pathology. Spatial displacement of fibre bundles is well characterised in adult gliomas. I am less familiar with the paediatric literature but tract displacement has been described in some childhood thalamic (Kiran et al 2013; Kim et al 2018) and brainstem tumours (Helton et al 2006; Xiao et al 2021). Effects on connections and structures inside the thalamus could be less pronounced. However, a small case series of 5 thalamic gliomas with diffusion MRI showed marked displacement of thalamic nuclei that was not predictable from anatomical scans (Kis et al 2014). Since registration algorithms cannot resolve boundaries at the nuclear/tract/functional level, inaccuracies in these substructures seems inevitable. It would enhance confidence in the findings if the authors could show that normative connectivity predictions do, or are likely to, hold true in midline gliomas. Perhaps there are relevant exemplars within open databases containing diffusion MRI data (e.g. UCSF-PDGM)?

Interpretationally, the data seem consistent with the idea that neural activity plays a role in glioma growth. A question is whether co-localisation offers causal evidence, and how well we can differentiate “neural activity” that is incidental vs instrumental to tumour growth?

The relative sparing of the occipital and parietal lobes (Fig 1, Fig 3f) is interesting (also noted in adults), given the thalamus is proposed as a central coordinating hub. Could the preferential involvement of some, but not all, highly-connected thalamic regions alternatively point towards characteristics other than neural activity facilitating tumour growth, or effecting a worse outcome from this pattern of growth?

Without the ability to selectively disrupt neural activity and show this halts tumour growth, the “real-world evidence for neural activity modulating tumour growth” (abstract) may be constrained. However, the data raise fascinating questions about what aspect of specific functional networks or network activity seem so preferred by gliomas

Small queries:

Why was 18 months chosen to classify long-term vs short-term survival given the median survival is typically 8-11 months, and a similar median was present in these cohorts (8-9 months from Fig 2b)?

Fig 1. The sensitivity of the voxel-based tumour location mapping may be enhanced using a multivariate rather than mass univariate approach (arguments put forward by numerous authors, eg Mah et al, Brain 2014 10.1093/brain/awu164). Was there any sub-threshold effect?

In the context of shared co-localisation with the ‘DMG network’ do you have thoughts what might drive the much lower survival even in the ‘low functional connectivity’ subgroup of pontine compared to thalamic tumours (Fig 2e vs 2f)?

Could the data in relation to tumour growth patterns (Fig g) be examined statistically as a percentage / proportion of DMG network (as computed) versus the percent of non-DMG network brain affected?

Fig 5 c.d. Do the reported p-values (0.03 and 0.042) reflect correction for multiple comparisons? It would help for visual comparison to match the x-axis scales.

I found it sometimes difficult to tease out the number of participants in specific sub-group analyses. It would be helpful to state these when describing key results (examples: long-term vs short-term survivors, numbers with TP53 vs BRAF/FGFR1, how many of the 23 subtotal resection children fell into high vs low network connectivity subgroups, …). Extended Data table 1 would be useful in the primary data.

Referee #3 (Remarks to the Author):

I co-reviewed this manuscript with one of the reviewers who provided the listed reports.

Referee #4 (Remarks to the Author):

N/A – withdrawn.

Referee #5 (Remarks to the Author):

This study evaluated the prognostic value of tumor location and tumor connectivity to distributed brain regions in patients with DMG. While overall survival was not significantly associated with lesion location while controlling for various covariates, there were significant differences in estimates of tumor connectivity between short vs. long-term survivors, which were used to define the “DMG network”. Subsequent analyses demonstrate the robustness of the DMG network and highlight its potential prognostic value as a predictor of survival outcomes in patients with DMG. Several follow-up analyses provide evidence to suggest that DMG infiltration/tumor growth preferentially occurs in locations within the DMG network, that neurometabolic changes in the DMG network occur at ages associated with peak thalamic and pontine DMG incidence, that the DMG network’s chemoarchitectural profile may make it susceptible to DMG growth, and that tumors with epigenetic

neural signatures and/or stem/progenitor cell-like states tend to exhibit greater connectivity to the DMG network. The authors conclude that their results provide real-world evidence of neural activity modulating tumor growth in humans.

I think that overall, this is a relatively strong study that provides important insights into the anatomical/connectomic, metabolic, chemoarchitectonic, and epigenetic properties of DMGs and how they relate to survival outcomes and incidence rates. The study has many strengths, including the use of discovery and validation datasets and the breadth of the research questions addressed. However, there are several aspects that could be improved.

The manuscript is well-written, but it is also extremely dense in terms of analytical content, and it is written in a hyper-abbreviated style that can make the reader feel overwhelmed by the rapid-fire presentation of new and often complex analyses and results. I found that I needed to constantly jump back and forth across the main text, figures, methods, and supplemental material to achieve anything beyond the most abstract/surface-level understanding each analysis, result, and conclusion described in the main text. The figures are also very dense, and they can at times be difficult to parse and/or follow. Some of the data visualizations take up a lot of space but are not very informative and feel like they should be moved to the supplemental material (e.g. Figure 3, A-C), while others are simple-but-unintuitive and would likely be better represented using “conventional” plots (e.g. Figure 4, H-G). I think the paper would easier to follow if results were spread across more figures with fewer panels, with the goal of ensuring that the included panels feature thorough representations of the primary results. For example, Figure 1 takes up nearly a whole page to show (a) a generic representation of the analysis methods in the discovery cohort (i.e. panels A and B), and (b) 3 slices of the main functional network result, while the main the structural DMG network result is inexplicably relegated to the supplement.

While the methods generally seem sound, there are several key details that are very difficult to ascertain from the manuscript, and these details could have potentially substantial implications for the validity and/or interpretation of several key results. For example, it is unclear whether models were trained in the discovery dataset and applied to the validation dataset vs. trained in the validation dataset using features from the discovery dataset, and there appears to be some circularity to the modeling analyses of patients with thalamic vs. pontine DMG.

There are also several methodological choices that would seem to predispose some analyses to fail. For example, they use a continuous survival outcome for the tumor location analysis and covary multiple factors, including tumor location. Covarying for lesion location in an analysis that attempts to relate outcome to lesion location seems counterintuitive. It is not clear why the authors would use this approach for the lesion analysis and use a categorical survival outcome with no covariates for the primary network analyses, especially since the continuous survival outcome produces much weaker results for the network analyses in the supplemental material. The “network sensitivity” analyses are also performed in a way that predisposes them to produce low correlations between the network templates in the cerebral cortex and the DMG network map that is primarily subcortical. An unthresholded brain-wide map would seem more appropriate for evaluating the target network against the Yeo networks. Several conclusions also appear to rest purely on qualitative/descriptive results, although quantitative analyses that could explicitly support the conclusions should be possible (e.g. “network sensitivity analyses”, tumor growth analyses, etc.).

Based on these limitations in the current version I recommend a Major Revision with re-review.

I provide detailed comments and suggestions below.

Major Comments

1. The main focus of the paper is on elucidation of the DMG network, yet visualization of the DMG networks is generally poor. The figures give the impression that the anatomical findings lack specificity, essentially encompassing the brainstem and thalamus. It would be good to include more comprehensive visualizations of the structural and functional networks since they are the main results of the paper on which everything else rests. The authors could explore whether a higher threshold would help for display purposes in demonstrating key nodes of this network.

2. The tumor location mapping approach evaluated the association between tumor location and overall survival using voxel-wise linear regressions. However, the primary network-mapping approach compared voxel-wise tumor connectivity estimates between short- and long-term survivors of DMG. Why were different primary outcomes used for the tumor location mapping vs. network mapping analyses? Extended Data Fig. 5 indicates that when continuous overall survival was used as the outcome for network mapping, the analysis failed to define the entire network even at more liberal statistical thresholds, which the authors interpret as reflecting reduced statistical power using this outcome variable. This suggests that the failure of the tumor location mapping approach could be at least in part due to its use of the continuous overall survival outcome variable, and that the implicit comparison between tumor location vs. network approaches may be biased against tumor location by the analysis strategy. There is not an obvious reason to use a continuous version of the outcome variable and include covariates for one analysis while using a categorical version of the outcome variable with no covariates for the other. The primary tumor location mapping analyses should use the same outcome and modeling strategy as the primary network mapping analyses. To properly compare these two, I would recommend voxel-wise logistic regressions with the same covariates for analyses of both tumor locations and networks, with both analyses using the same categorical outcome variable.

3. Related to the above comment, there is an implicit comparison between the prognostic value of tumor location and tumor network information based on the presence vs. absence of significant effects in the corresponding voxel-wise analyses. However, this is not a statistically valid method for determining if one feature (i.e. network connectivity) is more prognostically valuable than another (i.e. tumor location). It would be more compelling to perform an explicit comparison of the prognostic value provided by tumor location vs. network information. This could be achieved in several ways, for example, by constructing multivariate, data-driven tumor location and network models in the discovery dataset and then directly comparing their performance at predicting survival outcomes via cross-validation and/or via application to the independent validation dataset. This would allow for principled comparisons of tumor location and network information and support explicit conclusions about the relative prognostic value of each type of tumor feature. Related to this topic, there are clear prognostic implications of the DMG connectivity of tumor location. Analyses that may help the clinically oriented reader ascertain how robust of a predictor the connectivity values are could include R2 (predicted versus actual survival months) or accuracy for out of sample predictions binning groups into high or low survival groups.

4. The “sensitivity” analyses utilizing the 7 Yeo networks are not very compelling (Extended Data Fig. 7). The network maps were binary masks from the cortical parcellation atlas, while the network mapping results were continuous maps that (per the methods) do not appear to have been explicitly restricted to voxels within the cortical parcellation and that strongly feature subcortical/cerebellar structures. Comparing un-masked continuous statistical maps that include robust contributions from subcortical and cerebellar voxels to binary masks that are restricted to cortical parcels will artificially deflate correlations even between similar networks. It would make more sense to, for example, to use the Yeo network masks as seeds in the same rsfMRI dataset used to generate the tumor FC maps, and then compare the continuous whole-brain networks (i.e. including subcortical and cerebellar voxels) against the DMG network. Even then, though, it is unclear what a reasonable similarity threshold should be for determining whether the DMG is truly distinct from the 7 Yeo networks. Fundamentally, these analyses seem to be purely descriptive, and the rationale for how they demonstrate network sensitivity is unclear. The unthresholded, unmasked view of the DMG shown in Extended Fig.8 also suggests that the DMG spans the vast majority of the brain; is it possible that the DMG is just a union of several canonical networks? I think that these analyses should be revised, and that rationale for how they support the conclusions should be clearly stated in the manuscript.

5. There is no attempt to quantify the correspondence between the structural/functional DMGs derived from the discovery vs. validation cohorts. A spin permutation test could be used to explicitly test for statistically significant similarity between the discovery and validation cohort maps. The same could be done for the thalamic-DMG vs. pontine-DMG maps in Figure 2. Most claims about similarity (or lack thereof) in the manuscript are based on purely descriptive or qualitative comparisons.

6. Were the cox proportional hazards models trained in the discovery dataset and then applied to the validation dataset to obtain true estimates of out-of-sample prediction accuracy, or were tumor-DMG connectivity estimates in the validation dataset used to train an inferential model in the validation

dataset? This is important for interpreting results like the prediction of patient overall survival (78%, 89% with covariates) since it could still be optimistically biased (e.g. due to overfitting) if the models were trained in the validation dataset using estimates based on features identified in the discovery dataset. To make claims about prediction accuracy in the validation dataset, it would be necessary to train the models in the discovery dataset and then apply them directly to the discovery dataset, using classification cut-offs defined in the discovery dataset. Please clarify which dataset was used to train the models and define the classification cut-offs, and whether these are true out-of-sample prediction analyses vs. analyses demonstrating that models fit to features identified in the discovery dataset produce statistically significant effects when included in models trained in the validation dataset, and revise claims about prediction accuracy if necessary.

7. For the analyses aiming to ensure prognostic specificity of the DMG network, how was network-to-tumor connectivity computed?

8. The analyses of “all pontine tumors” and “all thalamic tumors” in Figure 2 appear to be circular in the sense that the thalamic-DMG and pontine-DMG networks were identified via tumor-network analyses in the subsets of patients with pontine/thalamic tumors on the basis that these networks showed significant group-level differences in FC between short- vs. long-term survivors within the subsets of patients with pontine/thalamic tumors that were used to define them. The resulting survival and modeling analyses that include mean tumor-DMG connectivity therefore appear to be circular, and the estimated effects/significance could be optimistically inflated (e.g. Vul et al., 2009). If these analyses are circular, then the Hazard Ratios and statistical significance of mean functional connectivity predictors of survival outcomes are not valid since the network used to measure mean functional connectivity was defined based on it showing a significant relationship to survival outcomes in those same patient samples. Please clarify whether these analyses are circular and adjust the reported results accordingly if necessary.

9. The analyses of patients with co-mutations in TP53 or BRAF/FGFR1 are not clearly explained in the main text or the methods. It’s also unclear how many patients were included in the analyses of the “restricted cohort of cases diagnosed only by tissue biopsy”. Based on the “modeling the prognostic impact of extent of resection” section, it appears this may be n=23, but the sample sizes should be clearly stated for each analysis within the section describing that analysis. Please clarify the methods and overarching rationale for this analysis, and please ensure that sample sizes are clearly reported for all analyses.

10. Many analyses simply state that they used “the DMG network”. However, many DMG networks were derived in this study, with two primary networks - a functional network and a structural network identified in the initial analyses of the Discovery cohort. Presumably, “the DMG network” refers to the functional network identified in the initial analyses of the Discovery cohort (e.g. based on Figures), but this should be made clear to the reader. Also, there does not appear to be any clear reason to focus on the functional network over the structural network, and I’m surprised that there are no analyses aimed at evaluating whether one approach is more prognostic than the other. Is there a strong conceptual or mechanistic reason to prioritize the functional network over the structural network? Even if there is, I think that readers (including myself) would be interested in understanding whether there are differences in prognostic value between the structural and functional networks. Multivariate analyses like the one described in Major Comment 2 could also provide insights into the relative prognostic value of the structural and functional networks.

11. The tumor infiltration analyses are interesting, but the claims about the preferential infiltration of the DMG network appear to be supported by only qualitative data (i.e. lines 354-356 and 371-372, Figure 3G, Extended Data Fig.18). I think the authors should explicitly test whether infiltration was preferential to the DMG network. This could be achieved by e.g. evaluating whether the proportion of new tumor tissue was significantly greater within vs. outside of the DMG network across patients? I think that something like this is necessary to support the stated conclusions.

12. The rationale for the cubic spline analysis of FDG-PET is unclear, and the methods do a poor job of explaining why cubic spline interpolation was considered necessary for this analysis and how it impacts the interpretation of results. How were within- vs. out-of-DMG network regions defined (e.g. any overlap with DMG network, minimum % overlap with DMG network, etc.)? How much of the brain/how many parcels were within vs. outside of the DMG network? The ROIs couldn’t be truly

defined “a priori” since they depend on the results of the DMG network analyses, but regardless, the specific method for defining them should be clearly reported.

13. Figure 4D – these results also appear to be descriptive. It seems like quantitative statistical tests could be used to demonstrate that the delta SUVmax topography for certain age bins is more similar to the DMG network than the delta SUVmax for other age bins. I think that this should be done to support the conclusions.

14. A primary conclusion of the paper is that it provides real-world evidence demonstrating that neural activity modulates tumor growth in humans. However, I don’t think that that it actually does this. Instead, it shows that tumors in locations with specific network connectivity patterns are associated with worse survival outcomes and have distinct chemoarchitectural and epigenetic features than tumors in locations that don’t exhibit those connectivity patterns. It also provides evidence indicating that age-related changes in neural metabolism within the identified network coincide with ages of peak tumor incidence. While together, these observations are consistent with the conclusion that neural activity is modulating tumor growth and provide valuable insights into how various tumor characteristics relate to survival outcomes and to each other, they don’t provide any direct evidence that it is neural activity itself that is directly modulating tumor growth. I think that this conclusion should be revised to reflect the fact that none of the analyses showed (or could have shown) a direct relationship between neural activity and tumor growth.

Minor Comments

1. It’s unclear how exactly the partial correlation analysis using the 4th ventricle CSF signal was performed. Was this a temporal signal that was included in an fcMRI nuisance regression model during preprocessing of data from each healthy individual? Or was this a temporal signal that was partialled out while computing the connectivity between the tumor and the rest of the brain in the post-processed data? I lean towards it being the latter, but it should be clear from the text.

3. It’s not clear why the post-hoc mediation analyses were necessary. Couldn’t tumor volume just have been included as a covariate for each voxel-wise model when generating the DMG networks? This is a more typical approach for lesion volume control in mass-univariate analyses, so it’s not clear why the post-hoc mediation analysis was used instead. It would be helpful to explain the rationale for this choice in the methods.

4. Many if not most analyses use mean tumor-to-DMG connectivity as the network feature. However, even patients with very high peak tumor-to-DMG connectivity values could have relatively low mean tumor-to-DMG connectivity estimates if there are many weak connections within the tumor. From a conceptual/mechanistic standpoint, is there a reason that the mean tumor-DMG connectivity should be more informative than the peak tumor-to-DMG connectivity or the sum of all tumor-to-DMG connectivity values (i.e. since this would not be penalized by low values in the same way as the mean)? Is it important from a conceptual or mechanistic standpoint that there is consistently strong connectivity between the tumor and DMG?

5. The extended data figures do not appear to be ordered chronologically with respect to when they are referenced in the paper. Please order the extended data figures to match when they are referenced in the main text.

6. Figure 3F - the heatmap is very poor quality. Is it not possible to use a more comprehensive/thorough visualization? It is very difficult to evaluate support for the conclusion that “Anatomically, tumor growth mapped to the DMG network with greater tumor intersection of network than non-network brain (Fig. 3f-g)” (lines 354 to 356) based on this figure. The plot in 3G helps, but an accompanying plot that shows e.g. the proportion of tumor growth into vs. outside of the DMG network across patients would provide more quantitative support for the qualitative results in Figure 3G (see also Major Comment 10). The same is true for Extended Data Figure 18, which only shows qualitative overlap at select slices for select example patients.

7. Figure 3E – this is not a very effective way to visualize the (lack of) differences in tumor volume between high vs. low connectomic resections since the distributions and central tendencies/dispersions are somewhat difficult to ascertain for each group. Box plots (as in Extended

Data Fig. 11) would be more effective.

8. Figure 3A-C takes up a lot of space but doesn't add much. It might be better off being moved to the supplement.

9. Figure 3H – I'm not sure that showing Spearman rank correlations as linear slope is valid since Spearman rank correlations reflect the monotonicity of the relationship, and so the linear slopes may not be accurate representations of the actual relationships. Why not just show the distribution of patient-level rho estimates along with the mean and 95% CIs using a single-axis chart (e.g. stacked dots with mean/95% CIs superimposed) instead?

10. Figure 3I – it's not clear from the methods how the 16 DMG network nodes were defined. Are these parcels? Clusters? This should be clarified. I'm also not sure that this panel is really very informative. I think it would be more informative to show growth trajectories both within and outside of the DMG so that readers could evaluate the extent to which the growth trajectories are constrained to the DMG in the example patients.

11. Extended Data Figure 9 – Why does the unthresholded ABCD map have so many "empty" voxels? Shouldn't it span the entire brain like GSP and Yeo maps? Does this reflect a data quality issue in the ABCD sample? Also, there is no quantitative comparison here. Would the thresholded GSP/Yeo maps show significant similarity to the ABCD map using e.g. spin permutation testing? Alternately, qualitative support would be stronger if the analyses were performed in all 3 datasets and the thresholded maps were shown in more detail than 3 orthogonal slices.

12. There is not sufficient context provided to frame the relevance/significance of the analyses focusing on neurotransmitter receptor and transporter densities across the DMG network. Even from an exploratory perspective, these results are interesting, but the conclusions suggest that they fit into broader knowledge about the conditions that give rise to DMG, and so it would be helpful if the authors could better explain how the observed results fit into what is known about the chemoarchitectural preferences of DMG. For example, while the Introduction mentions that DMG utilize glutamatergic/AMPA-receptor mediated and GABAergic, GABAa-receptor mediated synapses, little background is provided to suggest that findings related to cholinergic and serotonergic systems vs. dopaminergic systems would be relevant at all, much less support the conclusion that "tumor connectivity to the DMG network is not merely opportunistic but occurs within the context of an organized chemoarchitectural niche which DMG are known to exploit". It would be good to provide context for this claim to help readers who are less familiar with the known chemoarchitectural preferences of DMG better understand the significance of the results and the conclusions drawn from them.

13. It would be helpful if Figure 4H and Figure 4I were sorted to clarify whether specific systems/nuclei cluster together in terms of their contribution to the models. In general, though, this is an odd and somewhat difficult-to-parse way of visualizing regression/ANOVA results. Simple line or bar plots with CIs/error bars, either grouped by system/nuclei or rank-ordered and colored by system/nuclei, would honestly make it easier to parse and understand these results. I would also suggest splitting Figure 4 into 2 figures, with the FDG-PET results in Figure 4, the neurotransmitter system analyses in Figure 5, and the epigenetic analyses in Figure 6.

14. What were the factor levels in the one-way ANOVA? Neurotransmitter system? This should be clarified in the methods. The ANOVA F-test results should also be reported in addition to the post-hoc tests for completeness.

15. The methods say that sample sizes are reported for all statistical tests, but the sample sizes for specific analyses are often not unambiguously clear from the text and/or figures. Please make sure that sample sizes and relevant details about samples under analysis (e.g. whether a model was trained in one sample and applied to the other) are unambiguously clear from the main text and figures.

16. For the analyses described in line 335-337 do you have absolute tumor resection volume to use as a covariate instead of the percentage of tumor resected?

17. In line 367 – I may have missed it, but was there an analysis that directly evaluated the relationship between tumor volume and survival, or was this just done in the context of using lesion volume within network analyses? It would be surprising to me if tumor volume is not associated with survival, and I'm not sure that using it as a covariate in network analyses is a clear demonstration that there is no association of tumor volume and survival.

Author response to reviewer comments

Thank you for the opportunity to revise our manuscript, entitled: 'A prognostic human brain network for diffuse midline glioma' (DMG). We were delighted to see the enthusiastic comments and thorough, thoughtful suggestions. We have now addressed each comment with further analyses and clarifying edits to the manuscript and/or figures. To speak to specific questions, we have also included new patient cohorts and conducted a single-nucleus RNA sequencing (snRNA-seq) experiment to elucidate tissue-level differences between high- and low-connectivity DMG in primary patient surgical samples. These changes have significantly improved the manuscript, and we are grateful for your constructive input. New findings, together with additional control and verification experiments, are detailed in the point-by-point response below. Throughout, textual changes are emphasised in **bold font**.

Referee #1 (Remarks to the Author):

A. The team here ranging from Sidpra to Hargrave present a substantial body of research regarding neuronal activity and DMG, though the data is much more compelling regarding thalamic tumors compared to pontine tumors.

B. At this point, there is a great deal of preclinical work regarding neuro-to-glioma signaling in malignant brain tumors, prominently GBM and DMG. This imaging mapping clinical interrogation is however novel and commendable to bring such a fine team of scientists together on this clinical neuroimaging investigation.

C/D. Without paediatric neuroradiologic expertise I am limited in analysis of the methodology.

E. Ultimately, an expert group of scientists with years of experience in treating this patients, analyzing their correlative data, and performing laboratory experiments have come together to assess a substantial cohort. It is of immense value to continue investigating the imaging of these patients, as it is often under-utilized in the age of targeted therapies.

The major concern throughout the manuscript is that at times conclusions appear to overstate discoveries. This is evident even in the abstract as in the closing where it is written, "real-world evidence for neuronal activity modulating tumor growth in human." It would appear that the imaging data correlates with tumor presence and that resection of neuronal integrated tumor improves outcomes, however there is not clear in these human patients that neuronal activity is "modulating" tumor growth.

Also the discussion references a map to a "unified brain network" - it is unclear what the definition of that is – perhaps that is a standard paediatric neuroimaging term but I am unfamiliar with it.

A second major concern is that the discussion uses DMG in aggerate when the body of work shows dramatically different roles for thalamic vs. pontine tumors. It seems like the natural structure would be more appropriate that this tumor mapping is much more important for thalamic than pontine tumors.

There is also repeated language of divergence from Darwinian models of evolution - however, many aggressive tumors are not clonal many of them have no neuronal integration (that is known), so I am not sure there is biologic evidence this is a break from known patterns of evolution. Statements such as "our results define a model of DMG in which tumors arise..." in the discussion presses against the boundary of what is scientifically supported in the paper.

Thank you for your positive receipt of our work and for your constructive suggestions on how we may improve the manuscript. All points raised are important considerations which we believe have notably improved the quality and clarity of the analyses presented. To this end, we wish to first highlight three responses to points which are not later discussed in the Major or Minor comments, as below:

1. **The major concern throughout the manuscript is that at times conclusions appear to overstate discoveries. This is evident even in the abstract as in the closing where it is written, "real-world evidence for neuronal activity modulating tumor growth in human." It would appear that the imaging data correlates with tumor presence and that resection of neuronal integrated tumor improves outcomes, however there is not clear in these human patients that neuronal activity is "modulating" tumor growth.**

Thank you for raising this. We have comprehensively rewritten the manuscript to ensure that all conclusions are directly supported by our data. In particular, and as raised by other reviewers, whilst we do not directly measure or manipulate neural activity in relation to tumour growth in children with DMG, nor could we have aimed to do so, we infer neural activity by means of tumour functional connections defined by a normative paediatric connectome in relation to both patient overall survival and the anatomic pattern of tumour growth. We have, therefore, clarified the manuscript main text throughout and have added the following explicit acknowledgement of this limitation to the discussion:

"Further, whilst the role of neuromodulatory neurotransmitters in DMG growth, in particular combinatorial cholinergic, serotonergic, and noradrenergic signalling, remains to be fully described, preclinical reports have revealed the robust effects of brainstem cholinergic neurons and muscarinic signalling on DMG growth and invasion, with further emerging work indicating similar mechanistic importance for serotonergic and noradrenergic signalling.[5,24,25] ***Finally, none of the experiments presented herein demonstrate nor could have demonstrated a direct relationship between tumour neural activity and tumour growth in children. Within the framework of causal human brain mapping[42], however, the convergent data presented across multiple modalities are consistent with the hypothesis that DMG network connectivity is associated with DMG tumour growth and patient overall survival.****"*

2. **Also the discussion references a map to a "unified brain network" - it is unclear what the definition of that is – perhaps that is a standard paediatric neuroimaging term but I am unfamiliar with it.**

Thank you for highlighting this ambiguity. We take 'unified' to mean that the pattern of prognostic brain network connections in children with pontine DMG and thalamic DMG is conserved – i.e. topographically significantly similar. We show this in two main ways, as fully discussed in the Major comments. To speak directly to this specific comment, we have revised the word 'unified' in favour of the word 'conserved' as we believe this to be a more intuitive descriptor.

3. **There is also repeated language of divergence from Darwinian models of evolution - however, many aggressive tumors are not clonal many of them have no neuronal integration (that is known), so I am not sure there is biologic evidence this is a break from known patterns of evolution. Statements such as "our results define a model of DMG in which tumors arise..." in the discussion presses against the boundary of what is scientifically supported in the paper.**

We thank the reviewer for this important comment. We did not mean to say that our data show divergence from known patterns of evolution. Previous work has shown that the progression of DMG, in particular those tumours which metastasise beyond the primary tumour site, can only be partially explained by clonal evolution (Vinci … Jones. *Nat Med*. 2018.; Nikbakht … Nazarian. *Nat Commun*. 2016.). Our data build on these data to suggest that neural activity may at least partially account for this discrepancy. In response to the reviewer's insightful point, we have now removed this statement from the text in favour of the following:

"The tropism of tumour growth for connectivity peaks in the DMG network further reflects reported, neural activity-dependent directional tumour infiltration described in pre-clinical models of high-grade glioma[9,12,13] and offers an explanation to questions raised by whole-brain

human post-mortem pontine DMG analyses which define a conserved pattern of tumour anatomic progression insufficiently explained by current models of tumour evolution.[20,21]"

Points presented in a more detailed answerable format reside below:

F. Major —

****In the title and throughout the methods the authors refer to DMG, but in the methods its listed that "consensus clinical-radiographical…". Does that mean that some tumors without histone status are included and considered DMG? If tumors without primary histopathologic or integrated molecular diagnosis from primary tissue is not available, those tumors would best not be included or segregated as not being diffuse midline glioma, H3K27M-altered. Also, can the authors define "consensus"?**

We thank the reviewer for raising this important point. In this study, we include children with either biopsy-confirmed pontine or thalamic DMG, as per World Health Organisation (WHO) 2021 criteria (Louis … Ellison. *Neuro Oncol*. 2021.), whilst also recognising the fact that, historically, biopsies of pontine tumours were not performed (Albright … Hammond. *Neurosurgery*. 1993.) – a fact which we believe the current WHO classification does not acknowledge. We, therefore, take an inclusive approach to the inclusion of children with pontine DMG, also including those children who meet established clinical-radiological diagnostic criteria for diffuse intrinsic pontine glioma (DIPG) and whose disease course was in keeping with short-term (<18 month) overall survival. Specifically, we adopted the following criteria for the clinical-radiological diagnosis of DIPG: the rapid (<3 month) onset of cranial nerve palsies, long-tract signs, or cerebellar signs accompanied by MRI identification of an expansile, diffusely infiltrative mass arising from and involving ≥50% of the pons and which is T1-hypo/iso-intense, T2-hyperintense, and lacks or has minimal contrast-enhancement (Albright … Rourke. *J Neurosurg*. 1986.; Donaldson … Fisher. *J Clin Oncol*. 2006.; Lazow … Leach. *Neuro Oncol*. 2022.). We take 'consensus' to reflect the presence of both clinical and radiographic hallmarks of the disease. Tissue diagnosis was required for the inclusion of all thalamic DMG and all long-term (≥18 months) survivors of pontine DMG.

The neuroimaging of all included patients was reviewed by a senior board-certified paediatric neuroradiologist with >15 years post-qualification experience. Importantly, in a series of 77 children with paired histopathological and neuroimaging assessment from the International DIPG / DMG registry, neuroimaging diagnosis was >95% concordant with histopathological diagnosis when using these strict diagnostic criteria – including when some unusual features were included, which our study excluded, e.g. intratumoral haemorrhage and necrosis (Lazow … Leach. *Neuro Oncol*. 2022.). A similar audit at our institution demonstrated 98% radiologic diagnostic accuracy when compared to pathology ground-truth data for 43 children with biopsy-confirmed pontine DMG treated over the past 8 years (unpublished data; retrospective case note review January 2018 – January 2025). We are, therefore, confident that it would be unlikely for a child with an alternate diagnosis to be included, particularly given both radiologically misdiagnosed children in our review had >18 month overall survival. However, we acknowledge and agree that the inclusion of children with non-biopsied lesions warrants further consideration and, to speak to these concerns, we have implemented several changes:

1. We have clarified our inclusion criteria in the main text and methods (as below with revised text highlighted in bold for ease of reference throughout). Further, throughout the manuscript, we take care to only refer to DMG as 'H3K27-altered' when discussing subgroup analyses of children with biopsy-confirmed diagnoses.

    Main text introduction

    *"**Diffuse intrinsic pontine glioma (DIPG) and other diffuse midline gliomas (DMG)** are the leading cause of solid tumour-related death in children which, despite international clinical trials over the past two decades, retain notorious therapeutic resistance and universal lethality.[2,17,18]"*

Main text results

*"We first studied a discovery cohort of 125 children aged <18 years with primary pontine (n=106) or thalamic (n=19) DMG treated at Great Ormond Street Hospital for Children (GOSH), a large international paediatric neuro-oncology referral centre (**Fig. 1a; Supplementary Table 1**). **As pontine biopsies were historically not performed[28], children meeting clinical diagnostic criteria for DIPG[29–31] and with a disease course in keeping with short-term (<18 month)[32] overall survival were included. Throughout, we adopt this inclusive definition of DMG whilst also demonstrating reproducibility of findings across a restricted cohort of children with biopsied pontine DMG, H3K27-altered, in keeping with the World Health Organisation (WHO) 2021 definition of the disease.[1]**"*

Methods

"Inclusion criteria were identical across cohorts:

1. ***Consensus clinical-radiological diagnosis of pontine DMG, defined as the rapid (<3 month) onset of cranial nerve palsies, long-tract signs, or cerebellar signs accompanied by MRI identification of an expansile, diffusely infiltrative mass arising from and involving ≥50% of the pons and which is T1-hypo/iso-intense, T2-hyperintense, and lacks or has minimal contrast-enhancement.[29–31]***
   OR
2. *Neuropathological (tissue biopsy) diagnosis of a primary pontine or thalamic DMG, **H3K27-altered**, in line with WHO 2021 criteria.[1]*
   AND
3. *Child (aged <18 years).*
4. *Treatment-naïve brain MRI to include a three-dimensional (3D) volumetric T1-weighted sequence with high spatial resolution and two-dimensional (2D) T2-weighted or T2-weighted fluid-attenuated inversion recovery (FLAIR) sequences, both with slice thickness ≤5mm.*
5. *Complete clinical data available for analysis, defined as treatment covariates and time to last follow-up or death, as appropriate."*

2. To ensure reproducibility, we present identical tumour network mapping analyses in the Extended Data in which pontine DMG cohorts are restricted to children with biopsy-confirmed tumours only. In doing so, we demonstrate that the prognostic maps of brain network connections associated with patient short-term survival constructed using data across all pontine DMG **(Extended Data Fig. 3f)**; all thalamic DMG **(Extended Data Fig. 3g)**; and all biopsy-confirmed pontine DMG **(Extended Data Fig. 3h)** are significantly similar to each other and to the paper primary result of all pontine and thalamic DMG **(Extended Data Fig. 3a).** The same held true of DMG network structural connectivity (**Extended Data Fig. 4a,f-h**). Finally, the prognostic impact of network-to-tumour connectivity was conserved when we restricted to a cohort of biopsy-proven pontine DMG only **(Extended Data Fig. 6f).** These control analyses are shown below for ease of reference.

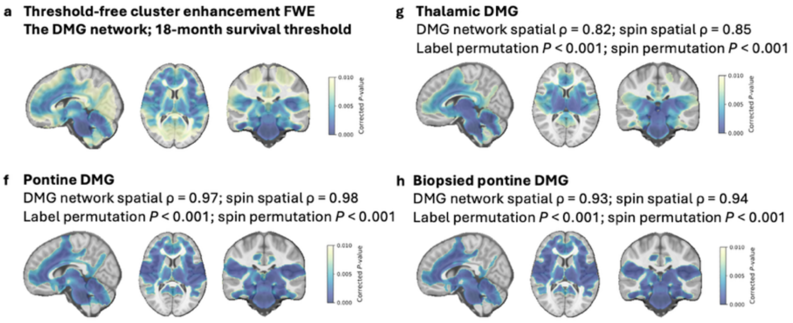

Extended Data Fig. 3a,f-h. Shared functional connectivity of pontine and thalamic DMG. *Visual and quantitative spatial permutation testing comparison of the DMG network **(A)** with prognostic brain networks constructed using identical methods across all pontine DMG **(F)**; all thalamic DMG **(G)**; and a restricted cohort of children with biopsy-confirmed pontine DMG **(H)**.*

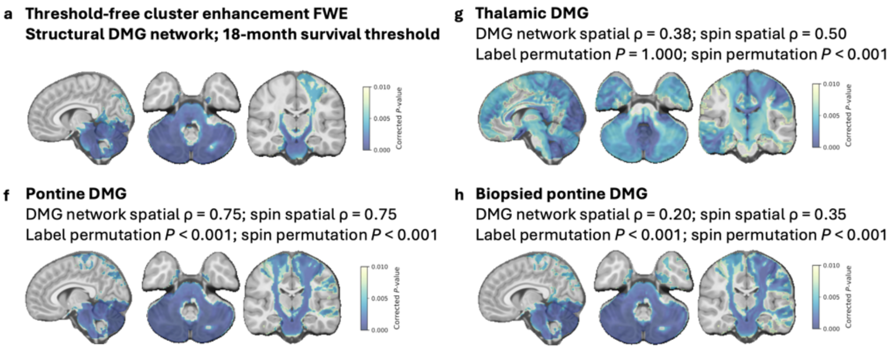

Extended Data Fig. 4a,f-h. Shared structural connectivity of pontine and thalamic DMG.
Visual and quantitative spatial permutation testing comparison of the DMG structural network ***(A)*** *with prognostic brain networks constructed using identical methods across all pontine DMG* ***(F)****; all thalamic DMG* ***(G)****; and a restricted cohort of children with biopsy-confirmed pontine DMG* ***(H)****.*

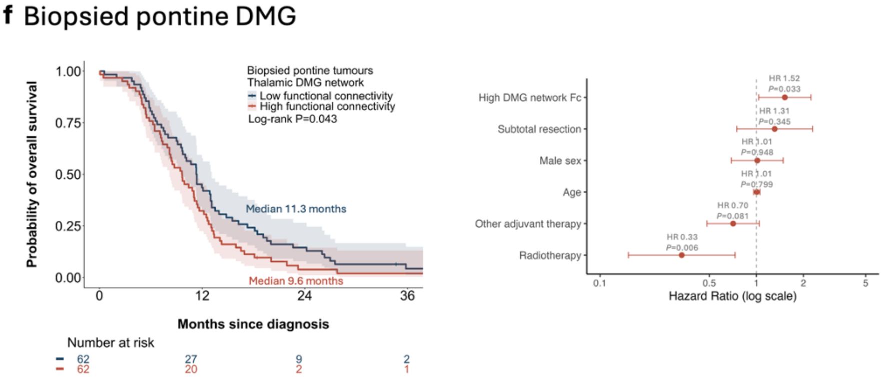

Extended Data Fig. 6f. Conserved prognostic impact of network-to-tumour connectivity in children with biopsy-confirmed pontine DMG.
External cohort network-to-tumour connectivity, computed using the discovery cohort DMG network, remained an independent prognostic factor when restricting to children with biopsy-confirmed pontine DMG. Kaplan-Meier median overall survival 9.6 vs. 11.3 months; 95% CI 8.2-11.4 vs. 9.7-13.1 months; log-rank P=0.043. Multivariable cox HR 1.52; 95% CI 1.03-2.23; P=0.033.

We have now also included additional text within the results to clarify and highlight these control data:

"The DMG network and its structural connectivity were robust to statistical methods (Extended Data Fig. 3a-d, Extended Data Fig. 4a-d); controlling for patient age and sex (Extended Data Fig. 3e, Extended Data Fig. 4e); controlling for tumour primary pontine or thalamic location (Extended Data Fig. 3f-g, Extended Data Fig. 4f-g); ***restricting to biopsied pontine DMG, H3K27-altered, only*** *(****Extended Data Fig. 3h, Extended Data Fig. 4h****); and controlling for mean tumour anatomic distortion (Extended Data Fig. 3i, Extended Data Fig. 4i).*

…

As previously described, this study included children with pontine DMG diagnosed via both clinical-radiological criteria and direct tissue biopsy. In addition to demonstrating the robust prognostic impact of DMG network-to-tumour connectivity on an independent validation cohort of children with biopsy-confirmed pontine DMG, H3K27-altered, only (PNOC trial cohort; Fig. 2b), we also restricted these pontine DMG on thalamic DMG network survival analyses to biopsied pontine tumours only, establishing an identical pattern of patient overall survival stratified by network-to-tumour connectivity (Extended Data Fig. 6f).*"*

3. Further to this, we have included a new and additional external validation cohort of 38 children with biopsy-confirmed pontine DMG treated within Pediatric Neuro-Oncology Consortium (PNOC) clinical trials PNOC003 and PNOC022 (NCT05009992 and NCT02274987, respectively). Identical tumour network mapping analyses performed across this cohort revealed a network of prognostic brain network connections identical to those defined in our discovery cohort DMG network, which included all children with pontine and thalamic DMG (**Main Text Fig. 1f**; whole brain spatial ρ=0.911, label permutation P<0.001; spin spatial ρ=0.885, spin permutation P<0.001). We also computed the network-to-tumour connectivity of patient tumours in the PNOC trial cohort to our discovery cohort DMG network, reproducing the prognostic impact of the DMG network across this cohort (**Main Text Fig. 2b**). Kaplan-Meier median overall survival 9.9 vs. 13.0 months; 95% CI 7.3-13.1 vs. 11.1- 21.9 months; log-rank *P*=0.052 Multivariable cox HR 2.68; 95% CI 1.17-6.13; *P*=0.020.

1f PNOC trial cohort network

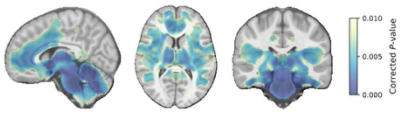

2b Independent PNOC trial cohort connectivity with the discovery DMG network

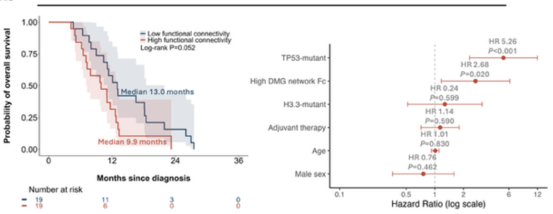

These texts are included in the main text results as below:

"To test for DMG network reproducibility, we performed tumour network mapping using identical methods across two independent cohorts of children with DMG: ***a multicentre external validation cohort, as previously described (n=125), and a second cohort of 38 children with biopsy-confirmed pontine DMG, H3K27-altered, included as a secondary analysis of Pediatric Neuro-Oncology Consortium (PNOC) trial data (Supplementary Table 1). Importantly, both independent cohorts demonstrated the same pattern of brain network connections associated with patient short-term overall survival, with strong and significant convergence to our primary, discovery cohort defined DMG network (Fig. 1e-f; Extended Data Fig. 4l).*** *Collectively, these data define a conserved, prognostic human brain network across pontine and thalamic DMG, connectivity with which is associated with shorter patient overall survival."*

…

"To validate the prognostic importance of the DMG network, the mean strength of network-to-tumour connectivity with the primary, discovery cohort defined DMG network was computed for each tumour across both external validation cohorts: data which were not used to define the DMG network. Network-to-tumour connectivity was computed as the weighted overlap of patient-level tumour functional connections with DMG network prognostic connections.[39] ***Kaplan-Meier survival analyses following the stratification of patient tumours using a median cut-off confirmed the inverse association of high network-to-tumour connectivity with overall survival in children with DMG across both external multicentre and PNOC trial validation cohorts (Fig. 2a-b). Importantly, univariable and multivariable cox-proportional hazards models incorporating clinical covariates reproduced these findings and confirmed network-to-tumour connectivity as an independent predictor of overall survival in children with DMG (Fig. 2a-b).*** *"*

Taken together, these data confirm identical results both in terms of DMG network spatial topography and the prognostic impact of network-to-tumour connectivity, across inclusion criteria for all children with pontine DMG and those with biopsy-confirmed pontine DMG, H3K27-altered.

****Figure data and relevant data comparing thalamic DMG to pontine DMG (again, are these all biopsied pontine tumors?) sees to show a relatively insignificant effect on the network in the pontine group. At best, the thalamic results are much more impressive which as a reader makes be believe there should be a much greater emphasis throughout the manuscript that this is prominent in thalamic networks, but much less important in pontine networks? I would recommend this is carried forward from the title through the manuscript, other the broad DMG classification could even inadvertently suggest equivalency of the findings in thalamus, pons, and spine when the spine is not even evaluated and the thalamic vs. pontine datasets are substantially distinct results.**

Thank you for raising this consideration. A key finding of our work is that, despite disparate anatomic origins, primary pontine and thalamic DMG share the same pattern of prognostic brain network connections associated with patient short-term overall survival and exhibit comparable network effects on patient overall survival.

We show this in several ways:

1. We demonstrate that the prognostic maps of brain network connections associated with patient short-term survival constructed using data across all pontine DMG **(Extended Data Fig. 3f)**; all thalamic DMG **(Extended Data Fig. 3g)**; and all biopsy-confirmed, H3K27-altered, pontine DMG **(Extended Data Fig. 3h)** are significantly similar to each other and to the paper primary result of all pontine and thalamic DMG **(Extended Data Fig. 3a).** The same held true of DMG network structural connectivity (**Extended Data Fig. 4a,f-h**), as shown above. This result demonstrates that the DMG network is conserved and reproducible across pontine and thalamic DMG.

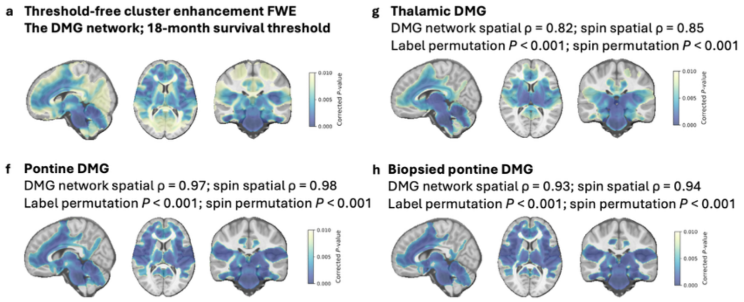

Extended Data Fig. 3a,f-h. Shared functional connectivity of pontine and thalamic DMG. *Visual and quantitative spatial permutation testing comparison of the DMG network **(A)** with prognostic brain networks constructed using identical methods across all pontine DMG **(F)**; all thalamic DMG **(G)**; and a restricted cohort of children with biopsy-confirmed pontine DMG **(H)**.*

2. In order to further confirm that pontine and thalamic DMG map to the same prognostic brain network, we took the networks generated from pontine DMG and thalamic DMG as shown above (**Extended Data Fig. 3f-g**) and computed the network-to-tumour connectivity of (1) thalamic DMG on the pontine DMG network and (2) pontine DMG on the thalamic DMG network. When patient tumours were stratified on the basis of their connectivity with these networks, a clear survival difference was identified across both pontine and thalamic DMG (**Main Text Fig. 2c-d**). We acknowledge that the magnitude of the raw survival difference in months is greater for thalamic than pontine DMG. However, both have comparable hazard ratios for network-to-tumour connectivity (1.42 pontine; 1.88 thalamic) which suggests a conserved network effect. Further, for pontine DMG, a disease without effective therapies and with median 10-month overall survival, a 1-month median survival difference (~10% overall survival) is clinically meaningful.

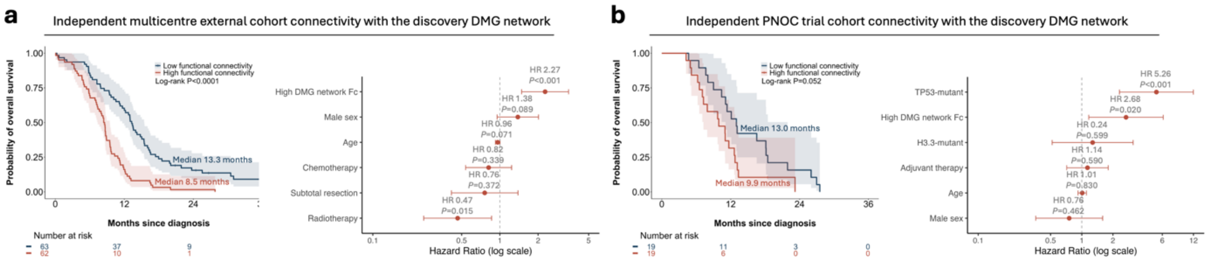

Main Text Fig. 2c-d. Conserved prognostic impact of network-to-tumour connectivity in children with pontine and thalamic DMG.
(C) *191 children with pontine DMG with network-to-tumour connectivity calculated on a thalamic DMG network. Kaplan-Meier median overall survival 8.7 vs. 9.7 months; 95% CI 8.2-9.6 vs. 8.9-11.4 months; log-rank P=0.046. Multivariable cox HR 1.42; 95% CI 1.06-1.90; P=0.019.*
(D) *59 children with thalamic DMG with network-to-tumour connectivity calculated on a pontine DMG network. Kaplan-Meier median overall survival 12.2 vs. 17.0 months; 95% CI 8.3-16.6 vs. 13.4- 31.0 months; log-rank P=0.026. Multivariable cox HR 1.88; 95% CI 1.03-3.46; P=0.041.*

3. We demonstrate that the longitudinal growth pattern of pontine and thalamic DMG map to the same DMG network nodes such that these tumours, despite distinct primary locations, have a conserved pattern of tumour anatomic progression, further reflecting their shared brain network connectivity and conserved network effects (**Main Text Fig. 3**; next page).

Together, these data demonstrate that pontine and thalamic DMG not only share the same pattern of prognostic brain network connections but that, independently, the overall survival of children with primary pontine DMG can be predicted from tumour connectivity with the thalamic network and vice versa. Similar hazard ratios obtained speak to an effect of similar magnitude despite different raw survival differences.

We agree that future studies should evaluate the potential impact of neuron-glioma interactions in children with spinal DMG. However, using current paediatric connectomes, it is not possible to evaluate connectivity within the spine or between the brain and spine. To partially speak to this question, we did include a cohort of children with intracranial metastases of primary spinal DMG, showing that these metastases map anatomically to the same DMG network and suggesting a similar, potentially tropic, network effect. These analyses are discussed in more detail in a second comment by the same reviewer in terms of tumour metastatic growth. We have added a sentence to the discussion in order to document this limitation, as below.

"Whilst we show that similar tropism applies to parenchymal intracranial metastases of primary spinal DMG, it remains to be demonstrated if similar network effects are associated with overall survival in these children."

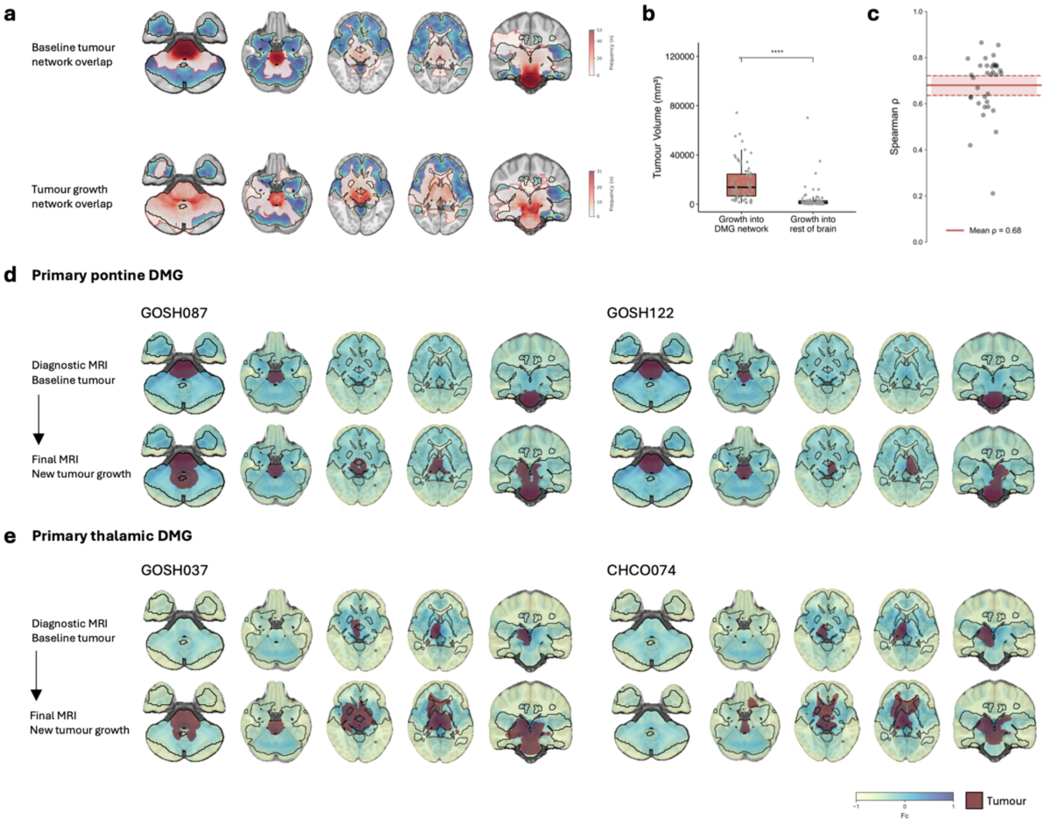

Main Text Fig. 3. Tumour growth follows DMG network trajectories.
(A) *Tumour-network overlap maps illustrate the anatomic distribution of DMG at baseline diagnostic MRI (top row) and new tumour growth from diagnostic to final MRI within two months of death (bottom row) for a subcohort of 71 children with paired MRI available for analysis. Tumour burden is plotted in red-white (red outline) as a summed heatmap representative of voxelwise frequency and DMG network values in blue-yellow (black outline).*
(B) *Tumour volumetric growth (mm^3) into the DMG network was significantly greater than into any other brain region between diagnostic and final MRI (median 13,768 vs. 1,270 mm^3; 95% CI 11,293-17,763 vs. 773-1,792 mm^3; P=6.85e^{-13}). Patient-level tumour growth volumes are shown in grey.*
(C) *Tumour growth into pre-defined DMG network nodes exhibited a strong positive dependence with node-to-tumour connectivity, correcting for the distance of tumour infiltration to each network node (mean Spearman's ρ=0.68; 95% CI 0.63-0.72). All but two individual patient correlations were significant after Benjamini-Hochberg correction ($P_{FDR}<0.05$) and the mean correlation was significantly greater than zero (two tailed Wilcoxon signed-rank test P=1.16e^{-10}). Patient-level rho-values (ρ) are shown in grey with the group-level median in red with bootstrapped 95% CI.*
(D-E) *Representative patient tumours at baseline diagnostic and final MRI across pontine* ***(D)*** *and thalamic* ***(E)*** *DMG (red) overlayed onto the DMG network (network peaks in dark blue, black outline). Tumour growth trajectories are plotted for each patient as a three-dimensional representation of tumour spread into DMG network nodes. GOSH087 and GOSH122 are illustrative of two pontine DMG, initially confined to the brainstem, which progressed to involve the thalami via the corticospinal tracts; GOSH087 also invaded the cerebellum. GOSH037 and CHCO74 are illustrative of two DMG, initially confined to the right thalami, which progressed to involve the orbitofrontal cortices, genu of the corpus callosum, contralateral thalami, deep grey matter, pallidum, and hippocampi. Both tumours also grew caudally: GOSH037 progressed through the midbrain, medulla, and pons to the cerebellum via the superior and middle cerebellar peduncles whilst CHCO074 progressed into the midbrain. These patterns of growth strongly aligned with DMG network connectivity, as shown.*

****What is the overlap of diffusion imaging data for this cohort? Couldn't the effect on the network be explained by simply more tumor cells present?**

This is an interesting question. Diffusion imaging data were leveraged in the context of a paediatric normative structural connectome across 497 healthy children from the Human Connectome Project Lifespan Development (HCP-D) dataset. We used this connectome to define the monosynaptic structural connections, associated with shorter patient overall survival, which DMG have with the rest of the brain (**Extended Data Fig. 4a**). Strong connectivity was observed with brain regions identified in the DMG network, particularly via the cerebellar peduncles, corticospinal tracts, corticobulbar tracts, and spinal-limbic pathways. This DMG structural network, despite reproducibility across methodological choices and two datasets as well as robustness to covariates, was non-prognostic (**Extended Data Fig. 7a**), emphasising the importance of distributed, polysynaptic connections on patient overall survival – as in our primary result derived from paediatric functional connectivity data.

4a Threshold-free cluster enhancement FWE
Structural DMG network; 18-month survival threshold

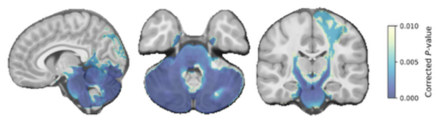

7a Independent multicentre external cohort connectivity with discovery cohort structural network

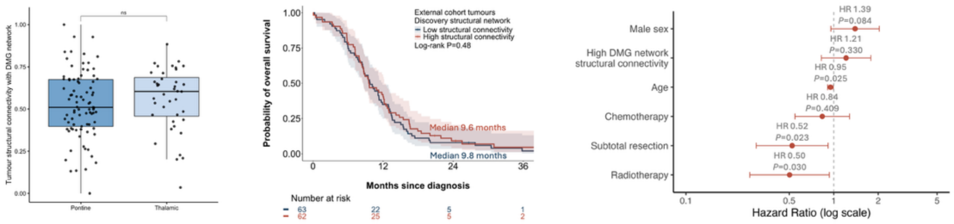

Extended Data Fig. 4a. DMG network structural connectivity.
(A) *DMG network mapping was repeated using dMRI data across 497 healthy children from the Human Connectome Project Lifespan Development (HCP-D) dataset, identifying a structural network of monosynaptic tumour connections associated with shorter patient overall survival* ***(A)****. Strong connectivity was observed with brain regions identified in the DMG network, particularly via the cerebellar peduncles, corticospinal tracts, corpus callosum, corticobulbar tracts, and spinal-limbic pathways.*

Extended Data Fig. 7a. DMG network functional connectivity, rather than DMG network structural connectivity, is associated with patient overall survival.
(A) *Comparison of external validation cohort network-to-tumour connectivity values revealed that there was no significant difference in structural connectivity to the DMG network between pontine DMG and thalamic DMG (median 0.510 vs. 0.604; two-tailed Mann-Whitney U-test P=0.234). DMG network-to-tumour structural connectivity was not independently associated with patient overall survival across the external multicentre validation cohort* ***(****centre and right****)****. Kaplan-Meier median overall survival 9.8 vs. 9.6 months; 95% CI 8.6-11.9 vs. 8.6-12.2 months; P=48. Multivariable cox HR 1.21; 95% CI 0.82-1.78; P=0.330.*

Though we did collect patient-specific diffusion imaging data from patients including in this study across all study sites, it was of insufficient and variable quality, precluding meaningful analysis. To speak directly to the question raised, we used patient tumour segmentations to assess the impact of tumour volume on patient overall survival. In doing so, we failed to identify as association between tumour volume and overall survival (univariate cox hazard ratio 1.00; *P*=0.569), in line with recent clinical literature (Jansen … Vuurden. *Neuro Oncol*. 2015.; Tam … Yeom. *Neuro Oncol Adv*. 2021.). We have included additional text in the results to highlight this finding:

"*Here, we perform tumour network mapping with the aim of identifying common network connections associated with DMG patient short-term survival, irrespective of tumour primary pontine or thalamic location.* ***Greater tumour volume was not associated with patient overall survival (univariate cox hazards ratio 1.00; P=0.569), in keeping with previous clinical reports[47,48], and so tumour***

network mapping analyses were performed controlling for volume, in line with previous work.[40,43,45]"

As also raised by Reviewer 5, by demonstrating a lack of association between tumour volume and patient overall survival in this manner, and by including tumour volume as a covariate in PALM when defining the DMG network, we are now able to avoid our extensive use of post-hoc mediation analyses and more elegantly show the independence of network effects from tumour volume.

****DMG are known to be metastatic, how was the presence of metastases incorporated in the analysis? Does local extension or far distant tumor metastases associate with any findings?**

Thank you for raising this important point. A central aspect of our manuscript is the analysis of how DMG metastatic growth maps to DMG network trajectories. We have revised the text to clarify this and to ensure that one figure (**Main Text Fig. 3**) focuses specifically on demonstrating this finding.

In brief, we first analysed tumour metastatic growth for a subcohort of 71 children with DMG who had a brain MRI within 2 months of death: a final picture of the disease. Comparison of tumour-network overlap maps at diagnosis and final follow-up MRI (**Main Text Fig. 3A**) shows significantly greater tumour burden within the DMG network than outside of the DMG network, as verified quantitatively in **Main Text Fig. 3B**. We then analysed a further restricted subcohort of 34 children whose tumour metastasised beyond the primary site. For these children, we asked whether tumour volumetric burden in defined, brain-wide DMG network nodes (for example, the hippocampus and primary motor cortex) would correlate with how connected each patient's tumour was with that node, correcting for the distance of each tumour from that node. Spearman's rho (ρ) for all but two patient tumours demonstrated a significant positive correlation (**Main Text Fig. 3C**), demonstrating that DMG metastasise towards network nodes with which they are most connected. In our manuscript, we refer to this as "tumour growth" rather than "tumour metastasis" though have clarified this in several sentences, for example: *"These combined observations led us to investigate whether tumour **metastatic** growth exhibits directionality to defined peaks in the DMG network."*.

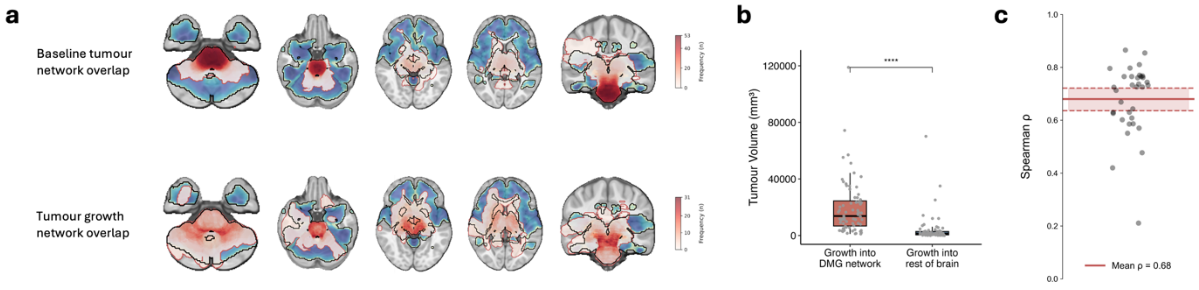

Main Text Fig. 3A-C. Tumour growth follows DMG network trajectories.
(A) *Tumour-network overlap maps illustrate the anatomic distribution of DMG at baseline diagnostic MRI (top row) and new tumour growth from diagnostic to final MRI within two months of death (bottom row) for a subcohort of 71 children with paired MRI available for analysis. Tumour burden is plotted in red-white (red outline) as a summed heatmap representative of voxelwise frequency and DMG network values in blue-yellow (black outline).*
(B) *Tumour volumetric growth (mm^3) into the DMG network was significantly greater than into any other brain region between diagnostic and final MRI (median 13,768 vs. 1,270 mm^3; 95% CI 11,293-17,763 vs. 773-1,792 mm^3; $P=6.85e^{-13}$). Patient-level tumour growth volumes are shown in grey.*
(C) *Tumour growth into pre-defined DMG network nodes exhibited a strong positive dependence with node-to-tumour connectivity, correcting for the distance of tumour infiltration to each network node (mean Spearman's ρ=0.68; 95% CI 0.63-0.72). All but two individual patient correlations were significant after Benjamini-Hochberg correction ($P_{FDR}<0.05$) and the mean correlation was significantly greater than zero (two tailed Wilcoxon signed-rank test $P=1.16e^{-10}$). Patient-level rho-values (ρ) are shown in grey with the group-level median in red with bootstrapped 95% CI.*

We also investigated whether the pattern of parenchymal (non-leptomeningeal) intracranial metastases of primary spinal DMG mapped to the DMG network. Anatomically, significant overlap in tumour burden was shown, with a similar significantly greater in-network than out-of-network tumour volume (**Extended Data Fig. 8.**).

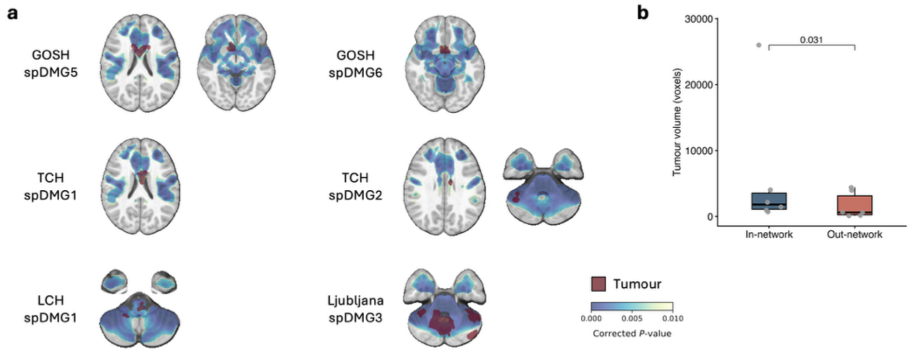

Extended Data Fig. 8. Intracranial metastases of primary spinal DMG exhibit anatomic tropism to the DMG network.
(A) *Six children with parenchymal (non-leptomeningeal) metastases of primary spinal DMG were identified from a large, independent international cohort of children with primary spinal DMG (n=36). All intracranial lesions anatomically mapped to the DMG network. GOSH: Great Ormond Street Hospital for Children NHS Foundation Trust; LCH: Lurie Children's Hospital; spDMG: spinal DMG; TCH: Texas Children's Hospital.*
(B) *Metastatic tumour volume (mm^3) in the DMG network was significantly greater than in the rest of the brain (median 1,813 vs. 618.5 mm^3; IQR 712-2,600 vs. 94-4,415 mm^3; two-tailed paired Wilcoxon signed-rank test P=0.031). Patient-level tumour volumes are shown in grey.*

Collectively, these data demonstrate that DMG metastatic growth aligns to circuit-specific trajectories within the DMG network. Tumour local extension was, volumetrically, significantly greater in-network than out-of-network brain whilst far distant metastases were more common in specific network nodes with greatest connectivity to the primary tumour.

****The connection of tumor initiation/growth/development to neurometabolic development is unclear. The area under the curve in Figure 4a would also include many other childhood brain tumors, for example medulloblastoma peaks between ages 4-7 but also is not dissimilar from the overall median age of all paediatric brain tumors. How is this being associated with DMG as opposed to other tumors? If tumors like medulloblastoma have overlapping incidences would it be beneficial to compare another subset of tumors hypothesized to not have this network and compare results?**

We thank the reviewer for sharing this important observation. We analysed [^{18}F]FDG-PET data across childhood to interrogate whether DMG network connections predictive of patient overall survival underwent substantially greater transitions in neurometabolic activity than any other brain areas or connections, and whether these changes mapped temporally to DMG peak age incidence. In doing so, we establish an association between DMG network connectivity, age-wise peak changes in neurometabolic activity, and the characteristic and bimodal age incidence of DMG.

As the reviewer notes, it is very true that several other childhood brain tumours also peak in incidence at these ages although none – to our knowledge – mimic the bimodal peak incidence of DMG. With this in mind, we take care not to state that the association of neurometabolic development with tumour incidence is specific to DMG, solely that a clear correlation is identified which warrants further mechanistic studies in preclinical models. Having thought carefully on the reviewer's concern and with this in mind, we thought it important to test whether the spatial topography of peak neurometabolic changes at the ages of peak DMG incidence (6 and 11 years, respectively), were significantly more similar to the DMG network than could be expected by chance. To this end, we implemented label and spin permutation testing, whereby each age-wise neurometabolic change was spatially compared to the DMG network against 10,000 null networks generated either through the random shuffling of survival data used to generate the DMG network or through random rotation of DMG network brain

regions 10,000 times. These analyses confirmed the spatiotemporal alignment of peak DMG incidence with peak, in-network changes in neurometabolic activity. To clarify this, we have added the following text to the results and have greatly simplified **Main Text Fig. 5**:

*"Strikingly, these data show that the peak positive neurometabolic change during childhood between 5-6 years of life is spatially dominated by brain areas in the DMG network, including the pons, whilst the second largest neurometabolic change during childhood, between 10-11 years of life, is also spatially dominated by brain areas in the DMG network, predominantly the thalamus (**Fig. 5). Importantly, anatomic maps of the peak neurometabolic change for ages 5-6 years and 10-11 years were significantly more similar to the DMG network than expected by chance upon both label and spin permutation testing and in contrast to peak neurometabolic changes at other ages** (**Extended Data Table 1**). Together, these data characterise the DMG network as a site of spatiotemporally organised neurometabolic development associated with tumour incidence."*

Main Text Fig. 5. DMG network topography spatiotemporally coincides with neurometabolic development.

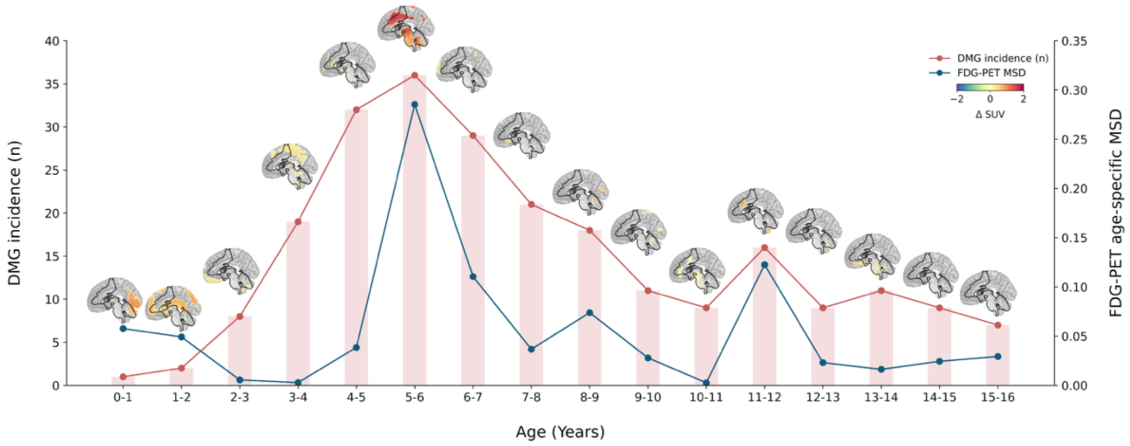

Line, bar, and scatter plot of whole cohort DMG age incidence (red; n=284, four patients aged >17 years omitted) with line and scatter plot of age-consecutive mean-squared differences (MSD) in brain-wide [18^F]FDG-PET signal (blue) across childhood (0-16 years) show the temporal alignment of peak DMG incidence with peak [18^F]FDG-PET signal changes (R=0.612, linear regression P=0.118). Brain regions with peak difference in [18^F]FDG-PET signal at each age across childhood are shown as brain-wide spatial maps plotted above each bar. Brain regions were identified by tagging each voxel across the brain with the age at which it underwent its greatest change in in [18^F]FDG-PET signal. Colours represent the voxelwise strength and direction of change in [18^F]FDG-PET signal between consecutive ages (red indicative of greater positive change, blue greater negative change). Peak changes in [18^F]FDG-PET signal between ages 5 and 6 years and 10 and 11 years, coinciding with age incidence peaks in DMG, strongly overlap with regions in the DMG network (ages 5-6 years peak changes DMG network whole brain spatial ρ=0.199, label permutation P<0.001, spin spatial ρ=0.512, spin permutation P<0.001; ages 10-11 years peak changes spatial ρ=0.044, label permutation P<0.001, spin ρ=0.179, spin permutation P<0.001).

As there is currently no evidence for the role of neuron-tumour synaptic communication in – for example – medulloblastoma, correlative analysis of medulloblastoma age incidence with changes in neurometabolic activity without confirmed network properties would risk incorrect extrapolation of preclinical evidence. Indeed, the role of neuronal or neurometabolic activity in other childhood brain tumours remains to be elucidated and we agree with the reviewer that more work is required to understand the tumour subtype-specific cancer neuroscience mechanisms at play. To this end, we have also added the following text to the discussion:

*"We further show that peak changes in neurometabolic activity across childhood localise to the DMG network, corresponding to the developmental emergence of brain circuits connected to and potentially communicative with DMG… **Whether such an association between physiologic neurometabolic activity and tumour incidence extends to other cerebral tumours of childhood, many of which share a similar pattern of early childhood peak incidence, remains to be demonstrated and requires a greater understanding of tumour subtype-intrinsic neuron-glioma interactions.**"*

Minor

****I would recommend a refrain from using "real-world", if the goal is to distinguish from preclinical work that could be stated in other ways.**

We thank the reviewer for this suggestion and agree with their recommendation. To this end, we have removed references to 'real-world' throughout the manuscript whilst using other phrases to denote distinctions between human and preclinical work, where appropriate.

****Conflicts are listed to include FxNeuromodulation and MapLight Therapeutics, however it was unclear how they were related to this work. Were they technologies incorporated in the analysis?**

These disclosures are for interests outside of the submitted work. More specifically, they did not influence the submitted work and had no role in any of the presented data and / or analyses. We have clarified this statement as below:

'**Outside of the submitted work, A.H. reports lecture fees for Boston Scientific and is a consultant for FxNeuromodulation whilst M.M. holds equity in MapLight Therapeutics. No financial or other relationships exist which influenced the submitted work.**'

G. No concerns

H. Discussed above

Referee #2 (Remarks to the Author):

This very interesting set of analyses implicates a highly interconnected network of brain regions in poor prognosis of childhood diffuse midline glioma. The authors interpret anatomical brain tumour data from a rare patient cohort in the context of normative MRI and PET imaging data. Although the presented approach is inferential rather than causal, the converging findings support recent proposals that neural activity might play a role in growth of glial brain tumours. The results in a sub-group of operated children further suggest that removing tumour subregions highly integrated with this network may also prolong survival.

Outstanding features of the work include:
1) use of an independent replication cohort and metastatic tumours of similar cell origin;
2) supplementation of the normative brain connectivity data with normative molecular and receptor density imaging data;
3) high quality and comprehensive post-hoc analyses exploring the specificity of the findings (e.g. robustness against different processing / statistical choices; verification that resting network topology is not affected by possible age differences using 9-10 year-old vs adult open datasets).

Thank you for your positive evaluation of our work and for your insightful critique. We are of the opinion that the additional analyses incorporated have strengthened the data presented, particularly regarding our tumour location mapping and network control analyses, for which we are grateful.

A few suggestions to help reinforce and support the proposed findings:
The paper's aim is to characterise brain-to-tumour connectivity and its relationship with clinical prognosis. A methodological uncertainty is how accurately networks derived from normative data represent connectivity and localisation of small subcortical nuclei in individuals with anatomy-distorting pathology. Spatial displacement of fibre bundles is well characterised in adult gliomas. I am less familiar with the paediatric literature but tract displacement has been described in some childhood thalamic (Kiran et al 2013 doi.org/10.1007/s10143-013-0452-3; Kim et al 2018, doi.org/10.14791/btrt.2018.6.e14) and brainstem tumours (Helton et al 2006, PMID: 16611765 ; Xiao et al 2021, doi.org/10.3171/2020.10.FOCUS20166). Effects on connections and structures inside the thalamus could be less pronounced. However, a small case series of 5 thalamic gliomas with diffusion MRI showed marked displacement of thalamic nuclei that was not predictable from anatomical scans (Kis et al 2014, DOI: 10.1227/NEU.0000000000000333). Since registration algorithms cannot resolve boundaries at the nuclear/tract/functional level, inaccuracies in these substructures seems inevitable. It would enhance confidence in the findings if the authors could show that normative connectivity predictions do, or are likely to, hold true in midline gliomas. Perhaps there are relevant exemplars within open databases containing diffusion MRI data (e.g. https://www.cancerimagingarchive.net/collection/ucsf-pdgm/)?

We thank the reviewer for this thoughtful comment and have taken care to address this question as fully as possible.

Firstly, we think it is important to recognise that, whilst DMGs and other diffuse gliomas, including adult glioblastoma, *IDH*-WT, do distort local anatomy to some extent, these tumours characteristically infiltrate the brain parenchyma, typically invading structures rather than compressing, distorting, or displacing them (Claes … Wesseling. *Acta Neuropathol*. 2007.). This observation may be first credited to Scherer's description of perineural satellitosis, the clustering of glioma cells around neuronal cell soma, in the late 1930s and early 1940s (Scherer. *Brain*. 1940.; reviewed in Gillespie & Monje. *Neuro Oncol*. 2010.). Clinically, we see this manifest in children who present with very large high-grade gliomas – at times involving a whole cerebral hemisphere – but who are not hemiplegic or profoundly disabled in the way you may expect for any other brain lesion of similar size. Indeed, early clinical reports note that it is rare for a child with DMG to present with hydrocephalus or pressure symptoms when compared to other cerebral tumours of childhood, including those located within the posterior fossa (e.g. medulloblastoma, pilocytic astrocytoma): children with DMG much more commonly present with focal neurologic deficits reflecting the pattern of tumour anatomic infiltration (Donaldson … Fisher. *J Clin Oncol*. 2006.; Hoffman … Fouladi. *J Clin Oncol*. 2018.). We also see this at the tissue-level: post-mortem studies have demonstrated that DMG cells diffusely infiltrate, rather than distort, patient anatomy – for example with invasion of the hippocampus, as demonstrated below (Caretti … Monje. *Acta Neuropathol*. 2014.; Barron … Monje. *Nature*. 2025.). In a similar manner, brainstem infiltration – rather than overt compression or destruction – is increasingly recognised as a marker of progressive GBM (Sahm … Deimling. *JAMA Neurol*. 2012.; Drumm … Horbinski. *Neuro Oncol*. 2020.)

Redacted

These observations speak to evidence from adult human patients with GBM and the fact that glioma-infiltrated brain circuits retain function: Krisha *et al*. demonstrate that neural activity within tumour-affected cortex is physiologically organised such that glioblastoma cortical infiltration maintains task-specific neuronal responses, including neuronal hyperexcitability, with tumour-affected cortex losing the ability to decode only complex word conditions (Krishna … Hervey-Jumper. *Nature*. 2023.). This functional re-organisation, rather than destruction, speaks to our preclinical observations – namely that DMG and other high-grade gliomas synaptically integrate with physiologically functional neural circuits, activity from which promotes tumour growth in an activity-dependent manner (Venkatesh … Monje. *Nature*. 2019.).

Redacted

Secondly, the potential effect of anatomic / tract distortion by tumours was screened and corrected for at tumour segmentation: all tumour segmentation masks were reviewed in patient and MNI space and manually corrected, if required (i.e. if inaccurately transformed), by a senior board-certified paediatric neuroradiologist with >15 years post-qualification experience. We carefully reviewed the literature highlighted by the reviewer and think it important to note that distortion of tracts does not preclude tumour infiltration of and communication with the same tract. For example, upon review of Kis *et al*., the thalamic nuclei, whilst displaced by tumours across patients, would still be considered infiltrated – in all instances, at least in part – upon neuroradiological review and so would have been included in our segmentation masks and connectivity seeds (Kis … Barzo. *Neurosurgery*. 2014.). Below, an exemplar patient from Kis *et al*. shows how the thalamic nuclei have been, to our mind, incorrectly categorised as separate to the tumour beside the tumour segmentation in the same plane drawn by our team in pink inclusive of all 'displaced' thalamic nuclei. Further to this, Kis *et al*. use methods from Behrens *et al*. *Nat Neuro*. 2003. to perform "*thalamic segmentation based on probabilistic DTT in thalamic glioma patients*" based on the "*find_the_biggest command on the thresholded output of "probtrackx"*. Simply put, Kis *et al*. computed the respective connectivity profiles of each nucleus with the rest of the brain in order "*to determine whether it may help to differentiate thalamic nuclei from the tumor tissue and localize them.*" Given that the tumour was not delineated from the healthy thalamus *a priori*, connectivity profiles of each thalamic nucleus defined across patients could be reflective of the connectivity profile of tumour tissue which has invaded the thalamic nuclei. As such, this is a flawed method to differentiate tumour tissue from healthy thalamic tissue – particularly given the overwhelming preclinical and clinical evidence for neuron-glioma synaptic integration (Venkatesh … Monje. *Nature*. 2019.; Taylor … Monje. *Nature*. 2023.; Barron … Monje. *Nature*. 2025.; Drexler … Monje. *Cell*. 2025.).

Redacted

In addition, across the referenced literature, authors provide tractographic 'evidence' for complete interruption of the corticospinal tracts within DMG tumours with apparent clinical absence of corresponding neurologic symptoms that would be expected with such transection – notably hemiparesis. Indeed, Helton *et al*. report 'mild' corticospinal symptoms at worst in 3/7 reported patients and 'absent' corticospinal symptoms in 4/7 reported patients (Helton … Ogg. *Am J Neuroradiol*. 2006.). Similarly, though Xiao *et al*. do not report tumour subtype-specific data and include only 5/54 brainstem glioblastomas (presumably DIPGs) and 15/54 grade 3/4 high-grade glioma, the reported prevalence of corticospinal tract interruption across all patients was 27/54 (25.0%) whilst the rate of reported hemiparesis was just 8/54 (14.8%) (Xiao … Zhang. *Neurosurg Focus*. 2021.). Such a discrepancy is difficult to reconcile clinically given that true interruption of the corticospinal tract would ordinarily be expected to produce significant and corresponding motor deficit. Taken together, these data suggest that some commonly used tractography techniques, which are typically deterministic and applied to poor-quality dMRI data, are not adequate to resolve the complex structural interactions between diffuse gliomas and neurons/tracts.

We do fully agree with the reviewer, however, that the question of whether tumour anatomic distortion could have influenced tumour network mapping results warrants due consideration. To this end, we have included an additional control analysis to evaluate the potential impact of anatomic distortion introduced by tumour growth within tumour network mapping. In this analysis, the degree of anatomic distortion for each tumour in our discovery cohort was defined as the mean ANTs SyN registration deformation field required to transform each patient brain into MNI space. Mean neuroanatomic distortion per patient was then covaried with patient short- or long-term survival and tumour volume in PALM to generate a control DMG network. The results of tumour network mapping with correction for tumour mean anatomic distortion across both functional and structural connectivity data were spatially compared to the DMG network using label and spin permutation tests. These demonstrated high topographical convergence across both functional and structural connectivity data and suggest that any potential tumour anatomic distortion has a minimal effect on brain-wide patterns of prognostic tumour connectivity (**Extended Data Fig. 3i; Extended Data Fig. 4i**). Additional text and figures have been included to describe these results and methods, as below for ease of reference:

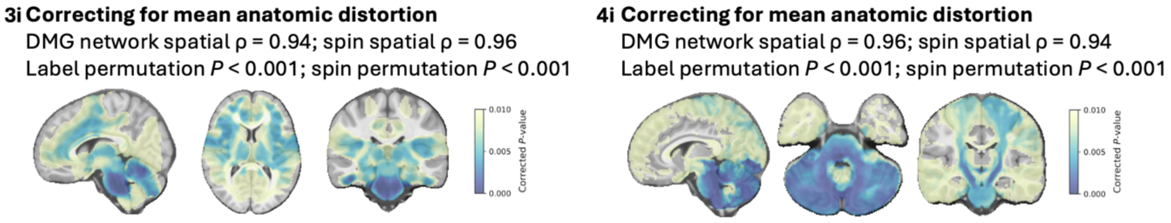

Extended Data Fig. 3i; Extended Data Fig. 4i
Identical tumour network mapping analyses performed across functional ***(3i)*** *and structural* ***(4i)*** *connectivity data demonstrate strong convergence to the primary DMG network upon label and spin permutation testing (P<0.001 in all instances).*

Main text results
"The DMG network and its structural connectivity were robust to statistical methods (Extended Data Fig. 3a-d, Extended Data Fig. 4a-d); controlling for patient age and sex (Extended Data Fig. 3e, Extended Data Fig. 4e); controlling for tumour primary pontine or thalamic location (Extended Data Fig. 3f-g, Extended Data Fig. 4f-g); restricting to biopsied pontine DMG only (Extended Data Fig. 3h, Extended Data Fig. 4h); ***and controlling for mean tumour anatomic distortion (Extended Data Fig. 3i, Extended Data Fig. 4i)***."

Methods
"Whilst all tumour segmentations normalised to MNI space were manually checked for neuroanatomic accuracy by a board-certified paediatric neuroradiologist (K.M.) against patient-specific imaging prior to seeding in ABCD1000, to further rule out the possibility that DMG-induced neuroanatomical distortions might influence tumour-specific normative functional connections, we also conducted an additional experiment covarying patient overall survival with mean tumour-associated anatomic distortion. Mean tumour-associated anatomic distortion was calculated by averaging the voxelwise SyN deformation fields required to normalise each patient brain to MNI space."

Beyond this control analysis, we did aim to collect patient-specific dMRI data from DMG patients across all included cohorts but were limited in our capacity to perform meaningful analysis due to heterogeneity in data quality across patients and centres (n=1 high angular resolution dMRI).

Redacted

Interpretationally, the data seem consistent with the idea that neural activity plays a role in glioma growth. A question is whether co-localisation offers causal evidence, and how well we can differentiate “neural activity” that is incidental vs instrumental to tumour growth?

Thank you for this insightful question. We agree that it is important to consider the level of causal evidence achieved by our data. As the spatial distribution of DMG in our cohort represent ‘incidental lesions’ – which we show to have conserved connectivity with a brain network associated with overall survival and tumour growth – tumour network mapping alone fulfils basic criteria for causality in human brain mapping (Siddiqi … Fox. *Nat Rev Neurosci*. 2022.). We developed this framework with reference to previous lesion network mapping studies of acute symptoms which arise secondary to brain lesions. In the present study, the strength of the causal relationship described is somewhat limited by the temporal delay between lesion onset (in this case, gliomagenesis) and the outcome of interest (in this case, overall survival), though this is partly overcome through extensive multivariable survival analyses incorporating all known clinical covariates. This said, the orthogonal analyses employed to validate the prognostic and biologic impact of the DMG network each fulfil causality criteria within this framework. To expand:

1. Dose-response, which refers to the association of a greater network effect with a greater behavioural outcome, is demonstrated in our data by tumour network mapping alone – in which connections associated with patient overall survival are defined (**Main Text Fig. 1.**). This observation, that patient tumours with greater DMG network connectivity exhibit shorter overall survival, is further confirmed across our survival analyses on external validation cohort patient data (**Main Text Fig. 2.**).

2. Specificity, which refers to the association of a network effect with a specific behavioural outcome rather than any other outcome, is shown in our data by demonstrating that the DMG network specifically predicts the overall survival of children with DMG rather than, for example, that of adult patients with glioblastoma, *IDH*-WT, despite shared mechanisms of neuron-glioma interactions in both tumour types (in addition to distinct mechanisms) (**Extended Data Fig. 6g-h.**).

3. Experimental manipulation, which refers to the association of targeted network perturbations with measurable differences in behavioural outcome, is shown in our data by the significant prognostic advantage of incidental, volume-agnostic high-connectomic resections in children with thalamic DMG (**Main Text Fig. 6.**).

4. Convergence, which refers to the convergence of results across data modalities and analyses, are demonstrated in the present manuscript by convergent results across functional connectivity, survival, growth, neurometabolic activity, snRNA-seq, and DNA methylation data. This criterion, in particular, enhances our confidence in the potential causal implications of the data presented for network effects on DMG growth and progression. Importantly, newly generated and analysed snRNA-seq data show that high-connectivity DMG exhibit relative enrichment of neuronal character and diverse synaptic genes (**Main Text Fig. 4.**) Not only this, but high-connectivity DMG were enriched for genes upregulated in response to NLGN3 exposure. NLGN3 is secreted in an activity-dependent manner and is required for DMG proliferation (Venkatesh … Monje. *Cell*. 2015.; Venkatesh … Monje. *Nature*. 2017.). Together, these tissue-level data suggest increased neuronal input in high-connectivity DMG and directly link DMG network connectivity with known mechanisms of DMG neuron-glioma interactions. Importantly, such an approach is being adopted elsewhere: snRNA-seq analysis of resected tumour tissue to confirm on-target effect via enrichment for tumour connectivity signatures is a secondary endpoint for the ongoing phase IIa trial investigating the use of perampanel for GBM, *IDH*-WT (PerSurge) (Heuer … Winkler. *BMC Cancer*. 2024.).

Collectively, whilst we do not provide direct evidence for neural activity modulating DMG growth in children, we do provide convergent evidence consistent with this hypothesis across multiple data modalities and temporal scales. Our use of convergent causal human brain mapping techniques and, in particular, our validation of DMG network connectivity at the tissue-level, offer robust evidence in support of the hypothesis that tumour connectivity is a reflection of underlying neural activity with measurable effects on patient-centred outcomes (overall survival; tumour volumetric growth).

Redacted

Though a full discussion of these ideas within the present manuscript is out of scope, we have included additional text within the discussion to reflect their importance, as below:

*"***Finally, none of the experiments presented herein demonstrate nor could have demonstrated a direct relationship between tumour neural activity and tumour growth in children. Within the framework of causal human brain mapping[42], however, the convergent data presented across multiple modalities are consistent with the hypothesis that DMG network connectivity is associated with DMG tumour growth and patient overall survival.***"*

The relative sparing of the occipital and parietal lobes (Fig 1, Fig 3f) is interesting (also noted in adults), given the thalamus is proposed as a central coordinating hub. Could the preferential involvement of some, but not all, highly-connected thalamic regions alternatively point towards characteristics other than neural activity facilitating tumour growth, or effecting a worse outcome from this pattern of growth?

This is an interesting question. To first enable better visualisation of the thalamic subregion(s) most implicated in DMG network topography, we included a plot of the 18 DMG network peaks derived from FSL cluster in **Main Text Figure 1**, as shown below:

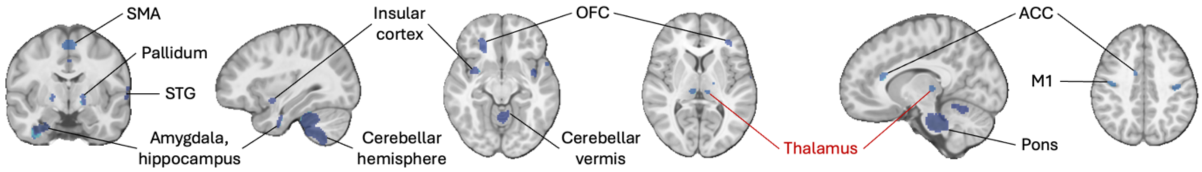

Main Text Fig. 1d. DMG network peak clusters.
DMG exhibit a specific pattern of brain functional connectivity associated with patient short-term overall survival. This includes strong positive connectivity with the centromedian, medial pulvinar, and ventral intermediate nuclei (labelled red for the purpose of this response).

This demonstrates that the DMG network includes fewer thalamic subregions connected to occipital and parietal cortices and instead exhibits peaks in the centromedian (CM), medial pulvinar, and ventral intermediate (Vim) nuclei. The former are association nuclei with widespread connectivity with the frontal, insular, cingulate, and somatomotor cortices – in line with DMG network topography and their known roles in arousal and attention (Setzer … Lewis. *Nat Commun*. 2022.) as well as higher-order cognition (Homman-Ludiye … Bourne. *J Anat*. 2019.) whilst the Vim forms part of a cerebellothalamocortical loop centred on primary motor cortex. This preferential pattern of thalamic connectivity supports our data which show a circuit-specific pattern of tumour anatomic progression to brain regions with DMG network connectivity independent of primary pontine or thalamic location (both exhibit CM, medial pulvinar, and Vim connectivity).

Preferential involvement of the CM thalamus and medial pulvinar may not only be a function of neural activity within the DMG network but possibly also the inherent “hub-like” network characteristics of these structures, as defined by their cortical connectivity profiles (Behrens … Matthews. *Nat Neuro*. 2003.; Sydnor … Satterthwaite. *Nat Neuro*. 2025). Moreover, the neurodevelopment of the human thalamus occurs in a nucleus-specific manner. Crucially to DMG, differentiation of glial precursors occurs in a medial-to-lateral manner across the thalamus, with greater populations of bipotentent glial progenitors within medial thalamic nuclei later in prenatal and earl postnatal development, including the CM thalamus and medial pulvinar (Kim … Nowakowksi. *Science*. 2023.; Govek … Camara. *Cell Rep*. 2022). The maturation of thalamo-cortical connectivity also occurs along a gradient, with associative circuits involving the CM thalamus and medial pulvinar exhibiting prolonged development (Sydnor … Satterthwaite. *Nat Neuro*. 2025) and potentially reflecting a window of vulnerability to tumour initiation – be that neuronal, neurometabolic, or cellular, that DMG exploits by virtue of its connectivity with these nuclei. Further, recent snRNA-seq data of 149,771 human pons nuclei spanning the first trimester of life to early adulthood demonstrate upregulated synaptic gene expression in healthy pontine early oligodendrocyte lineage cells during early childhood, coinciding with the peak age incidence of DMG, and suggesting an interplay between neural, neurometabolic, and transcriptional vulnerability (Ding … Wedemeyer. *bioRxiv*. 2025.).

Similarly, GBM typically occur within brain network hubs and in relation to adult neural stem-cell and OPC niches (Mandal … Suckling. *Brain*. 2020.). Given the high metabolic cost of maintaining a large number of connections and synapses in hub regions, this observation raises the possibility of neurometabolic influences on gliomagenesis. Indeed, recent work has shown that, whilst GBM exhibit similar glucose uptake to that of healthy cortex, GBM metabolism is hijacked from physiological processes, including tricarboxylic acid (TCA) cycle oxidation and amino acid neurotransmitter synthesis in healthy neurons, towards processes which synthesise nucleotides to fuel tumour growth and therapeutic resistance (Scott … Wahl. *Nature*. 2025.). Further to this, the high neurometabolic load of hub regions, in which gliomas preferentially occur, may confer a state of elevated cellular

stress, providing metabolic conditions which inadvertently favour oncogenesis. These data mirror our finding that peak transitions in neurometabolic activity within the DMG network reflect the spatiotemporal incidence of DMG (**Main Text Fig. 5.**). To directly address the questions raised in the main text, we have specifically included the CM thalamus, medial pulvinar, and ventral intermediate nucleus in figure legends and within the Results section as below:

*"These analyses identified a distributed brain network associated with shorter patient overall survival (**Fig. 1c; Extended Data Fig. 3a**). More specifically, tumours exhibiting greater positive functional connectivity with the pons, **thalamus (centromedian (CM), medial pulvinar, and ventral intermediate nucleus (Vim)),** motor cortex (M1 and supplementary motor area), insular cortex, orbitofrontal cortex, limbic system (amygdala, hippocampus, basal ganglia, cingulate cortex), superior temporal gyrus, and cerebellum (lobules I-V, VIII, and X) were associated with shorter patient overall survival (**Fig. 1d; Supplementary Table 2**)."*

"Fig. 1. Pontine and thalamic DMG map to a conserved, prognostic brain network.
(D) *DMG exhibit a specific pattern of brain functional connectivity associated with patient short-term overall survival. This includes strong positive connectivity with the pons, **thalamus (centromedian, medial pulvinar, and ventral intermediate nuclei)**, motor cortex (M1 and supplementary motor area), orbitofrontal and insular cortices, limbic system (amygdala, hippocampus, basal ganglia, anterior cingulate cortex), and cerebellum (lobules I-V, VIII, and X)."*

We have also added further text to the discussion to enable consideration of these thought-provoking ideas, many of which require further study:

*"The tropism of tumour growth for connectivity peaks in the DMG network further reflects reported, neural activity-dependent directional tumour infiltration described in pre-clinical models of high-grade glioma[9,12,13] and offers an explanation to questions raised by multi-site, whole-brain human whole-exome and whole-genome sequencing studies of post-mortem pontine DMG which define a conserved pattern of tumour anatomic progression insufficiently explained by current models of tumour evolution.[20,21] **To illustrate, we find that the DMG network includes fewer thalamic subregions connected to occipital and parietal cortices and, instead, exhibits peaks in the CM thalamus and medial pulvinar (association nuclei with widespread frontal, insular, cingulate, and somatomotor cortical connectivity[79]) as well as the Vim, which forms part of a cerebellothalamocortical network centred on primary motor cortex.[80,81] This preferential pattern of thalamic connectivity reflects the circuit-specific pattern of tumour anatomic progression to DMG network brain regions independent of primary pontine or thalamic location.** Whilst we show that similar tropism applies to parenchymal intracranial metastases of primary spinal DMG, it remains to be demonstrated if similar network effects are associated with overall survival in these children.*

…

*Neural activity is implicated not only in potentiating the growth of established glioma but also in glioma initiation and maintenance.[80,81] We demonstrate that high network-to-tumour connectivity is associated with the enrichment of gene programmes promoting neuronal character and synaptic integration. Not only this, but high connectivity DMG were relatively enriched for genes upregulated in DMG cells in response to neuronal input, validating DMG network stratification at single-cell resolution. We further show that peak changes in neurometabolic activity across childhood localise to the DMG network, corresponding to the developmental emergence of brain circuits connected to and potentially communicative with DMG. **This observation also raises the possibility of a neurometabolic influence on DMG gliomagenesis: not only does increased neurometabolic activity reflect increased neural activity and maturation, but a developmental period of increased cellular stress during which DMG may acquire secondary, obligate mutations.[82–84] The high neurometabolic demand of these circuits may also reflect the inherent hubness of brain-wide DMG network connectivity, as demonstrated in GBM which typically occur within brain network hubs as well as in relation to adult neural stem-cell and OPC niches.[85] Interestingly, emerging snRNA-seq data of 149,771 human pontine nuclei spanning the first trimester of life to early adulthood demonstrate upregulated synaptic gene expression in healthy pontine early oligodendrocyte lineage cells during early childhood, coinciding with the peak age incidence of DMG, and suggesting an interplay between neural, neurometabolic, and***

transcriptional vulnerability.[86] *Whether such an association between physiologic neurometabolic activity and tumour incidence extends to other cerebral tumours of childhood, many of which share a similar pattern of early childhood peak incidence, remains to be demonstrated and requires a greater understanding of tumour subtype-intrinsic neuron-glioma interactions."*

Without the ability to selectively disrupt neural activity and show this halts tumour growth, the "real-world evidence for neural activity modulating tumour growth" (abstract) may be constrained. However, the data raise fascinating questions about what aspect of specific functional networks or network activity seem so preferred by gliomas.

Thank you for raising this. We have, as previously mentioned, revised some of the language in our manuscript, where appropriate, to better reflect conclusions directly supported by our data. In particular, and as raised by other reviewers, whilst we do not directly measure or manipulate neural activity in relation to tumour growth in children with DMG, nor could we have aimed to do so, we infer neural activity by means of tumour functional connections defined by a normative paediatric connectome in relation to both patient overall survival and the anatomic pattern of tumour growth. We have, therefore, clarified the manuscript main text throughout and have added the following explicit acknowledgement of this limitation to the discussion:

"Further, whilst the role of neuromodulatory neurotransmitters in DMG growth, in particular combinatorial cholinergic, serotonergic, and noradrenergic signalling, remains to be fully described, preclinical reports have revealed the robust effects of brainstem cholinergic neurons and muscarinic signalling on DMG growth and invasion.[5] **Finally, none of the experiments presented herein demonstrate nor could have demonstrated a direct relationship between tumour neural activity and tumour growth in children. Within the framework of causal human brain mapping[42], however, the convergent data presented across multiple modalities are consistent with the hypothesis that DMG network connectivity is associated with DMG tumour growth and patient overall survival.**"

Small queries:

Why was 18 months chosen to classify long-term vs short-term survival given the median survival is typically 8-11 months, and a similar median was present in these cohorts (8-9 months from Fig 2b)?

Early studies reporting long-term survivorship in children with DIPG used a threshold ≥24 months to define long-term survivorship (Hoffman … Fouladi. *J Clin Oncol*. 2018.) whilst some more recent studies have tended to use ≥18 months as a reflection of approximately two times the median overall survival of these children (Roberts … Koschmann. *Acta Neuropathol*. 2023.). Consistent with other lesion network mapping studies (e.g. Joutsa … Fox. *Nat Med*. 2022.), we employ a binary cut-off to define the DMG prognostic network though do recognise that the binary stratification of a continuous measure (overall survival) may prove problematic. To speak to these concerns, we implemented two specific analyses:

1. Replication of tumour network mapping analyses across structural and functional connectivity data using a ≥24 month threshold for long-term overall survival. As shown below visually and quantitatively using label and spin permutation tests, this analysis enabled us to confirm extremely high spatial similarity between 24 month threshold control networks and the 18 month threshold DMG network. We have included these data in our results and **Extended Data Figs. 3j,4j**, as below (fMRI data left; dMRI data right):

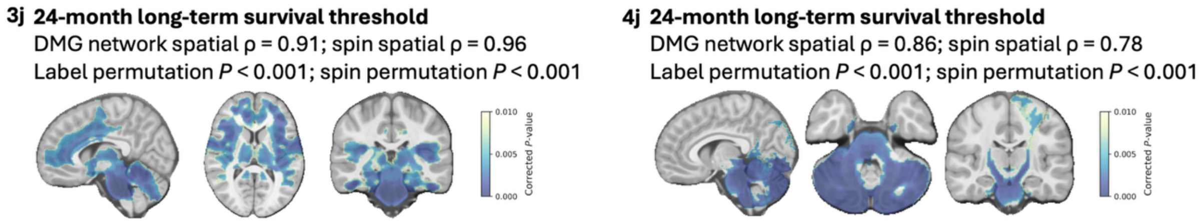

"The DMG network was also reproducible when using both clinically reported cut-offs for DMG patient long-term survival (≥18[32] and ≥24[52] months, respectively; Extended Data Fig. 3a,j, Extended Data Fig. 4a,j) *and with a continuous contrast for patient overall survival, though this captured less of the network at more liberal statistical thresholds (**Extended Data Fig. 3k**, **Extended Data Fig. 4k**)."*

2. Replication of tumour network mapping analyses across structural and functional connectivity data using a continuous outcome measure (overall survival in months). In a similar manner to our previous analysis, continuous maps were spatially compared to 18 month threshold primary result functional and structural DMG networks. However, in this instance, comparisons were made between unthresholded maps, given the lack of statistically significant voxelwise results due to the lower statistical power provided by a continuous survival outcome. As shown below visually and quantitatively using label and spin permutation tests, this continuous analysis enabled us to confirm high spatial similarity between continuous control networks and the 18 month DMG network. We have included these data in our results and **Extended Data Figs. 3k,4k**, as below:

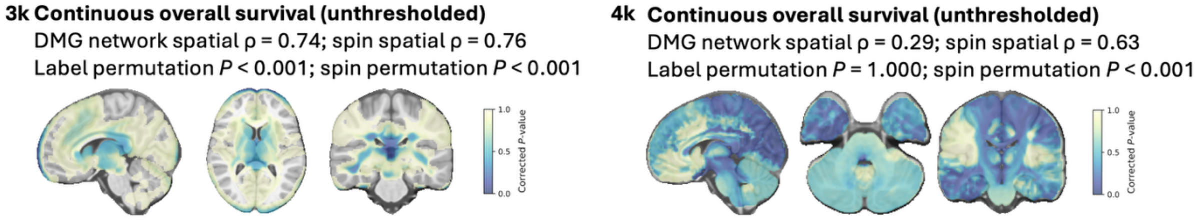

"The DMG network was also reproducible when using both clinically reported cut-offs for DMG patient long-term survival (≥18[32] and ≥24[52] months, respectively; Extended Data Fig. 3a,j, Extended Data Fig. 4a,j) ***and with a continuous contrast for patient overall survival, though this captured less of the network at more liberal statistical thresholds*** *(**Extended Data Fig. 3k**, **Extended Data Fig. 4k**)."*

Fig 1. The sensitivity of the voxel-based tumour location mapping may be enhanced using a multivariate rather than mass univariate approach (arguments put forward by numerous authors, eg Mah et al, Brain 2014 10.1093/brain/awu164). Was there any sub-threshold effect?

Thank you for this important suggestion. To address this, we performed multivariate VLSM in addition to the existing univariate VSLM analyses for tumour location mapping. Multivariate VLSM did not identify any prognostically significant voxels. The subthreshold map ($P_{uncorrected}$>0.005) is visualised in **Extended Data Fig. 1g.** This map was used to stratify external validation cohort patients into 'low' and 'high risk groups by their VLSM map overlap score – i.e. the degree of tumour overlap with multivariate map weights. Kaplan-Meier survival curves identified no significant difference in overall survival between high and low multivariate map score-defined risk groups (**Extended Data Fig. 1i**). This finding was confirmed upon the inclusion of clinical covariates in a multivariable cox model (**Extended Data Fig. 1k**). As alluded to, we have now included the following data in **Extended Data Fig. 1** to reflect these analyses:

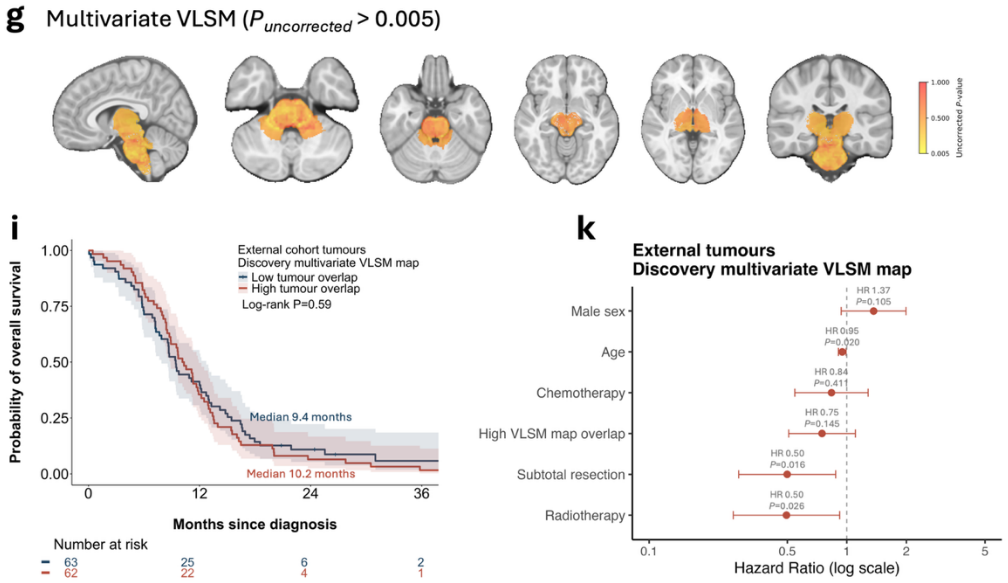

Extended Data Fig. 1g,i-k. Multivariate tumour location mapping.
(G) *Tumour location mapping was repeated across the discovery network with multivariate methods, the results of which did not identify any voxels across pontine or thalamic DMG associated with shorter overall survival ($P_{uncorrected}$>0.005). The overlap of each tumour in an external, independent validation cohort (n=125 children with DMG) was then computed with each unthresholded discovery VLSM map (univariate and multivariate) to generate a VLSM map overlap score per tumour.*
(I) *Kaplan-Meier survival curve of all children in the external validation cohort demonstrates no survival difference between tumours with a high vs. low overlap with the unthresholded discovery cohort univariate VLSM map.*
(K) *Results of a multivariable cox proportional hazards model confirm no prognostic impact of univariate VLSM map overlap.*

The methods have also been revised to report these analyses:

"We replicated these analyses implementing multivariate support vector classification (SVC)-VSLM, which can identify complex dependences between neighbouring voxels that traditional univariate VLSM approaches cannot and based on established best-practice recommendations.[35,36] SVC-VSLM analyses were limited to voxels involved in ≥5% of tumours, controlling for tumour volume and multiple comparisons. Significance was assessed using 10,000 Freedman-Lane permutations. Results of univariate VLSM and multivariate SVC-VLSM analyses were considered significant at two-tailed family-wise error (FWE) corrected P_{FWE}<0.05 and <0.005, respectively, as in previous work.[43]"

In the context of shared co-localisation with the 'DMG network' do you have thoughts what might drive the much lower survival even in the 'low functional connectivity' subgroup of pontine compared to thalamic tumours (Fig 2e vs 2f)?

We thank the reviewer for this insightful question. We have previously described the clinical deterioration of children with pontine DMG as a consequence of increasing brainstem failure (Zanten … Hargrave. *Neuro Oncol*. 2016.). Indeed, brainstem infiltration and subsequent dysfunction are also increasingly recognised markers of progressive GBM (Sahm … Deimling. *JAMA Neurol*. 2012.; Drumm … Horbinski. *Neuro Oncol*. 2020.) – though this takes more time over the disease course due to the supratentorial hemispheric location of most tumours. Similarly, in children with thalamic DMG, longer overall survival may be partly explained by the fact that progression to the brainstem takes longer by virtue of their primary location. We have added a sentence to the discussion to address this, as below:

"Here, we show that pontine and thalamic DMG map to a conserved brain network, connectivity with which is independently and inversely associated with patient overall survival. ***Despite co-localisation within the DMG network, comparable network effects on patient overall survival, and the shared circuit-specific pattern of tumour anatomic progression, overall survival remained significantly shorter for children with pontine DMG, even for those patients with low network-to-tumour connectivity. This may be at least partially explained by our previous description of the clinical deterioration of children with pontine DMG as a consequence of increasing brainstem dysfunction.[79]****"*

Could the data in relation to tumour growth patterns (Fig g) be examined statistically as a percentage / proportion of DMG network (as computed) versus the percent of non-DMG network brain affected?

This is a very good point. We have now implemented this analysis and added an additional subplot to **Main Text Fig. 3b** to reflect this (as below). As hypothesised, tumour volumetric growth within the DMG network was significantly greater than tumour volumetric growth out of the DMG network between diagnostic and final MRI (median 13,768 vs. 1,270 mm^3; 95% CI 11,293-17,763 vs. 773-1,792 mm^3; two-tailed paired Wilcoxon signed-rank test P=6.85e^{-13}). Patient-level tumour growth volumes are shown in grey.

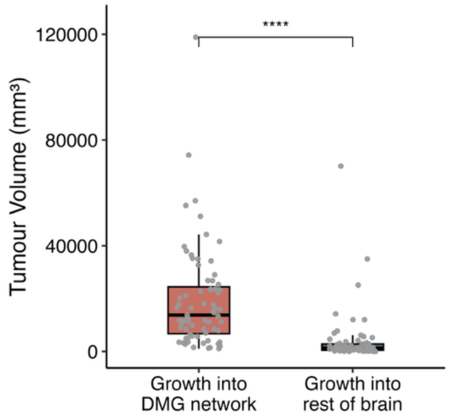

Fig 5 c.d. Do the reported p-values (0.03 and 0.042) reflect correction for multiple comparisons? It would help for visual comparison to match the x-axis scales.

We have revised these analyses to permit the inclusion of new snRNA-seq data. We have ensured that all snRNA-seq analyses include correction for multiple comparisons and that plots have matched axis scales.

I found it sometimes difficult to tease out the number of participants in specific sub-group analyses. It would be helpful to state these when describing key results (examples: long-term vs short-term survivors, numbers with TP53 vs BRAF/FGFR1, how many of the 23 subtotal resection children fell into high vs low network connectivity subgroups, …). Extended Data table 1 would be useful in the primary data.

Thank you for raising this important point. We have expanded the text to include greater rationale for specific analyses, as well as clearer documentation of which patients form part of specific analyses. We have also included **Main Text Fig. 1a** as a methodologic overview (below) and have ensured that figure legends reporting specific analyses contain the specific power of those analyses.

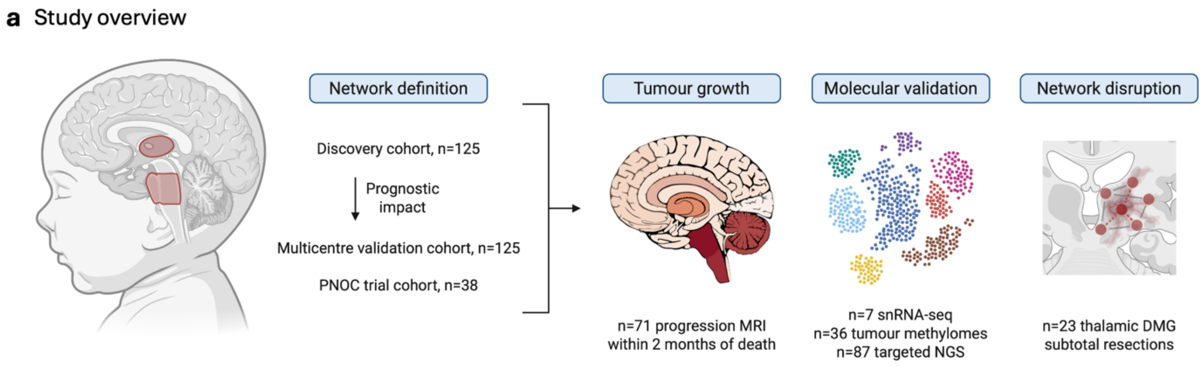

Referee #3 (Remarks to the Author):

I co-reviewed this manuscript with one of the reviewers who provided the listed reports.

Thank you for your review of our work. We have responded to your comments within your co-reviewer's report.

Referee #4 (Remarks to the Author):

N/A – withdrawn.

Referee #5 (Remarks to the Author):

This study evaluated the prognostic value of tumor location and tumor connectivity to distributed brain regions in patients with DMG. While overall survival was not significantly associated with lesion location while controlling for various covariates, there were significant differences in estimates of tumor connectivity between short vs. long-term survivors, which were used to define the "DMG network". Subsequent analyses demonstrate the robustness of the DMG network and highlight its potential prognostic value as a predictor of survival outcomes in patients with DMG. Several follow-up analyses provide evidence to suggest that DMG infiltration/tumor growth preferentially occurs in locations within the DMG network, that neurometabolic changes in the DMG network occur at ages associated with peak thalamic and pontine DMG incidence, that the DMG network's chemoarchitectural profile may make it susceptible to DMG growth, and that tumors with epigenetic neural signatures and/or stem/progenitor cell-like states tend to exhibit greater connectivity to the DMG network. The authors conclude that their results provide real-world evidence of neural activity modulating tumor growth in humans.

I think that overall, this is a relatively strong study that provides important insights into the anatomical/connectomic, metabolic, chemoarchitectonic, and epigenetic properties of DMGs and how they relate to survival outcomes and incidence rates. The study has many strengths, including the use of discovery and validation datasets and the breadth of the research questions addressed. However, there are several aspects that could be improved.

The manuscript is well-written, but it is also extremely dense in terms of analytical content, and it is written in a hyper-abbreviated style that can make the reader feel overwhelmed by the rapid-fire presentation of new and often complex analyses and results. I found that I needed to constantly jump back and forth across the main text, figures, methods, and supplemental material to achieve anything beyond the most abstract/surface-level understanding each analysis, result, and conclusion described in the main text. The figures are also very dense, and they can at times be difficult to parse and/or follow. Some of the data visualizations take up a lot of space but are not very informative and feel like they should be moved to the supplemental material (e.g. Figure 3, A-C), while others are simple-but-unintuitive and would likely be better represented using "conventional" plots (e.g. Figure 4, H-G). I think the paper would easier to follow if results were spread across more figures with fewer panels, with the goal of ensuring that the included panels feature thorough representations of the primary results. For example, Figure 1 takes up nearly a whole page to show (a) a generic representation of the analysis methods in the discovery cohort (i.e. panels A and B), and (b) 3 slices of the main functional network result, while the main the structural DMG network result is inexplicably relegated to the supplement.

While the methods generally seem sound, there are several key details that are very difficult to ascertain from the manuscript, and these details could have potentially substantial implications for the validity and/or interpretation of several key results. For example, it is unclear whether models were trained in the discovery dataset and applied to the validation dataset vs. trained in the validation dataset using features from the discovery dataset, and there appears to be some circularity to the modeling analyses of patients with thalamic vs. pontine DMG.

There are also several methodological choices that would seem to predispose some analyses to fail. For example, they use a continuous survival outcome for the tumor location analysis and covary multiple factors, including tumor location. Covarying for lesion location in an analysis that attempts to relate outcome to lesion location seems counterintuitive. It is not clear why the authors would use this approach for the lesion analysis and use a categorical survival outcome with no covariates for the primary network analyses, especially since the continuous survival outcome produces much weaker results for the network analyses in the supplemental material. The "network sensitivity" analyses are also performed in a way that predisposes them to produce low correlations between the network templates in the cerebral cortex and the DMG network map that is primarily subcortical. An unthresholded brain-wide map would seem more appropriate for evaluating the target network against the Yeo networks. Several conclusions also appear to rest purely on qualitative/descriptive results, although quantitative analyses that could explicitly support the conclusions should be possible (e.g. "network sensitivity analyses", tumor growth analyses, etc.).

Based on these limitations in the current version I recommend a Major Revision with re-review.

I provide detailed comments and suggestions below.

Thank you for your positive comments and detailed recommendations. We have re-written a significant proportion of the manuscript to provide more methodologic clarity in the main text. We have also revised and simplified all figures to ensure a clearer and statistically valid presentation of the main findings, all of which are now offered with quantitative support. These changes, in combination with the inclusion of additional verification and control experiments, have greatly strengthened the submitted work for which we are grateful.

Major Comments

1. The main focus of the paper is on elucidation of the DMG network, yet visualization of the DMG networks is generally poor. The figures give the impression that the anatomical findings lack specificity, essentially encompassing the brainstem and thalamus. It would be good to include more comprehensive visualizations of the structural and functional networks since they are the main results of the paper on which everything else rests. The authors could explore whether a higher threshold would help for display purposes in demonstrating key nodes of this network.

We thank the reviewer for this valuable comment. We agree that visualisation of the DMG network should be improved and would enhance the presented findings. To this end, we have revised **Main Text Fig. 1** to include three-dimensional axial slices through the DMG network as well as sagittal and coronal slices through the brainstem. We have also included a visualisation of DMG network peaks, defined using FSL cluster, to provide better anatomic specificity. Peak cluster centre of gravity co-ordinates and voxel size are also provided in **Supplementary Table 2**.

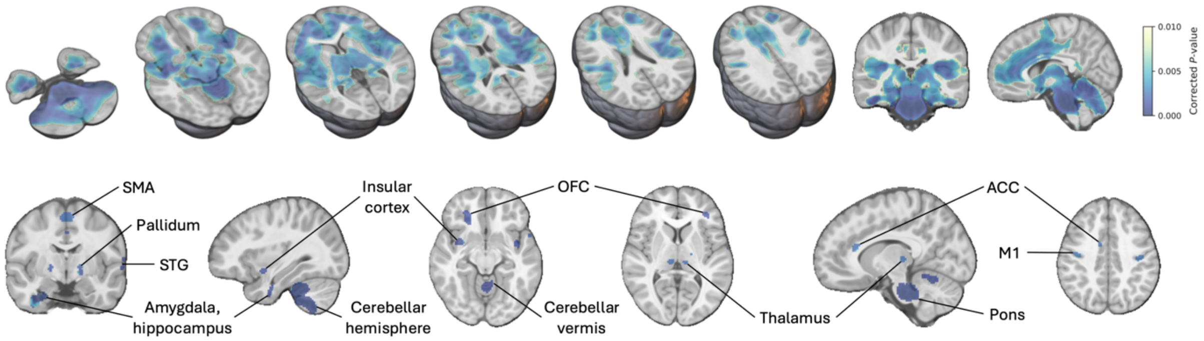

Main Text Fig. 1d.
DMG exhibit a specific pattern of brain functional connectivity associated with patient short-term overall survival. This includes strong positive connectivity with the pons, thalamus (centromedian, medial pulvinar, and ventral intermediate nuclei), motor cortex (M1 and supplementary motor area), orbitofrontal and insular cortices, limbic system (amygdala, hippocampus, basal ganglia, anterior cingulate cortex), and cerebellum (lobules I-V, VIII, and X).

	Centre of gravity coordinates			P_{FWE}	Voxels (n)	Cluster location(s)
	x	y	z			
**Cluster 1**	-2.55	-41.20	-40.60	P≤0.0005	6639	Brainstem, cerebellum
**Cluster 2**	-2.27	-55.70	-12.60	P≤0.0005	324	Cerebellar vermis
**Cluster 3**	-63.50	-0.60	2.54	P≤0.0005	122	Left superior temporal gyrus
**Cluster 4**	23.30	34.20	-11.00	P≤0.0005	102	Right orbitofrontal cortex
**Cluster 5**	33.00	-5.98	-35.60	P≤0.0005	93	Right hippocampus
**Cluster 6**	35.50	1.21	-15.80	P≤0.0005	77	Right amygdala, right insular cortex
**Cluster 7**	-36.40	36.20	8.01	P≤0.0005	65	Left orbitofrontal cortex
**Cluster 8**	-39.20	0.15	-14.60	P≤0.0005	60	Left amygdala, left insular cortex
**Cluster 9**	-19.40	-12.20	-1.02	P≤0.0005	75	Left pallidum
**Cluster 10**	-42.00	-18.10	39.30	P≤0.0005	173	Left precentral gyrus
**Cluster 11**	8.70	-24.00	5.79	P≤0.0005	109	Right thalamus
**Cluster 12**	18.20	-8.84	0.01	P≤0.0005	35	Right pallidum
**Cluster 13**	18.40	-28.50	59.70	P≤0.0005	21	Right precentral gyrus
**Cluster 14**	-1.03	-7.79	59.60	P≤0.0005	93	Right and left supplementary motor area
**Cluster 15**	6.79	-1.08	42.30	P≤0.0005	37	Right cingulate cortex
**Cluster 16**	-26.60	-36.40	-3.43	P≤0.0005	29	Left hippocampus
**Cluster 17**	-10.30	27.80	17.50	P≤0.0005	21	Left cingulate cortex
**Cluster 18**	-10.80	-25.20	4.26	P≤0.0005	20	Left thalamus

Supplementary Table 2.
*Significant clusters in the DMG network. Clusters are illustrated spatially in **Main Text Fig. 1d**. Coordinates are reported in MNI space (mm) with cluster sizes in voxels (1.0 x 1.0 x 1.0mm). Several clusters included more than one neuroanatomic location, listed under cluster location(s).*

More broadly, throughout the manuscript, we have worked to improve the visualisation of presented networks and have ensured their quantitative comparison to one another, where appropriate. An exception worth noting is visualisation of the structural DMG network which continues to be presented in **Extended Data Fig. 4**. Our rationale for this is that, despite demonstrating reproducibility to methodological choices and covariates, structural network-to-tumour connectivity was non-prognostic across both external validation datasets. We discuss this finding and its implications in response to a later comment by the same reviewer.

2. The tumor location mapping approach evaluated the association between tumor location and overall survival using voxel-wise linear regressions. However, the primary network-mapping approach compared voxel-wise tumor connectivity estimates between short- and long-term survivors of DMG. Why were different primary outcomes used for the tumor location mapping vs. network mapping analyses? Extended Data Fig. 5 indicates that when continuous overall survival was used as the outcome for network mapping, the analysis failed to define the entire network even at more liberal statistical thresholds, which the authors interpret as reflecting reduced statistical power using this outcome variable. This suggests that the failure of the tumor location mapping approach could be at least in part due to its use of the continuous overall survival outcome variable, and that the implicit comparison between tumor location vs. network approaches may be biased against tumor location by the analysis strategy. There is not an obvious reason to use a continuous version of the outcome variable and include covariates for one analysis while using a categorical version of the outcome variable with no covariates for the other. The primary tumor location mapping analyses should use the same outcome and modeling strategy as the primary network mapping analyses. To properly compare these two, I would recommend voxel-wise logistic regressions with the same covariates for analyses of both tumor locations and networks, with both analyses using the same categorical outcome variable.

Thank you for raising this inconsistency. To clarify, we did perform tumour location mapping between binarised short- and long-term survival patient groups rather than using continuous survival outcomes in these analyses but incorrectly termed this 'regression' in our original manuscript Methods. Please accept our apologies for this confusion. Further, we agree that we should have standardised modelling strategies between tumour location mapping and tumour network mapping as far as possible. To speak to these concerns, we have revised the Methods text to reflect our use of a univariate voxel-wise Liebrmeister test between short- and long-term tumours for primary lesion location mapping analyses, as below:

"Identifying tumour voxels associated with patient overall survival
Tumour location frequency maps were plotted in MNI space as the sum of all binary tumour masks using ANTs iMath functions. ***To establish whether tumour anatomic location alone was associated with patient overall survival, we first implemented a univariate voxel-based lesion symptom mapping (VLSM) approach. A voxel-wise Liebermeister test was performed to compare voxels between short- and long-term survival groups in NiiStat (https://github.com/neurolabusc/NiiStat).[34,43] Analyses were limited to voxels involved in ≥5% of tumours, controlling for tumour volume and multiple comparisons.[33,35,36,100]*** *Significance at each voxel was assessed using 2000 Freedman-Lane (shuffled) permutations. Given the significantly shorter overall survival in children with pontine versus thalamic DMG, we also performed univariate VLSM across pontine and thalamic DMG separately to control for the prognostic effect of primary tumour location."*

As stated in the amended Methods section above, and with thanks to the reviewer's careful comment, we also re-performed these analyses using the exact same modelling strategy as for tumour network mapping – namely, using only tumour volume as a covariate (**Extended Data Fig. 1**). The results of this analysis identified a region spanning the entire pons as significantly associated with shorter overall survival. We interpret this as being due to the well-described difference in overall survival between children with primary pontine vs. thalamic DMG (Mackay … Jones. *Cancer Cell*. 2017.) and the clinical deterioration of children with pontine DMG as a consequence of increasing brainstem dysfunction, as described by our group (Zanten … Hargrave. *Neuro Oncol*. 2013.). Given this, and analogous to the manner in which tumour network mapping analyses were performed, we performed tumour location mapping for pontine and thalamic DMG separately to answer whether there is a

specific location within the pons or thalamus that drives shorter survival outcomes for pontine and thalamic DMG, respectively. In doing so, we did not identify any statistically significant pontine or thalamic tumour voxels associated with overall survival for pontine or thalamic DMG, respectively. Included unthresholded pontine and thalamic VLSM maps also enable direct comparison with pontine and thalamic DMG networks (**Extended Data 1.d-f.**).

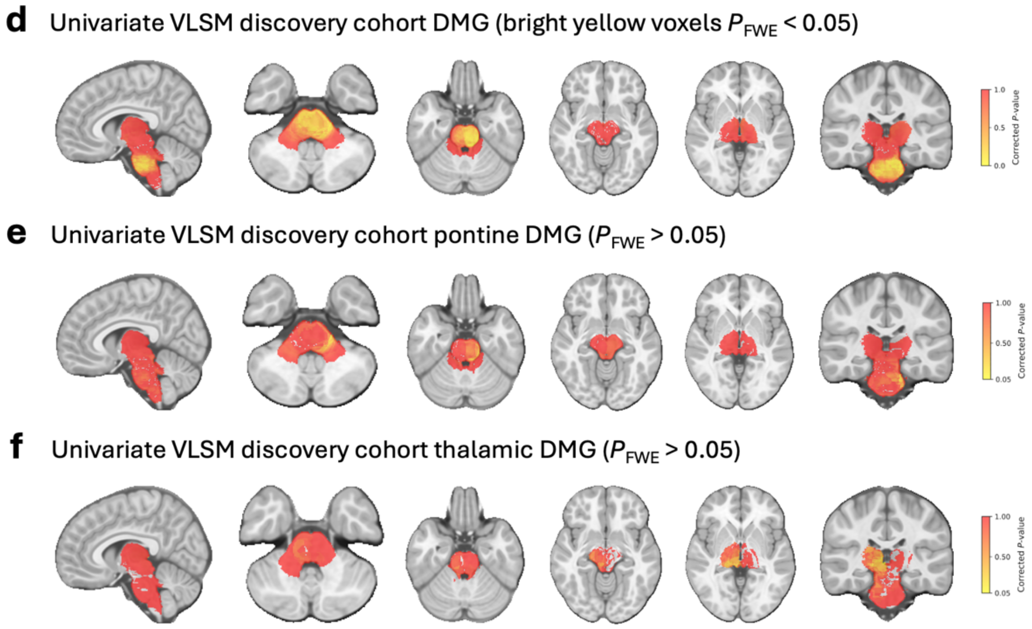

Extended Data Fig. 1d-f. DMG tumour location mapping by voxel-based lesion symptom mapping (VLSM).
Results of mass-univariate lesion location mapping, restricted to at least 5% of overlapping voxels between tumours, across DMG in the discovery cohort (D) expectedly revealed that tumour location within any pontine voxel was associated with shorter overall survival (P_{FWE}<0.05). When analyses were repeated restricted to pontine (E) and thalamic (F) DMG, respectively, no voxels significantly associated with shorter overall survival were identified (P_{FWE}>0.05).

3. Related to the above comment, there is an implicit comparison between the prognostic value of tumor location and tumor network information based on the presence vs. absence of significant effects in the corresponding voxel-wise analyses. However, this is not a statistically valid method for determining if one feature (i.e. network connectivity) is more prognostically valuable than another (i.e. tumor location). It would be more compelling to perform an explicit comparison of the prognostic value provided by tumor location vs. network information. This could be achieved in several ways, for example, by constructing multivariate, data-driven tumor location and network models in the discovery dataset and then directly comparing their performance at predicting survival outcomes via cross-validation and/or via application to the independent validation dataset. This would allow for principled comparisons of tumor location and network information and support explicit conclusions about the relative prognostic value of each type of tumor feature. Related to this topic, there are clear prognostic implications of the DMG connectivity of tumor location. Analyses that may help the clinically oriented reader ascertain how robust of a predictor the connectivity values are could include R2 (predicted versus actual survival months) or accuracy for out of sample predictions binning groups into high or low survival groups.

We thank the reviewer for this important follow-on comment. As discussed in response to the above, primary pontine rather than thalamic tumour location is well-associated with shorter patient overall survival (Mackay … Jones. *Cancer Cell*. 2017.). We formally corroborate these results with our univariate VLSM across both pontine and thalamic DMG (Extended Data Fig. 4d) and this was the rationale for why we corrected for primary tumour location in our original tumour location mapping analyses – in which we asked whether there were specific voxels within the pons or thalamus associated with patient overall survival for pontine DMG and thalamic DMG only, respectively. These failed to identify any locations within these structures associated with shorter patient overall survival (***Extended Data Fig. 1e-f.;*** as above).

To speak to the reviewer's question, we have included additional analyses to directly interrogate the effect of tumour location on patient overall survival. Methodologically, we tested this is an analogous manner to that of computing network-to-tumour connectivity: i.e. subthreshold maps for both univariate and multivariate VSLM analyses were used to stratify external validation cohort patients into 'low' and 'high risk groups by their VLSM map overlap score – i.e. the degree of tumour overlap with multivariate map weights. Kaplan-Meier survival curves identified no significant difference in overall survival between high and low VLSM score-defined risk groups: findings which were confirmed upon the inclusion of clinical covariates in a multivariable cox model (**Extended Data Fig. 1h-k**).

We have now detailed these analyses in the tumour location mapping methods:

"We replicated these analyses implementing multivariate support vector classification (SVC)-VSLM, which can identify complex dependences between neighbouring voxels that traditional univariate VLSM approaches cannot and based on established best-practice recommendations.[35,36] SVC-VSLM analyses were limited to voxels involved in ≥5% of tumours, controlling for tumour volume and multiple comparisons. Significance was assessed using 10,000 Freedman-Lane permutations. Results of univariate VLSM and multivariate SVC-VLSM analyses were considered significant at two-tailed family-wise error (FWE) corrected P_{FWE}<0.05 and <0.005, respectively, as in previous work.[43]

Interrogating the impact of tumour VLSM map scores on patient overall survival
The resulting univariate and multivariate VLSM maps represented unified, group-level maps of the prognostic strength of each voxel across overlapping DMG. We interrogated whether VLSM maps could determine patient overall survival based on tumour location alone, irrespective of the presence or absence of voxels significantly associated with overall survival in the discovery cohort, using data from an independent, multicentre external validation cohort, with identical inclusion criteria and identical methodology for tumour segmentation and brain MRI pre-processing. Each binary tumour segmentation in the external validation cohort was weighted by overlapping voxels in each VLSM map. A mean 'tumour VLSM map score' was then computed as the average of these weighted values for univariate and multivariate analyses, respectively.[39] Patients were stratified into two risk groups using a median cut-off for each VLSM map: high risk (defined as median or above-median tumour VLSM map score) and low risk (defined as below-median tumour VLSM map score). Kaplan-

Meier models were then used to model overall survival for each VLSM map score-defined risk group, with the difference between groups evaluated using a two-tailed log-rank test. We also interrogated whether tumour VLSM map score could act as an independent predictor of patient overall survival via inclusion as a variable in both univariable and multivariable cox proportional hazards models, correcting for patient demography (age at diagnosis and sex) and clinical management (extent of resection, and completion of adjuvant radiotherapy and chemotherapy). In all instances, the proportional hazards assumption was verified prior to analysis and results were expressed as hazard ratios with 95% confidence intervals (CI) and corresponding P values."

Further, we have included their results in the manuscript main text, as below and in **Extended Data Fig.1**, with data shown overleaf:

"**Finally, we performed multivariate VLSM across all tumours, which considers the dependencies between voxels rather than treating each voxel in isolation, and which also failed to identify specific voxels associated with patient short-term survival (Fig. 1b, Extended Data Fig. 1g). To test the prognostic impact of subthreshold VLSM results (defined as weak and non-significant voxelwise associations with patient short-term survival), we collected data from an independent cohort of children aged <18 years with primary pontine (n=80) or thalamic (n=45) DMG and identical inclusion criteria treated at one of three international collaborating centres or included in the HERBY clinical trial (NCT01390948[38]; Supplementary Table 1). The weighted overlap of each tumour in this external validation cohort with our defined univariate and multivariate VLSM maps, respectively, was computed and averaged to generate an overlap score in line with methods previously described.[39] Patient tumours stratified into high- and low-overlap risk groups using a median cut-off exhibited no difference in overall survival across both univariate and multivariate VLSM tested using the Kaplan-Meier method (Extended Data Fig. 1h-i). The non-prognostic impact of tumour-VLSM map overlap was further confirmed by univariable and multivariable cox proportional hazards models including clinical covariates (age, sex, extent of resection, and completion of adjuvant radiotherapy and chemotherapy; Extended Data Fig. 1j-k).** Given the influence of neural activity on DMG progression in murine models[3–8], and the fact that human DMG exhibit a common pattern of brain infiltration (**Extended Data Fig. 2**), we hypothesised that **tumour connectivity with an underlying brain network would better explain the observed variance in** patient overall survival."

As suggested, we also constructed additional multivariable cox proportional hazards models to directly compare the prognostic value of DMG functional / structural network and tumour location information. To enable more detailed discussion of network results, we report these results and discuss their importance in response to Major Comment 10 by the same reviewer.

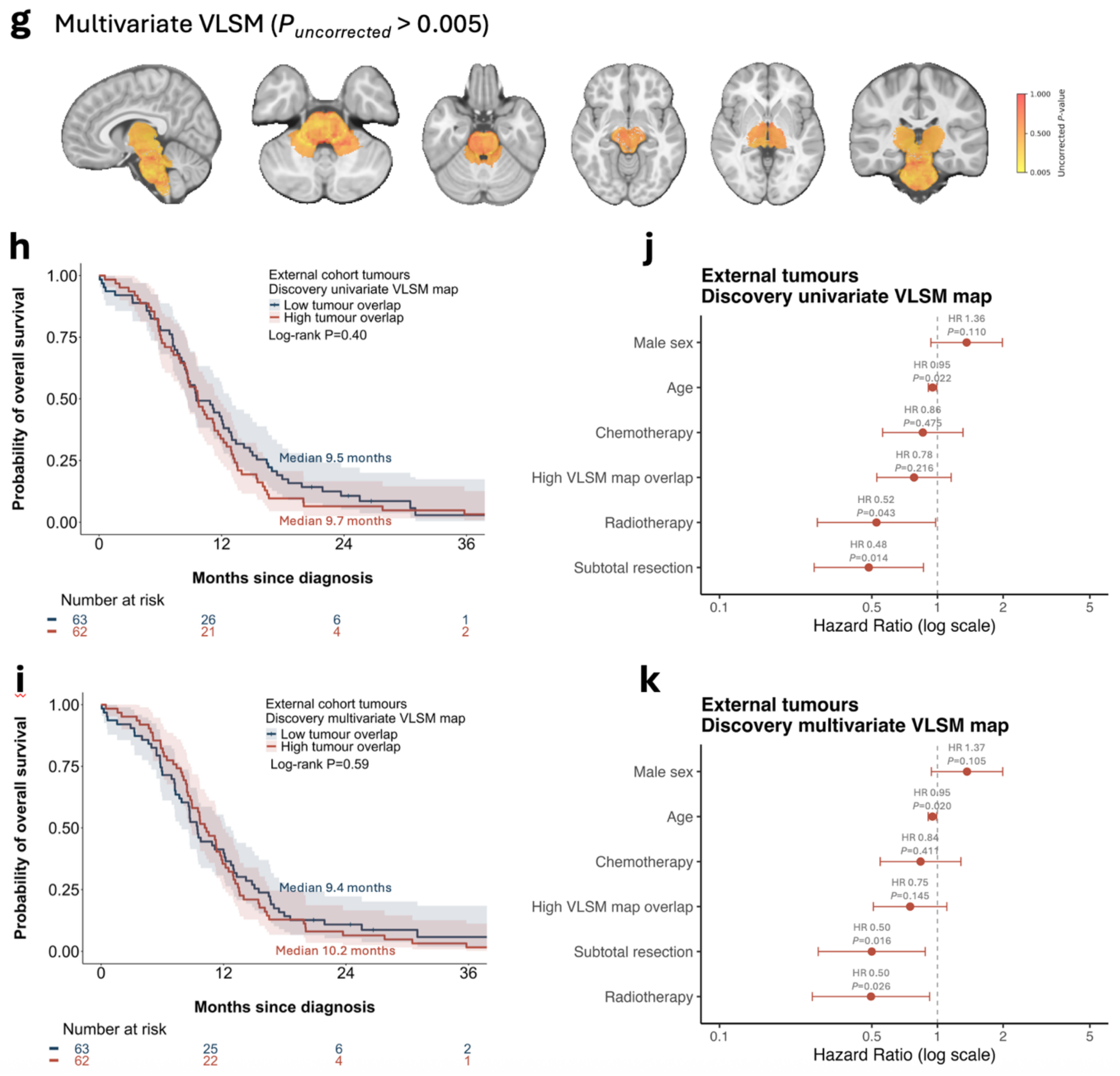

Extended Data Fig 1g-k. DMG tumour location mapping by voxel-based lesion symptom mapping (VLSM). *Tumour location mapping was repeated across the discovery network with multivariate methods, the results of which did not identify any voxels across pontine or thalamic DMG associated with shorter overall survival **(G**; $P_{uncorrected}$>0.005). The overlap of each tumour in an external, independent validation cohort (n=125 children with DMG) was then computed with each unthresholded discovery VLSM map (univariate and multivariate) to generate a VLSM map overlap score per tumour. **(H-I)** Kaplan-Meier survival curves of all children in the external validation cohort demonstrate no survival difference between tumours with a high vs. low overlap with the unthresholded discovery cohort univariate or multivariate VLSM map. **(J-K)** Results of multivariable cox proportional hazards models confirm no prognostic impact of univariate and multivariate VLSM map overlap.*

The comment regarding the predictive power of network-to-tumour connectivity is addressed fully in Major Comment 6. In brief, we performed five-fold stratified cross-validation across the external validation cohort, calculating network-to-tumour connectivity on the discovery cohort DMG network. Within each fold, network-to-tumour connectivity values from 40% patients (n=50) were computed and used to define a median connectivity cut-off value which was then applied to a multivariable cox proportional hazards model across the remaining, held-out patients (60%; n=75). Median connectivity cut-off values remained stable when patients were randomly shuffled within training sets across folds. Corresponding hazards ratios and concordance indices demonstrated consistent, independent, and accurate prediction of patient overall survival (median hazard ratio 3.33; median concordance index 0.70). These results are now presented in **Main Text Fig.2e-g.**, as below. We did not perform similar analyses for tumour location given the poor performance of VLSM overlap scores, even at the univariate level.

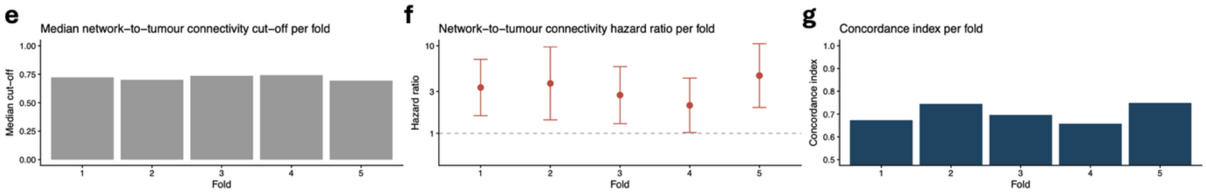

Main Text Fig. 2e-g. DMG-network-to-tumour connectivity predicts overall survival in children with DMG.
(E-G) *Results of five-fold stratified cross-validation demonstrate a stable cut-off for median network-to-tumour connectivity* ***(E)****; significant hazard ratios across all folds (mean hazard ratio 3.33)* ***(F)****; and accurate prediction in out-of-sample data (median concordance index 0.70)* ***(G)****. In all forest plots, mean values are represented by solid circles and whiskers represent 95% CI.*

4. The "sensitivity" analyses utilizing the 7 Yeo networks are not very compelling (Extended Data Fig. 7). The network maps were binary masks from the cortical parcellation atlas, while the network mapping results were continuous maps that (per the methods) do not appear to have been explicitly restricted to voxels within the cortical parcellation and that strongly feature subcortical/cerebellar structures. Comparing un-masked continuous statistical maps that include robust contributions from subcortical and cerebellar voxels to binary masks that are restricted to cortical parcels will artificially deflate correlations even between similar networks. It would make more sense to, for example, to use the Yeo network masks as seeds in the same rsfMRI dataset used to generate the tumor FC maps, and then compare the continuous whole-brain networks (i.e. including subcortical and cerebellar voxels) against the DMG network. Even then, though, it is unclear what a reasonable similarity threshold should be for determining whether the DMG is truly distinct from the 7 Yeo networks. Fundamentally, these analyses seem to be purely descriptive, and the rationale for how they demonstrate network sensitivity is unclear. The unthresholded, unmasked view of the DMG shown in Extended Fig.8 also suggests that the DMG spans the vast majority of the brain; is it possible that the DMG is just a union of several canonical networks? I think that these analyses should be revised, and that rationale for how they support the conclusions should be clearly stated in the manuscript.

Thank you for highlighting the flaw in this analysis and, more broadly, for challenging their conceptual purpose. First, we agree that comparing the DMG network to binary masks of Yeo canonical network regions is not a robust analysis to test for spatial similarity of the DMG network to each of the Yeo networks. Furthermore, given that the DMG network represents the confluence of multiple brain regions contributed by several Yeo canonical networks, as the reviewer notes and as shown for other disease-relevant human brain networks (Siddiqi … Fox. *Nat Neuro*. 2024.; Ji … Schaper. *Nat Commun*. 2025), we also agree that quantifying the spatial similarly of each Yeo map to the DMG network and testing the prognostic value of each in patients with DMG is not particularly useful. We have, therefore, removed these supplementary analyses from the manuscript.

Importantly, this comment also caused us to carefully consider the preclinical work upon which our study is based: namely, that the otherwise healthy brain forms neuron-to-glioma synapses with DMG cells, activity from with promotes tumour growth (Venkatesh … Monje. *Nature*. 2019.). In no way do we mean to suggest that the DMG network is more than a prognostic map of these hijacked brain-wide connections specific to this tumour type. Specificity analyses should, therefore, hinge on the latter aim: namely, to demonstrate that the connectivity of other high-grade glioma with the DMG network is non-prognostic for that tumour type. To model this, we turned to adult patients with GBM, a tumour type with similarly overwhelming clinical and preclinical evidence for neuron-glioma synaptic integration – many of the mechanisms of which are conserved with DMG (Osswald … Winkler. *Nature*. 2015.; Venkataramani … Winkler. *Nature*. 2019.; Venkataramani … Winkler. *Cell*. 2022.; Hausmann … Winkler. *Nature*. 2023.). We then collected two independent cohorts of patients with GBM, *IDH*-WT: (1) the open access UCSF-PDGM dataset (n=219; Calabrese … Hess. *Radiol Artif Intell*. 2022.) and (2) a newly generated dataset from Queen Square (n=272). GBM patient tumours were segmented by board-certified neuroradiologists and tumour network mapping performed. Patient-wise GBM tumour connectivity with the DMG network was computed, using identical methods to those for children with DMG, and identical survival analyses performed: first, using the Kaplan-Meier method, and second, using a multivariable cox proportional hazards model with the inclusion of relevant clinical covariates. Across both cohorts and survival analysis methods, GBM connectivity with the DMG network was non-prognostic, demonstrating that the DMG network uniquely predicts the overall survival of children with DMG – i.e. network specificity.

We have revised the manuscript methods section to reflect this, as below:

*"**Specificity testing of DMG network prognostic impact***
Finally, to ensure the prognostic specificity of the DMG network, the impact of DMG network-to-tumour connectivity on overall survival was assessed against across two independent cohorts of adult patients (aged >18 years) with glioblastoma (GBM), IDH-WT: (1) a previously reported, open access 219 patient cohort from the University of California, San Francisco (UCSF-PDGM)[68] and (2) a newly generated institutional 272 patient cohort from the National Hospital for Neurology and Neurosurgery (NHNN), Queen Square, London. Previously generated tumour segmentation masks in the UCSF-PDGM data were manually checked

against available T2-FLAIR sequences whilst tumour segmentation masks for patients at NHNN were manually segmented from all available MRI sequences at diagnosis per patient, as previously described. GBM locations were used as seeds in the ABCD1000 connectome[40] to obtain brain-wide maps of tumour functional connections across both cohorts, enabling the computation of DMG network-to-tumour connectivity per patient. Survival analyses were replicated using identical methods and with the inclusion of clinical covariates which are reported to influence the overall survival of adult patients with GBM, IDH-WT: patient demography (age at diagnosis and sex); O^6-methylguanine-DNA methyltransferase (MGMT) promoter methylation; and clinical management (extent of resection, and completion of adjuvant radiotherapy and chemotherapy)."

We have also included these data in the manuscript main text and in **Extended Data Fig. 6g-h.**, as below:

"***Finally, we hypothesised that the DMG network, i.e. the pattern of prognostic brain network connections in children with DMG, would specifically predict the overall survival of children with DMG rather than other patients with high-grade glioma. This hypothesis mirrors recent preclinical evidence for distinct, tumour subtype-specific mechanisms of neuron-glioma interaction.[6,66,67] To speak to this, we collected and analysed two independent cohorts of adult patients with glioblastoma (GBM), IDH-WT: (1) a previously reported cohort of 229 patients included in the open access UCSF-PDGM dataset[68] and (2) an institutional cohort of 272 patients treated at the National Hospital for Neurology and Neurosurgery, London (Supplementary Table 3). Across both cohorts, GBM connectivity with the DMG network was not associated with or predictive of patient overall survival (Extended Data Fig. 6g-h). Taken together, these findings demonstrate that, despite disparate anatomic origins, tumour connectivity with the DMG network is independently and specifically predictive of overall survival in children with pontine and thalamic DMG.***"

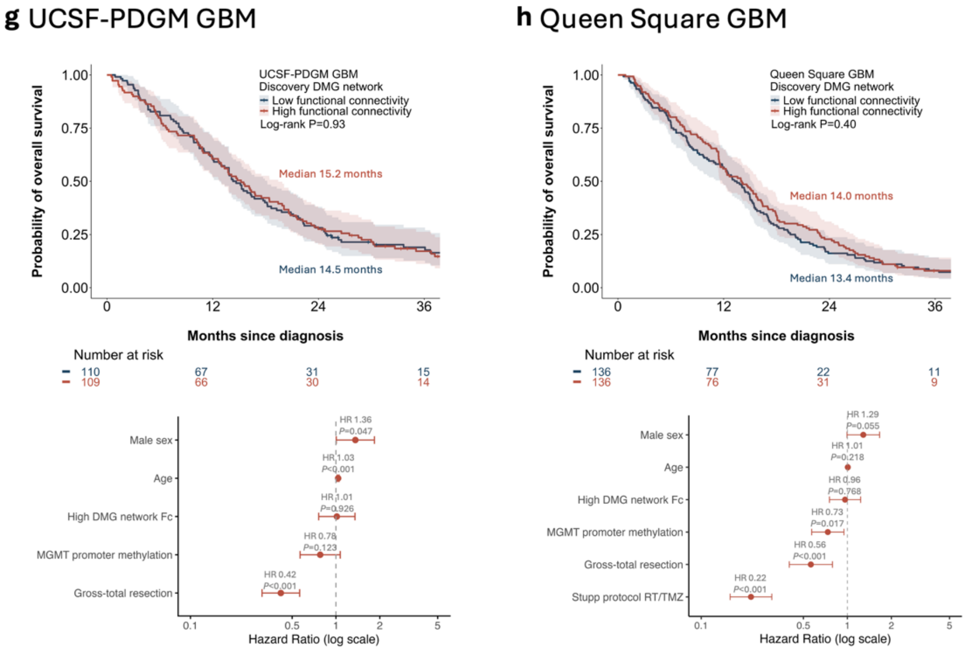

Extended Data Fig. 6g-h. The prognostic power of the DMG network is specific to DMG.
(G-H) *Kaplan-Mieier survival curves and multivariable cox proportional hazards models demonstrate that GBM connectivity with the DMG network is not prognostic across two validation datasets and that, therefore, the DMG network is specific to DMG.* ***(G)*** *UCSF-PDGM. Kaplan-Meier median overall survival 14.5 vs. 15.2 months; 95% CI 12.8-18.2 vs. 13.0-19.4 months; P=0.930. Multivariable cox HR 1.01; 95% CI 0.76-1.35; P=0.281.* ***(H)*** *Queen Square. Kaplan-Meier median overall survival 13.8 vs. 14.0 months; 95% CI 11.8-15.2 vs. 11.6-16.2 months; P=0.40. Multivariable cox HR 0.96; 95% CI 0.75-1.23; P=0.768. In all Kaplan-Meier curves, the statistical difference between groups was evaluated using the log-rank test with error bars representative of 95% CI. In all forest plots, mean values are represented by solid circles and whiskers represent 95% CI.*

5. There is no attempt to quantify the correspondence between the structural/functional DMGs derived from the discovery vs. validation cohorts. A spin permutation test could be used to explicitly test for statistically significant similarity between the discovery and validation cohort maps. The same could be done for the thalamic-DMG vs. pontine-DMG maps in Figure 2. Most claims about similarity (or lack thereof) in the manuscript are based on purely descriptive or qualitative comparisons.

We thank the reviewer for this valuable suggestion. We have now implemented spin permutation tests throughout the manuscript to ensure that previously qualitative claims are now supported by quantitative evidence. Further, as the DMG network is a predominantly subcortical network, we performed further label permutation testing throughout. In all instances, both results are provided with corresponding spatial similarity values. Specifically, we tested the correspondence between:

1. The discovery cohort DMG network and the external validation cohort network / PNOC trial cohort network (**Main Text Fig. 1d-f.)**.
2. The DMG functional network and all control networks, including pontine and thalamic networks (**Extended Data Fig. 3a-l.**).
3. The DMG structural network and all control networks, including pontine and thalamic networks (**Extended Data Fig. 4a-l.**).
4. The DMG network defined using the ABCD1000 functional connectome and the GSP1000 and Yeo1000 functional connectomes (**Extended Data Fig. 5a-c.**).
5. The DMG network and neurometabolic peak change maps at each childhood age (**Supplementary Table 6**).

To reflect these changes, we have added the following text to the results and methods, respectively:

Results:

"In all instances, the similarity of control and validation networks to the DMG network was assessed against a null distribution of 10,000 networks using two methods: (1) label permutation testing[46,56], in which each patient's functional connectivity map was randomly paired with another patient's overall survival value to generate each null network, and (2) spin permutation testing[56,57], in which the DMG network was parcellated and randomly rotated to generate each null network."

Methods:

"The correspondence in brain-wide spatial topography between the DMG network and each control network was assessed using both label permutation[47,57] and spin permutation[57,58] tests. Firstly, the whole-brain spatial Spearman correlation, or 'spatial similarity', between each control network and the DMG network was compared against a null distribution of 10,000 permuted network results generated by randomly permuting short- and long-term survival labels 10,000 times across patients in the discovery cohort, yielding an empiric P value reflecting the significance of each observed spatial similarity. Next, spatial autocorrelation-preserving permutation testing, or 'spin testing', was achieved by parcellating the DMG network into paediatric MINCI 60MB SynthSeg masks[108] and randomly rotating the spherical projections of the parcellated network 10,000 times. Each control network was parcellated into the same brain regions and its spatial similarity to each randomly rotated DMG network computed. The P value for each spin test was computed as for each label permutation test. Although spin permutation tests are reported to inflate P values and output more liberal statistical estimates due to preserved spatial autocorrelation between null models[57,109], both spin and label permutation tests were performed and reported, given the widespread use of spin testing."

6. Were the cox proportional hazards models trained in the discovery dataset and then applied to the validation dataset to obtain true estimates of out-of-sample prediction accuracy, or were tumor-DMG connectivity estimates in the validation dataset used to train an inferential model in the validation dataset? This is important for interpreting results like the prediction of patient overall survival (78%, 89% with covariates) since it could still be optimistically biased (e.g. due to overfitting) if the models were trained in the validation dataset using estimates based on features identified in the discovery dataset. To make claims about prediction accuracy in the validation dataset, it would be necessary to train the models in the discovery dataset and then apply them directly to the discovery dataset, using classification cut-offs defined in the discovery dataset. Please clarify which dataset was used to train the models and define the classification cut-offs, and whether these are true out-of-sample prediction analyses vs. analyses demonstrating that models fit to features identified in the discovery dataset produce statistically significant effects when included in models trained in the validation dataset, and revise claims about prediction accuracy if necessary.

Thank you for bringing this to our attention. In all instances, we used a discovery dataset to define a network and a second, independent and held-out dataset to evaluate the prognostic importance of that network. However, as the reviewer correctly notes, to test true prediction, we should define the network using dataset (A); define a cut-off on that network using dataset (B); and test prediction using that cut-off on dataset (C). We use the concordance index as a measure of predictive accuracy for time-to-event data, in keeping with previous work (Tam … Mattonen. *Neuro Oncol Adv*. 2021.). We have now implemented this approach and have documented this in the methods, as below:

"To enable testing of true prediction of patient overall survival based on DMG network-to-tumour connectivity, we performed stratified five-fold cross-validation.[39,111] Within each fold, network-to-tumour connectivity values were computed across 40% (n=50) patients using the discovery cohort DMG network as a reference. A median risk cut-off from this group was then used to stratify the remaining 60% (n=75) held-out patients into high or low connectivity DMG risk groups, the prognostic impact of which was assessed via multivariable cox proportional hazards models incorporating clinical covariates as described. Median risk cut-offs and DMG network-to-tumour connectivity hazards ratios were obtained per fold and their median values summarised across all five folds. In addition, the concordance index comparison of model-predicted survival outcome with patient survival outcome across each fold was computed, enabling the evaluation of model accuracy in a non-circular fashion for time-to-event data.[48]"

The results of these analyses are also reported in the main text results:

"Having established network-to-tumour connectivity as a robust correlate of patient overall survival, we next sought to confirm its predictive power. To this end, we performed five-fold stratified cross-validation across external validation cohort data in line with previous work.[39] Within each fold, network-to-tumour connectivity values from 40% patients (n=50) were computed and used to define a median connectivity cut-off value which was then applied to a multivariable cox proportional hazards model across the remaining, held-out patients (60%; n=75). Median connectivity cut-off values remained stable when patients were randomly shuffled within training sets across folds (Fig. 2e). Corresponding hazards ratios and concordance indices demonstrated consistent, independent, and accurate prediction of patient overall survival (Fig. 2f-g; median hazard ratio 3.33; median concordance index 0.70)."

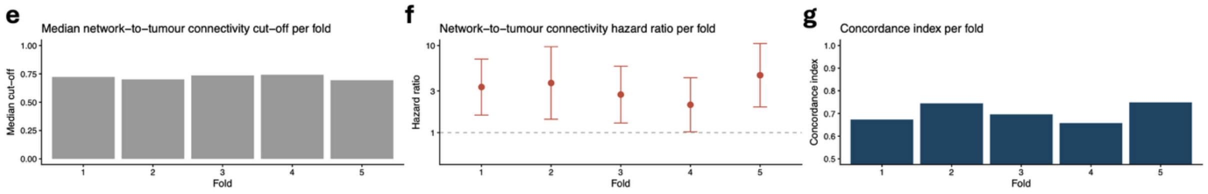

Main Text Fig. 2e-g. DMG-network-to-tumour connectivity predicts overall survival in children with DMG.
(E-G) *Results of five-fold stratified cross-validation demonstrate a stable cut-off for median network-to-tumour connectivity* ***(E)****; significant hazard ratios across all folds (mean hazard ratio 3.33)* ***(F)****; and accurate prediction in out-of-sample data (median concordance index 0.70)* ***(G)****. In all forest plots, mean values are represented by solid circles and whiskers represent 95% CI.*

7. For the analyses aiming to ensure prognostic specificity of the DMG network, how was network-to-tumor connectivity computed?

Thank you for the opportunity to clarify. We have included additional text in the methods to address this important question:

"We assessed the impact of mean network-to-tumour connectivity on patient overall survival. Mean network-to-tumour functional connectivity was derived by weighting the functional connections of each patient tumour to the DMG network with T values in the DMG network, restricted to only prognostically significant voxels, and then computing the mean of this weighted tumour connectivity. We hereafter refer to this as each patient's DMG-network-to-tumour functional connectivity. Such an approach has been successfully leveraged to test the out-of-sample robustness of connections associated with clinical improvements following effective deep brain stimulation for obsessive-compulsive disorder[39,110], Parkinson's disease [39], Gilles de la Tourette's disorder[39], and dystonia[39]."

We also considered it important to test if mean tumour-to-network connectivity retained its prognostic impact in out-of-sample data when using a different method to compute network-to-tumour connectivity. To this end, we computed tumour-to-network connectivity using a whole-brain Spearman correlation between patient-specific tumour network maps and a pre-computed connectome (PCC) map of the DMG network generated through seeding of weighted DMG network nodes. This method has also been previously reported by our group (e.g. Schaper … Fox. *JAMA Neurol*. 2023.) and is now also described in the methods, as below:

"To ensure that the prognostic impact of DMG network-to-tumour connectivity was robust to methodological choices, we repeated the above survival analysis using an orthogonal method to compute DMG network-to-tumour connectivity for each patient. DMG network peaks were used as weighted seeds in ABCD1000 to obtain a brain-wide distributed map representative of functional connectivity with brain regions of increased or decreased risk of shorter overall survival.[43] Each patient tumour-specific functional connectivity map across the external validation cohort was spatially compared to this pre-computed connectome (PCC) discovery DMG network peak functional connectivity map with a whole-brain spatial Spearman correlation.[40,43,45,104] Patients were stratified into high and low risk groups based on a median cut-off in spatial similarity values, where high spatial similarities were indicative of high DMG network-to-tumour functional connectivity."

Through these analyses, we demonstrate that the prognostic impact of DMG network functional connectivity is robust to the method by which network-to-tumour connectivity is computed. Both results are now included in the manuscript main text results (**Main Text Fig. 2a.** and **Extended Data Fig.6a.**, respectively) with additional text to further clarify our original method of computing tumour-to-network connectivity, as below:

"To validate the prognostic importance of the DMG network, the mean strength of network-to-tumour connectivity with the primary, discovery cohort defined DMG network was computed for each tumour across both external validation cohorts: data which were not used to define the DMG network. ***Network-to-tumour connectivity was computed as the weighted overlap of patient-level tumour functional connections with DMG network prognostic connections.[39]*** *Kaplan-Meier survival analyses following the stratification of patient tumours using a median cut-off confirmed the inverse association of high network-to-tumour connectivity with overall survival in children with DMG across both external multicentre and PNOC trial validation cohorts (**Fig. 2a-b**). Importantly, univariable and multivariable cox-proportional hazards models incorporating clinical covariates reproduced these findings and confirmed network-to-tumour connectivity as an independent predictor of overall survival in children with DMG (**Fig. 2a-b**). Findings were also replicated across connectomes (**Extended Data Fig. 5d-e**)* ***and using an orthogonal method to compute network-to-tumour connectivity in which each patient tumour-specific functional connectivity map was spatially compared to a pre-computed connectome (PCC) map generated through weighted seeding of DMG network peaks (Extended Data Fig. 6a).[43]"***

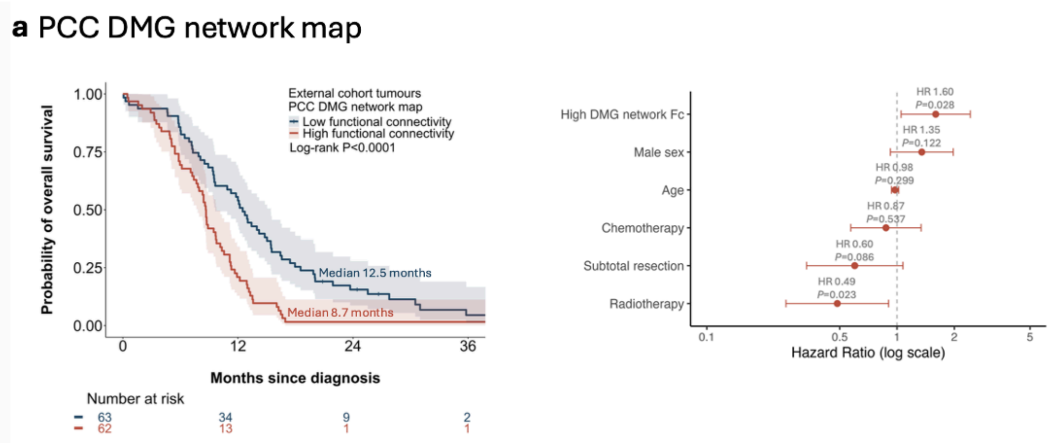

Extended Data Fig. 6. The prognostic significance of DMG network-to-tumour connectivity is robust to methodological choices.
(A) *Kaplan-Meier survival curve and associated multivariable cox proportional hazard model constructed using external validation cohort data with network-to-tumour connectivity calculated on the discovery cohort derived DMG network. The prognostic impact of network-to-tumour connectivity is robust when computed using an alternate, spatial-correlation method. Kaplan-Meier median overall survival 8.7 vs. 12.5 months; 95% CI 7.8-9.8 vs.9.6-15.4 months; P<0.0001. Multivariable cox HR 1.60; 95% CI 1.05-2.43; P=0.028.*

8. The analyses of "all pontine tumors" and "all thalamic tumors" in Figure 2 appear to be circular in the sense that the thalamic-DMG and pontine-DMG networks were identified via tumor-network analyses in the subsets of patients with pontine/thalamic tumors on the basis that these networks showed significant group-level differences in FC between short- vs. long-term survivors within the subsets of patients with pontine/thalamic tumors that were used to define them. The resulting survival and modeling analyses that include mean tumor-DMG connectivity therefore appear to be circular, and the estimated effects/significance could be optimistically inflated (e.g. Vul et al., 2009). If these analyses are circular, then the Hazard Ratios and statistical significance of mean functional connectivity predictors of survival outcomes are not valid since the network used to measure mean functional connectivity was defined based on it showing a significant relationship to survival outcomes in those same patient samples. Please clarify whether these analyses are circular and adjust the reported results accordingly if necessary.

This is an important point which we thank the reviewer for the opportunity to clarify.

In all instances, we used a discovery dataset to define a network and a second, independent and held-out dataset to evaluate the prognostic importance of that network. For example, external cohort network-to-tumour connectivity values were computed using the discovery cohort network. To more directly address the reviewer's question regarding pontine and thalamic tumour survival analyses, we employed the following method:

1. A pontine network was constructed using all pontine tumours across the discovery and external validation cohorts.
2. A thalamic network was constructed using all thalamic tumours across the discovery and external validation cohorts.
3. Survival analyses of pontine tumours were then performed with network-to-tumour connectivity calculated using the thalamic DMG defined network.
4. Survival analyses of thalamic tumours were then performed with network-to-tumour connectivity calculated using the pontine DMG defined network.

These analyses were performed to further confirm convergence of pontine and thalamic DMG networks beyond label and spin permutation tests – i.e. to confirm if the prognostic connections from one tumour location would predict the overall survival of the other tumour location. In this paradigm, no analyses were circular as distinct cohorts were used to (1) define the network and (2) evaluate the impact of that network on patient overall survival.

To clarify this in the manuscript, we have greatly simplified **Main Text Fig. 2.**, whilst also providing much greater methodologic description in the figure legend as below:

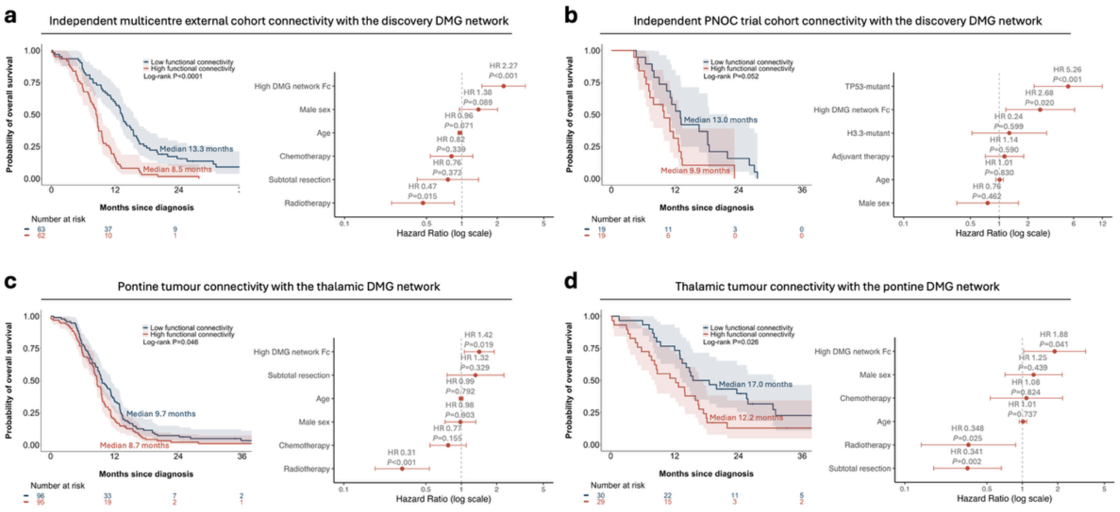

Fig. 2. DMG-network-to-tumour connectivity predicts overall survival in children with DMG. ***(A-D)*** *Kaplan-Meier survival curves and associated multivariable cox proportional hazard models demonstrate shorter overall survival in children whose tumours exhibit higher DMG network connectivity and confirm network-to-tumour connectivity as an independent predictor of patient overall survival.* ***(A)*** *125 children with primary pontine and thalamic DMG in an independent, external validation cohort with network-to-tumour connectivity calculated on the discovery DMG network. Kaplan-Meier median overall survival 8.5 vs. 13.3 months; 95% CI 7.4-9.5 vs. 11.7-15.5 months; P<0.0001. Multivariable cox HR 2.27; 95% CI 1.48-3.47; P<0.001.* ***(B)*** *38 children with biopsy-confirmed pontine DMG with network-to-tumour connectivity calculated on the discovery DMG network. Kaplan-Meier median overall survival 9.9 vs. 13.0 months; 95% CI 7.3-13.1 vs. 11.1-21.9 months; P=0.052. Multivariable cox HR 2.68; 95% CI 1.17-6.13; P=0.020.* ***(C)*** *191 children with pontine DMG with network-to-tumour connectivity calculated on a thalamic DMG network. Kaplan-Meier median overall survival 8.7 vs. 9.7 months; 95% CI 8.2-9.6 vs. 8.9-11.4 months; P=0.046. Multivariable cox HR 1.42; 95% CI 1.06-1.90; P=0.019.* ***(D)*** *59 children with thalamic DMG with network-to-tumour connectivity calculated on a pontine DMG network. Kaplan-Meier median overall survival 12.2 vs. 17.0 months; 95% CI 8.3-16.6 vs. 13.4-31.0 months; P=0.026. Multivariable cox HR 1.88; 95% CI 1.03-3.46; P=0.041.* ***(E-G)*** *Results of five-fold stratified cross-validation demonstrate a stable cut-off for median network-to-tumour connectivity* ***(E)****; significant hazard ratios across all folds (mean hazard ratio 3.33)* ***(F)****; and accurate prediction in out-of-sample data (median concordance index 0.70)* ***(G)****. In all Kaplan-Meier curves, the statistical difference between groups was evaluated using the log-rank test with error bars representative of 95% CI. In all forest plots, mean values are represented by solid circles and whiskers represent 95% CI.*

Further, we have added the following paragraph to the results in order to clarify the rationale, methodology, and results of these analyses:

"To formally test whether pontine and thalamic DMG exhibit prognostic convergence to the same brain network, we performed tumour network mapping separately on all pontine and all thalamic DMG to define respective pontine and thalamic DMG networks associated with patient short-term survival (Extended Data Fig. 3f-g). The mean connectivity strength of pontine DMG was then computed with the thalamic DMG network whilst the mean connectivity strength of thalamic DMG was computed with the pontine DMG network. Using this paradigm, we were able to test, in a non-circular manner, whether pontine and thalamic DMG connectivity with the alternate location DMG network was associated with patient overall survival. Kaplan-Meier survival curves consistently demonstrated the adverse prognostic impact of high network-to-tumour connectivity across all pontine DMG (Fig. 2c) and all thalamic DMG (Fig. 2d). Univariable and multivariable cox-proportional hazards models incorporating clinical covariates reproduced these findings and confirmed network-to-tumour connectivity as an independent predictor of overall survival in children with pontine and thalamic DMG (Fig. 2c-d)."

9. The analyses of patients with co-mutations in TP53 or BRAF/FGFR1 are not clearly explained in the main text or the methods. It's also unclear how many patients were included in the analyses of the "restricted cohort of cases diagnosed only by tissue biopsy". Based on the "modeling the prognostic impact of extent of resection" section, it appears this may be n=23, but the sample sizes should be clearly stated for each analysis within the section describing that analysis. Please clarify the methods and overarching rationale for this analysis, and please ensure that sample sizes are clearly reported for all analyses.

Thank you for raising this. We have revised the manuscript main text to provide clear rationale for our interrogation of potential differences in network-to-tumour connectivity in patients with different tumour co-mutations, as below:

*"**DMG are characterised by canonical (H3.1/H3.2) or non-canonical (H3.3) alterations in histone 3[60,61] or EZHIP [60,61]overexpression[62], all of which result in polycomb repressor complex 2 (PRC2) inhibition[63], global loss of H3K27 trimethylation, and widespread epigenetic dysregulation.[64] More recent work has also demonstrated that DMG may be stratified into two, molecularly defined, prognostic groups based on the presence of oncogenic variants in TP53 or BRAF/FGFR1.[32,65] To investigate a potential association between network-to-tumour connectivity and oncohistone variant or somatic co-mutation, we collected paediatric solid tumour next-generation DNA panel sequencing data for a subcohort of 87 children with primary pontine or thalamic DMG, H3K27-altered, and compared the mean strength of DMG-network-to-tumour connectivity for each alteration. Negative results obtained suggest that the strength of network-to-tumour connectivity is independent of both tumour histone status and co-mutational burden (Extended Data Fig. 6e).**"*

We have also revised the representation of these analyses and their associated legend in the Extended Data, as below:

e Network-to-tumour connectivity

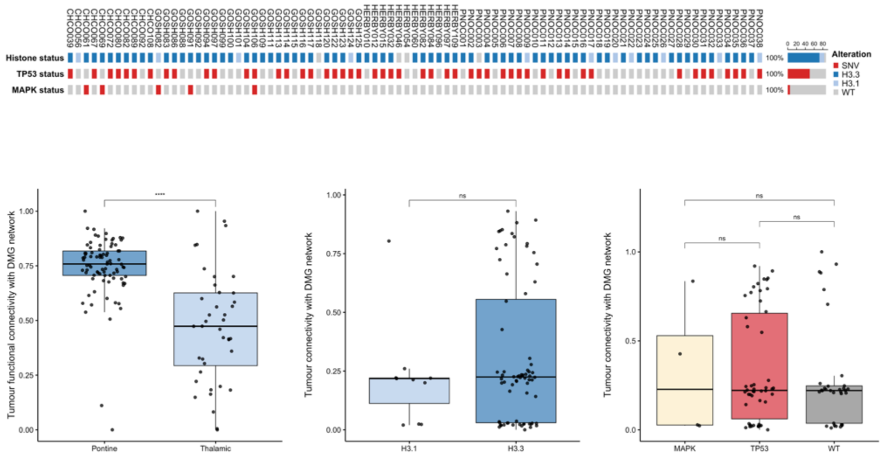

Extended Data Fig. 6e. The prognostic impact of DMG network-to-tumour connectivity is independent of co-mutations.
(E) *Comparison of external validation cohort network-to-tumour connectivity values revealed that pontine DMG exhibited greater DMG network functional connectivity than thalamic DMG (median 0.758 vs. 0.473; two-tailed Mann-Whitney U-test P=3.12e-9). Importantly, comparison of 87 tumours across the whole cohort with next-generation DNA panel sequencing identified no difference in network-to-tumour connectivity when stratifying by oncohistone variant (H3.1 vs. H3.3; median Fc 0.218 vs. 0.225; two-tailed Mann-Whitney U-test P=0.401) and somatic mutation (MAPK pathway (BRAF/FGFR1) vs. TP53 vs. WT; median Fc 0.227 vs. 0.222 vs. 0.221; MAPK vs. WT two-tailed Mann-Whitney U-test P=0.934; TP53 vs. WT two-tailed Mann-Whitney U-test P=0.856). For these latter analyses, network-to-tumour connectivity values were calculated for pontine and thalamic DMG*

from the opposing DMG network (as in ***Main Text Fig. 2****) and normalised within each group, enabling pan-cohort comparison.*

More broadly, we have expanded the manuscript main text to include greater rationale for all included analyses, as well as clearer documentation of which patients form part of specific analyses. Further, we have included **Main Text Fig. 1a** as a methodologic overview (below) and have ensured that figure legends reporting specific analyses contain the specific power of those analyses.

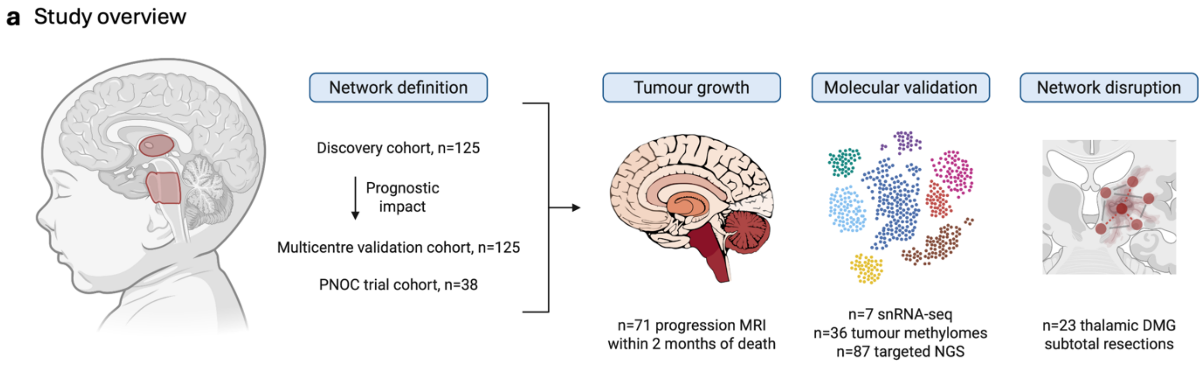

10. Many analyses simply state that they used "the DMG network". However, many DMG networks were derived in this study, with two primary networks - a functional network and a structural network identified in the initial analyses of the Discovery cohort. Presumably, "the DMG network" refers to the functional network identified in the initial analyses of the Discovery cohort (e.g. based on Figures), but this should be made clear to the reader. Also, there does not appear to be any clear reason to focus on the functional network over the structural network, and I'm surprised that there are no analyses aimed at evaluating whether one approach is more prognostic than the other. Is there a strong conceptual or mechanistic reason to prioritize the functional network over the structural network? Even if there is, I think that readers (including myself) would be interested in understanding whether there are differences in prognostic value between the structural and functional networks. Multivariate analyses like the one described in Major Comment 2 could also provide insights into the relative prognostic value of the structural and functional networks.

Thank you for suggesting that we interrogate the DMG structural network more closely and for noting the need for greater clarity in our specification of which network we take to be the "DMG network". To first clarify the latter, we have added the below sentence to the main text results:

"**Hereafter, we refer to this discovery cohort derived map of functional brain network connections associated with shorter patient overall survival as the DMG network (Fig. 1d.).**"

We have also avoided referring to other networks as "DMG" networks – instead, for example, referring to them as the "external validation cohort network" or the "PNOC trial cohort network" to maintain this distinction. This has the added benefit of clarifying which data were used to generate each network.

To speak directly to the reviewer's recommendation of interrogating the prognostic value of the DMG structural network, we have now replicated our survival analyses (Kaplan-Meier and cox proportional hazards) across these data: calculating the network-to-tumour connectivity of external validation cohort tumours on the discovery cohort derived DMG structural network. Though we demonstrate that the pattern of brain network connectivity associated with patient short-term survival (DMG structural network) is reproducible across discovery and validation cohorts (**Extended Data Fig. 4a,l.**), these analyses failed to demonstrate that DMG structural network-to-tumour connectivity could stratify patients into survival risk groups or independently predict patient overall survival (**Extended Data Fig. 7a.**). This finding is now reported in the main text, as below with data overleaf, and caused us to focus predominantly on the DMG functional network:

"**Similarly, DMG network structural connectivity, representative of monosynaptic neuron-tumour connections, was non-prognostic (Extended Data Fig. 7a).**"

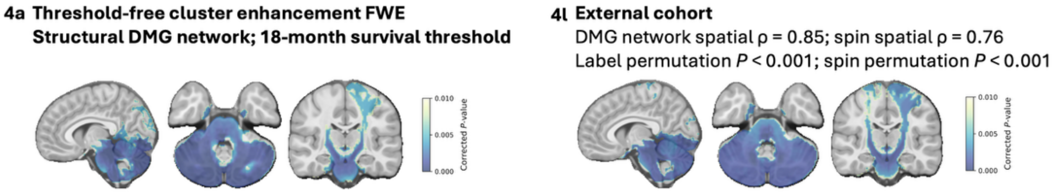

Extended Data Fig. 4a,l. DMG network structural connectivity is robust to statistical and methodological choices across dMRI data though does not predict patient overall survival across independent cohorts.
*DMG network mapping was repeated using dMRI data across 497 healthy children from the Human Connectome Project Lifespan Development (HCP-D) dataset, identifying a structural network of monosynaptic tumour connections associated with shorter patient overall survival **(A)**. Strong connectivity was observed with brain regions identified in the DMG network, particularly via the cerebellar peduncles, corticospinal tracts, corpus callosum, corticobulbar tracts, and spinal-limbic pathways… Finally, the structural DMG network was reproducible using patient data from an independent, external validation cohort **(L**; spatial ρ=0.854, label permutation P<0.001; spin spatial ρ=0.760, spin permutation P<0.001**)**.*

Further to this, and in response to Major Comment 3 by the same reviewer, we compared the relative prognostic value of tumour location and DMG structural / functional network information. To this end, we constructed additional multivariable cox proportional hazards models incorporating these covariates (**Extended Data Fig. 7.**). These data demonstrate a robust and persistent association between DMG functional network-to-tumour connectivity on patient overall survival but not between DMG structural network-to-tumour connectivity or DMG tumour location and patient overall survival.

We have included additional text in the Main Text to reflect these results, as below:

"To confirm this finding and to directly compare the prognostic impact of tumour network and tumour location information, we constructed additional cox proportional hazards models incorporating these covariates. These consistently demonstrated a robust association between high network-to-tumour functional connectivity and patient overall survival but failed to demonstrate any association between high network-to-tumour structural connectivity or tumour-VLSM map overlap and patient overall survival (Extended Data Fig. 7b-c). Collectively, we demonstrate that mean DMG network-to-tumour functional connectivity is robustly and reproducibly associated with patient overall survival."

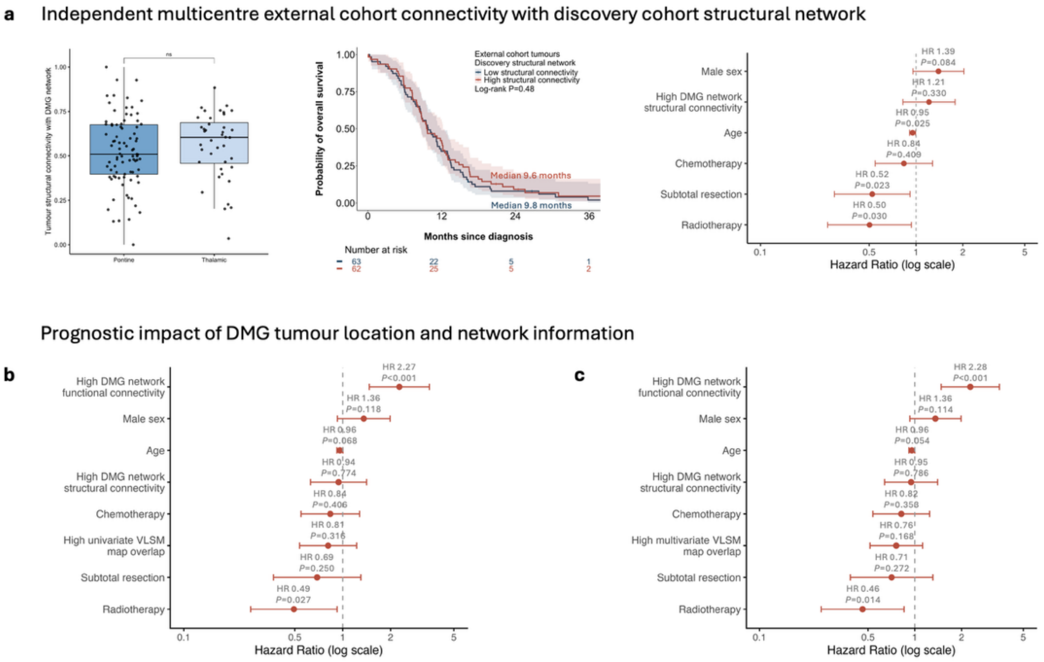

Extended Data Fig. 7. DMG network functional connectivity, rather than tumour location and DMG network structural connectivity, is associated with patient overall survival.
(A) *Comparison of external validation cohort network-to-tumour connectivity values revealed that there was no significant difference in structural connectivity to the DMG network between pontine DMG and thalamic DMG (median 0.510 vs. 0.604; two-tailed Mann-Whitney U-test P=0.234). DMG network-to-tumour structural connectivity was not independently associated with patient overall survival across the external multicentre validation cohort* **(***centre and right***)***. Kaplan-Meier median overall survival 9.8 vs. 9.6 months; 95% CI 8.6-11.9 vs. 8.6-12.2 months; P=0.48. Multivariable cox HR 1.21; 95% CI 0.82-1.78; P=0.330.*
(B-C) Direct comparison of the prognostic impact of tumour location and tumour network information across the external multicentre validation cohort (**B** – univariate VLSM; **C** – multivariate VLSM**)**. In both instances, network-to-tumour functional connectivity was an independent predictor of patient overall survival (**B**: multivariable cox HR 2.27 95% CI 1.47-3.51; *P*<0.001; **C**: multivariable cox HR 2.28 95% CI 1.48-3.51; *P*<0.001**)**.

Further, as the reviewer is right to note, there is conceptual importance to this finding. Namely, that our combined survival analyses emphasise the importance of distributed, network-level connections on patient overall survival – as shown by our primary functional DMG network – rather than isolated structural connections. We have added text to the discussion to this effect, as below:

'**The prognostic impact of brain-wide DMG network functional connectivity emphasises the importance of distributed, network-level monosynaptic and polysynaptic connections on patient overall survival.**"

11. The tumor infiltration analyses are interesting, but the claims about the preferential infiltration of the DMG network appear to be supported by only qualitative data (i.e. lines 354-356 and 371-372, Figure 3G, Extended Data Fig.18). I think the authors should explicitly test whether infiltration was preferential to the DMG network. This could be achieved by e.g. evaluating whether the proportion of new tumor tissue was significantly greater within vs. outside of the DMG network across patients? I think that something like this is necessary to support the stated conclusions.

This is a very good point. We have now implemented this analysis and added an additional subplot to **Main Text Fig. 3b** to reflect this (as below). As hypothesised, tumour volumetric growth within the DMG network was significantly greater than tumour volumetric growth out of the DMG network between diagnostic and final MRI (median 13,768 vs. 1,270 mm^3; 95% CI 11,293-17,763 vs. 773-1,792 mm^3; two-tailed paired Wilcoxon signed-rank test P=6.85e^{-13}). Patient-level tumour growth volumes are shown in grey.

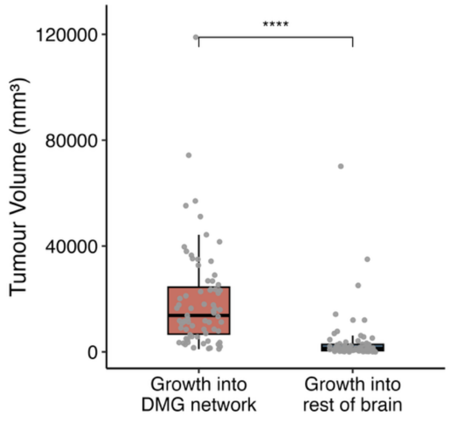

12. The rationale for the cubic spline analysis of FDG-PET is unclear, and the methods do a poor job of explaining why cubic spline interpolation was considered necessary for this analysis and how it impacts the interpretation of results. How were within- vs. out-of-DMG network regions defined (e.g. any overlap with DMG network, minimum % overlap with DMG network, etc.)? How much of the brain/how many parcels were within vs. outside of the DMG network? The ROIs couldn't be truly defined "a priori" since they depend on the results of the DMG network analyses, but regardless, the specific method for defining them should be clearly reported.

Thank you for highlighting this. Cubic spline functions were fit to [^{18}F]FDG-PET data, as reported in previous studies which have determined the complex age trend for glucose uptake across childhood (Kuzawa … Lange. *Proc Natl Acad Sci USA*. 2014.). In this approach, we used spline interpolation to harmonise age-wise [^{18}F]FDG-PET changes across childhood with continuous ages (to the nearest month) of patients across cohorts. However, we recognise that interpolation of [^{18}F]FDG-PET data between ages risks overestimating the linear relationship between DMG age incidence and age-wise changes in [^{18}F]FDG-PET signal. With this in mind, we now take a different and more intuitive approach in which we compare age-wise changes in [^{18}F]FDG-PET signal with DMG patient ages rounded to the nearest year. With either analytic method, the strong linear relationship between DMG age incidence and [^{18}F]FDG-PET signal is preserved (revised *R=0.612, P=0.012*). To speak to the reviewer's second question, brain regions were considered within the DMG network if ≥50% voxels in each brain region parcel overlapped with a binarised DMG network map.

We have updated the manuscript methods to reflect both of these changes, as below:

"Modelling DMG incidence with [^{18}F]FDG-PET data
DMG incidence was extracted from frequency of diagnosis at each age by rounding age at diagnosis across the whole study cohort down to the nearest year. Age-averaged [^{18}F]FDG-PET data were obtained from a population-level paediatric [^{18}F]FDG-PET atlas comprised of 795 children with inclusion criteria and acquisition parameters as previously reported.[75] The voxel-wise mean-squared difference (MSD) between age-consecutive whole-brain standardised uptake values (SUV) was computed, reflecting the mean difference in [^{18}F]FDG-PET uptake between consecutive ages, and plotted against DMG incidence data across childhood (0-16 years) rounded to the nearest whole year. A linear regression model was fit between age-wise MSD and DMG incidence to assess the variance explained by model fit. 95% CI for the variance in DMG age incidence data explained by [^{18}F]FDG-PET signal changes was evaluated using 2000 bootstrap iterations. Next, to assess the specificity of the linear relationship between [^{18}F]FDG-PET signal and DMG incidence, age-averaged [^{18}F]FDG-PET data were parcellated according to paediatric MINCI 60MB SynthSeg anatomic masks (n=46). Brain region anatomic masks were defined as within the DMG network if ≥50% voxels in each mask overlapped with those in the DMG network; brain regions not meeting this criterion were categorised as out of the DMG network. Region-wise and age-wise [18F]FDG-PET signal changes within and out-of-DMG network were then compared in relation to their linear regression model fit with DMG age incidence using a two-tailed Mann-Whitney U-test with correction for multiple comparisons.

Spatiotemporal specificity of peak childhood [^{18}F]FDG-PET changes
[^{18}F]FDG-PET maps were compared to identify voxelwise differences between consecutive ages. The age at which the maximal difference in normalised [^{18}F]FDG-PET signal occurred across all ages was identified per voxel and assigned this age value. Voxelwise spatial maps of age-wise peak metabolic changes were thereby generated and compared for age-wise spatial similarity with the DMG network with label permutation and spin permutation tests, as described."

Given this more intuitive methodologic approach and the removal of spline interpolation, we have also opted for a greatly simplified and more understandable visual presentation of these data, as below:

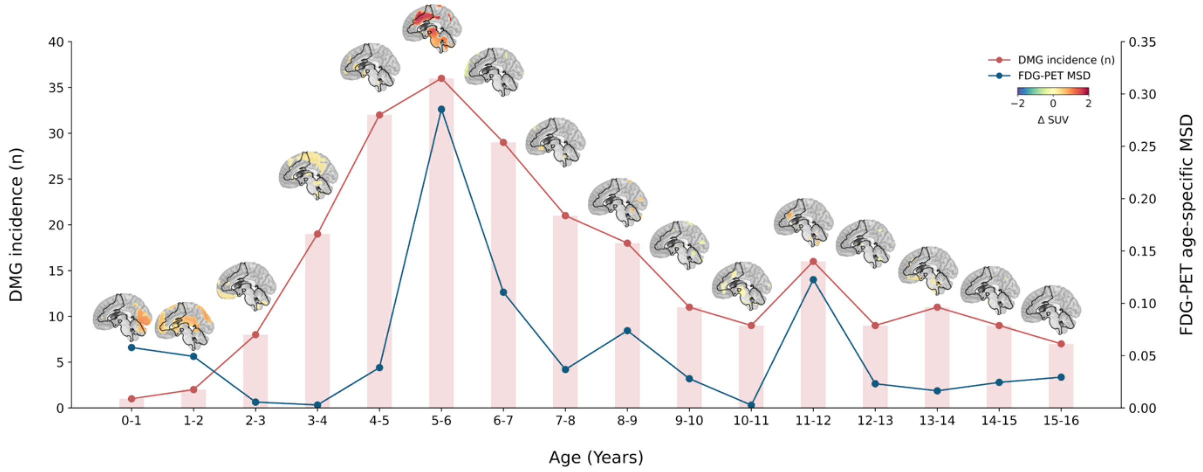

Fig. 5. DMG network topography spatiotemporally coincides with neurometabolic development.
Line, bar, and scatter plot of whole cohort DMG age incidence (red; n=284, 4 patients aged >17 years omitted) with line and scatter plot of age-consecutive mean-squared differences (MSD) in brain-wide $[18^{F}]$FDG-PET signal (blue) across childhood (0-16 years) show the temporal alignment of peak DMG incidence with peak $[18^{F}]$FDG-PET signal changes (R=0.612, linear regression P=0.012). Brain regions with peak difference in $[18^{F}]$FDG-PET signal at each age across childhood are shown as brain-wide spatial maps plotted above each bar. Brain regions were identified by tagging each voxel across the brain with the age at which it underwent its greatest change in in $[18^{F}]$FDG-PET signal. Colours represent the voxelwise strength and direction of change in $[18^{F}]$FDG-PET signal between consecutive ages (red indicative of greater positive change, blue greater negative change). Peak changes in $[18^{F}]$FDG-PET signal between ages 5 and 6 years and 10 and 11 years, coinciding with age incidence peaks in DMG, strongly overlap with regions in the DMG network (ages 5-6 years peak changes DMG network whole brain spatial ρ=0.199, label permutation P<0.001, spin spatial ρ=0.512, spin permutation P<0.001; ages 10-11 years peak changes spatial ρ=0.044, label permutation P<0.001, spin ρ=0.179, spin permutation P<0.001).

Finally, these changes are reflected in the manuscript results:

*“**The early childhood peak incidence of pontine gliomas has been reported since the early 1950s**.[73] Specifically, DMG occur in a distinct spatiotemporal pattern during childhood and are rare in adulthood with the pontine and thalamic disease exhibiting peak incidence at 6 and 11 years of life, respectively.[2] This broadly aligns with periodic changes in postnatal myelination, an observation concordant with the OPC-like lineage of DMG, as well as with key neurodevelopmental stages.[27,69,74] Given that increased neural activity is critical for high-grade glioma initiation and infiltration, we hypothesised that the physiological neurometabolic development of brain circuits connected to the DMG network would explain the spatiotemporal pattern of DMG incidence.[3,9] Here, we harness a **physiologic**, population-level atlas of [^{18}F]fluoro-2-deoxy-D-glucose PET ([^{18}F]FDG-PET) imaging across childhood to investigate whether DMG incidence and the spatial organisation of the DMG network are correlated with neurometabolic development.[75] First, we interrogated the relationship between DMG incidence and [^{18}F]FDG-PET signal within the DMG network **from 0-16 years of life**. To this end, DMG whole cohort age incidence and voxelwise mean-squared differences in consecutive, age-averaged [^{18}F]FDG-PET signal within the DMG network were compared across ages (Fig. 5).[76] Regression of **age incidence and age consecutive signal changes exhibited a strong linear dependence (R=0.612, P=0.012)**, with the maximal peak change in [^{18}F]FDG-PET signal at 5-6 years of life and the second maximal peak change at 11-12 years of life, coinciding with the relative peak age incidence of pontine and thalamic DMG, respectively (Fig. 5). **To evaluate the specificity of the linear relationship between DMG age incidence and changes in [^{18}F]FDG-PET signal across childhood to the DMG network, we extracted [^{18}F]FDG-PET signal from 46 brain regions pre-defined as within or out of the DMG network. Regression of each brain regional model against DMG whole cohort age incidence identified that changes in [^{18}F]FDG-PET signal in brain regions within the DMG network explained significantly greater variance in DMG***

incidence across all of childhood than [^{18}F]FDG-PET signal in the rest of the brain (median in-network R=0.327 vs. median out-of-network R=0.197; two-tailed Mann-Whitney U-test P_{FDR}=0.015).

We next sought to investigate whether these peak changes in ***neurometabolic activity were specific to brain regions in the DMG network using an orthogonal, data-driven analysis****. To this end, [18F]FDG-PET maps across childhood were* ***compared and the greatest*** *voxelwise changes in normalised [18F]FDG-PET signal during childhood plotted by age. Strikingly, these data show that the peak positive neurometabolic change during childhood between 5-6 years of life is spatially dominated by brain areas in the DMG network, including the pons, whilst the second largest neurometabolic change during childhood, between 10-11 years of life, is also spatially dominated by brain areas in the DMG network, predominantly the thalamus (****Fig. 5****).* ***Importantly, anatomic maps of the peak neurometabolic change for ages 5-6 years and 10-11 years were significantly more similar to the DMG network than expected by chance upon both label and spin permutation testing and in contrast to peak neurometabolic changes at other ages (Extended Data Table 1).*** *Together, these data characterise the DMG network as a site of spatiotemporally organised neurometabolic development associated with tumour incidence. This spatiotemporal alignment defines a neurodevelopmental window in which brain regions involved in the DMG network undergo their most significant neurometabolic transitions, possibly reflecting critical periods of vulnerability to DMG initiation via neural activity."*

13. Figure 4D – these results also appear to be descriptive. It seems like quantitative statistical tests could be used to demonstrate that the delta SUVmax topography for certain age bins is more similar to the DMG network than the delta SUVmax for other age bins. I think that this should be done to support the conclusions.

Thank you for highlighting this; we agree that formal tests should be used. To this end, we have implemented both label and spin permutation tests to evaluate the spatial similarities of each age-wise change in neurometabolic activity to the DMG network and their statistical significance. In doing so, we found that peak neurometabolic changes at ages 5-6 and 10-11 years are significantly more similar to the DMG network than could be expected by chance when compared to all other ages. The full data for these analyses have been included in **Extended Data Table 1**, as below:

	Label permutation test		Spin permutation test	
	ρ	p_{FDR}	ρ	p_{FDR}
**0-1 years**	-0.03	0.018	-0.13	0.436
**1-2 years**	-0.08	1.000	-0.14	0.366
**2-3 years**	-0.05	<0.001	-0.13	0.463
**3-4 years**	-0.07	1.000	-0.19	0.454
**4-5 years**	0.06	<0.001	0.12	0.461
**5-6 years***	0.20	<0.001	0.51	0.001
**6-7 years**	-0.05	<0.001	-0.15	0.402
**7-8 years**	-0.05	<0.001	-0.14	0.420
**8-9 years**	0.04	<0.001	0.16	0.221
**9-10 years**	-0.02	1.000	-0.01	0.924
**10-11 years***	0.12	<0.001	0.37	0.002
**11-12 years***	0.08	0.228	0.37	0.023
**12-13 years**	0.02	<0.001	0.01	0.980
**13-14 years**	0.04	1.000	0.13	0.258
**14-15 years**	0.02	<0.001	0.08	0.639
**15-16 years**	-0.03	<0.001	-0.08	0.440

Supplementary Table 6. Spatial comparison of age-wise changes in neurometabolic activity with DMG network topography.
Values represent label and spin permutation test results. Asterisks represent significant spatial similarity of peak, age-wise neurometabolic changes with DMG network topography. Red text indicates negative values.

We have also included additional text in the results and **Main Text Fig. 5** legend to reflect this:

Strikingly, these data show that the peak positive neurometabolic change during childhood between 5-6 years of life is spatially dominated by brain areas in the DMG network, including the pons, whilst the second largest neurometabolic change during childhood, between 10-11 years of life, is also spatially dominated by brain areas in the DMG network, predominantly the thalamus (Fig. 5). ***Importantly, anatomic maps of the peak neurometabolic change for ages 5-6 years and 10-11 years were significantly more similar to the DMG network than expected by chance upon both label and spin permutation testing and in contrast to peak neurometabolic changes at other ages (Supplementary Table 6).****Together, these data characterise the DMG network as a site of spatiotemporally organised neurometabolic development associated with tumour incidence."*

14. A primary conclusion of the paper is that it provides real-world evidence demonstrating that neural activity modulates tumor growth in humans. However, I don't think that that it actually does this. Instead, it shows that tumors in locations with specific network connectivity patterns are associated with worse survival outcomes and have distinct chemoarchitectural and epigenetic features than tumors in locations that don't exhibit those connectivity patterns. It also provides evidence indicating that age-related changes in neural metabolism within the identified network coincide with ages of peak tumor incidence. While together, these observations are consistent with the conclusion that neural activity is modulating tumor growth and provide valuable insights into how various tumor characteristics relate to survival outcomes and to each other, they don't provide any direct evidence that it is neural activity itself that is directly modulating tumor growth. I think that this conclusion should be revised to reflect the fact that none of the analyses showed (or could have shown) a direct relationship between neural activity and tumor growth.

Thank you for raising this. We have, as previously mentioned in our response to Reviewers 1 and 2, revised some of the language in our manuscript, where appropriate, to better reflect conclusions directly supported by our data. In particular, whilst we do not directly measure or manipulate neural activity in relation to tumour growth in children with DMG, nor could we have aimed to do so, we infer neural activity by means of tumour functional connections defined by a normative paediatric connectome in relation to both patient overall survival and the anatomic pattern of tumour growth. We have, therefore, clarified the manuscript main text throughout and have added the following explicit acknowledgement of this limitation to the discussion:

"Further, whilst the role of neuromodulatory neurotransmitters in DMG growth, in particular combinatorial cholinergic, serotonergic, and noradrenergic signalling, remains to be fully described, preclinical reports have revealed the robust effects of brainstem cholinergic neurons and muscarinic signalling on DMG growth and invasion.[5] **Finally, none of the experiments presented herein demonstrate nor could have demonstrated a direct relationship between tumour neural activity and tumour growth in children. Within the framework of causal human brain mapping[42], however, the convergent data presented across multiple modalities are consistent with the hypothesis that DMG network connectivity is associated with DMG tumour growth and patient overall survival.**"

Specifically, to the reviewer's point regarding our manuscript conclusion, we have also revised this text to read:

"Taken together, our results define a spatially conserved map of prognostic brain network connections in children with DMG and are consistent with preclinical evidence that DMG growth and infiltration are robustly regulated by diverse, brain-wide neuronal interactions. *Pharmacologic and surgical targeting of these defined vulnerabilities may be critical for the development of novel, circuit-level therapeutics for this devastating childhood cancer."*

Minor Comments

1. It's unclear how exactly the partial correlation analysis using the 4th ventricle CSF signal was performed. Was this a temporal signal that was included in an fcMRI nuisance regression model during preprocessing of data from each healthy individual? Or was this a temporal signal that was partialled out while computing the connectivity between the tumor and the rest of the brain in the post-processed data? I lean towards it being the latter, but it should be clear from the text.

Thank you for the opportunity to clarify this aspect of our methodology. We partialled out temporal CSF signal when computing each patient tumour functional connectivity map prior to tumour network mapping in PALM. To clarify this, we have revised the main text, as below:

"The parenchymal origin of pontine fMRI signal was confirmed through the inclusion of fourth ventricular cerebrospinal fluid signal as a co-regressor in partial correlation analysis when computing the functional connectivity of each patient tumour with the rest of the brain[53] (Extended Data Fig. 3l) and through visualisation of increasingly extreme statistical thresholds (Extended Data Fig. 3m-n)."

We have also clarified this in the Methods section, as below:

"Further, given the brainstem-predominant location of DMG, we sought to exclude the possibility of the DMG network being derived from or confounded by fMRI signal originating within surrounding CSF (e.g. secondary to heartbeat or CSF pulsations).[108] ***We verified this by implementing a voxelwise partial correlation analysis when computing whole-brain-to-tumour connectivity for each patient, controlling for temporal signal derived from a fourth ventricular CSF mask[53]. Tumour network mapping was then repeated in PALM across these patient tumour functional connectivity maps with CSF signal partialled out.*** *We also tested the convergence of our results across three normative connectomes with different pre-processing methods: ABCD1000[49], Yeo1000[54], and GSP1000.[55] DMG network signal origin was also interrogated using increasingly strict, stepped statistical thresholds to visualise and eliminate noise."*

[No minor comment 2.]

3. It's not clear why the post-hoc mediation analyses were necessary. Couldn't tumor volume just have been included as a covariate for each voxel-wise model when generating the DMG networks? This is a more typical approach for lesion volume control in mass-univariate analyses, so it's not clear why the post-hoc mediation analysis was used instead. It would be helpful to explain the rationale for this choice in the methods.

This is an important point and for which we had no specific rationale. We agree that a more elegant analytic approach would be to include tumour volume as a covariate in PALM when generating the DMG network, as in our previous work (Joutsa … Fox. *Nat Med*. 2022.; Schaper … Fox. *JAMA Neurol*. 2023.). To this end, we have reworked all analyses to use this 'new' DMG network across the manuscript and removed all post-hoc mediation analyses.

Related to this and in response to a similar, latter comment made by the same reviewer, we used patient tumour segmentations to assess the direct impact of tumour volume on patient overall survival using a univariate cox model. In doing so, we failed to identify as association between tumour volume and overall survival (univariate cox hazard ratio 1.00; *P*=0.569), in line with recent clinical literature (Jansen … Vuurden. *Neuro Oncol*. 2015.; Tam … Yeom. *Neuro Oncol Adv*. 2021.). We have included additional text in the results to highlight this finding and our revised methodologic approach:

"*Here, we perform tumour network mapping with the aim of identifying common network connections associated with DMG patient short-term survival, irrespective of tumour primary pontine or thalamic location.* ***Greater tumour volume was not associated with patient overall survival (univariate cox hazards ratio 1.00; P=0.569), in keeping with previous clinical reports[47,48], and so tumour network mapping analyses were performed controlling for volume, in line with previous work.[40,43,45]"***

4. Many if not most analyses use mean tumor-to-DMG connectivity as the network feature. However, even patients with very high peak tumor-to-DMG connectivity values could have relatively low mean tumor-to-DMG connectivity estimates if there are many weak connections within the tumor. From a conceptual/mechanistic standpoint, is there a reason that the mean tumor-DMG connectivity should be more informative than the peak tumor-to-DMG connectivity or the sum of all tumor-to-DMG connectivity values (i.e. since this would not be penalized by low values in the same way as the mean)? Is it important from a conceptual or mechanistic standpoint that there is consistently strong connectivity between the tumor and DMG?

This is an interesting question which, to answer, we performed additional survival analyses as the reviewer suggested: stratifying external validation cohort patients by their maximum, minimum, and summed network-to-tumour functional connectivity. In doing so, we confirmed that only mean network-to-tumour connectivity provided meaningful prognostic information, i.e. was able to predict patient overall survival across multiple datasets and analytic methods (Kaplan-Meier; cox proportional hazards). Minimum network-to-tumour connectivity did stratify patients into survival groups using the Kaplan-Meier method though this reflected a tumour-wide shift towards higher or lower mean network-to-tumour connectivity (Spearman's ρ=0.23; *P*=0.011). We have included these data in the main text and **Extended Data Fig. 6b-d**, as below. Conceptually, the fact that maximum, minimum, and summed connectivity did not stratify patients into survival groups does support our hypothesis that neural activity within a brain-wide network, rather than discrete communication between individual regions with very high or low connectivity, influences patient overall survival.

"Conceptually, we also sought to understand whether minimum, maximum, or summed network-to-tumour connectivity would demonstrate a similar prognostic effect. Notably, minimum tumour connectivity did stratify patients into meaningful survival groups using the Kaplan-Meier method (Extended Data Fig. 6b), though this reflected a general, tumour-wide shift towards higher or lower mean network-to-tumour connectivity (Spearman's ρ=0.23; P=0.011). Further, multivariable cox proportional hazards models constructed across these measures failed to demonstrate an independent association with patient overall survival (Extended Data Fig. 6b-d), reinforcing the importance of distributed, network-level effects rather than isolated strong or weak connections. Similarly, DMG network structural connectivity, representative of monosynaptic neuron-tumour connections, was non-prognostic (Extended Data Fig. 7a)."

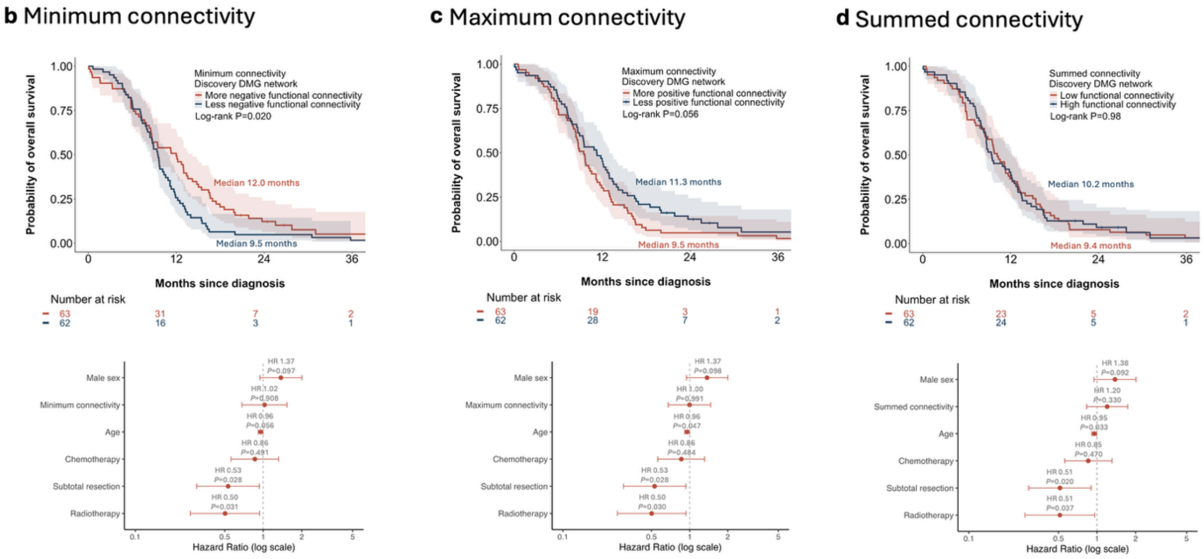

Extended Data Fig. 6b-d. The prognostic significance of DMG network-to-tumour connectivity is robust to methodological choices.
(B-D) *Kaplan-Meier survival curves and associated multivariable cox proportional hazard models constructed using external validation cohort data with network-to-tumour connectivity calculated on the discovery cohort derived DMG network.*

__(B)__ Minimum network-to-tumour connectivity is weakly non-prognostic but does not independently predict patient overall survival. Kaplan-Meier median overall survival 9.5 vs. 12.0 months; 95% CI 8.4-10.9 vs. 8.6-14.2 months; P=0.020. Multivariable cox HR 1.02; 95% CI 0.68-1.53; P=0.908.
__(C)__ Maximum network-to-tumour connectivity is weakly non-prognostic but does not independently predict patient overall survival. Kaplan-Meier median overall survival 9.5 vs. 11.3 months; 95% CI 8.4-11.1 vs. 8.9-13.0 months; P=0.056. Multivariable cox HR 1.00; 95% CI 0.68-1.47; P=0.991.
__(D)__ Summed network-to-tumour connectivity is non-prognostic. Kaplan-Meier median overall survival 9.4 vs. 10.2 months; 95% CI 8.6-12.2 vs. 8.5-12.2 months; P=0.980. Multivariable cox HR 1.20; 95% CI 0.83-1.74; P=0.330.

In all Kaplan-Meier curves, the statistical difference between groups was evaluated using the log-rank test with error bars representative of 95% CI. In all forest plots, mean values are represented by solid circles and whiskers represent 95% CI.

5. The extended data figures do not appear to be ordered chronologically with respect to when they are referenced in the paper. Please order the extended data figures to match when they are referenced in the main text.

Sorry for this inconsistency. We have comprehensively rewritten the manuscript to enhance readability and, in doing so, have ensured that Extended Data Figures / Tables and Supplementary Tables appear chronologically within the Main Text. We have also limited the volume of the Extended Data to 10 A4 items, in line with editorial guidelines.

6. Figure 3F - the heatmap is very poor quality. Is it not possible to use a more comprehensive/thorough visualization? It is very difficult to evaluate support for the conclusion that "Anatomically, tumor growth mapped to the DMG network with greater tumor intersection of network than non-network brain (Fig. 3f-g)" (lines 354 to 356) based on this figure. The plot in 3G helps, but an accompanying plot that shows e.g. the proportion of tumor growth into vs. outside of the DMG network across patients would provide more quantitative support for the qualitative results in Figure 3G (see also Major Comment 10). The same is true for Extended Data Figure 18, which only shows qualitative overlap at select slices for select example patients.

We agree that visualisation of these results could be improved. To this end, we have replaced the previously included heatmap with more comprehensive tumour network overlap maps at diagnosis and at final follow-up MRI for all 71 children with this paired data available (**Main Text Fig. 3a.).** As discussed above for Major Comment 10 by the same reviewer, we have also included a quantitative comparison of tumour growth into the DMG network vs. into the rest of the brain (**Main Text Fig. 3b.).**

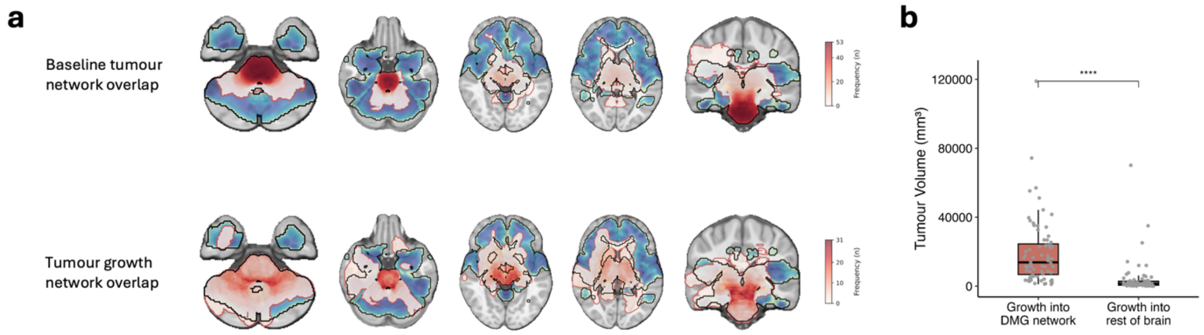

Fig. 3. Tumour growth follows DMG network trajectories.
__(A)__ Tumour-network overlap maps illustrate the anatomic distribution of DMG at baseline diagnostic MRI (top row) and new tumour growth from diagnostic to final MRI within two months of death (bottom row) for a subcohort of 71 children with paired MRI available for analysis. Tumour burden is plotted in red-white (red outline) as a summed heatmap representative of voxelwise frequency and DMG network values in blue-yellow (black outline).
__(B)__ Tumour volumetric growth within the DMG network was significantly greater than tumour volumetric growth out of the DMG network between diagnostic and final MRI (median 13,768 vs.

1,270 mm^3; 95% CI 11,293-17,763 vs. 773-1,792 mm^3; two-tailed paired Wilcoxon signed-rank test P=6.85e^{-13}). Patient-level tumour growth volumes are shown in grey.

These data represent analyses across all 71 patients with paired diagnostic and final follow-up MRI available for comparison, demonstrating a cohort-wide effect. To illustrate these findings at the patient-level, which may be more intuitive for some readers, we have also included a more comprehensive visualisation of tumour growth for the same four exemplar patients as previously shown, but taking care to present a greater number of axial slices as well as new coronal and sagittal sections, as below:

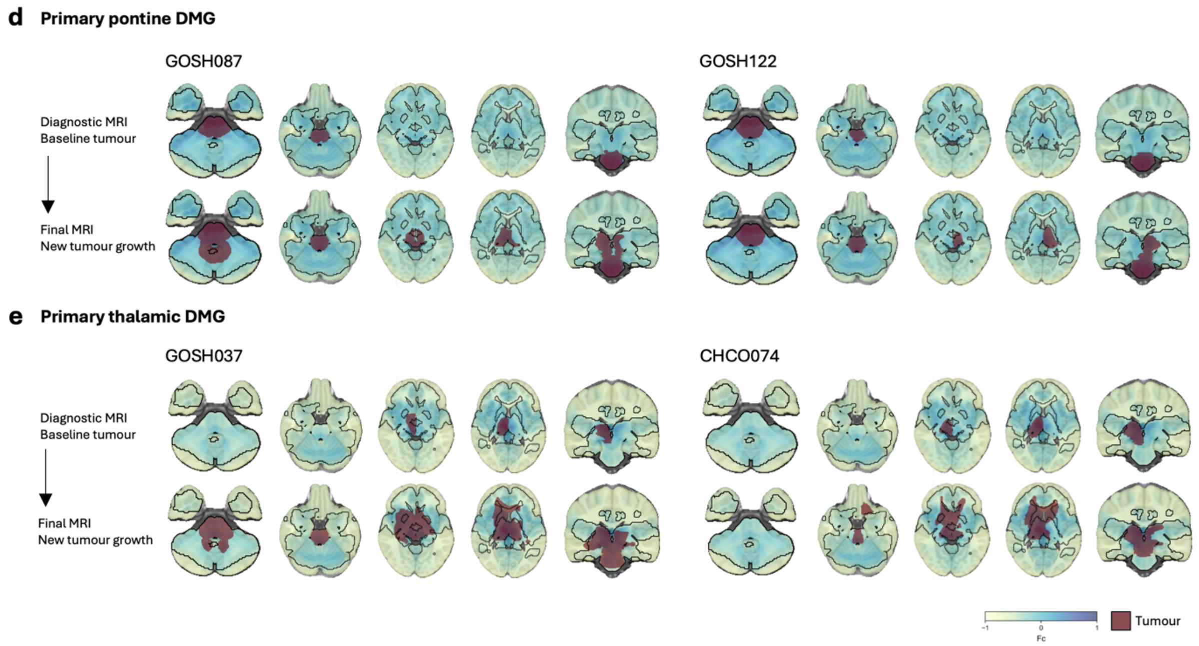

Fig. 3. Tumour growth follows DMG network trajectories.
(D,E) *Representative patient tumours at baseline diagnostic and final MRI across pontine* ***(D)*** *and thalamic* ***(E)*** *DMG (red) overlayed onto the DMG network (network peaks in dark blue, black outline). Tumour growth trajectories are plotted for each patient as a three-dimensional representation of tumour spread into DMG network nodes. GOSH087 and GOSH122 are illustrative of two pontine DMG, initially confined to the brainstem, which progressed to involve the thalami via the corticospinal tracts; GOSH087 also invaded the cerebellum. GOSH037 and CHCO74 are illustrative of two DMG, initially confined to the right thalami, which progressed to involve the orbitofrontal cortices, genu of the corpus callosum, contralateral thalami, deep grey matter, pallidum, and hippocampi. Both tumours also grew caudally: GOSH037 progressed through the midbrain, medulla, and pons to the cerebellum via the superior and middle cerebellar peduncles whilst CHCO074 progressed into the midbrain. These patterns of growth strongly aligned with DMG network connectivity, as shown.*

7. Figure 3E – this is not a very effective way to visualize the (lack of) differences in tumor volume between high vs. low connectomic resections since the distributions and central tendencies/dispersions are somewhat difficult to ascertain for each group. Box plots (as in Extended Data Fig. 11) would be more effective.

We agree that visualisation of resected tumour volume could be improved and, as mentioned by the same reviewer in a separate comment, that this should include both the percentage of tumour volume resected – as is typical for extent of resection – as well as the raw tumour volume resected. To this end, we have replaced this panel with two box plots with individual datapoints in grey, as below:

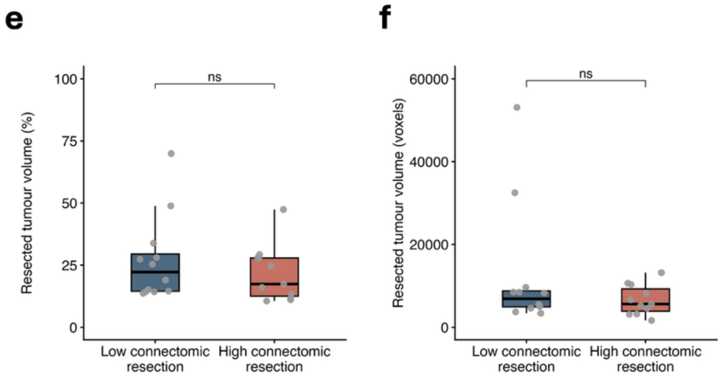

Main Text Fig. 6. Survival benefit of high connectomic resections in children with primary thalamic DMG.
(E-F) *No difference was identified in the volumetric percentage of tumour resected* ***(E****; median 22.2 vs. 17.4%; two-tailed Mann-Whitney U-test P=0.372****)*** *or in the raw volume of tumour resected* ***(F****; median 6,892 vs. 5,603 mm^3; two-tailed Mann-Whitney U-test P=0.479****)*** *when comparing low- and high-connectivity resections.*

8. Figure 3A-C takes up a lot of space but doesn't add much. It might be better off being moved to the supplement.

We agree that previous Main Text Fig. 3A did not add to the message of the paper and have removed it from the current manuscript. However, we have found that, when explaining the concept of "connectomic resections" to neuro-oncologists, neurosurgeons, and other clinicians who may be less familiar with human connectomic analyses, this figure has helped to clarify our methods and what is meant. Conversely yet similarly, when working with neuroscientists who have not encountered neurosurgical concepts, the same holds true. Given the important clinical implications of this result as the basis of a planned future clinical trial, we have retained the methodologic figures, as below, for clarity.

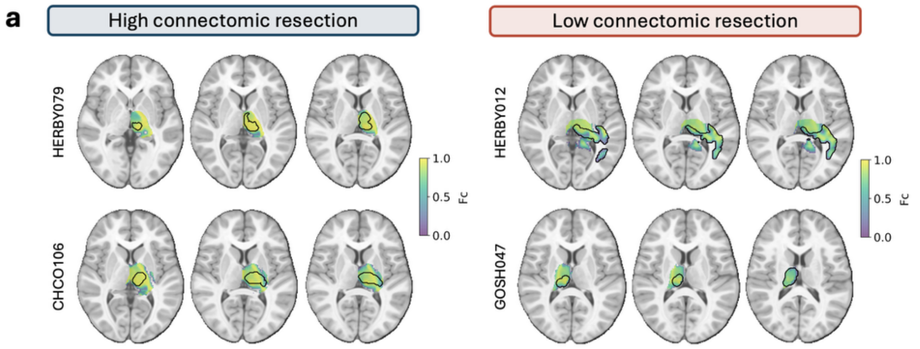

Main Text Fig. 6. Survival benefit of high connectomic resections in children with primary thalamic DMG. *(A) Tumour resection maps illustrate intratumoral, subregion-specific tumour tissue connectivity with the DMG network in children with thalamic DMG and high-connectomic or low-connectomic subtotal resections. High connectivity tumour subregions are depicted in yellow whilst low connectivity tumour subregions are depicted in dark blue. Resected tumour margins are defined in black ink.*

9. Figure 3H – I'm not sure that showing Spearman rank correlations as linear slope is valid since Spearman rank correlations reflect the monotonicity of the relationship, and so the linear slopes may not be accurate representations of the actual relationships. Why not just show the distribution of patient-level rho estimates along with the mean and 95% CIs using a single-axis chart (e.g. stacked dots with mean/95% CIs superimposed) instead?

Thank you for highlighting this statistical error. We have revised this figure to exactly fit the reviewer's recommendation, as below:

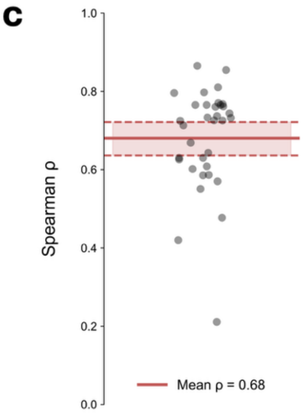

Main Text Fig. 3. Tumour growth follows DMG network trajectories. (C) *Tumour growth into pre-defined DMG network nodes exhibited a strong positive dependence with node-to-tumour connectivity, correcting for the distance of tumour infiltration to each network node (mean Spearman's ρ=0.68; 95% CI 0.63-0.72). All but two individual patient correlations were significant after Benjamini-Hochberg correction (P_{FDR}<0.05). Patient-level rho-values (ρ) are shown in grey with the group-level median in red with bootstrapped 95% CI.*

10. Figure 3I – it's not clear from the methods how the 16 DMG network nodes were defined. Are these parcels? Clusters? This should be clarified. I'm also not sure that this panel is really very informative. I think it would be more informative to show growth trajectories both within and outside of the DMG so that readers could evaluate the extent to which the growth trajectories are constrained to the DMG in the example patients.

Thank you for bringing this ambiguity to our attention. DMG network nodes were defined using FSL Cluster via an unsupervised clustering method (Smith … Matthews. *Neuroimage*. 2004.). We have revised the results to clarify this and have also highlighted these clusters in **Main Text Fig. 1d**., as previously discussed:

*"To examine the specificity of DMG growth to peaks of greatest connectivity in the DMG network, we restricted our follow-up cohort to 34 children with tumour progression beyond the primary site and computed the connectivity of each tumour with pre-defined DMG network **peak clusters** (**Fig. 1d,** Supplementary Table 2), controlling for the distance of each primary tumour to each network **peak**. Correlative analyses identified greater tumour volumetric burden in DMG network **peaks** with which each tumour was more functionally connected (Fig. 3c)."*

The methods have also been revised to further clarify this approach:

*"**Regions of peak prognostic significance in the DMG network were identified using an unsupervised, cluster identification algorithm via FSL Cluster[105] using a minimum voxel count threshold ≥20.[99] DMG network cluster** peak significance values, centre of gravity (COG) coordinates, **and neuroanatomic location, hereafter termed network 'nodes' or 'peaks', were reported.**[105]"*

Secondly, in line with the reviewers' suggestion and as previously shown, we have replaced all spider plots with (1) quantitative comparison of cohort-wide growth within and out of the DMG network and (2) more detailed tumour-network overlap maps comparing tumour anatomic growth with DMG network topography at diagnosis at final follow-up MRI across both the cohort and four individual patients. In these instances, we have taken care to provide greater anatomic visualisation.

11. Extended Data Figure 9 – Why does the unthresholded ABCD map have so many "empty" voxels? Shouldn't it span the entire brain like GSP and Yeo maps? Does this reflect a data quality issue in the ABCD sample? Also, there is no quantitative comparison here. Would the thresholded GSP/Yeo maps show significant similarity to the ABCD map using e.g. spin permutation testing? Alternately, qualitative support would be stronger if the analyses were performed in all 3 datasets and the thresholded maps were shown in more detail than 3 orthogonal slices.

Thank you for this important observation. We erroneously plotted the FWE-corrected TFCE DMG network (ABCD1000 result) alongside the FWE-corrected unthresholded voxelwise Yeo1000 and GSP1000 networks. We have revised these panels to present the unthresholded TFCE maps across all connectomes, as below. These are also spatially compared using both label and spin permutation tests, demonstrating significant similarity of both GSP1000 and Yeo1000 networks to the primary ABCD1000 DMG network (**Extended Data Fig. 5a-c.**). Beyond this, we also hypothesised that, if these networks were topographically similar to the DMG network, they should also stratify patients into survival risk groups. To this end, we replicated survival analyses for network-to-tumour connectivity using values derived from both the GSP1000 and Yeo1000 networks. These demonstrated clear and clinically meaningful survival differences between children with high and low DMG network connectivity, in line with our paper primary result (**Extended Data Fig. 5d-e.**). These results are shown below and are also included in the manuscript main text: 'Findings were also replicated across connectomes (**Extended Data Fig. 5d-e**).'

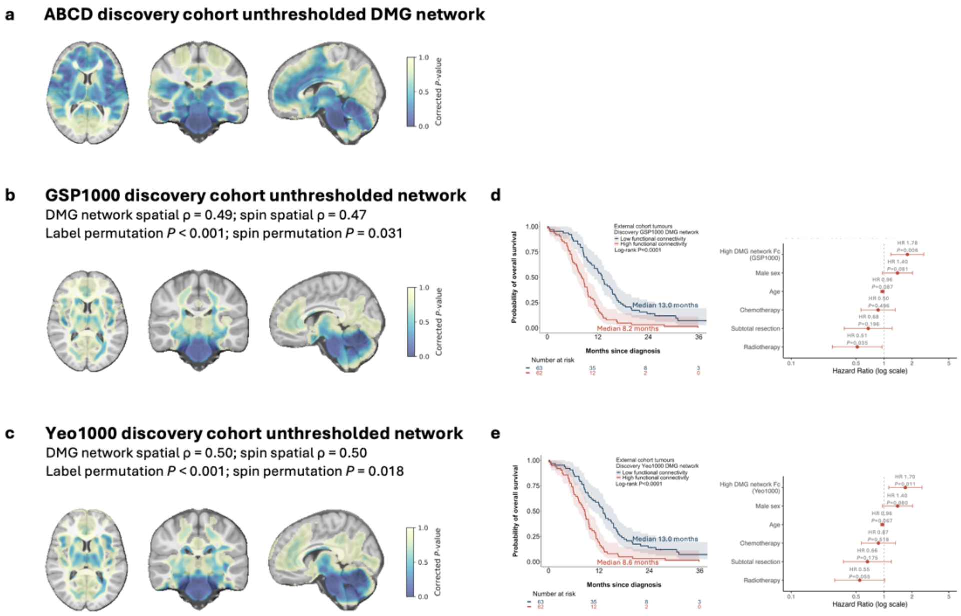

Extended Data Fig. 5. DMG network results and prognostic impact are robust to connectome. *The functional DMG network identified in our primary analysis* ***(Main Text Fig.1.)*** *was constructed from a resting-state fMRI dataset of 1000 healthy children aged 9±0.2 years (ABCD;* ***A****). Network peaks and spatial topography remain unchanged across two independent connectomes constructed from resting-state functional connectivity data from 1000 healthy adults.* ***(B)*** *GSP1000: spatial ρ=0.549, label permutation P<0.001; spin spatial ρ=0.638, spin permutation P<0.001.* ***(C)*** *Yeo1000: whole brain spatial ρ=0.546, label permutation P<0.001; spin spatial ρ=0.604, spin permutation P<0.001. Connectivity maps are shown unthresholded to facilitate comparison. Network-to-tumour connectivity was then computed for children in the external validation cohort across discovery cohort generated GSP1000 and Yeo1000 networks using identical methods as those for the primary, ABCD-derived DMG network. Kaplan-Meier survival curves demonstrate a clear and clinically meaningful survival difference when patients are stratified by network-to tumour connectivity whilst multivariable cox proportional hazards models confirm network-to-tumour connectivity as an independent predictor of patient overall survival* ***(D-E)****.* ***(D)*** *GSP1000: Kaplan-Meier median overall survival 8.2 vs. 13.3 months; 95% CI 7.1-9.5 vs. 11.1-15.5 months; P<0.0001. Multivariable cox HR 1.78; 95% CI 1.18-2.69; P=0.006.* ***(E)*** *Yeo1000: Kaplan-Meier median overall survival 8.6 vs. 13.0 months; 95% CI 7.2-9.6 vs. 10.6-15.5 months; P<0.0001. Multivariable cox HR 1.70; 95% CI 1.12-2.56; P=0.011.*

12. There is not sufficient context provided to frame the relevance/significance of the analyses focusing on neurotransmitter receptor and transporter densities across the DMG network. Even from an exploratory perspective, these results are interesting, but the conclusions suggest that they fit into broader knowledge about the conditions that give rise to DMG, and so it would be helpful if the authors could better explain how the observed results fit into what is known about the chemoarchitectural preferences of DMG. For example, while the Introduction mentions that DMG utilize glutamatergic/AMPA-receptor mediated and GABAergic, GABAa-receptor mediated synapses, little background is provided to suggest that findings related to cholinergic and serotonergic systems vs. dopaminergic systems would be relevant at all, much less support the conclusion that "tumor connectivity to the DMG network is not merely opportunistic but occurs within the context of an organized chemoarchitectural niche which DMG are known to exploit". It would be good to provide context for this claim to help readers who are less familiar with the known chemoarchitectural preferences of DMG better understand the significance of the results and the conclusions drawn from them.

Thank you for raising this. We agree that we should clarify our rationale for these experiments. To this end, we have added text to the introduction to state from the outset that DMG are known to exploit a diverse repertoire of synaptic signalling for growth, including cholinergic (Drexler … Monje. *Cell*. 2025.). and serotonergic (Drexler … Monje. & Byun … Monje. *Manuscripts in preparation*.) synapses – as below:

"Recent work has established DMG integration and communication with otherwise healthy neural circuits via both paracrine signalling (brain-derived neurotrophic factor and neuroligin-3 ***(NLGN3)****)[4,7,8] and bona fide, electrophysiologically-functional* ***neuron-to-glioma synapses across a diverse neurotransmitter repertoire including*** *glutamatergic, calcium-permeable AMPA-receptor mediated[3,4],* ***cholinergic M1/M3 receptor-mediated[5], and GABAergic GABAA-receptor-mediated[6]*** *neuron-to-glioma synapses. In animal models, depolarisation of glioma membranes directly drives tumour growth through voltage-dependent mechanisms which remain to be fully elucidated.[3,4] Neuronal cell soma may be local to or distant from and projecting towards malignant cells, with engagement of brain-wide neural populations in glioma circuits, activity from which acts as a putative driver of tumour progression.[9,12,13]* ***For example, long-range cholinergic projections from the midbrain pedunculopontine and laterodorsal tegmental nuclei, respectively, promote the circuit-dependent growth of pontine and thalamic DMG in preclinical models.[5] Though the impact of other neuromodulatory neurons and neurotransmitters on DMG growth remains to be fully explored, early preclinical reports have suggested that similar principles apply: for example, serotonergic projections from the dorsal and median raphe demonstrably promote the circuit-specific growth of DMG in the pons, thalamus, and cerebral cortex.[24] Eary reports also indicate that noradrenergic projections from the locus coeruleus promote DMG growth in preclinical models.[25]"***

We also tested this hypothesis at the tissue-level: collecting seven primary patient surgical samples and performing snRNA-seq to examine differential patterns of gene enrichment in neuroimaging-defined high- and low-connectivity DMG. These data are now included in **Main Text Fig. 4.** and demonstrate enrichment of glutamatergic, cholinergic, serotonergic, and noradrenergic gene signatures in high-connectivity DMG specifically. Our chemoarchitectural data follow from this to offer an orthogonal, brain-wide test of the same principle. We specifically hypothesised that the connectivity profile of the DMG network would include cholinergic and serotonergic projections, which we were able to test with dominance analyses of PET-derived whole-brain maps for different neurotransmitter systems. We have prefaced these chemoarchitectural analyses with the following:

"**These observations, and preclinical evidence for the robust regulation of DMG growth by diverse synaptic inputs[3,6], including neuromodulatory inputs[5,24], raise the question of whether the DMG network represents an organised functional architecture – composed of specific neurotransmitter projections – or more spuriously connected brain areas hijacked during tumour development.**"

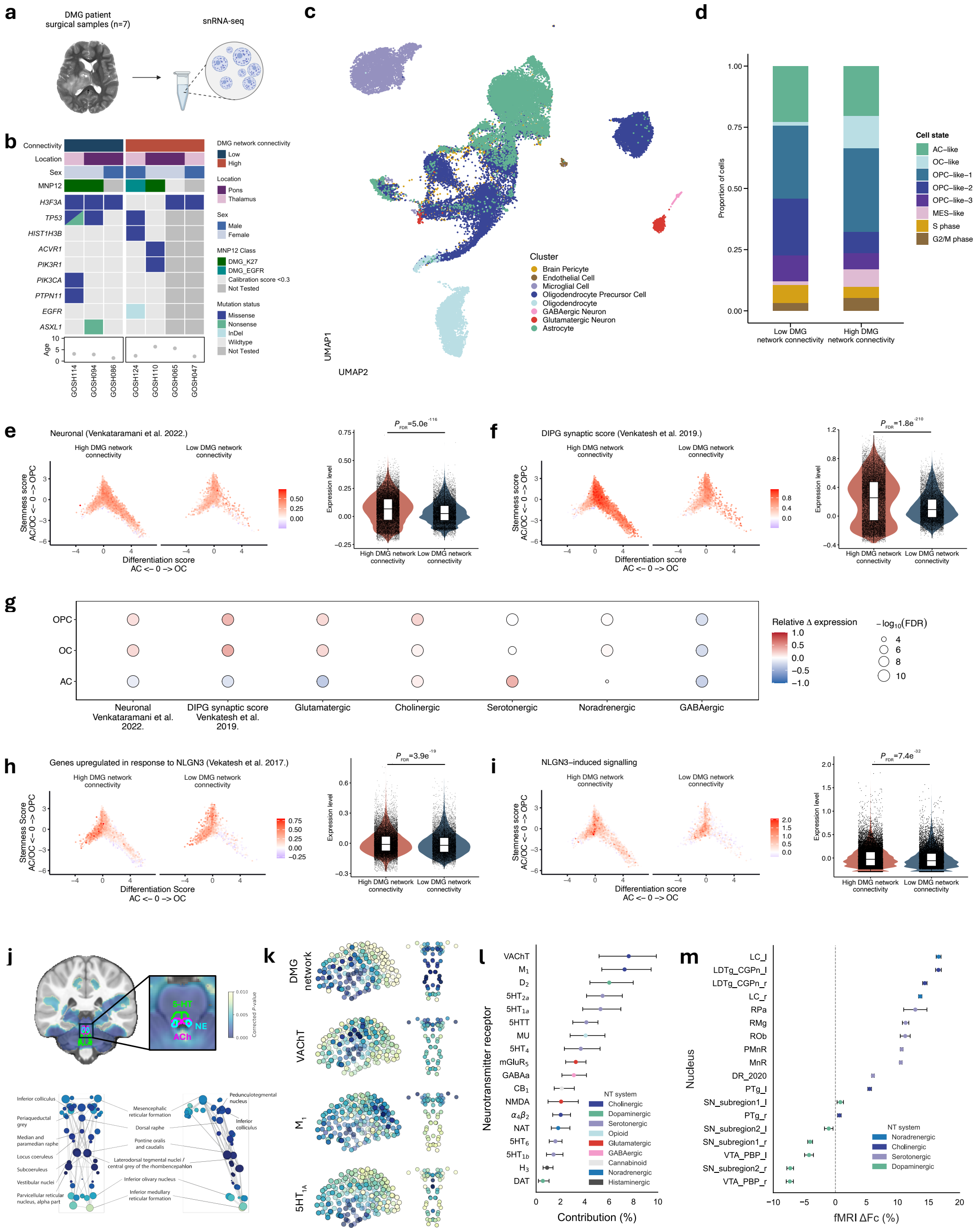

Fig. 4. Synaptic gene enrichment in high-connectivity DMG supports DMG network chemoarchitectural organisation.

(A) *Methodologic schema.*

(B) *snRNA-seq cohort characteristics (n=7) and tumour somatic mutations identified through paediatric solid tumour next-generation DNA panel sequencing.*

(C) *Uniform manifold approximation and projection (UMAP) of 42,504 single-nuclei and identified cell type clusters.*

(D) *Frequency of malignant cell states in high- and low-connectivity DMG.*

(E-F) *Malignant cell signature expression projected onto cell lineage (x-axis; astrocytic to oligodendroglial differentiation) and stemness (y-axis; stem to differentiated) scores with corresponding violin plots. The red-blue colour overlay indicates relative gene signature enrichment: neuronal* ***(E)*** *and DIPG synaptic score* ***(F)****.*

(G) *Dot plot representation of relative neurotransmitter gene signature enrichment in high- versus low-connectivity DMG.*

(H-I) *Malignant cell signature expression projected onto cell lineage (x-axis; astrocytic to oligodendroglial differentiation) and stemness (y-axis; stem to differentiated) scores with corresponding violin plots. The red-blue colour overlay indicates relative gene signature enrichment: genes upregulated in response to NLGN3* ***(H)*** *and genes encoding NLGN3-induced signalling protein* ***(I)****.*
(J) *Spatial representation of brainstem nuclei DMG network connectivity strength. Network peaks are depicted in dark blue. For visualisation, only the strongest 5% of brainstem nucleus-nucleus functional connections are shown.*
(K) *Top three brain-wide neurotransmitter receptor and transporter density distributions with greatest contribution to DMG network chemoarchitecture.*
(L) *Percentage neurotransmitter contribution to the overall fit of a multiple linear regression model between summed DMG network functional connectivity across parcellated brain regions and brainstem nuclei and 18 cortical neurotransmitter receptor and transporter density profiles (R^2_{adj}=0.39).*
(M) *Pairwise comparison of nucleus-to-tumour connectivity across short- and long-term survivors of DMG demonstrated significantly greater connectivity to cholinergic (P_{FWE}=0.001), serotonergic (P_{FWE}=0.0002), and noradrenergic (P_{FWE}=0.0004) nuclei than dopaminergic nuclei in short-term survivors (one-way ANOVA F-statistic=16.84, P=6.40e^{-05} with Tukey's post-hoc test; factor neurotransmitter system with four levels).*

Signature enrichment across malignant single-nuclei (n=32,543) was assessed using a two-tailed Wilcoxon signed-rank test.

13. It would be helpful if Figure 4H and Figure 4I were sorted to clarify whether specific systems/nuclei cluster together in terms of their contribution to the models. In general, though, this is an odd and somewhat difficult-to-parse way of visualizing regression/ANOVA results. Simple line or bar plots with CIs/error bars, either grouped by system/nuclei or rank-ordered and colored by system/nuclei, would honestly make it easier to parse and understand these results. I would also suggest splitting Figure 4 into 2 figures, with the FDG-PET results in Figure 4, the neurotransmitter system analyses in Figure 5, and the epigenetic analyses in Figure 6.

Thank you for this suggestion. We have re-plotted these figures as box and whisker plots given our aim to present an estimated value with corresponding confidence intervals. Both figures are also sorted and coloured by system which does better demonstrate cholinergic and serotonergic dominance (**Main Text Fig. 4l-m**):

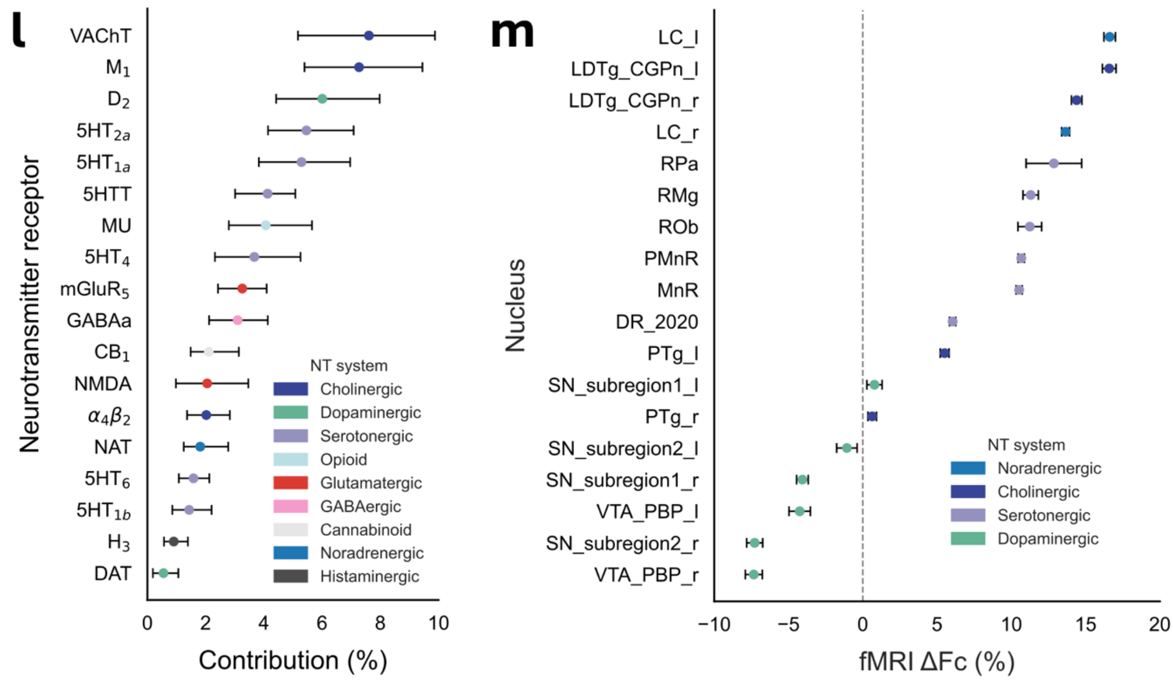

Fig. 4. Synaptic gene enrichment in high-connectivity DMG supports DMG network chemoarchitectural organisation.
(L) Percentage neurotransmitter contribution to the overall fit of a multiple linear regression model between summed DMG network functional connectivity across parcellated brain regions and brainstem nuclei and 18 cortical neurotransmitter receptor and transporter density profiles (R^2_{adj} = 0.39).
(M) Pairwise comparison of nucleus-to-tumour connectivity across short- and long-term survivors of DMG demonstrated significantly greater connectivity to cholinergic (P_{FWE}=0.001), serotonergic (P_{FWE}=0.0002), and noradrenergic (P_{FWE}=0.0004) nuclei than dopaminergic nuclei in short-term survivors (one-way ANOVA F-statistic=16.84, P=6.40e^{-05} with Tukey's post-hoc test).

To speak to the reviewer's second suggestion, we agree that these data and our data demonstrating and association between DMG incidence with age-wise changes in neurometabolic development are better presented in separate figures. We have now implemented this change: the chemoarchitectural data are presented alongside our snRNA-seq data (**Main Text Fig. 4.**) whilst the [^{18}F]FDG-PET data are presented as a standalone figure (**Main Text Fig. 5.**).

14. What were the factor levels in the one-way ANOVA? Neurotransmitter system? This should be clarified in the methods. The ANOVA F-test results should also be reported in addition to the post-hoc tests for completeness.

Thank you for highlighting this. The factor level was neurotransmitter system with four levels. We have clarified this in the manuscript methods as below:

"**To compare the connectivity profile of short-term and long-term survivors to brainstem neuromodulatory nuclei, mean nucleus-to-tumour connectivity values were computed and compared using a one-way ANOVA with Tukey's post-hoc test (factor neurotransmitter system with four levels: cholinergic, serotonergic, noradrenergic, and dopaminergic).**"

We have also clarified this in the legend of **Main Text Fig. 4m.** and included the ANOVA F-test results as suggested:

"Pairwise comparison of nucleus-to-tumour connectivity across short- and long-term survivors of DMG demonstrated significantly greater connectivity to cholinergic (P_{FWE}=0.001), serotonergic (P_{FWE}=0.0002), and noradrenergic (P_{FWE}=0.0004) nuclei than dopaminergic nuclei in short-term survivors (**one-way ANOVA F-statistic=16.84, P=6.40e^{-05} with Tukey's post-hoc test; factor neurotransmitter system with four levels**)."

15. The methods say that sample sizes are reported for all statistical tests, but the sample sizes for specific analyses are often not unambiguously clear from the text and/or figures. Please make sure that sample sizes and relevant details about samples under analysis (e.g. whether a model was trained in one sample and applied to the other) are unambiguously clear from the main text and figures.

We thank the reviewer for this comment and have revised all main text and extended data figure legends to ensure sample sizes are now clearly stated. We also agree with the previous comment regarding the dense style of our initial manuscript and have, therefore, comprehensively re-written the manuscript main text to ensure clarity of analytic method throughout.

16. For the analyses described in line 335-337 do you have absolute tumor resection volume to use as a covariate instead of the percentage of tumor resected?

Yes. We have replicated these analyses using both the percentage of tumour resected and the absolute volume of tumour resected with identical results, as below:

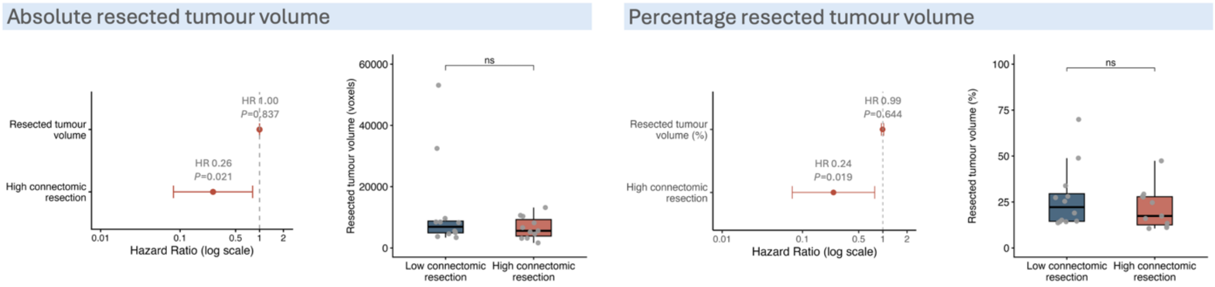

Adapted from Main Text Fig. 6. Survival benefit of high connectomic resections in children with primary thalamic DMG.
Results of multivariable cox proportional hazards models demonstrate that the prognostic impact of high connectomic resection in children with thalamic DMG is independent of extent of resection (percentage of tumour resected: HR 0.24; 95% CI 0.07-0.79; P=0.019 | absolute tumour volume resected: HR 0.26; 95% CI 0.08-0.82; P=0.021). Mean values are represented by solid circles and whiskers represent 95% CI. No difference was identified in the volumetric percentage of tumour resected (median 22.2 vs. 17.4%; two-tailed Mann-Whitney U-test P=0.372) or in the absolute volume of tumour resected (median 6,892 vs. 5,603 mm^3; two-tailed Mann-Whitney U-test P=0.479) when comparing low- and high-connectivity resections.

A percentage is, however, typically used to measure extent of resection in the neurosurgical and neuro-oncological literature given the fact that absolute volume does not differentiate a small resection of a large tumour and a large resection of a small tumour, both of which may be equal in volume (e.g. Hatoum … Weil. *JAMA Netw Open*. 2022.; Karschnia … Tonn. *Neuro Oncol*. 2022.). We have, therefore, retained our original cox proportional hazards result incorporating the percentage of tumour volume resected as a covariate but have included the bar chart comparisons of both the percentage of tumour resected and the absolute tumour volume resected between low- and high-connectomic subgroups in revised **Main Text Fig. 6.**

17. In line 367 – I may have missed it, but was there an analysis that directly evaluated the relationship between tumor volume and survival, or was this just done in the context of using lesion volume within network analyses? It would be surprising to me if tumor volume is not associated with survival, and I'm not sure that using it as a covariate in network analyses is a clear demonstration that there is no association of tumor volume and survival.

Yes. We used univariate cox proportional hazards models to test this though agree with the reviewer that the result of this analysis should be more clearly reported in the manuscript. Our analyses failed to identify as association between tumour volume and overall survival (univariate cox hazard ratio 1.00; *P*=0.569), in line with recent clinical literature (Jansen … Vuurden. *Neuro Oncol*. 2015.; Tam … Yeom. *Neuro Oncol Adv*. 2021.). We have included additional text in the results to highlight this finding:

Here, we perform tumour network mapping with the aim of identifying common network connections associated with DMG patient short-term survival, irrespective of tumour primary pontine or thalamic location. ***Greater tumour volume was not associated with patient overall survival (univariate cox hazards ratio 1.00; P=0.569), in keeping with previous clinical reports[47,48], and so tumour network mapping analyses were performed controlling for volume, in line with previous work.[40,43,45]*** *Given our aim to identify polysynaptic connections between spatially disparate brain regions in addition to direct structural connections through organised white-matter tracts, whole-brain-to-tumour connectivity analyses were performed across paediatric resting-state functional connectivity (functional MRI; fMRI)[49] and structural connectivity (diffusion-weighted MRI; dMRI)[50,51] data.*

Reviewer comments & author response:
‘A prognostic human brain network for diffuse midline glioma’

Reviewer reports

Referee #1 (Remarks to the Author):

The extensive revision by the authors is appreciated though a critical component must be addressed first. In one of their responses they have written "We, therefore, take an inclusive approach to the inclusion of children with pontine DMG, also including those children who meet established clinical-radiological diagnostic criteria for diffuse intrinsic pontine glioma and who disease was in keeping with short-term (<18 month) overall survival."

Despite being an inclusive approach, it does not meet any consensus diagnostic criteria for diffuse midline glioma and would instead would be diffuse intrinsic pontine glioma so I would ask that data then be reclassified as DIPG throughout or only histopathologically confirmed diagnostic tissue cases are included for analysis.

Referee #2 (Remarks to the Author):

Thank you for the detailed responses and additional analyses performed. These are extensive, thoughtful and improve the robustness of the results. Methodological and analytical comments have been addressed in as far as feasible given data available to the authors and within publicly released datasets.

Ultimately all three reviewers highlighted reservations around the extent to which the data can be interpreted as evidence that neural activity is the critical factor modulating tumour growth. Acknowledging the more nuanced discussion in the revised manuscript and overall emphasis on identification of a prognostic network, a tempering limitation remains that the data can neither prove nor disprove this central mechanistic hypothesis, although the findings provide further compelling indications and avenues for research.

Referee #3 (Remarks to the Author):

I co-reviewed this manuscript with one of the reviewers who provided the listed reports.

Referee #5 (Remarks to the Author):

The authors were very comprehensive in their responses to my prior comments. My remaining questions are minor and mostly seek clarification.

- The text indicates that 5-fold cross-validation was used to evaluate the predictive utility of network-to-tumor connectivity, but states that within each fold, models were trained on 60% of the data and tested on the held-out 40%. Normally, 5-fold cross-validation would train a model on 80% of the data (4/5 folds) and test the model on the held out 20% of the data (i.e. held out fold). This seems to actually be 5 repetitions of a 60/40 hold-out cross-validation, not a five-fold cross-validation. Please clarify and/or correct this.

- How are Freedman-Lane permutations performed for a Liebermeister test? Freedman-Lane permutation tests involve residualizing the outcome with respect covariates and then permuting the residuals (e.g. Winkler et al., 2014). How can this be accomplished with categorical outcomes while maintaining the category labels? Looking at the NiiStat code, it does not appear that covariates are included in Liebermeister test analyses or associated permutation tests (e.g. see https://github.com/neurolabusc/NiiStat/blob/master/nii_stat_core.m - functions lieber_permSub() and lieberSub(). It seems like these analyses may not actually control for lesion size based on evaluation of the code. Can you confirm whether these analyses actually controlled for lesion size, and if so, how?

- Relatedly, how are Freedman-Lane permutations performed for a Support Vector Classifier? The Freedman-Lane approach is specifically designed for the general linear model, and doesn’t seem like

it would work for an SVC. Indeed, the NiiStat SVM function seems to regress the lesion volume out of the lesion data, which is not consistent with Freedman-Lane (https://github.com/neurolabusc/NiiStat/blob/master/nii_stat_svm.m). Please verify that statistical methods are accurately described.

- How were multiple comparisons corrections accomplished for the VLSM and SVC-VLSM analyses? If voxel-level permutations were used, is 10,000 iterations enough (i.e. the smallest p-value possible would be 1/10001)? Voxel-wise permutation methods are known to be overly conservative (e.g. Mirman et al., 2018 - https://pubmed.ncbi.nlm.nih.gov/28847712/). Why not just use the same approach and correction method as used for the LNM analyses (i.e. PALM)?

- The description of the predictive analyses is somewhat unclear. If I understand correctly, it appears that the network was defined at the group level using the full discovery cohort, and then 5 repeat hold-out analyses (60/40 splits) were performed using one of the independent validation cohorts in which a training set partitioned from the validation cohort was used to define a median risk-cut off, which was then applied to the held-out test set also partitioned from the validation cohort before finally fitting the multivariable cox proportional hazards models in the held-out test set. For a “true” predictive approach, the entire modeling approach, from definition of the DMG network to the fitting the proportional hazards model, should ideally be performed in the training set, and then the fully parametrized model should be applied to the test set to obtain predictions that can be compared to the observed outcomes. This could be done using models that only include the clinical/demographic covariates, and these could be compared against models that also include DMG network connectivity in terms of their out-of-sample prediction performance. I can see an argument for fitting the proportional hazards model in the validation dataset, but this does result in the analyses not being a “true” predictive analysis because the validation set is being used to parametrize the proportional hazards model. The approach should be clarified in the text.

- For the analysis of tumor growth into the DMG network, a Mann-Whitney U-test was performed to compare tumor volume within vs. outside of the network. This appears to be the incorrect statistical test since these are dependent measures (i.e. for each patient, the tumor growth inside the network is being compared against the tumor growth outside of the network). A signed rank test would be more appropriate for dependent samples. This is also relevant for the analysis of the spinal DMG cohort.

- For the analysis assessing concordance of functional connectivity and tumor burden per node, the text states that “the voxelwise degree of tumour overlap with each of the 18 DMG network nodes was computed for each tumour progression map, defined as the percent overlap of tumour volume relative to the volume of each network node mask”. It is unclear whether this means that the overlap was computed as the percentage of the tumor that occupied each node, or as the percentage of each node that was occupied by each node. These are distinct measures, and so it should be clear which is being used.

- For the analyses referenced in the above comment, the text states that the connectivity values were distance-corrected, but it does not describe how this correction was performed. This should be clearly stated in the text.

- The authors seem to use at least 2 different parcellations (e.g. MINCI 60MB SynthSeg and Schaefer-Yeo 400 region parcellation + Brainstem Navigator). It’s not clear why a single parcellation was not used. Also, does the Schaefer-Yeo + Brainstem Navigator parcellation include parcels for subcortical regions other than the brainstem?

- The Statistical Analyses section states that sample sizes are reported for all analyses, but this is not always the case (at least, the sample sizes are not always clear from the text). Please make sure that sample sizes are clear from the text, and that it is also clear which samples are used for which analyses (e.g. there are portions where it is unclear whether an analysis is performed in the discovery cohort vs. one or more of the independent validation cohorts).

- Extended Data Fig. 3 – the color bar for panel (N) seems inconsistent with the colormap.

- The network overlays shown in Extended Data Fig. 5 appear to be thresholded, but the labels say they are unthresholded.

Author response to reviewer comments

We thank the editor and reviewers for their continued engagement and feedback. We are grateful for their recognition of the conceptual innovations of our study and for their thoughtful and rigorous evaluations during this second round which have helped us to improve the clarity of the presented work. We are pleased to have incorporated editorial and stylistic suggestions whilst addressing all remaining reviewer concerns with point-by-point responses provided below. Specific changes to the manuscript text are quoted and highlighted in **bold** ink.

Referee #1 (Remarks to the Author):

The extensive revision by the authors is appreciated though a critical component must be addressed first. In one of their responses they have written "We, therefore, take an inclusive approach to the inclusion of children with pontine DMG, also including those children who meet established clinical-radiological diagnostic criteria for diffuse intrinsic pontine glioma and who disease was in keeping with short-term (<18 month) overall survival."

Despite being an inclusive approach, it does not meet any consensus diagnostic criteria for diffuse midline glioma and would instead would be diffuse intrinsic pontine glioma so I would ask that data then be reclassified as DIPG throughout or only histopathologically confirmed diagnostic tissue cases are included for analysis.

We sincerely thank the reviewer for their evaluation of our revised manuscript. We agree that diagnostic clarity is paramount and have, therefore as requested, reclassified all non-biopsied pontine tumours as DIPG throughout the manuscript. Further to this, we have also revised the manuscript methods and main text statement copied above, to read as below.

Manuscript methods

"Inclusion criteria were identical across cohorts:

1. *Consensus clinical-radiological diagnosis of **DIPG**, defined as the rapid (<3 month) onset of cranial nerve palsies, long-tract signs, or cerebellar signs accompanied by MRI identification of an expansile, diffusely infiltrative mass arising from and involving ≥50% of the pons and which is T1-hypo/iso-intense, T2-hyperintense, and lacks or has minimal contrast-enhancement.[30–32]*

OR

2. *Neuropathological (tissue biopsy) diagnosis of a primary pontine or thalamic **DMG**, H3K27-altered, in line with WHO 2021 criteria.[1]"*

Manuscript main text

*"We first studied a discovery cohort of 125 children aged <18 years with **primary pontine DMG/DIPG (n=106) or thalamic DMG (n=19)** treated at Great Ormond Street Hospital for Children (GOSH; Fig. 1a; Supplementary Table 1). As pontine biopsies were historically not performed[29], children meeting clinical diagnostic criteria for DIPG[30–32] and with a disease course in keeping with short-term (<18 month)[33] overall survival were included. **In keeping with the World Health Organisation (WHO) 2021 definition of the disease[1], biopsied tumours are classified as DMG, H3K27-altered, whilst non-biopsied pontine tumours are classified as DIPG. Throughout, reproducibility of findings is demonstrated for the combined (DMG/DIPG) patient cohort and across restricted cohorts of children with biopsied pontine or thalamic DMG, H3K27-altered.**"*

Referee #2 (Remarks to the Author):

Thank you for the detailed responses and additional analyses performed. These are extensive, thoughtful and improve the robustness of the results. Methodological and analytical comments have been addressed in as far as feasible given data available to the authors and within publicly released datasets.

Ultimately all three reviewers highlighted reservations around the extent to which the data can be interpreted as evidence that neural activity is the critical factor modulating tumour growth. Acknowledging the more nuanced discussion in the revised manuscript and overall emphasis on identification of a prognostic network, a tempering limitation remains that the data can neither prove nor disprove this central mechanistic hypothesis, although the findings provide further compelling indications and avenues for research.

We thank the reviewer for their recognition of the extensive analyses presented in this manuscript and agree that the data presented do not provide causal evidence for the hypothesis that neural activity modulates tumour growth in humans. Rather, as the reviewer notes, the primary contribution of this work is the definition and multimodal validation of a clinically relevant prognostic brain network. The spatial organisation, functional properties, and biologic associations of this network are consistent with a growing body of mechanistic preclinical evidence which demonstrates a fundamental role of neural activity in DMG tumour progression. In this way, our findings extend and begin to translate the preclinical literature by providing convergent evidence in children and by generating testable hypotheses for future early-phase interventional studies and clinical trials. Confirmation of neural activity as a causal driver of tumour progression in children will require direct ground-truth neural recordings or perturbational studies. We view the present work as providing a strong rationale and empiric foundation for such studies, while refraining from causal inference and noting this explicitly in the main text discussion:

'*Finally, none of the experiments presented herein demonstrate nor could have demonstrated a direct relationship between tumour neural activity and tumour growth in children.*'

Referee #3 (Remarks to the Author):

I co-reviewed this manuscript with one of the reviewers who provided the listed reports.

We thank the reviewer for their continued and constructive evaluation of our work.

Referee #5 (Remarks to the Author):

The authors were very comprehensive in their responses to my prior comments. My remaining questions are minor and mostly seek clarification.

We thank the reviewer for their thorough and thoughtful review from the previous round of revision. Addressing these statistical comments has notably improved the analyses presented and we are grateful for this additional input.

- The text indicates that 5-fold cross-validation was used to evaluate the predictive utility of network-to-tumor connectivity, but states that within each fold, models were trained on 60% of the data and tested on the held-out 40%. Normally, 5-fold cross-validation would train a model on 80% of the data (4/5 folds) and test the model on the held out 20% of the data (i.e. held out fold). This seems to actually be 5 repetitions of a 60/40 hold-out cross-validation, not a five-fold cross-validation. Please clarify and/or correct this.

- The description of the predictive analyses is somewhat unclear. If I understand correctly, it appears that the network was defined at the group level using the full discovery cohort, and then 5 repeat hold-out analyses (60/40 splits) were performed using one of the independent validation cohorts in which a training set partitioned from the validation cohort was used to define a median risk-cut off, which was then applied to the held-out test set also partitioned from the validation cohort before finally fitting the multivariable cox proportional hazards models in the held-out test set. For a "true" predictive approach, the entire modeling approach, from definition of the DMG network to the fitting the proportional hazards model,

should ideally be performed in the training set, and then the fully parametrized model should be applied to the test set to obtain predictions that can be compared to the observed outcomes. This could be done using models that only include the clinical/demographic covariates, and these could be compared against models that also include DMG network connectivity in terms of their out-of-sample prediction performance. I can see an argument for fitting the proportional hazards model in the validation dataset, but this does result in the analyses not being a "true" predictive analysis because the validation set is being used to parametrize the proportional hazards model. The approach should be clarified in the text.

We agree with the reviewer that our predictive modelling approach required clarification and revision to ensure a true predictive framework. We have now implemented these additional analyses and address both reviewer comments below. First, we have now adopted a fully external validation framework rather than relying on internal cross-validation. To this end, we define the DMG network exclusively using the discovery cohort and estimate all cox proportional hazards model parameters within this same cohort. Two models were fitted for overall survival exclusively within the discovery cohort:

1. Multivariable clinical model (age, sex, tumour location, extent of resection, and adjuvant radiotherapy and chemotherapy).
2. Multivariable combined clinical-connectivity model with DMG network-to-tumour connectivity and the above listed clinical covariates.

For each model, cox regression coefficients (β values) were frozen and the fitted (parameterised) model applied to the independent external validation cohort, generating per-patient overall survival estimates. Prognostic discrimination was quantified using the concordance index (C) and the incremental predictive value of network-to-tumour connectivity was assessed as the difference in concordance between combined and clinical-only models (ΔC). Statistical uncertainty was estimated using non-parametric bootstrap with 2000 iterations.

When applied to the independent validation cohort, the inclusion of DMG network-to-tumour connectivity significantly improved model predictive performance beyond clinical variables alone (concordance index 0.621 vs. 0.577; ΔC=0.044, 95% CI 0.016–0.074, *P*=0.002). In summary, implementation of this predictive framework confirms that DMG network-to-tumour connectivity adds additional and independent prognostic information to that obtained from routine clinical variables. The manuscript main text and methods have been updated as below to reflect this.

Manuscript methods

"To confirm out-of-sample prediction, we also fitted clinical-only and combined clinical-connectivity multivariable cox proportional hazards models exclusively on the discovery cohort incorporating patient age and sex and tumour location, DMG network-to-tumour connectivity, extent of resection, and adjuvant radiotherapy and chemotherapy. Model coefficients were subsequently frozen and the fully parameterised models applied to generate overall survival estimates across the independent external validation cohort. Prognostic discrimination was quantified using the concordance index (C) and the incremental predictive value of network-to-tumour connectivity was assessed as the difference in concordance between combined and clinical-only models (ΔC). Statistical uncertainty around ΔC was estimated using non-parametric bootstrap resampling of patients within the external cohort with 2000 iterations."

Manuscript main text

"Having established network-to-tumour connectivity as a robust correlate of patient overall survival, we next sought to confirm its predictive power and incremental prognostic value beyond standard clinical variables. To this end, ***we fitted clinical only and combined clinical-connectivity multivariable cox proportional hazards models on all patients included in the discovery cohort and applied the fully parameterised models with frozen coefficients to the independent external validation cohort, comparing model discriminatory power. In the external validation cohort, the inclusion of network-to-tumour connectivity improved prognostic discrimination relative to the inclusion of clinical variables only (concordance index 0.621 vs. 0.577; ΔC=0.044, 95% CI 0.016–0.074, P=0.002), confirming that network-to-tumour connectivity provides independent prognostic information beyond established clinical factors."***

- How are Freedman-Lane permutations performed for a Liebermeister test? Freedman-Lane permutation tests involve residualizing the outcome with respect covariates and then permuting the residuals (e.g. Winkler et al., 2014). How can this be accomplished with categorical outcomes while maintaining the category labels? Looking at the NiiStat code, it does not appear that covariates are included in Liebermeister test analyses or associated permutation tests (e.g. see https://github.com/neurolabusc/NiiStat/blob/master/nii_stat_core.m - functions lieber_permSub() and lieberSub(). It seems like these analyses may not actually control for lesion size based on evaluation of the code. Can you confirm whether these analyses actually controlled for lesion size, and if so, how?

- Relatedly, how are Freedman-Lane permutations performed for a Support Vector Classifier? The Freedman-Lane approach is specifically designed for the general linear model, and doesn't seem like it would work for an SVC. Indeed, the NiiStat SVM function seems to regress the lesion volume out of the lesion data, which is not consistent with Freedman-Lane (https://github.com/neurolabusc/NiiStat/blob/master/nii_stat_svm.m). Please verify that statistical methods are accurately described.

- How were multiple comparisons corrections accomplished for the VLSM and SVC-VLSM analyses? If voxel-level permutations were used, is 10,000 iterations enough (i.e. the smallest p-value possible would be 1/10001)? Voxel-wise permutation methods are known to be overly conservative (e.g. Mirman et al., 2018 - https://pubmed.ncbi.nlm.nih.gov/28847712/). Why not just use the same approach and correction method as used for the LNM analyses (i.e. PALM)?

We thank the reviewer for raising these important methodological points and for the opportunity to clarify our analytic approach. We address the above three comments together and believe that our revised analyses and more precise wording improve the quality of the presented work.

For both Liebermeister testing and support vector classification (SVC), non-parametric label permutation testing with correction for family-wise error (FWE) based on the maximum statistic across voxels was used and implemented in NiiStat, rather than Freedman-Lane permutation testing. We also confirm that lesion volume was – incorrectly – not controlled for in Liebermeister testing as this method does not accommodate the inclusion of nuisance covariates. We apologise for the inaccurate description of these analyses in the Methods.

In light of these limitations, we have replaced Liebermeister testing with a voxelwise general linear model (GLM)-based analysis, in which short- vs. long-term survivorship was modelled as a binary regressor and tumour volume included as a covariate, thereby more closely mirroring our analytic approach for tumour network mapping. Statistical inference was performed with 2000 Freedman-Lane permutations and FWE correction implemented in NiiStat, in line with best practice recommendations and previous work from our group (Winkler *et al*. *Neuroimage*. 2014.; Sperber *et al*. *Hum Brain Mapp*. 2017.; Karnath *et al*. *Neuroimage*. 2018.; Stark. User Manual and Tutorial for NiiStat. 2018.; Schaper *et al*. *JAMA Neurol*. 2023.). Further, as the reviewer suggested and to further ensure the consistency of VLSM with the methods employed for tumour network mapping, we replicated univariate VLSM in PALM with threshold-free cluster enhancement (TFCE). Whilst results of both voxelwise FWE-corrected (NiiStat) and TFCE FWE-corrected (PALM) analyses have been included for visualisation, TFCE results have been prioritised and carried forward for exploratory survival analyses (Extended Data Fig. 1i,k), enabling direct head-to-head comparison of the prognostic value of tumour location vs. DMG network information (Extended Data Fig. 7b).

Finally, to further improve control for lesion volume in our multivariate VLSM approach, we revised our methods to implement support vector regression in SVR-LSM, a widely-used toolbox for multivariate and cluster-based VLSM, in which short- vs. long-term survivorship was modelled as a binary regressor while controlling for lesion volume using direct total lesion volume control (dTLVC) and correcting for multiple testing, similarly in line with best-practice recommendations for multivariate VLSM and previous work from our group (Mah *et al*. *Brain*. 2014.; DeMarco & Turkeltaub. *Hum Brain Mapp*. 2018.; Schaper *et al*. *JAMA Neurol*. 2023.). The revised, subthreshold SVR-VLSM map has been carried forward for exploratory survival analyses (Extended Data Fig. 1j,l) and for direct head-to-head comparison with DMG network connectivity prognostic information (Extended Data Fig. 7c).

Finally, we have also taken the opportunity to clarify in the manuscript methods that, when pontine and thalamic tumours were split into distinct subcohorts for univariate VSLM, we restricted analyses to voxels contributed by ≥7 patients to avoid single-patient-driven effects, particularly in the thalamic DMG patient cohort (total *n*=19), in line with best-practice recommendations for VLSM (Sperber *et al*. *Hum Brain Mapp*. 2017.; Karnath *et al*. *Neuroimage*. 2018.; Stark. User Manual and Tutorial for NiiStat. 2018.).

We have now updated the manuscript Methods and referenced Figures to reflect this revised approach, as below.

Manuscript methods

"Identifying tumour voxels associated with patient overall survival

Tumour location frequency maps were plotted in MNI space as the sum of all binary tumour masks using ANTs iMath functions. To establish whether tumour anatomic location alone was associated with patient overall survival, we first implemented a univariate voxel-based lesion symptom mapping (VLSM) approach. A voxelwise ***general linear model with permutation testing was applied to the data to compare voxels between short- and long-term survival groups in NiiStat*** *(https://github.com/neurolabusc/NiiStat).[32,77] Analyses were limited to voxels involved in ≥5% tumours, controlling for tumour volume and* ***correcting for*** *multiple comparisons.[77–80] Significance at each voxel was assessed using 2000 Freedman-Lane (shuffled) permutations.* ***Given the conservative nature of voxel-based permutation testing[81], we replicated univariate VLSM by further implementing threshold-free cluster enhancement (TFCE) in FMRIB Software Library (FSL) Permutation Analysis of Linear Models (PALM).[78,82] Given the significantly shorter overall survival in children with pontine versus thalamic DMG, we also performed univariate VLSM across pontine DMG/DIPG and thalamic DMG separately to control for the prognostic effect of primary tumour location. In each of these analyses, we limited VLSM to voxels involved in ≥7 patient tumours to avoid single case-driven effects, in line with best-practice recommendations.[77,79,80] We replicated*** *analyses implementing multivariate support vector* ***regression*** *(SV****R****)-VSLM, which can identify complex dependencies between neighbouring voxels that traditional univariate VLSM approaches cannot.[83,84] SV****R****-VSLM* ***was implemented in SVR-LSM[85], a Matlab toolbox for multivariate and cluster-based lesion symptom mapping, and*** *limited to voxels involved in ≥5% tumours, controlling for tumour volume* ***using direct total lesion volume control (dTLVC)*** *and* ***correcting for*** *multiple comparisons. Significance was assessed using 10,000 permutations. Results of univariate VLSM and multivariate SV****R****-VLSM were considered significant at two-tailed family-wise error (FWE) corrected P_{FWE}<0.05 and <0.005, respectively, as in previous work.[32]"*

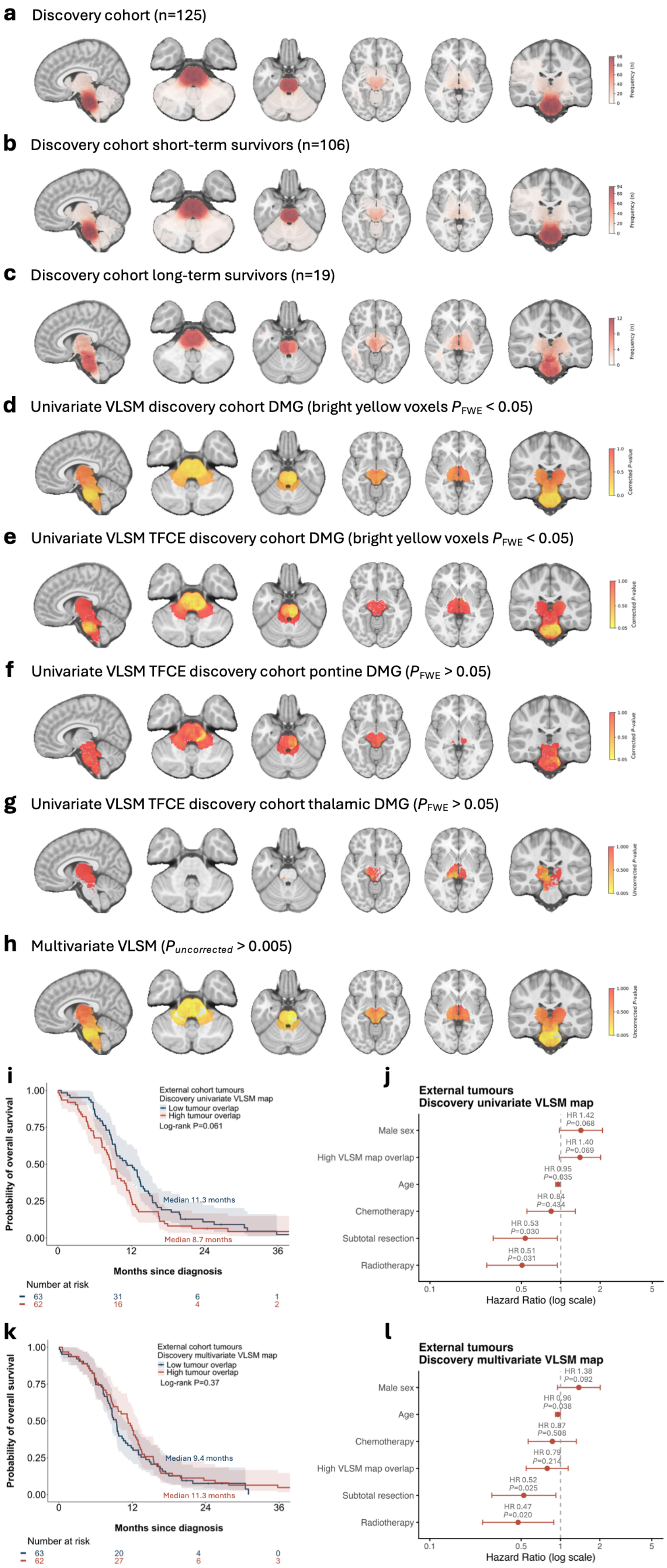
a Discovery cohort (n=125)
b Discovery cohort short-term survivors (n=106)
c Discovery cohort long-term survivors (n=19)
d Univariate VLSM discovery cohort DMG (bright yellow voxels $P_{FWE} < 0.05$)
e Univariate VLSM TFCE discovery cohort DMG (bright yellow voxels $P_{FWE} < 0.05$)
f Univariate VLSM TFCE discovery cohort pontine DMG ($P_{FWE} > 0.05$)
g Univariate VLSM TFCE discovery cohort thalamic DMG ($P_{FWE} > 0.05$)
h Multivariate VLSM ($P_{uncorrected} > 0.005$)
Frequency (n)
Corrected P-value
Uncorrected P-value
i
External cohort tumours
Discovery univariate VLSM map
Low tumour overlap
High tumour overlap
Log-rank P=0.061
Median 11.3 months
Median 8.7 months
Probability of overall survival
Months since diagnosis
Number at risk
63 31 6 1
62 16 4 2
j
External tumours
Discovery univariate VLSM map
Male sex HR 1.42 P=0.068
High VLSM map overlap HR 1.40 P=0.069
Age HR 0.95 P=0.035
Chemotherapy HR 0.84 P=0.434
Subtotal resection HR 0.53 P=0.030
Radiotherapy HR 0.51 P=0.031
Hazard Ratio (log scale)
k
External cohort tumours
Discovery multivariate VLSM map
Low tumour overlap
High tumour overlap
Log-rank P=0.37
Median 9.4 months
Median 11.3 months
Probability of overall survival
Months since diagnosis
Number at risk
63 20 4 0
62 27 6 3
l
External tumours
Discovery multivariate VLSM map
Male sex HR 1.38 P=0.092
Age HR 0.96 P=0.038
Chemotherapy HR 0.87 P=0.508
High VLSM map overlap HR 0.79 P=0.214
Subtotal resection HR 0.52 P=0.025
Radiotherapy HR 0.47 P=0.020
Hazard Ratio (log scale)

Extended Data Fig 1. DMG tumour location mapping by voxel-based lesion symptom mapping (VLSM).

Summed heatmaps of tumour anatomic locations as delineated on patient baseline MRI across the discovery cohort ***(A;*** *n=125****)*** *and for short- and long-term survivors within the discovery cohort* ***(B-C****, respectively****)****. There was no difference in tumour volume between short-term and long-term survivors of DMG (two-tailed Mann Whitney U-test P=0.131). Short-term survivor median tumour volume 25815mm*^{3} *(IQR 20735-33705mm*^{3}*). Long-term survivor median tumour volume 23820mm*^{3} *(IQR 15745-28980mm*^{3}*). Results of mass-univariate lesion location mapping****, both without and with threshold-free cluster enhancement (TFCE),*** *restricted to at least 5% of overlapping voxels between tumours, across DMG in the discovery cohort****, respectively (D-E),*** *expectedly revealed that tumour location within any pontine voxel was associated with shorter overall survival (*$P_{FWE}<0.05$*). When* ***TFCE*** *analyses were repeated restricted to pontine* ***(F)*** *and thalamic* ***(G)*** *DMG, respectively, no voxels significantly associated with shorter overall survival were identified* ***(H****;* $P_{FWE}>0.05$***)****. Tumour location mapping was repeated across the discovery network with multivariate methods, the results of which did not identify any voxels across pontine or thalamic DMG associated with shorter overall survival* ***(H****;* $P_{uncorrected}>0.005$*). The overlap of each tumour in an external, independent validation cohort (n=125 children with DMG) was then computed with each unthresholded discovery VLSM map (univariate and multivariate) to generate a VLSM map overlap score per tumour.* ***(I,K)*** *Kaplan-Meier survival curves of all children in the external validation cohort demonstrate no survival difference between tumours with a high vs. low overlap with the unthresholded discovery cohort univariate or multivariate VLSM map.* ***(J-L)*** *Results of multivariable cox proportional hazards models confirm no prognostic impact of univariate and multivariate VLSM map overlap. Throughout, brain slices are depicted in radiological orientation.*

- For the analysis of tumor growth into the DMG network, a Mann-Whitney U-test was performed to compare tumor volume within vs. outside of the network. This appears to be the incorrect statistical test since these are dependent measures (i.e. for each patient, the tumor growth inside the network is being compared against the tumor growth outside of the network). A signed rank test would be more appropriate for dependent samples. This is also relevant for the analysis of the spinal DMG cohort.

Thank you for highlighting this error. We have now implemented signed rank testing for both tumour volumetric analyses, updating both figures and methods as below. Importantly, results remain supportive of the fact that in-DMG-network tumour growth is significantly greater than out-of-DMG-network tumour growth.

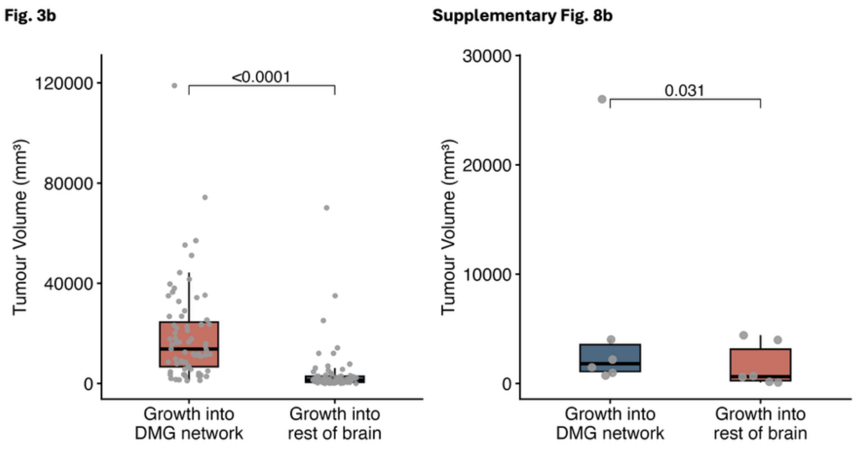

- For the analysis assessing concordance of functional connectivity and tumor burden per node, the text states that "the voxelwise degree of tumour overlap with each of the 18 DMG network nodes was computed for each tumour progression map, defined as the percent overlap of tumour volume relative to the volume of each network node mask". It is unclear whether this means that the overlap was computed as the percentage of the tumor that occupied each node, or as the percentage of each node that was occupied by each node. These are distinct measures, and so it should be clear which is being used.

- For the analyses referenced in the above comment, the text states that the connectivity values were distance-corrected, but it does not describe how this correction was performed. This should be clearly stated in the text.

Thank you for highlighting this ambiguity. We address both reviewer comments below.

We computed tumour burden as the volumetric percentage of each tumour that occupied each node whilst functional connectivity values between each DMG network node and each tumour were divided by the Euclidian distance (mm) between each node centroid and each tumour. This has now been clarified in the manuscript methods, as below:

"The voxelwise degree of tumour overlap with each of the 18 DMG network nodes was computed for each tumour progression map, defined as the percent overlap of tumour volume relative to the volume of each network node mask. Simultaneously, mean node-to-tumour functional connectivity values were computed from each patient-specific functional connectivity map. ***These mean functional connectivity values were then distance-corrected by division by the Euclidian distance (mm) from the centroid of each DMG network node to each baseline tumour****. Intra-individual Spearman's correlation of distance-normalised functional connectivity values and tumour burden per node were computed and averaged for group-level inference. Group-level 95% CI were obtained via bootstrapping with 2000 iterations."*

- The authors seem to use at least 2 different parcellations (e.g. MINCI 60MB SynthSeg and Schaefer-Yeo 400 region parcellation + Brainstem Navigator). It's not clear why a single parcellation was not used. Also, does the Schaefer-Yeo + Brainstem Navigator parcellation include parcels for subcortical regions other than the brainstem?

We agree with the reviewer that use of one, rather than two, parcellations would be more intuitive. MINCI 60MB SynthSeg parcellations were used for tumour growth and neurometabolic development analyses (Main Text Figures 3 and 5) whilst Schaefer-Yeo 400 and Brainstem Navigator parcels were adopted as part of open access code to perform brain-wide dominance analyses for chemoarchitectural characterisation of the DMG network (https://github.com/netneurolab/hansen_brainstemfc/; Main Text Figure 4). Upon review of our methods, we can confirm that the combined Schaefer-Yeo 400 and Brainstem Navigator parcels used for chemoarchitectural dominance analysis did not include subcortical areas other than the brainstem and diencephalon – a significant limitation given the anatomic location of DMG and spatial topography of the DMG network which we are grateful to the reviewer for raising.

To address this limitation, we have now performed a dominance analysis for the DMG network against whole-brain maps of the same 18 neurotransmitter receptor and transporter densities without parcellation (https://github.com/netneurolab/neuromaps). In doing so, we ensure the inclusion of all cortical and subcortical brain regions within our analysis. Results of this revised analytic approach largely replicated our original results, confirming that DMG network spatial topography is characterised by combinatorial cholinergic and serotonergic signalling. However, our results now align even more strongly with preclinical evidence for the influence of neuromodulatory neurotransmitters on DMG growth, exhibiting chemoarchitectural convergence to specific receptor subtypes known to modulate DMG growth (serotonergic 5HT2a – Drexler *et al*. *bioRxiv*. 2026.; and cholinergic M1 – Drexler *et al*. *Cell*. 2025.). In addition, and as the reviewer notes, this approach has greatly simplified the parcellation scheme used throughout our manuscript as all analyses now employ only MINCI 60MB SynthSeg. We have updated the manuscript Methods, Results, and Main Text Figure 4k-l to reflect this, as below.

Manuscript methods

"Chemoarchitectural dominance analyses

*The chemoarchitectural profile of the DMG network was investigated by leveraging publicly available data representing the receptor and transporter densities of 18 unique neurotransmitter receptors and transporters derived from a PET atlas of nine human neurotransmitter systems (https://github.com/netneurolab/**neuromaps**).[70,71] These data were used to model the **voxelwise** brain-wide distribution of human neurotransmitter receptors and transporters and to identify which neurotransmitter receptor and transporter density data best characterised the DMG network. **To this end, the DMG network and 18 neurotransmitter receptor and transporter density maps were re-sampled to 3mm voxel space and masked to include only grey matter brain regions, ensuring consistent resolution and data availability across maps.**[112,113] A multiple linear regression model was then fit to the neurotransmitter receptor and transporter density data to predict connectivity strength across the DMG network. The dominance of each neurotransmitter density profile to this overall model fit was then assessed by fitting this same regression model to every possible combination of input variables. The total dominance for each neurotransmitter was reported, which is defined as the relative increase in R2adj when adding the same neurotransmitter data to each input variable combination, and represents the relative contribution of each neurotransmitter receptor and transporter distribution to the distribution of DMG network connectivity strength.[70] To compare the connectivity profile of short-term and long-term survivors to brainstem neuromodulatory nuclei, mean nucleus-to-tumour connectivity values were computed and compared using a one-way ANOVA with Tukey's post-hoc test (factor neurotransmitter system with four levels: cholinergic, serotonergic, noradrenergic, and dopaminergic)."*

Manuscript main text

*"Dominance analyses identified serotonergic signalling**, in particular via 5HT$_{2A}$**, as the neurotransmitter that aligned best with DMG network topography (>20% model fit; Fig. 4k-l). Importantly, cholinergic signalling **via M1 muscarinic receptors** provided the **second** greatest individual contribution to DMG network chemoarchitecture (**5.1%** model fit; Fig. 4k-l)."*

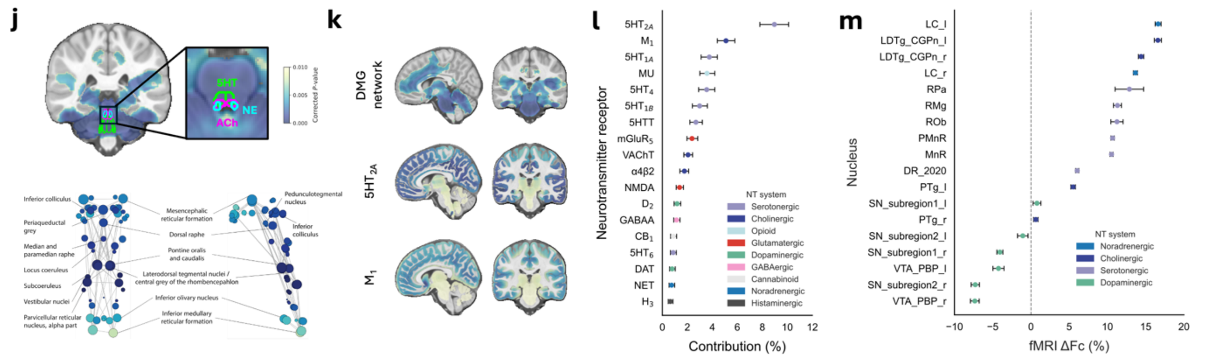

*Fig. 4j-m. **(J)** Spatial representation of brainstem nuclei DMG network connectivity strength. Network peaks are depicted in dark blue. For visualisation, only the strongest 5% of brainstem nucleus-nucleus functional connections are shown. **(K)** Top **two** brain-wide neurotransmitter receptor and transporter density distributions with greatest contribution to DMG network chemoarchitecture. **(L)** Percentage neurotransmitter contribution to the overall fit of a multiple linear regression model between summed DMG network functional connectivity across the brain and 18 neurotransmitter receptor and transporter density profiles (R^2_{adj}=**0.44**). **(M)** Pairwise comparison of nucleus-to-tumour connectivity across short- and long-term survivors of DMG demonstrated significantly greater connectivity to cholinergic (P_{FWE}=0.001), serotonergic (P_{FWE}=0.0002), and noradrenergic (P_{FWE}=0.0004) nuclei than dopaminergic nuclei in short-term survivors (one-way ANOVA F-statistic=16.84, P=6.40e^{-05} with Tukey's post-hoc test; factor neurotransmitter system with four levels).*

- The Statistical Analyses section states that sample sizes are reported for all analyses, but this is not always the case (at least, the sample sizes are not always clear from the text). Please make sure that sample sizes are clear from the text, and that it is also clear which samples are used for which analyses (e.g. there are portions where it is unclear whether an analysis is performed in the discovery cohort vs. one or more of the independent validation cohorts).

We have revised the manuscript main text and methods to ensure that sample sizes are reported throughout, typically in figure legends.

- Extended Data Fig. 3 – the color bar for panel (N) seems inconsistent with the colormap.

Thank you for raising this ambiguity. The blue colour bar is a correct representation of the thresholded primary DMG network. The orange overlays represent stepwise increases in statistical threshold. We have added a second colour bar to panel (N) to clarify this, as below.

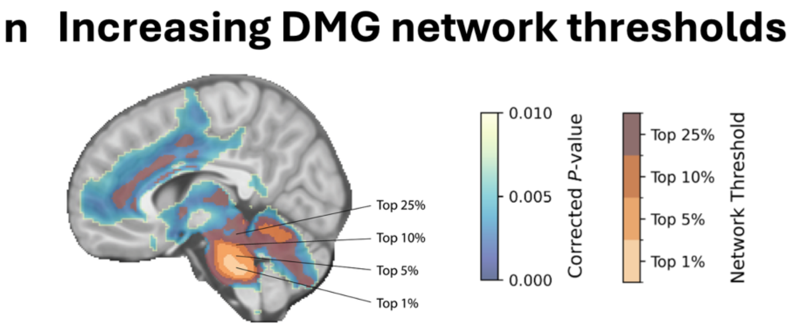

- The network overlays shown in Extended Data Fig. 5 appear to be thresholded, but the labels say they are unthresholded.

All network overlays presented in Extended Data Fig. 5 are unthresholded TFCE maps.

Reviewer comments & author response:
'A prognostic human brain network for diffuse midline glioma'

Reviewer reports

Referee #1 (Remarks to the Author):

The further defining of DIPG and DMG has improved the interpretability of this paediatric report. I remain concerned by some answers to comments such as "We thank the reviewer for their recognition of the extensive analyses presented in this manuscript and agree that the data presented do not provide causal evidence for the hypothesis that neural activity modulates tumour growth in humans." I would prefer if this was more clear throughout the manuscript as the tone still remains too conclusive of a causal connection, despite sentences such as 'Finally, none of the experiments presented herein demonstrate nor could have demonstrated a direct relationship between tumour neural activity and tumour growth in children.' in the opinion of this reviewer.

Referee #2 (Remarks to the Author):

My comments were addressed in the previous round. The authors have added a further statement appropriately highlighting the correlative rather than causal nature of the work.

Referee #3 (Remarks to the Author):

I co-reviewed this manuscript with one of the reviewers who provided the listed reports.

Referee #5 (Remarks to the Author):

The authors have thoroughly addressed my concerns. I list some minor edits the authors may wish to incorporate.

I do not feel the need to review revisions made in response to these comments.

Minor Comments

- For the independent validation analyses using Cox proportional hazards models, it should also be stated which nonparametric CI was computed (e.g. percentile, bias-corrected and accelerated, etc.), and how the reported p-value (p=0.002) was obtained.

- The updated methods state that the SVR-LSM analysis was thresholded at a corrected p<0.005, but the updated figure included in the response shows that it was thresholded at an uncorrected p<0.005. This should be corrected in the text.

- P.7 – Line 274 – "It has been used to successfully identify causative brain networks and therapeutic targets for both canonical circuit disorders…". Lesion-network mapping is capable of identifying normative functional connections that are statistically associated with the outcome of interest, but in most if not all cases it is not sufficient to establish a causal relationship between the entire identified network and the outcome of interest. I would recommend hedging this phrasing by saying something like "…identify brain networks associated with…". These are ultimately associative analyses applied to observational data, and so I think the results should be interpreted from that perspective.

- P.8 – "Greater tumour volume was not associated with patient overall survival, (univariate cox hazard ratio 1.00; P=0.569), in keeping with previous clinical reports, and so tumour network mapping analyses were performed controlling for volume, in line with previous work.". Typically, you want to control for things that you expect to influence the outcome of interest, so the way that this is worded is somewhat strange. I think it's fine to covary for tumor size, but the wording here makes it sound like this was done specifically because there was not a significant relationship between tumor volume and survival.

- Figure 1B should probably be updated to reflect that the VLSM analyses were not completely null.

- Unless I am misunderstanding something, it seems like it would not be possible to conclude that the average of the SyN deformation field would represent the “mean tumor-associated anatomic distortion”. In the registration step, the tumor itself was

excluded using cost-function masking, and many factors other than the tumor could lead the registration to require substantial deformations (e.g. head size, which would likely at least partially reflect age and sex) unless linear registrations were also used prior to performing SyN. I think that the rationale behind this should be explained in more detail.

- Methods – “Although spin permutation tests are reported to inflate P values and output more liberal statistical estimates due to preserved spatial autocorrelation between null models”. My understanding is that “spatially constrained null models” (e.g. spin permutation tests) are actually more conservative and less likely to inflate p-values than spatially naïve null models, in part because they preserve spatial autocorrelation, but that they can still exhibit high false positive rates (e.g. https://doi.org/10.1016/j.neuroimage.2021.118052). It’s not clear what spin permutation tests are being compared to in the quoted excerpt, but if it’s spatially naïve permutation tests, then I believe the excerpt is misrepresenting the cited papers.

- Methods – “ Interrogating predictive accuracy of patient overall survival by DMG network-to-tumour functional connectivity”. This section still refers to the repeated 60/40 splits as “stratified five-fold cross-validation”. A single five-fold cross-validation would split the data into 5 folds, with 4 folds used for model training and the 5th fold used for model testing (i.e. an 80/20 split). What is described in the text is 5 repetitions of a 60/40 hold-out cross-validation. “Stratified cross-validation” typically means that the folds themselves are stratified according to some variable (e.g. the outcome variable) to ensure consistent representation of that variable across folds, but that does not appear to be done here. The text should be revised accordingly.

- Methods – “The voxelwise degree of tumour overlap with each of the DMG network nodes was computed for each tumour progression map, defined as the percent overlap of tumour volume relative to the volume of each network node mask”. I still find this wording confusing. Does this mean that it is defined as the proportion of each DMG network node that intersects each tumor?

- Methods – Several portions of the methods state that analyses were corrected for multiple comparisons without specifying how they were corrected (e.g. P. 30, lines 1165-1166). The specific method used to correct for multiple comparisons should be reported for each analysis.

- Figure 5 – I’m not sure that I understand why there are separate rho values provided for “whole-brain rho” vs. “spin rho”. Does this simply reflect one test using voxels and the other using parcels, and so separate spatial correlations are computed using voxels vs. parcels?

- Despite the moderate-to-high spatial correlations for maps shown in Supplemental Figures 3-5, there appear to be some potentially meaningful differences in the cortical areas represented across maps. It might be helpful to include something like frequency maps showing how often each region was identified across analyses so that the reader can better evaluate which regions are most robust to methodological variations.

Author response to reviewer comments

We sincerely thank the editor and reviewers for their continued engagement and positive feedback. We are grateful for the opportunity to incorporate further textual adjustments in response to this feedback. In this revised submission, we have carefully reviewed the manuscript text and revised sentences that could be interpreted as implying a causal relationship. In doing so, we more clearly emphasise the correlative nature of our findings. Requested statistical clarifications are incorporated in full.

Below, we provide a detailed, point-by-point response to all reviewer comments. Specific changes to the manuscript text are quoted and highlighted in **bold** ink.

Referee #1 (Remarks to the Author):

The further defining of DIPG and DMG has improved the interpretability of this paediatric report.

We thank the reviewer for their time and for their constructive evaluation of our work. We are pleased that the clear distinction between DIPG and DMG included in the revised manuscript has improved the interpretability of the presented data and are grateful for this suggestion.

I remain concerned by some answers to comments such as "We thank the reviewer for their recognition of the extensive analyses presented in this manuscript and agree that the data presented do not provide causal evidence for the hypothesis that neural activity modulates tumour growth in humans." I would prefer if this was more clear throughout the manuscript as the tone still remains too conclusive of a causal connection, despite sentences such as 'Finally, none of the experiments presented herein demonstrate nor could have demonstrated a direct relationship between tumour neural activity and tumour growth in children.' in the opinion of this reviewer.

Thank you for this recommendation. We have carefully worked through and reviewed the manuscript text to identify and revise language implying causality. In doing so, the text more clearly emphasises the correlative nature of our findings. Specific textual changes are listed below:

**Manuscript section**	**Previous manuscript draft**	**Revised manuscript**
**Introduction, text**	Leveraging patient clinical data and human paediatric connectomic data, here we define the brain-wide functional and structural connectivity profile of DMG. In this manner, we establish network-to-tumour functional connectivity as an independent predictor of DMG patient overall survival and delineate circuit-specific trajectories of DMG tumour growth.	*Leveraging patient clinical data and human paediatric connectomic data, here we define the* ***spatial topography of brain network connections associated with short-term survival in children with DMG and delineate circuit-specific trajectories of DMG tumour growth.***
**Results, text**	Lesion network mapping is a computational neuroimaging method which harnesses population-level (n=1000) connectomic data to model common brain network connections across different lesion locations.[29–31] It has been used to successfully identify causative brain networks and therapeutic targets for both canonical circuit disorders,	*"Lesion network mapping is a computational neuroimaging method which harnesses population-level (n=1000) connectomic data to model common brain network connections across different lesion locations.[29–31] It has been used to successfully identify brain networks and therapeutic targets* ***associated with*** *canonical circuit disorders,*

	including focal and generalised epilepsy[32,33], as well as less canonical circuit disorders including addiction and depression.[34,35]	*including focal and generalised epilepsy[32,33], as well as less canonical circuit disorders including addiction and depression.[34,35]"*
**Results, text**	These results indicate that, though DMG exhibit connectivity with constitutively active, hub-like brain regions, the prognostic value of the DMG network cannot be attributed to connectome structure but rather a disease-specific prognostic circuit.	*These results indicate that, though DMG exhibit connectivity with constitutively active, hub-like brain regions, the prognostic value of the DMG network **is insufficiently explained by** connectome structure **and instead reflects a** disease-specific prognostic circuit.*
**Results, subheading**	Tumour progression exhibits directionality to DMG network nodes	*Tumour progression **maps to DMG network architecture***
**Results, text**	These observations, and preclinical evidence for the robust regulation of DMG growth by diverse synaptic inputs[3,6], including neuromodulatory inputs[5,9,18], raised the question of whether the DMG network represents an organised functional architecture composed of specific neurotransmitter projections – or more spuriously connected brain areas hijacked during tumour development.	*These observations, and preclinical evidence for the robust regulation of DMG growth by diverse synaptic inputs[3,6], including neuromodulatory inputs[5,9,18], raised the question of whether the DMG network represents a functional architecture composed of specific neurotransmitter projections.*
**Results, text**	As cortical functional topographies demonstrably align with projections from brainstem neuromodulatory nuclei, we next sought to define the underlying chemoarchitecture of the DMG network and the influence of brain-wide neurotransmitter receptor and transporter density on network topography.	*As cortical functional topographies demonstrably align with projections from brainstem neuromodulatory nuclei, we next sought to define the underlying chemoarchitecture of the DMG network and the **spatial alignment** of brain-wide neurotransmitter receptor and transporter density **with** network topography.*
**Results, text**	Collectively, these results indicate that tumour connectivity with the DMG network is not merely opportunistic but occurs within the context of an organised chemoarchitectural niche which DMG are known to exploit.	***Taken together**, these results **suggest** that tumour connectivity with the DMG network occurs within a chemoarchitectural niche which DMG are known to exploit.*

Referee #2 (Remarks to the Author):

My comments were addressed in the previous round. The authors have added a further statement appropriately highlighting the correlative rather than causal nature of the work.

We thank the reviewer for their careful evaluation of the revised manuscript and for their positive feedback.

Referee #3 (Remarks to the Author):

I co-reviewed this manuscript with one of the reviewers who provided the listed reports.

We thank the reviewer for their time and thoughtful consideration of our work.

Referee #5 (Remarks to the Author):

The authors have thoroughly addressed my concerns. I list some minor edits the authors may wish to incorporate.

I do not feel the need to review revisions made in response to these comments.

We thank the reviewer for their thorough and detailed review of our work. Methodologic suggestions were extremely helpful in strengthening the manuscript and we are pleased that our revised manuscript has addressed these concerns.

Minor Comments

- For the independent validation analyses using Cox proportional hazards models, it should also be stated which nonparametric CI was computed (e.g. percentile, bias-corrected and accelerated, etc.), and how the reported p-value (p=0.002) was obtained.

We have revised the manuscript methods section as below to clarify these methodologic details.

"To confirm out-of-sample prediction, we fitted clinical-only and combined clinical-connectivity multivariable cox proportional hazards models exclusively on the discovery cohort, incorporating patient age and sex and tumour location, DMG network-to-tumour connectivity, extent of resection, and adjuvant radiotherapy and chemotherapy. Model coefficients were subsequently frozen and the fully parameterised models applied to generate overall survival estimates across the independent external validation cohort. Prognostic discrimination was quantified using the ***Harrell*** *concordance index (C-index)* ***calculated from model-derived risk scores****. The incremental predictive value of network-to-tumour connectivity was assessed as the difference in C-index between combined and clinical-only models* ***(ΔC-index) across the external validation cohort****. ΔC-index and the statistical uncertainty around ΔC-index was estimated using* ***paired*** *non-parametric bootstrap resampling of patients within the external validation cohort with 2000 iterations.* ***A 95% confidence interval was calculated using the percentile bootstrap method. Two-sided P-values were derived from the bootstrap distribution of ΔC-index relative to the null hypothesis ΔC-index=0.****"*

- The updated methods state that the SVR-LSM analysis was thresholded at a corrected p<0.005, but the updated figure included in the response shows that it was thresholded at an uncorrected p<0.005. This should be corrected in the text.

Thank you for highlighting this error in Supplementary Figure 1h. Results of SVR-LSM were considered significant at a family-wise error corrected P<0.005, as stated in the methods. Given no voxels survived this statistical threshold, all results were presented at P_{FWE}>0.005. We are sorry that this was incorrectly labelled as $P_{uncorrected}$ within the figure panel. We have updated Supplementary Figure 1h and the associated figure legend to reflect this, as below.

h Multivariate VLSM (P_{FWE} > 0.005)

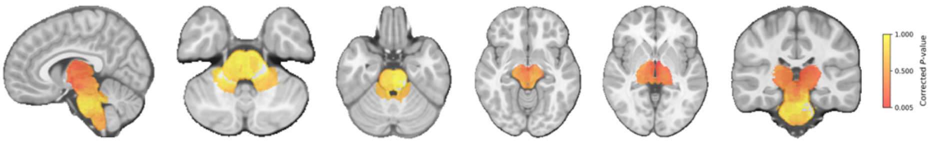

"Tumour location mapping was repeated across the discovery network with multivariate methods, the results of which did not identify any voxels across pontine DMG/DIPG or thalamic DMG associated with shorter overall survival ***(H****; P_{FWE}>0.005).*"

- P.7 – Line 274 – "It has been used to successfully identify causative brain networks and therapeutic targets for both canonical circuit disorders…". Lesion-network mapping is capable of identifying normative functional connections that are statistically associated with the outcome of interest, but in most if not all cases it is not sufficient to establish a causal relationship between the entire identified network and the outcome of interest. I would recommend hedging this phrasing by saying something like "…identify brain networks associated with…". These are ultimately associative analyses applied to observational data, and so I think the results should be interpreted from that perspective.

We agree with the reviewer and have tempered this language to more accurately reflect the associative findings generated by lesion network mapping, as below.

"Lesion network mapping is a computational neuroimaging method which harnesses population-level (n=1000) connectomic data to model common brain network connections across different lesion locations.[29–31] It has been used to successfully identify brain networks and therapeutic targets ***associated with*** *canonical circuit disorders, including focal and generalised epilepsy[32,33], as well as less canonical circuit disorders including addiction and depression.[34,35]"*

- P.8 – "Greater tumour volume was not associated with patient overall survival, (univariate cox hazard ratio 1.00; P=0.569), in keeping with previous clinical reports, and so tumour network mapping analyses were performed controlling for volume, in line with previous work.". Typically, you want to control for things that you expect to influence the outcome of interest, so the way that this is worded is somewhat strange. I think it's fine to covary for tumor size, but the wording here makes it sound like this was done specifically because there was not a significant relationship between tumor volume and survival.

Thank you for raising this. We agree that the wording of this sentence could be improved to better reflect the fact that secondary tumour volume survival analyses were performed to enable comparison with previous clinical studies. We have, therefore, re-written the sentence as below.

"***Tumour volume was included as a covariate in tumour network mapping analyses to account for potential confounding by lesion size, consistent with prior work.[29,32,34] In this cohort, tumour volume was not associated with patient overall survival (univariate cox hazard ratio 1.00; P=0.569), in keeping with prior clinical reports.[36,37]***"

- Figure 1B should probably be updated to reflect that the VLSM analyses were not completely null.

Fig.1b-c are included primarily as methodologic illustrations of VLSM and tumour network mapping for a general scientific audience. A full description and explanation of VLSM results is provided within the manuscript main text results and Extended Data Fig.1.

Throughout Fig.1, results are presented as thresholded maps of statistically significant voxels. For VLSM, this threshold was defined as $P_{FWE}<0.05$. In the primary VLSM analysis, the entire pons was associated with shorter patient overall survival which, as we describe, reflects the well-established difference in survival between children with pontine DMG/DIPG and those with thalamic DMG, rather than localisation to specific anatomic subregions. Consistent with this interpretation, when VLSM was performed separately within pontine and thalamic tumours, no voxels within either structure were associated with overall survival. Similarly, SVR-LSM, which considers the relationship between neighbouring voxels, did not identify any voxels significantly associated with overall survival. Finally, tumour overlap scores derived from univariate VLSM analyses were not associated with survival across independent datasets. Together, these analyses demonstrate that tumour location shows only a broad association with overall survival at the level of primary anatomic location (pontine vs. thalamic). For this reason, VLSM results are illustrated as effectively null in Fig. 1b relative to tumour network mapping results, which remained robust across multiple lines of control and survival analyses.

- Unless I am misunderstanding something, it seems like it would not be possible to conclude that the average of the SyN deformation field would represent the "mean tumor-associated anatomic distortion". In the registration step, the tumor itself was excluded using cost-function masking, and many factors other than the tumor could lead the registration to require substantial deformations (e.g. head size, which would likely at least partially reflect age and sex) unless linear registrations were also used prior to performing SyN. I think that the rationale behind this should be explained in more detail.

Thank you for raising this important point. We take the SyN transform to represent mean peritumoral anatomic distortion across the whole brain rather than intratumoral anatomic distortion. This is supported by the fact that volumetrically larger tumours were associated with greater magnitude mean voxelwise SyN deformation fields (Spearman ρ=0.176; P=0.0491). As the reviewer notes, however, the resulting deformation field could also be influenced by other factors including patient age and head size. We have clarified our definition of peritumoral anatomic distortion and included this potential confound in the manuscript methods, as below:

*"…to further rule out the possibility that tumour-induced neuroanatomical distortions might influence tumour-specific normative functional connections, we also conducted an additional experiment covarying patient overall survival with **a measure of** mean **peritumoral** anatomic distortion. **Whilst also potentially reflective of patient head size, age, and sex, the mean** voxelwise SyN deformation fields required to normalise each patient brain to MNI space **excluding the tumour cost-function mask were used as a measure of mean peritumoral anatomic distortion."***

- Methods – "Although spin permutation tests are reported to inflate P values and output more liberal statistical estimates due to preserved spatial autocorrelation between null models". My understanding is that "spatially constrained null models" (e.g. spin permutation tests) are actually more conservative and less likely to inflate p-values than spatially naïve null models, in part because they preserve spatial autocorrelation, but that they can still exhibit high false positive rates (e.g. https://doi.org/10.1016/j.neuroimage.2021.118052). It's not clear what spin permutation tests are being compared to in the quoted excerpt, but if it's spatially naïve permutation tests, then I believe the excerpt is misrepresenting the cited papers.

Thank you for highlighting this ambiguity. We agree with the reviewer that data support spin permutation testing as a more conservative approach to spatially naïve permutation tests. Rather, we intended to convey that spin permutation tests are more statistically liberal than label permutation tests in which raw input data are permuted to create a null distribution of spatial correlations by randomly shuffling each patient's survival value with a different patient's network map. This is reflected in our data, with spin permutation test results having lower P-values than corresponding label permutation tests. We have revised the manuscript methods to more accurately reflect our intended meaning, as below.

"Both spin and label permutation tests were performed and reported given the widespread use of spin testing despite reports that spin permutation tests likely inflate the statistical significance of spatial similarity between network maps, when compared to label permutation-based approaches."

- Methods – " Interrogating predictive accuracy of patient overall survival by DMG network-to-tumour functional connectivity". This section still refers to the repeated 60/40 splits as "stratified five-fold cross-validation". A single five-fold cross-validation would split the data into 5 folds, with 4 folds used for model training and the 5th fold used for model testing (i.e. an 80/20 split). What is described in the text is 5 repetitions of a 60/40 hold-out cross-validation. "Stratified cross-validation" typically means that the folds themselves are stratified according to some variable (e.g. the outcome variable) to ensure consistent representation of that variable across folds, but that does not appear to be done here. The text should be revised accordingly.

This text was incorrectly included in the previous manuscript revision and does not reflect our current methodologic approach for confirming out-of-sample prediction. Our current approach does not use cross-validation. Rather, we fit a cox model in the discovery cohort and apply the trained model to predict overall survival in the independent, external validation cohort (fully described in Minor Point 1). We have ensured that this text has been removed from the current manuscript version, thank the reviewer for noting this error, and apologise for its inclusion.

- Methods – "The voxelwise degree of tumour overlap with each of the DMG network nodes was computed for each tumour progression map, defined as the percent overlap of tumour volume relative to the volume of each network node mask". I still find this wording confusing. Does this mean that it is defined as the proportion of each DMG network node that intersects each tumor?

Yes, we define the voxelwise degree of tumour overlap as the proportion (percent overlap) of each DMG network node that intersected each tumour. We have further clarified this sentence in the manuscript methods, using the reviewer's wording which we believe to be more intuitive, as below:

*"The voxelwise degree of tumour overlap with each of the 18 DMG network nodes was computed for each tumour progression map, **defined as the proportion (percent overlap) of each DMG network node that intersected each tumour.**"*

- Methods – Several portions of the methods state that analyses were corrected for multiple comparisons without specifying how they were corrected (e.g. P. 30, lines 1165-1166). The specific method used to correct for multiple comparisons should be reported for each analysis.

We have corrected this specific omission as below and have worked through the manuscript text to ensure that the method of correction for multiple comparisons is clearly stated in all instances.

"*Region-wise and age-wise [18F]FDG-PET signal changes within and out-of-DMG network were then compared in relation to their linear regression model fit with age incidence using a two-tailed Mann-Whitney U-test with **Benjamini-Hochberg** correction for multiple comparisons.*"

- Figure 5 – I'm not sure that I understand why there are separate rho values provided for "whole-brain rho" vs. "spin rho". Does this simply reflect one test using voxels and the other using parcels, and so separate spatial correlations are computed using voxels vs. parcels?

Yes. In line with each method, label permutation tests were performed across voxels whilst spin permutation tests were performed across parcels. We have clarified this in the figure legend, as below, with clear reference to voxels vs. parcels:

"*Peak changes in [^{18F}]FDG-PET signal between ages 5 and 6 years and 10 and 11 years, coinciding with age incidence peaks in DMG, strongly overlap with regions in the DMG network (ages 5-6 years peak changes DMG network **voxelwise** spatial ρ=0.199, label permutation P<0.001, spin spatial ρ=0.512, spin permutation P<0.001; ages 10-11 years peak changes **voxelwise** spatial ρ=0.044, label permutation P<0.001, spin **spatial** ρ=0.179, spin permutation P<0.001).*"

- Despite the moderate-to-high spatial correlations for maps shown in Supplemental Figures 3-5, there appear to be some potentially meaningful differences in the cortical areas represented across maps. It might be helpful to include something like frequency maps showing how often each region was identified across analyses so that the reader can better evaluate which regions are most robust to methodological variations.

Thank you for raising this important consideration. We have implemented these analyses in full with textual changes as below.

Manuscript Methods

"***Finally, the convergence of brain regions identified across all control analyses was also compared through the visualisation of control network overlap maps plotted as overlapping binarised voxels surviving statistical thresholds.***"

Manuscript Results & Extended Data Fig.5

"***Finally, visualisation of thresholded brain regions associated with short-term survival demonstrated convergent topography, particularly across DMG network functional connectivity (Extended Data Fig. 5e-f).***"

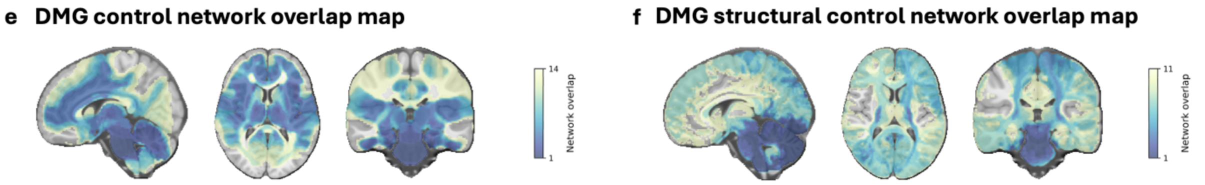

*"**(E-F)** Frequency maps of brain regions significantly associated with shorter patient overall survival across control analyses (**Extended Data Figs.3-4**) for functional connectivity **(E)** and structural connectivity **(F)** data."*
